# Stereodivergent Organocatalytic Intramolecular Michael Addition/Lactonization for the Asymmetric Synthesis of Substituted Dihydrobenzofurans and Tetrahydrofurans

**DOI:** 10.1002/chem.201402684

**Published:** 2014-07-02

**Authors:** Dorine Belmessieri, Alix de la Houpliere, Ewen D D Calder, James E Taylor, Andrew D Smith

**Affiliations:** [a] EaStCHEM, School of Chemistry, University of St. Andrews North Haugh, St. Andrews, KY16 9ST (UK) E-mail: ads10@st.andrews.ac.uk

**Keywords:** asymmetric catalysis, cinchona alkaloid, isothiourea, Michael addition, organocatalysis, oxygen heterocycles, stereodivergent

## Abstract

A stereodivergent asymmetric Lewis base catalyzed Michael addition/lactonization of enone acids into substituted dihydrobenzofuran and tetrahydrofuran derivatives is reported. Commercially available (*S*)-(−)-tetramisole hydrochloride gives products with high *syn* diastereoselectivity in excellent enantioselectivity (up to 99:1 d.r._*syn*/*anti*_, 99 % ee_*syn*_), whereas using a cinchona alkaloid derived catalyst gives the corresponding *anti*-diastereoisomers as the major product (up to 10:90 d.r._*syn*/*anti*_, 99 % ee_*anti*_).

## Introduction

Substituted tetrahydrofurans (THFs) and dihydrobenzofurans are important structural motifs found within many natural products and biologically active molecules.[1] For example, THF and dihydrobenzofuran cores are found within natural-product classes including macrolides,[1a, e] pterocarpans,[1b] acetogenins,[1f] polyether ionophores,[1h] and plant lignans.[1d, i] As a consequence, a large number of asymmetric synthetic methodologies towards both substituted THFs and dihydrobenzofurans has been developed.[2, 3] However, there are relatively few organocatalytic methodologies for the synthesis of either THFs or dihydrobenzofurans.

One strategy that has been utilized for the organocatalytic synthesis of THFs is intramolecular oxy-Michael addition to construct the THF ring.[4] For example, Asano and Matsubara showed that a thiourea–cinchona alkaloid-based bifunctional organocatalyst effectively promotes the asymmetric intramolecular oxy-Michael addition of ε-hydroxy-α,β-unsaturated ketones to form a range of 2-substituted THF derivatives in excellent yield with high levels of enantioselectivity.[4c] More recently, Corbett and Johnson have synthesized highly functionalized bicyclic dialkyl ethers through a diaryl prolinol-catalyzed intermolecular oxy-Michael addition/Michael desymmetrization reaction between *p*-quinols and α,β-unsaturated aldehydes, giving a range of products in high levels of diastereo- and enantioselectivity.[4b] Nicewicz and co-workers have reported an alternative approach to THFs through both intra- and intermolecular organic photocatalytic polar-radical cyclisation reactions between alcohols and alkenes to form a range of substituted THFs with modest levels of diastereoselectivity.[5]

**Scheme 1:**
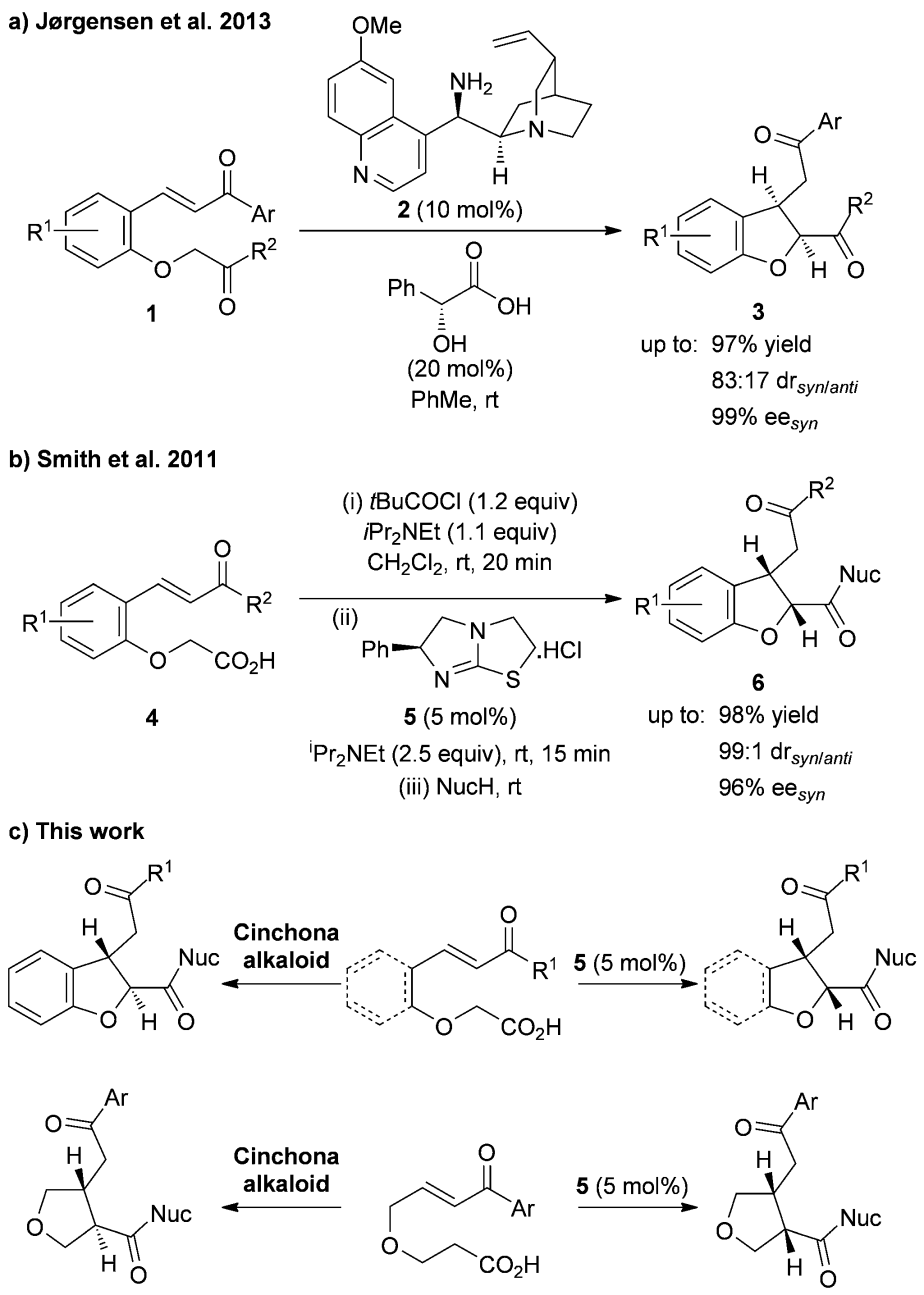
Intramolecular Michael addition/lactonization for the synthesis of *syn*-2,3-dihydrobenzofurans catalyzed by a) cinchona alkaloid derivative 2;[6a] b) (*S*)-(−)-tetramisole hydrochloride 5.[9j] c) Proposed stereodivergent synthesis of 2,3-dihydrobenzofuran, 2,3-THF, and 3,4-THF derivatives.

Substituted dihydrobenzofurans have also been synthesized by using organocatalysis,[6] with both imine- and enamine-based strategies used to construct the dihydrobenzofuran ring stereoselectively. For example, Jørgensen and co-workers reported that mandelic acid salts of primary amino-cinchona alkaloid **2** catalyze the intramolecular cyclisation of aryloxyacetophenones containing a pendant enone **1** to form *syn*-2,3-substituted dihydrobenzofuran derivatives **3** (up to 83:17 d.r._*syn*/*anti*_) in good yield with high enantioselectivity (up to 99 % *ee_syn_*; Scheme 1 a).[6a] Zhou and co-workers have reported that a primary amine/thiourea bifunctional catalyst promotes a related intramolecular cyclisation onto nitro-alkenes, forming *anti*-2,3-dihydrobenzofurans with reasonable levels of diastereoselectivity and high enantioselectivity.[6b] However, considering their synthetic importance, the development of catalytic asymmetric routes towards substituted THF and dihydrobenzofuran derivatives is still a worthwhile goal.

We have previously developed a number of organocatalytic methodologies based on Michael addition/cyclization cascades of Michael acceptors with ammonium enolates generated from isothiourea-based catalysts[7, 8] and carboxylic acids.[9–11] For example, this strategy has been successfully applied to the asymmetric synthesis of dihydropyranones,[9g, j] dihydropyridones,[9i] α-hydrazino esters,[9h] β-lactams,[9b] and more recently, for the synthesis of pyridines[9e] and pyrones.[9c] Of particular relevance is the application of this strategy to the highly diastereo- and enantioselective synthesis of *syn*-2,3-substituted dihydrobenzofurans through (*S*)-(−)-tetramisole hydrochloride **5** catalyzed intramolecular Michael addition/lactonization of in situ activated enone acids **4**, followed by nucleophilic ring-opening (Scheme 1b).[9j] This methodology was subsequently utilized for the stereoselective synthesis of substituted pyrrolidine derivatives using amine-tethered enone/acid substrates.[9f] Herein, the extension of this methodology for the stereoselective synthesis of substituted THFs and dihydrobenzofurans is described. In particular, this manuscript probes the development of a stereodivergent catalyst controlled protocol that allows access to either *syn*- or *anti*-substituted THF and dihydrobenzofuran derivatives from common enone/acid substrates through judicious choice of organocatalyst (Scheme 1 c).

## Results and Discussion

### Optimization of *anti*-dihydrobenzofuran synthesis

Having previously reported an efficient synthesis of *syn*-2,3-substituted dihydrobenzofurans catalyzed by (*S*)-(−)-tetramisole hydrochloride **5**,[9j] initial studies looked to develop complementary methodology to access the corresponding *anti*-2,3-substituted dihydrobenzofurans. Enone acid **7**, readily prepared from salicylaldehyde in two steps, was chosen as a model substrate.[12] Because cinchona alkaloids had previously been shown to give opposite diastereoselectivity compared with isothiourea catalysts in the synthesis of pyrrolidine derivatives,[9f] a range of cinchona derivatives was synthesized and tested in the intramolecular Michael addition/lactonization (Table 1).[13] Enone acid **7** was added at room temperature to a mixture of quinine **12** (20 mol %), Mukaiyama reagent derivative **8** (1.5 equiv) and *i*Pr_2_NEt (2.5 equiv) in CH_2_Cl_2_ and stirred at room temperature for 1 h before addition of MeOH.[14] Pleasingly, *anti*-dihydrobenzofuran **11** was obtained as the major diastereoisomer (36:64 d.r._*syn*/*anti*_) in a promising 81 % *ee_anti_*, but in only 35 % combined yield (Table 1, entry 1).[15] Using quinine derivatives with protected hydroxyl groups (Me **13**, Ac **14**, Bn **15**) as catalysts led to an improvement in *ee* (up to 97 % *ee_anti_*) but no significant change in diastereoselectivity or yield (Table 1, entries 2–4). However, by using OTMS-quinine **16** (TMS=trimethylsilyl) as the catalyst further increased the diastereoselectivity in favor of *anti*-**11** (27:73 d.r._*syn*/*anti*_), which was also obtained in an excellent 96 % *ee_anti_* and 76 % combined yield (Table 1, entry 5). The use of OTMS-cinchonidine **17** and pseudoenantiomeric OTMS-cinchonine **18** led to a reduction in yield, but using OTMS-quinidine **19** gave a further increase in diastereoselectivity (20:80 d.r._*syn*/*anti*_) (Table 1, entries 6–8). Performing the reaction with OTMS-quinidine **19** at 0 °C improved the enantioselectivity (99 % *ee_anti_*), but significantly reduced the combined yield to 30 % (Table 1, entry 9). Finally, by using a catalytic amount of 4-dimethylaminopyridine (DMAP) during the final ring-opening step with MeOH improved the combined isolated yield to 62 % with the major diastereoisomer *anti*-**11** (20:80 d.r._*syn*/*anti*_) obtained in 98 % ee_*anti*_ and the minor diastereoisomer *syn*-**10** obtained in 63 % ee_*syn*_ (Table 1, entry 10).[16, 17]

**Table 1:** Reaction optimization for the synthesis of *anti*-2,3-dihydrobenzofuran 11

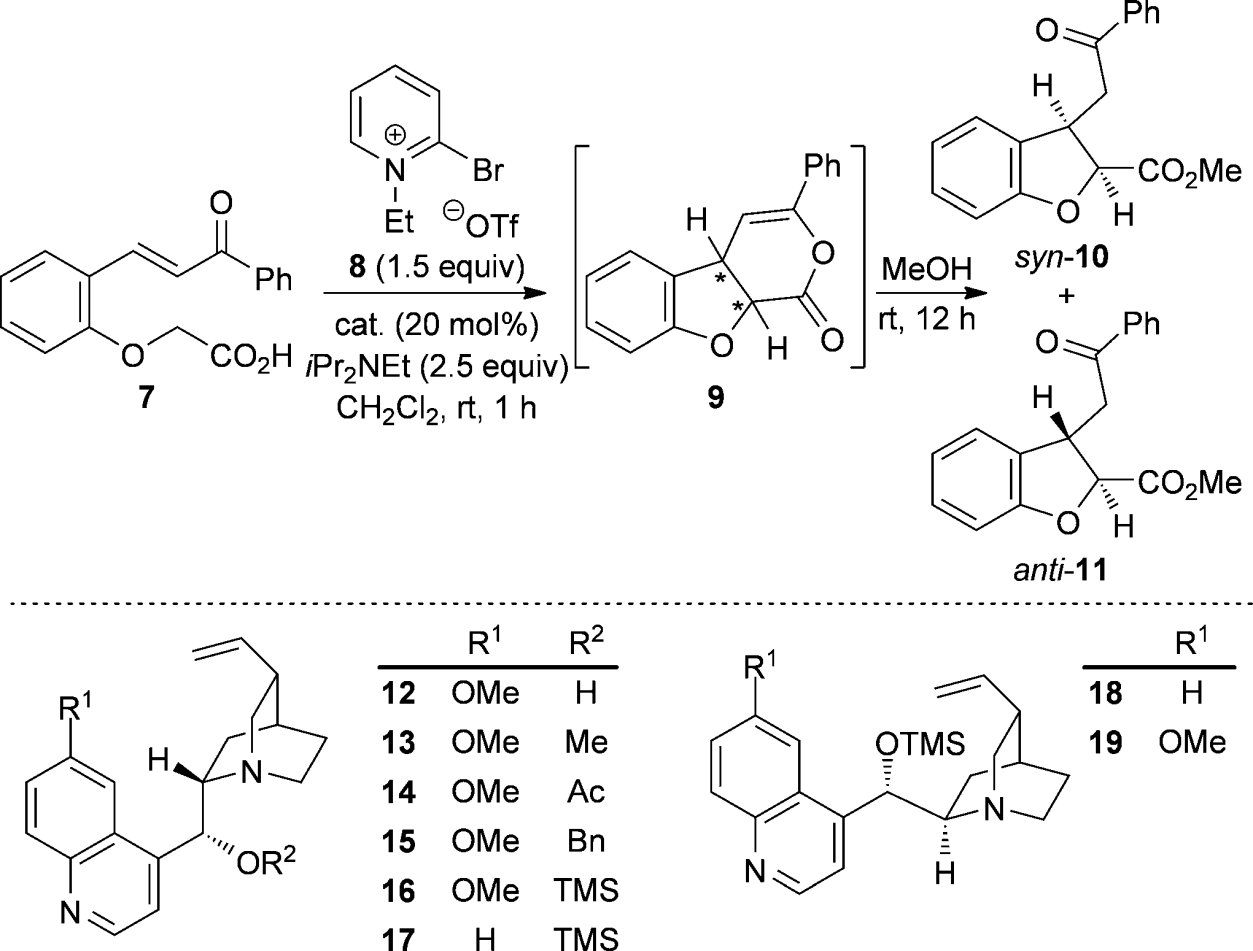					
Entry	Catalyst	Yield [%]^[a]^	d.r._*syn*/*anti*_^[b]^	*ee_syn_* [%]^[c]^	*ee_anti_* [%]^[c]^
1	**12**	35	36:64	35 (*ent*)	81 (*ent*)
2	**13**	41	42:58	45 (*ent*)	97 (*ent*)
3	**14**	30	33:67	52 (*ent*)	92 (*ent*)
4	**15**	51	32:68	64 (*ent*)	95 (*ent*)
5	**16**	76	27:73	55 (*ent*)	96 (*ent*)
6	**17**	49	36:64	20 (*ent*)	84
7	**18**	51	24:76	42	95
8	**19**	50	20:80	69	96
9^[d]^	**19**	30	20:80	59	99
10^[e]^	**19**	62	20:80	63	98

[a] Combined isolated yield of both diastereoisomers. [b] Determined by ^1^H NMR analysis of the crude reaction product. [c] Determined by HPLC analysis. [d] Reaction performed at 0 °C. [e] Ring opening with MeOH and DMAP (cat.).

### Scope of 2,3-dihydrobenzofurans and 2,3-tetrahydrofurans

The scope of the stereodivergent 2,3-substituted dihydrobenzofuran synthesis was then investigated, comparing the (*S*)-(−)-tetramisole hydrochloride **5** promoted *syn*-dihydrobenzofuran methodology with the newly optimized OTMS-quinidine **19** promoted *anti*-dihydrobenzofuran methodology (Table 2). Firstly, the scope of the *syn*-dihydrobenzofuran synthesis by using 5 mol % (*S*)-(−)-tetramisole hydrochloride **5** as a catalyst was extended. A range of enone acids was treated with pivaloyl chloride and *i*Pr_2_NEt in CH_2_Cl_2_ to form the corresponding acid anhydrides in situ. After 20 minutes, (*S*)-(−)-tetramisole hydrochloride **5** (5 mol %) and additional *i*Pr_2_NEt were added, and after one hour at room temperature MeOH was added to ring-open the initial polycyclic lactone products.[14] A number of different aryl and alkyl enone substituents was tolerated, forming *syn*-2,3-dihydrobenzofuran products **20**–**25** as single diastereoisomers in good yield (up to 98 %) and excellent *ee* values (up to 99 % *ee_syn_*).[18] In the cases when electron-withdrawing substituents were present (4-CF_3_ **22** and 4-Cl **23**), the enantioselectivity of the reaction was slightly lowered. This process could be performed on a gram scale by using 1 mol % (*S*)-(−)-tetramisole hydrochloride **5**, with 2.0 g of *syn*-dihydrobenzofuran **20** synthesized as a single diastereoisomer in excellent *ee* (95 % yield, 99:1 d.r._*syn*/*anti*_, 99 % *ee_syn_*).

**Table 2:** Intramolecular Michael addition/lactonization for the synthesis of *syn* and *anti*-2,3-dihydrobenzofurans

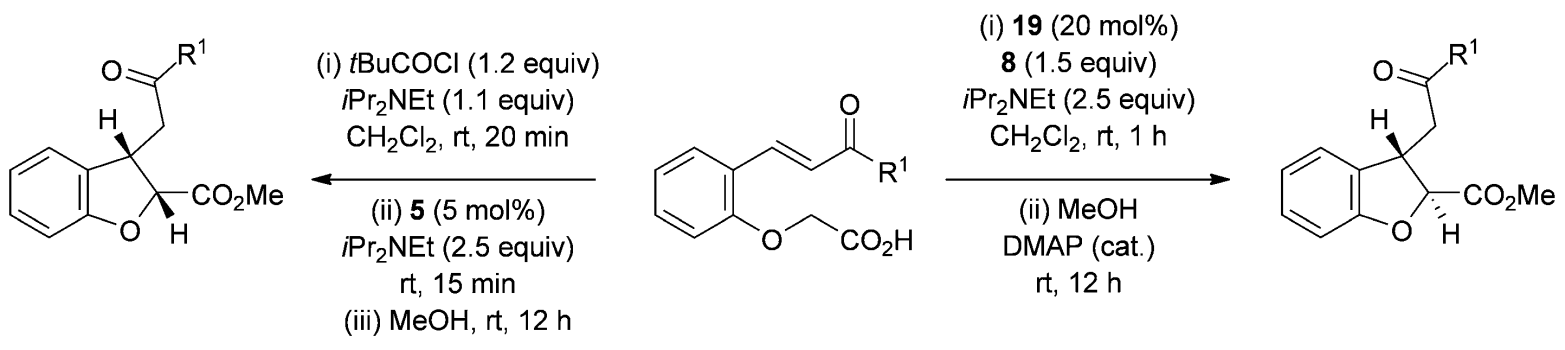							
*syn*-2,3-Dihydrobenzofurans				*anti*-2,3-Dihydrobenzofurans			
Major product	Yield^[a]^ d.r._*syn*/*anti*_^[b]^ *ee_syn_*^[c]^	Major product	Yield^[a]^ d.r._*syn*/*anti*_^[b]^ *ee_syn_*^[c]^	Major product	Yield^[e]^ d.r._*syn*/*anti*_^[b]^ *ee_anti_*^[c]^ (*ee_syn_*)^[c]^	Major product	Yield^[e]^ d.r._*syn*/*anti*_^[b]^ *ee_anti_*^[c]^ (*ee_syn_*)^[c]^
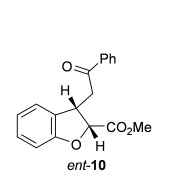	98 %	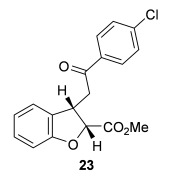	63 %	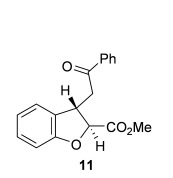	62 %	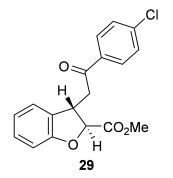	60 %
	>99:1 d.r.		>99:1 d.r.		20:80 d.r.		26:74 d.r.
	94 % *ee*		85 % *ee*		98 % *ee_anti_* (63 % *ee_syn_*)		96 % *ee_anti_* (42 % *ee_syn_*)
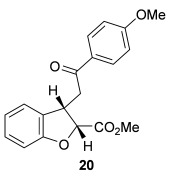	95 %^[d]^	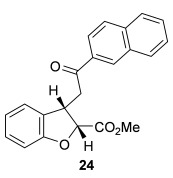	64 %	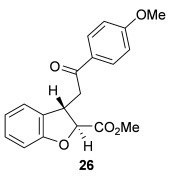	70 %^[f]^	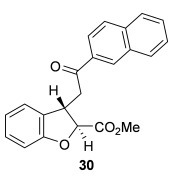	56 %
	>99:1 d.r.		>99:1 d.r.		15:85 d.r.		23:77 d.r.
	99 % *ee*		95 % *ee*		99 % *ee_anti_* (51 % *ee_syn_*)		97 % *ee_anti_* (57 % *ee_syn_*)
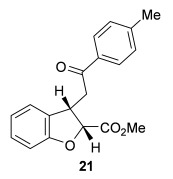	95 %	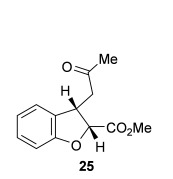	87 %	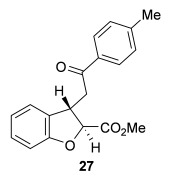	60 %	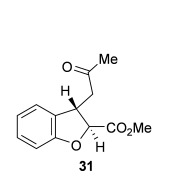	73 %
	>99:1 d.r.		>99:1 d.r.		18:82 d.r.		22:78 d.r.
	94 % *ee*		95 % *ee*		98 % *ee_anti_* (63 % *ee_syn_*)		99 % *ee_anti_* (43 % *ee_syn_*)
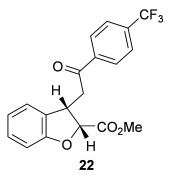	64 %			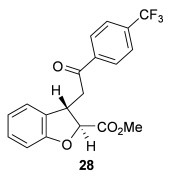	55 %		
	>99:1 d.r.					37:63 d.r.		
	94 % *ee*					94 % *ee_anti_* (48 % *ee_syn_*)		

[a] Isolated yield. [b] Determined by ^1^H NMR analysis of the crude reaction product. [c] Determined by HPLC analysis. [d] 6.40 mmol scale, 1 mol % **5**. [e] Combined isolated yield of both diastereoisomers. [f] 6.40 mmol scale.

Next, the scope of the complementary *anti*-2,3-dihydrobenzofuran methodology by using OTMS-quinidine **19** as the catalyst was investigated (Table 2). The enone acids were reacted under the optimal conditions by using 20 mol % **19** as a catalyst to give *anti*-2,3-dihydrobenzofurans **26**–**31** as the major products after ring-opening with MeOH and catalytic DMAP. Various aryl enone substituents could be incorporated, with the electronic nature of the substituents having a noticeable effect on the diastereoselectivity of the reaction. In the case of electron-donating substituents (4-MeO **26** and 4-Me **27**), the diastereoselectivity was marginally increased (15:85 d.r._*syn*/*anti*_ and 18:82 d.r._*syn*/*anti*_, respectively) compared with **11** (20:80 d.r._*syn*/*anti*_), whereas electron-withdrawing substituents (4-CF_3_ **28** and 4-Cl **29**) resulted in a decrease in diastereoselectivity (37:63 d.r._*syn*/*anti*_ and 26:74 d.r._*syn*/*anti*_, respectively). An alkyl enone substituent was also tolerated, with *anti*-**31** (22:78 d.r._*syn*/*anti*_) formed in 73 % combined yield and excellent 99 % *ee_anti_*. In all cases, the major *anti*-2,3-dihydrobenzofuran products **26**–**31** were obtained with excellent levels of enantiocontrol (up to 99 % ee_*anti*_), whilst the minor *syn*-diastereoisomers were obtained in significantly lower *ee* (42–63 % *ee_syn_*). The *anti*-2,3-dihydrobenzofuran synthesis was also amenable to scale-up, with 1.2 g of *anti*-**26** formed in 70 % isolated yield.

Having successfully developed a stereodivergent synthesis of 2,3-dihydrobenzofurans, the application of this intramolecular Michael addition/lactonization protocol was investigated for the synthesis of 2,3-substituted THFs (Table 3). Firstly, the intramolecular Michael addition/lactonization of (*E*)-2-((5-oxo-5-phenylpent-3-en-1-yl)oxy)acetic acid (R=Ph) by using 10 mol % (*S*)-(−)-tetramisole hydrochloride **5** as a catalyst was studied.[12] Pleasingly, the desired cyclisation worked well, although as before, the initial fused THF proved to be unstable to chromatographic purification. Therefore, benzylamine was added to form ring-opened *syn*-2,3-THF **32** in 70 % yield with excellent levels of diastereoselectivity (98:2 d.r._*syn*/*anti*_) and enantioselectivity (99 % *ee_syn_*).[19] The scope of this process was then investigated by using various substituted linear enone acids. Both aryl and alkyl substituted enones could be utilized, forming the corresponding *syn*-2,3-THFs **33**–**36** in good yield (up to 73 %) with excellent levels of stereoselectivity (up to 98:2 d.r._*syn*/*anti*_, 99 % *ee_syn_*). The initial fused THFs could also be ring-opened with different nucleophiles, including methanol, pyrrolidine, and sodium hydroxide to form the corresponding ester **37**, tertiary amide **38**, and carboxylic acid **39** substituted *syn*-2,3-THFs, respectively.

**Table 3:** Intramolecular Michael addition/lactonization for the synthesis of *syn*-2,3-THFs

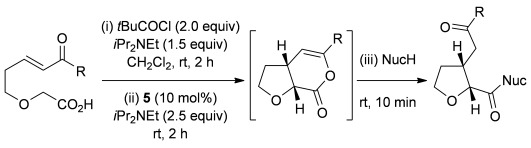			
Major product	Yield^[a]^ d.r._*syn*/*anti*_^[b]^ *ee_syn_*^[c]^	Major product	Yield^[a]^ d.r._*syn*/*anti*_^[b]^ *ee_syn_*^[c]^
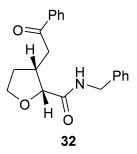	70 %	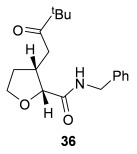	69 %
	>99:1 d.r.		>99:1 d.r.
	>99 % *ee*		98 % *ee*
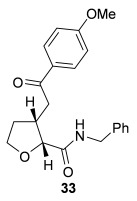	62 %	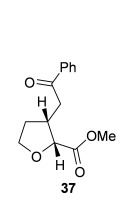	61 %
	>99:1 d.r.		97:3 d.r.
	99 % *ee*		>99 % *ee*
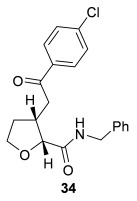	73 %	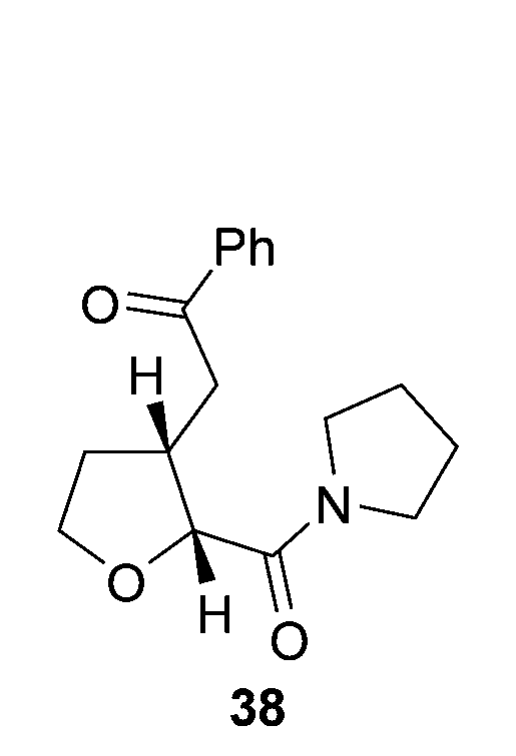	49 %
	>99:1 d.r.		>99:1 d.r.
	99 % *ee*		99 % *ee*
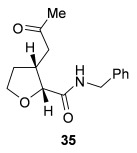	50 %	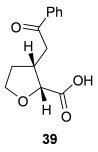	76 %
	73:27 d.r.		>99:1 d.r.
	98 % *ee*		>99 % *ee*

[a] Isolated yield. [b] Determined by ^1^H NMR analysis of the crude reaction product. [c] Determined by HPLC analysis.

**Scheme 2:**
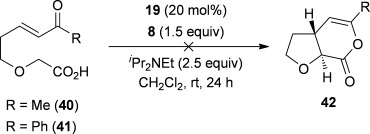
Unsuccessful intramolecular Michael addition/lactonization for the synthesis of *anti*-2,3-THFs.

Next, the synthesis of *anti*-2,3-THFs was investigated by using OTMS-quinidine **19** as a catalyst. However, reaction of enone acid **40** under the optimal conditions found previously gave no conversion into the desired *anti*-2,3-THF **42** even after extended reaction times (Scheme 2). Attempts to promote the cyclisation by increasing the catalyst loading and increasing the temperature also proved to be unsuccessful. The reaction was also unsuccessful when phenyl-substituted enone acid **41** was used, demonstrating that the intramolecular Michael addition/lactonization protocol is limited to the synthesis of *syn*-2,3-THFs by using (*S*)-(−)-tetramisole hydrochloride **5** as the catalyst.

### Synthesis of 3,4-tetrahydrofurans

The position of the oxygen tether within the enone acids was then investigated in an attempt to form 3,4-substituted THF derivatives (Table 4). To investigate this possibility, (*E*)-3-((4-oxo-4-phenylbut-2-en-1-yl)oxy)propanoic acid (Ar=Ph) was treated with pivaloyl chloride and *i*Pr_2_NEt followed by 5 mol % (*S*)-(−)-tetramisole hydrochloride **5**. After one hour, the mixed anhydride intermediate had been fully consumed, and crude ^1^H NMR analysis showed that the desired fused *syn*-3,4-THF **43** was present as a single diastereoisomer (>99:1 d.r._*syn*/*anti*_). In this case, the product was stable to chromatographic purification, allowing fused *syn*-3,4-THF **43** to be isolated in 66 % yield and excellent 98 % *ee_syn_* (Table 4). Fused *syn*-3,4-THF **43** could also be ring opened in situ through addition of benzylamine, forming amide **46** in 69 % yield as a single diastereoisomer (>99:1 d.r._*syn*/*anti*_) with essentially no loss in enantioselectivity (96 % *ee_syn_*). Enones containing either electron-donating (4-OMe) or electron-withdrawing (4-Cl) aromatic substituents were also tolerated in this process, forming either fused *syn*-3,4-THFs (**44** and **45**, respectively) or the corresponding ring-opened products (**47** and **48**, respectively) in good yield with excellent levels of stereocontrol (>99:1 d.r., up to>99 % *ee*).[20]

**Table 4:** Intramolecular Michael addition/lactonization for the synthesis of *syn* and *anti*-3,4-THFs

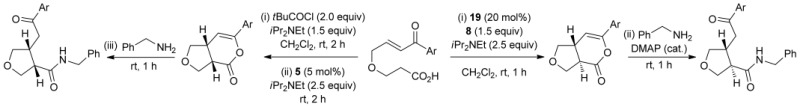							
*syn*-3,4-THFs				*anti*-3,4-THFs			
Major product	Yield^[a]^ d.r._*syn*/*anti*_^[b]^ *ee_syn_*^[c]^	Major product	Yield^[a]^ d.r._*syn*/*anti*_^[b]^ *ee_syn_*^[c]^	Major product	Yield^[d]^ d.r._*syn*/*anti*_^[b]^ *ee_anti_*^[c]^ (*ee_syn_*)^[c]^	Major product	Yield^[d]^ d.r._*syn*/*anti*_^[b]^ *ee_anti_*^[c]^ (*ee_syn_*)^[c]^
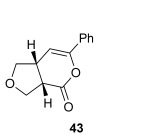	66 %	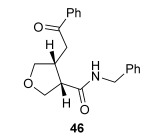	69 %	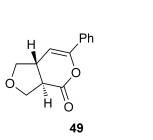	58 %^[e]^	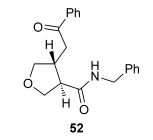	56 %
	>99:1 d.r.		>99:1 d.r.		16:84 d.r.		20:80 d.r.
	98 % *ee*		96 % *ee*		>99 % *ee_anti_* (78 % *ee_syn_*)		>99 % *ee_anti_* (81 % *ee_syn_*)
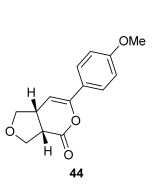	67 %	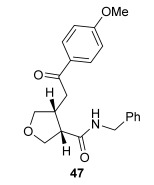	64 %	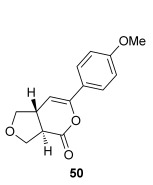	58 %	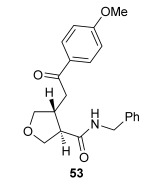	47 %
	>99:1 d.r.		>99:1 d.r.		15:85 d.r.		10:90 d.r.
	99 % *ee*		97 % *ee*		>99 % *ee_anti_* (77 % *ee_syn_*)		>99 % *ee_anti_* (80 % *ee_syn_*)
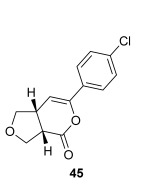	52 %	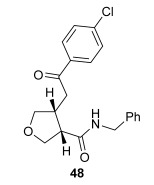	67 %	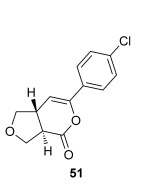	58 %	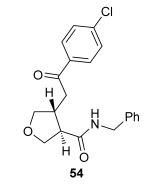	60 %
	>99:1 d.r.		>99:1 d.r.		18:82 d.r.		20:80 d.r.
	94 % *ee*		98 % *ee*		>99 % *ee_anti_* (75 % *ee_syn_*)		>99 % *ee_anti_* (76 % *ee_syn_*)

[a] Isolated yield. [b] Determined by ^1^H NMR analysis of the crude reaction product. [c] Determined by HPLC analysis. [d] Combined isolated yield of both diastereoisomers. [e] Isolated yield of single diastereoisomer.

Pleasingly, treating these linear enone acids with Mukaiyama derivative **8** and 20 mol % OTMS-quinidine **19** gave the complementary fused *anti*-3,4-THFs **49**–**51** with levels of diastereocontrol comparable with those observed in the *anti*-2,3-dihydrobenzofuran products (20:80 to 10:90 d.r._*syn*/*anti*_, Table 4). Fused *anti*-3,4-THF derivatives **49**–**51** were also stable to purification and could be isolated in reasonable yields with excellent levels of enantioselectivity for the major *anti*-diastereoisomers (>99 % *ee_anti_*).[19] As observed previously, the minor *syn*-3,4-THF products were consistently formed in lower *ee* (up to 77 % *ee_syn_*). The fused *anti*-3,4-THF products could also be ring-opened in situ with benzylamine and a catalytic amount of DMAP, forming substituted *anti*-3,4-THFs **52**–**54** in acceptable yield with comparable levels of stereoselectivity with the fused products.

**Scheme 3:**
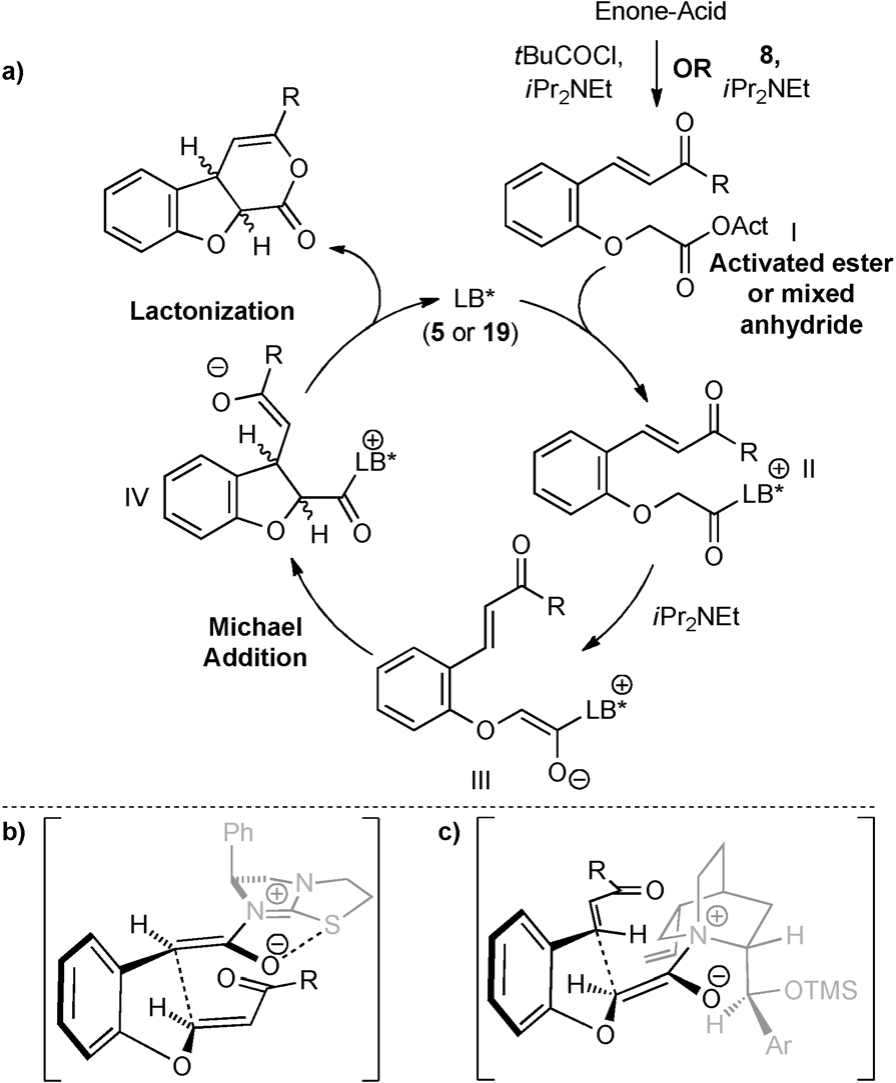
a) Proposed mechanism for Lewis-base (LB*)-catalyzed Michael addition/lactonization of activated enone acids into 2,3-dihydrobenzofurans. b) Proposed pretransition state assembly by using (*S*)-(−)-tetramisole hydrochloride 5 as catalyst. c) Proposed pretransition state assembly by using OTMS-quinidine 19 catalyst.

The proposed catalytic cycle for the synthesis of 2,3-dihydrobenzofurans is shown in Scheme 3 a. Firstly, pivaloyl chloride or Mukaiyama derivative **8** reacted with the enone acid to form a mixed anhydride or activated ester, respectively (represented by generic species **I**) to enable nucleophilic attack from the Lewis-base catalyst (**II**). Deprotonation to form a (*Z*)-ammonium enolate (**III**) followed by intramolecular Michael addition forms the new C–C bond and two stereocenters (**IV**). Finally, lactonization releases the Lewis-base catalyst and the polycyclic product, which can be ring-opened upon addition of a nucleophile. The stereochemical outcome of the (*S*)-(−)-tetramisole hydrochloride **5**-catalyzed reactions can be rationalized through the pretransition-state assembly shown in Scheme 3 b. The enolate oxygen lies in a *syn* conformation to the S atom, allowing either an n_O_ to σ*_C−S_ interaction or favorable electrostatic stabilization.[21] Michael addition onto the Si face of the enone *anti* to the stereodirecting group of the catalyst gives the observed *syn* products. Using OTMS-quinidine **19** as the catalyst the prochiral centers could adopt a conformation in which the two hydrogen atoms are staggered to minimize unfavorable steric interaction with the ethylene bridge within the catalyst, accounting for the *anti* diastereoselectivity observed (Scheme 3 c).[22, 23] An analogous mechanism and stereochemical rationale can be applied to the reactions to form *syn*-2,3-THF, *syn*-3,4-THF, and *anti*-3,4-THF derivatives.

## Conclusion

A stereodivergent synthesis of substituted THF and dihydrobenzofuran derivatives through a Lewis-base-promoted Michael addition/lactonization reaction of enone acids has been developed. The use of (*S*)-(−)-tetramisole hydrochloride **5** as a catalyst selectively gave *syn*-2,3-dihydrobenzofurans, *syn*-2,3-THFs, and *syn*-3,4-THFs with excellent levels of diastereo- and enantioselectivity (up to 99:1 d.r._*syn*/*anti*_, 99 % *ee_syn_*). Alternatively, the use of OTMS-quinidine **19** as a catalyst allowed preferential access to the corresponding *anti*-2,3-dihydrobenzofurans and *anti*-3,4-THF derivatives, with good levels of diastereoselectivity (up to 10:90 d.r._*syn*/*anti*_) and excellent levels of enantioselectivity for the major *anti* diastereoisomers (up to 99 % *ee_anti_*). Ongoing studies within this laboratory are focused on the use of Lewis-base catalysis for the synthesis of other heterocyclic compounds.

## Experimental Section

For general experimental details, full characterization data, NMR spectra, and HPLC traces, see the Supporting Information.

### General procedure for the synthesis of *syn*-2,3-dihydrobenzofurans (10, 20–25)

The desired enone acid (1.0 equiv) and *i*Pr_2_NEt (1.1 equiv) were dissolved in CH_2_Cl_2_ (to give 0.2 m solution of acid) in a flame-dried round-bottomed flask and cooled to 0 °C before *t*BuCOCl (1.2 equiv) was added dropwise. After 20 min, (*S*)-(−)-tetramisole hydrochloride **5** (5 mol %) and *i*Pr_2_NEt (2.5 equiv) were added and the reaction mixture was warmed to RT and stirred for 15 min. MeOH was added, and the reaction stirred for 1 h at RT. The solvent was evaporated, and the crude residue was purified directly by column chromatography on silica gel.

Authentic racemic samples were obtained by using (*rac*)-(±)-tetramisole hydrochloride **5** (5 mol %) as the catalyst.

### General procedure for the synthesis of *anti*-2,3-dihydrobenzofurans (11, 26–31)

OTMS-Quinidine **19** (20 mol %), *i*Pr_2_NEt (2.5 equiv), and Mukaiyama derivative **8** (1.5 equiv) were dissolved in CH_2_Cl_2_ (to give 0.3 m solution of **8**) in a flame-dried round-bottomed flask under an N_2_ atmosphere. A solution of the desired enone acid (1.0 equiv) in CH_2_Cl_2_ (0.4 m) was added dropwise, and the reaction was stirred for 1 h at RT. MeOH and DMAP (cat.) were added, and the reaction was stirred at RT overnight. The solvent was evaporated, and the crude residue was purified directly by column chromatography on silica gel.

Authentic racemic samples were obtained by using 1,4-diazabicyclo[2.2.2]octane (DABCO; 20 mol %) as the catalyst.

### General procedure for the synthesis of *syn*-2,3-THFs (32–39)

The desired enone acid (1.0 equiv) and *i*Pr_2_NEt (1.5 equiv) were dissolved in CH_2_Cl_2_ (to give 0.2 m solution of acid) in a flame-dried round-bottomed flask before *t*BuCOCl (2.0 equiv) was added dropwise. After 2 h, (*S*)-(−)-tetramisole hydrochloride **5** (10 mol %) and *i*Pr_2_NEt (2.5 equiv) were added, and the reaction mixture was stirred for 2 h at RT. The reaction was concentrated under reduced pressure before the desired nucleophile was added and stirred for 10 min. The resulting solution was diluted in CH_2_Cl_2_, washed with 2 m HCl (×2), saturated NaHCO_3_ (×2), and brine, dried over MgSO_4_, filtered, and concentrated under reduced pressure. The crude product was purified by column chromatography on silica gel.

Authentic racemic samples were obtained by using (*rac*)-(±)-tetramisole hydrochloride **5** (5 mol %) as the catalyst.

### General procedure for the synthesis of *syn*-3,4-THFs (43–48)

The desired enone acid (1.0 equiv) and *i*Pr_2_NEt (1.5 equiv) were dissolved in CH_2_Cl_2_ (to give 0.2 m solution of acid) in a flame-dried round-bottomed flask before *t*BuCOCl (2.0 equiv) was added dropwise. After 2 h, (*S*)-(−)-tetramisole hydrochloride **5** (5 mol %) and *i*Pr_2_NEt (2.5 equiv) were added, and the reaction mixture was stirred at RT. After 2 h, the reaction was concentrated under reduced pressure and purified directly by chromatography column on silica gel. Alternatively, the desired nucleophile was added, and the reaction stirred for 10 min. The resulting solution was diluted in CH_2_Cl_2_, washed with 2 m HCl (×2), saturated NaHCO_3_ (×2), and brine, dried over MgSO_4_, filtered, and concentrated under reduced pressure. The crude product was purified by column chromatography on silica gel.

Authentic racemic samples were obtained by using (*rac*)-(±)-tetramisole hydrochloride **5** (5 mol %) as the catalyst.

### General procedure for the synthesis of *anti*-3,4-THFs (49–54)

OTMS-Quinidine **19** (20 mol %), *i*Pr_2_NEt (2.5 equiv), and Mukaiyama derivative **8** (1.5 equiv) were dissolved in CH_2_Cl_2_ (to give 0.3 m solution of **8**) in a flame-dried round-bottomed flask under an N_2_ atmosphere. A solution of the desired enone acid (1.0 equiv) in CH_2_Cl_2_ (0.4 m) was added dropwise, and the reaction was stirred at for 1 h at RT. The reaction was concentrated under reduced pressure and purified directly by chromatography column on silica gel. Alternatively, benzylamine (5.0 equiv) and DMAP (cat.) were added, and the reaction stirred for 1 h at RT. The resulting solution was diluted in CH_2_Cl_2_, washed with 1 m HCl (×2), saturated NaHCO_3_ (×2), and brine (×2), dried over MgSO_4_, filtered, and concentrated under reduced pressure. The crude product was purified by column chromatography on silica gel.

Authentic racemic samples were obtained by using DABCO (20 mol %) as the catalyst.

## Supporting Information

As a service to our authors and readers, this journal provides supporting information supplied by the authors. Such materials are peer reviewed and may be re-organized for online delivery, but are not copy-edited or typeset. Technical support issues arising from supporting information (other than missing files) should be addressed to the authors.

miscellaneous_information
