# Supplementary material for: Stereodivergent Organocatalytic Intramolecular Michael Addition/Lactonization for the Asymmetric Synthesis of Substituted Dihydrobenzofurans and Tetrahydrofurans
Source: Chemistry. 2014 Jul 2;20(31):9762–9. doi: 10.1002/chem.201402684 (PMC4517160; doi:10.1002/chem.201402684)

CHEMISTRY
A European Journal

Supporting Information

© Copyright Wiley-VCH Verlag GmbH & Co. KGaA, 69451 Weinheim, 2014

Stereodivergent Organocatalytic Intramolecular Michael Addition/ Lactonization for the Asymmetric Synthesis of Substituted Dihydrobenzofurans and Tetrahydrofurans

Dorine Belmessieri, Alix de la Houpliere, Ewen D. D. Calder, James E. Taylor, and Andrew D. Smith*[a]

chem_201402684_sm_miscellaneous_information.pdf

Stereodivergent Organocatalytic Intramolecular Michael Addition-Lactonisation for the Asymmetric Synthesis of Substituted Dihydrobenzofurans and Tetrahydrofurans

*Dorine Belmessieri, Alix de la Houpliere, Ewen D. D. Calder, James E. Taylor, Andrew D. Smith**

EaStCHEM, School of Chemistry, University of St Andrews, North Haugh, St Andrews, KY16 9ST, U.K.

ads10@st-andrews.ac.uk

General Experimental ········ S2

General Procedures ········ S4

Synthesis of Catalysts and Starting Materials ········ S8

*Data for Cinchona Alkaloid Derivatives (**14–19**)* ········ S8

Data for Alkene-Acids ········ S12

Data for Enone-Acids ········ S13

Synthesis of Dihydrobenzofuran and Tetrahydrofuran Derivatives ········ S20

*Data for syn-2,3-Dihydrobenzofurans (**10**, **20–25**)* ········ S20

*Data for anti-2,3-Dihydrobenzofurans (**11**, **26–31**)* ········ S25

*Data for syn-2,3-Tetrahedrofurans (**32–39**)* ········ S32

*Data for syn-3,4-Tetrahydrofurans (**43–48**)* ········ S38

*Data for anti-3,4-Tetrahydrofurans (**49–54**)* ········ S42

Conformation of Stereochemistry ········ S48

References ········ S50

^{1}H and ^{13}C{^{1}H} NMR Spectra ········ S51

HPLC Traces ········ S149

General Experimental

Reactions were performed in flame-dried glassware under an Ar or N_2 atmosphere unless otherwise stated. Anhydrous CH_2Cl_2, Et_2O, THF and toluene were obtained from an MBraun SPS-800 system. Petrol is defined as petroleum ether 40–60 °C. All other solvents and commercial reagents were used as received without further purification unless otherwise stated. Room temperature (rt) refers to 20−25 °C. Temperatures of 0 °C and −78 °C were obtained using ice/water and CO_2(s)/acetone baths respectively.

Analytical thin layer chromatography was performed on pre-coated aluminium plates (Kieselgel 60 F_{254} silica). Plates were visualised under UV light (254 nm) or by staining with either phosphomolybdic acid or $KMnO_4$ followed by heating. Flash column chromatography was performed on Kieselgel 60 silica in the solvent system stated.

Melting points were recorded on an Electrothermal 9100 melting point apparatus.

Optical rotations were measured on a Perkin Elmer Precisely/Model-341 polarimeter operating at the sodium D line with a 100 mm path cell at 20 °C.

HPLC analyses were obtained on two separate machines; either a Gilson HPLC consisting of a Gilson 305 pump, Gilson 306 pump, Gilson 811C dynamic mixer, Gilson 805 manometric module, Gilson 401C dilutor, Gilson 213XL sample injector and sample detection was performed with a Gilson 118 UV/vis detector or a Shimadzu HPLC consisting of a DGU-20A5 degasser, LC-20AT liquid chromatography SIL-20AHT autosampler, CMB-20A communications bus module, SPD-M20A diode array detector and a CTO-20A column oven that allows the temperature to be set from 25–40 °C. Separation was achieved using Chiralcel OD-H and OJ-H columns or Chiralpak AD-H, AS-H, IA, IB, IC, and ID columns.

Infrared spectra (ν_{max}) were recorded on a Shimadzu IRAffinity-1 Fourier transform IR spectrophotometer using either thin film or solid using Pike MIRacle ATR accessory. Analysis was carried out using Shimadzu IRsolution v1.50 and only characteristic peaks are reported.

^{1}H, ^{13}C{^{1}H}, and ^{19}F{^{1}H} NMR spectra were acquired on either a Bruker Avance 300 {δ_H (300 MHz), δ_C (75 MHz), δ_F (282 MHz)}, a Bruker Avance II 400 {δ_H (400 MHz), δ_C (100 MHz), δ_F (376 MHz)} or a Bruker Ultrashield 500 {δ_H (500 MHz), δ_C (125 MHz), δ_F (471 MHz)} spectrometer at ambient temperature in the deuterated solvent stated. Chemical shifts, δ, are quoted in parts per million (ppm) and are referenced to the residual solvent peak. Coupling constants, *J*, are quoted in Hertz (Hz) to the nearest 0.1 Hz. The following abbreviations are used: s, singlet; d, doublet; t, triplet; q, quartet; dd,

doublet of doublets; ddd, doublet of doublet of doublets; td, triplet of doublets; tt, triplet of triplets; m, multiplet; app., apparent and br., broad.

Mass spectrometry (*m/z*) data were acquired by electrospray ionisation (ES), electron impact (EI), chemical ionisation (CI), atmospheric pressure chemical ionisation (APCI) or nanospray ionisation (NSI) at the EPSRC UK National Mass Spectrometry Service Centre at Swansea University.

General Procedures

General Procedure 1: *Trimethylsilyl Protection of Cinchona Alkaloids*

Trimethylsilyl chloride (1.2 eq) was added to a solution of the required cinchona alkaloid (1.0 eq) in the anhydrous solvent stated (0.07 M) and was stirred overnight at rt. The reaction was quenched with $NaHCO_3$ and extracted with CH_2Cl_2 (× 3). The combined organic phases were washed with brine, dried over $MgSO_4$, filtered and concentrated *in vacuo*. The crude product was purified by chromatography column.

General Procedure 2: *Preparation of Enone-Acids from Salicylaldehyde Derivatives*

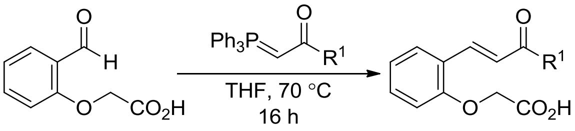

The required phosphorane (1.1 eq) was added to a solution of 2-(formylphenoxy)acetic acid (1.0 eq) in anhydrous THF (0.2 M) under an atmosphere of Ar. The solution was heated at reflux overnight (*cf* 70 °C) before being cooled to rt and basified to pH 12 with 1 M NaOH. The mixture was washed with EtOAc (× 3), the aqueous was then acidified to pH 2 with 1 M. HCl and extracted with EtOAc (× 3). The combined organic extracts were washed with brine (× 2), dried over $MgSO_4$,, filtered and concentrated *in vacuo* to afford the corresponding enone-acid.

General Procedure 3: *Preparation of Enone-Acids from Alkene-Acids*

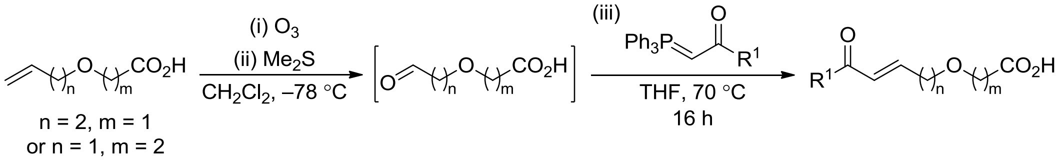

The required alkene-acid (1.0 eq) was dissolved in CH_2Cl_2 (0.01 M) and cooled to –78 °C. A stream of O_3 was bubbled through the solution until a persistent pale blue colour was obtained. The reaction was quenched with dimethyl sulfide (2.2 eq) and allowed to warm to rt. The solution was concentrated *in vacuo* before being dissolved in THF. The required phosphorane was added and the solution was heated at reflux (*cf* 70 °C) for 2 h before being concentrated *in vacuo*. The crude product was basified to pH 12 with 1 M NaOH and washed with EtOAc (× 3). The aqueous layer was acidified to pH 2 with 1 M. HCl and extracted with EtOAc (× 3). The combined organic extracts were washed with brine (× 2), dried over $MgSO_4$,, filtered and concentrated *in vacuo* to afford the corresponding enone-acid.

General Procedure 4: *Synthesis of syn-2,3-Dihydrobenzofurans (**10**, **20-25**)*

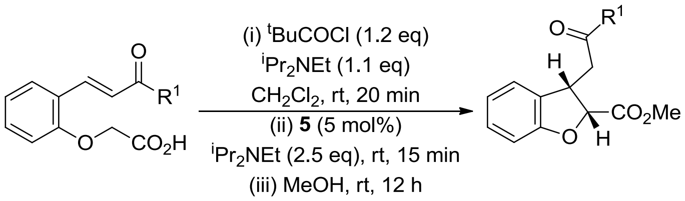

The desired enone-acid (1.0 eq) and ${}^{i}Pr_2NEt$ (1.1 eq) were dissolved in CH_2Cl_2 (to give 0.2 M solution of acid) in a flame-dried round-bottomed flask and cooled to 0 °C before ${}^{t}BuCOCl$ (1.2 eq) was added dropwise. After 20 min, (*S*)-(–)-tetramisole hydrochloride **5** (5 mol%) and ${}^{i}Pr_2NEt$ (2.5 eq) were added and the reaction mixture was warmed to rt and stirred for 15 min. MeOH was added and the reaction stirred for 1 h at rt. The solvent was evaporated and the crude residue was purified directly by column chromatography on silica gel.

Authentic racemic samples were obtained using (*rac*)-(±)-tetramisole hydrochloride **5** (5 mol%) as the catalyst.

General Procedure 5: *Synthesis of anti-2,3-Dihydrobenzofurans (**11**, **26-31**)*

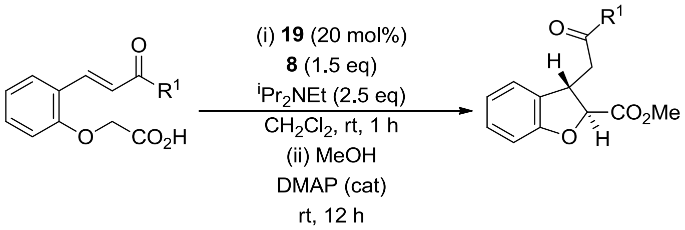

OTMS-Quinidine **19** (20 mol%), ${}^{i}Pr_2NEt$ (2.5 eq), and Mukaiyama derivative **8** (1.5 eq) were dissolved in CH_2Cl_2 (to give 0.3 M solution of **8**) in a flame-dried round-bottomed flask under an N_2 atmosphere. A solution of the desired enone-acid (1.0 eq) in CH_2Cl_2 (0.4 M) was added dropwise and the reaction stirred for 1 h at rt. MeOH and DMAP (cat.) were added and the reaction stirred at rt overnight. The solvent was evaporated and the crude residue was purified directly by column chromatography on silica gel.

Authentic racemic samples were obtained using DABCO (20 mol%) as the catalyst.

General Procedure 6: *Synthesis of syn-2,3-Tetrahydrofurans (**32-39**)*

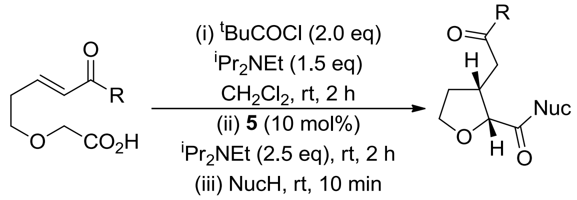

The desired enone-acid (1.0 eq) and ${}^{i}Pr_2NEt$ (1.5 eq) were dissolved in CH_2Cl_2 (to give 0.2 M solution of acid) in a flame-dried round-bottomed flask before ${}^{t}BuCOCl$ (2.0 eq) was added dropwise. After 2 h, (*S*)-(–)-tetramisole hydrochloride **5** (10 mol%) and ${}^{i}Pr_2NEt$ (2.5 eq) were added and the reaction mixture was stirred for 2 h at rt. The reaction was concentrated under reduced pressure before the desired nucleophile was added and stirred for 10 min. The resulting solution was diluted in CH_2Cl_2, washed with 2 M HCl (× 2), saturated $NaHCO_3$ (× 2) and brine, dried over $MgSO_4$, filtered and concentrated under reduced pressure. The crude product was purified by column chromatography on silica gel.

Authentic racemic samples were obtained using (*rac*)-(±)-tetramisole hydrochloride **5** (5 mol%) as the catalyst.

General Procedure 7: *Synthesis of syn-3,4-Tetrahydrofurans (**43-48**)*

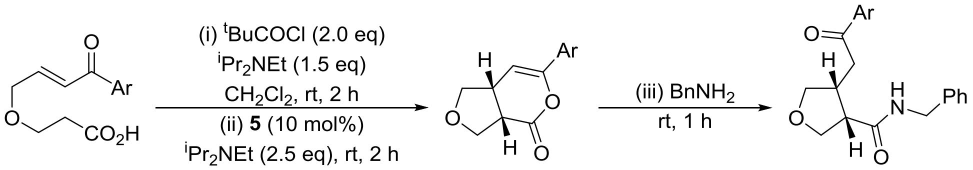

The desired enone-acid (1.0 eq) and ${}^{i}Pr_2NEt$ (1.5 eq) were dissolved in CH_2Cl_2 (to give 0.2 M solution of acid) in a flame-dried round-bottomed flask before ${}^{t}BuCOCl$ (2.0 eq) was added dropwise. After 2 h, (*S*)-(–)-tetramisole hydrochloride **5** (5 mol%) and ${}^{i}Pr_2NEt$ (2.5 eq) were added and the reaction mixture was stirred at rt. After 2 h the reaction was concentrated under reduced pressure and purified directly by chromatography column on silica gel. Alternatively, the desired nucleophile was added and the reaction stirred for 10 min. The resulting solution was diluted in CH_2Cl_2, washed with 2 M HCl (× 2), saturated $NaHCO_3$ (× 2) and brine, dried over MgSO4, filtered and concentrated under reduced pressure. The crude product was purified by column chromatography on silica gel.

Authentic racemic samples were obtained using (*rac*)-(±)-tetramisole hydrochloride **5** (5 mol%) as the catalyst.

General Procedure 8: *Synthesis of anti-3,4-Tetrahydrofurans (**49-54**)*

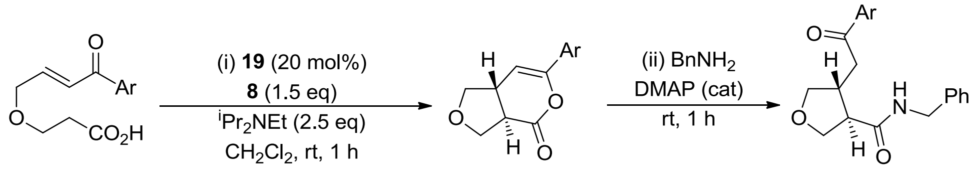

OTMS-Quinidine **19** (20 mol%), $^{i}Pr_2NEt$ (2.5 eq), and Mukaiyama derivative **8** (1.5 eq) were dissolved in CH_2Cl_2 (to give 0.3 M solution of **8**) in a flame-dried round-bottomed flask under an N_2 atmosphere. A solution of the desired enone-acid (1.0 eq) in CH_2Cl_2 (0.4 M) was added dropwise and the reaction stirred at for 1 h at rt. The reaction was concentrated under reduced pressure and purified directly by chromatography column on silica gel. Alternatively, benzylamine (5 eq) and DMAP (cat.) were added and the reaction stirred for 1 h at rt. The resulting solution was diluted in CH_2Cl_2, washed with 1 M HCl (× 2), saturated $NaHCO_3$ (× 2) and brine (× 2), dried over $MgSO_4$, filtered and concentrated under reduced pressure. The crude product was purified by column chromatography on silica gel.

Authentic racemic samples were obtained using DABCO (20 mol%) as the catalyst.

Synthesis of Catalysts and Starting Materials

Data for Cinchona Alkaloid Derivatives

OAc-Quinine (14)

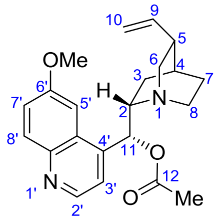

Acetyl chloride (40 µL, 0.46 mmol) was added to a solution of quinine (100.0 mg, 0.30 mmol) in CH_2Cl_2 (10 mL). After stirring at rt for 24 h the reaction was quenched with $NaHCO_3$ (15 mL), extracted with CH_2Cl_2 (× 3), washed with brine, dried over $MgSO_4$, filtered and concentrated *in vacuo* to give the title compound **14** (112 mg, 99%) as a cloudy oil. $[\alpha]_D^{20}$ −41.7 (*c* 0.6, $CHCl_3$); ν_{max} (film) 1731 (C=O), 1619 (C=C), 1508 (C=C); ^{1}H NMR (300 MHz, $CDCl_3$) δ_H: 1.48–1.59 (2H, m, C(3)H^AH^B and C(7)H^AH^B), 1.68–1.75 (1H, m, C(4)*H*), 1.82–1.90 (2H, m, C(3)H^AH^B and C(7)H^AH^B), 2.11 (3H, s, C(12)CH_3), 2.25–2.32 (1H, m, C(5)*H*), 2.56–2.70 (2H, m, C(6)H^AH^B and C(8)H^AH^B), 3.00–3.15 (2H, m, C(2)*H* and C(6)H^AH^B), 3.32–3.40 (1H, m, C(8)H^AH^B), 3.95 (3H, s, OCH_3), 4.97–5.02 (2H, m, C(10)H_2), 5.82 (1H, ddd, *J* 17.2, 10.2, 7.2, C(9)*H*), 6.49 (1H, d, *J* 7.2, C(11)*H*), 7.33–7.38 (3H, m, C(3')*H*, C(5')*H* and C(7')*H*), 8.00 (1H, d, *J* 9.2, C(8')*H*), 8.72 (1H, d, *J* 4.5, C(2')*H*); ^{13}C{^{1}H} NMR (75 MHz, $CDCl_3$) δ_C: 21.2 (C(12)CH_3), 24.3 (*C*(3)), 27.6 (*C*(4)), 27.8 (*C*(7)), 39.7 (*C*(5)), 42.5 (*C*(8)), 55.8 (O*C*H_3), 56.6 (*C*(6)), 59.1 (*C*(2)), 73.8 (*C*(11)), 101.5 (*C*(5')), 114.6 (*C*(10)), 118.9 (*C*(3')), 121.9 (*C*(7')), 127.1 (*C*(4a')), 131.9 (*C*(8')), 141.7 (*C*(9)), 143.6 (*C*(8a')), 144.9 (*C*(4')), 147.5 (*C*(2')), 158.0 (*C*(6')), 170.1 (*C*(12)); *m/z* (ES$^+$) 367 ([M+H]$^+$, 100 %); HRMS (ES$^+$) $C_{22}H_{27}N_2O_3$ [M+H]$^+$ found 367.2019, requires 367.2016 (+0.8 ppm).

OBn-Quinine (15)

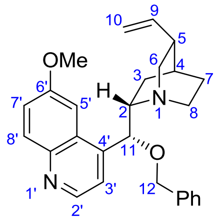

Quinine (100 mg, 0.31 mmol) was added to a suspension of NaH (19 mg, 0.77 mmol) in anhydrous DMF (10 mL) under an atmosphere of Ar. After stirring at rt for 2 h, benzyl bromide (50 µL, 0.40 mmol) was added dropwise and the solution was stirred overnight at rt. The reaction was quenched with brine and extracted with EtOAc (× 3). The combined organic phases were washed with brine,

dried over $MgSO_4$, filtered and concentrated *in vacuo*. The crude product was purified by chromatography column (Petrol : EtOAc : MeOH, 50:50:5) to give the title compound **15** (35 mg, 29 %) as a yellow oil; ^{1}H NMR (300 MHz, $CDCl_3$) δ_H: 1.46–1.69 (2H, m, C(3)H^AH^B and C(7)H^AH^B), 1.71–1.92 (3H, m, C(3)H^AH^B, C(7)H^AH^B and C(4)*H*); 2.24–2.37 (1H, br. m, C(5)*H*), 2.59–2.79 (2H, br. m, C(6)H^AH^B and C(8)H^AH^B), 3.05–3.24 (2H, br. m, C(2)*H* and C(6)H^AH^B), 3.38–3.56 (1H, br. m, C(8)H^AH^B), 3.93 (3H, s, OCH_3), 4.45 (2H, d, *J* 3.2, C(12)H_2), 4.88–5.00 (2H, m, C(10)H_2), 5.28–5.39 (1H, br. m, C(11)*H*), 5.72 (1H, ddd, *J* 17.1, 10.3, 7.2, C(9)*H*), 7.27–7.42 (7H, m, C(5')*H*, C(7')*H* and Ph*H* (×5)), 7.49 (1H, d, *J* 4.4, C(3')*H*), 8.05 (1H, d, *J* 9.7, C(8')*H*), 8.76 (1H, d, *J* 4.4 C(2')*H*); ^{13}C{^{1}H} NMR (75 MHz, $CDCl_3$) δ_C: 22.4 (*C*(3)), 27.6 (*C*(7)) 28.0 (*C*(4)), 39.9 (*C*(5)), 43.3 (*C*(8)), 56.0 (O*C*H_3), 57.0 (*C*(6)), 60.3 (*C*(2)), 71.3 (*C*(12)), 80.4 (*C*(11)), 101.3 (*C*(5')), 114.7 (*C*(10)), 119.1 (*C*(3')), 122.0 (Ar*C*), 127.8 (Ar*C*), 127.9 (Ar*C*), 128.6 (Ar*C*), 132.0 (*C*(8')), 137.9 (Ar*C*). 141.7 (*C*(9)), 144.4 (Ar*C*), 144.9 (Ar*C*), 147.7 (*C*(2')), 158.0 (*C*(6')).

OTMS-Quinine (16)

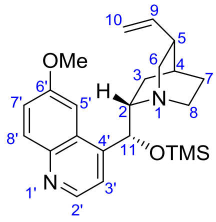

Trimethylsilyl chloride (40 μL, 0.37 mmol) and quinine (100 mg, 0.31 mmol) in anhydrous CH_2Cl_2 (5 mL) were reacted according to general procedure 1. The crude product was purified by column chromatography (EtOAc : MeOH, 85:15) to give the title compound **16** (65 mg, 53%) as a yellow oil. $[\alpha]_D^{20}$ −205.7 (*c* 0.6, $CHCl_3$); ν_{max} (film) 1619 (C=C), 1508 (C=C), 1241(Si-Me), 1007 (Si-OR); ^{1}H NMR (300 MHz, $CDCl_3$) δ_H: 0.03 (9H, s, Si(CH_3)$_3$), 1.27–1.38 (1H, br. m, C(3)H^AH^B), 1.49–1.61 (1H, br. m, C(7)H^AH^B), 1.77–1.91 (3H, br. m, C(3)H^AH^B, C(7)H^AH^B and C(4)*H*), 2.28–2.32 (1H, br. m, C(5)*H*), 2.74–2.75 (2H, br. m, C(6)H^AH^B and C(8)H^AH^B), 2.95–3.07 (1H, br. m, C(2)*H*), 3.08–3.21 (1H, br. m, C(6)H^AH^B), 3.52–3.61 (1H, br. m, C(8)H^AH^B), 3.96 (3H, s, OCH_3), 4.85–4.93 (2H, m, C(10)H_2), 5.57–5.63 (1H, br. m, C(9)*H*), 5.92–5.93 (1H, br. m, C(11)*H*), 7.26–7.44 (3H, m, C(3')*H*, C(5')*H* and C(7')*H*), 7.97–7.99 (1H, m, C(8')*H*), 8.67–8.68 (1H, m, C(2)*H*); ^{13}C{^{1}H} NMR (100 MHz, $CDCl_3$) δ_C: 0.3 (Si(*C*H_3)$_3$), 20.0 (*C*(3)), 27.7 (*C*(7)), 29.7 (*C*(4)), 39.3 (*C*(5)), 43.2 (*C*(8)), 56.4 (O*C*H_3), 56.7 (*C*(6)), 60.8 (*C*(2)), 71.6 (*C*(11)), 100.7 (*C*(5')), 115.1 (*C*(10)), 118.8 (*C*(3')), 122.1 (*C*(7')), 126.2 (*C*(4a')), 131.8 (*C*(8')), 140.7 (*C*(9)), 144.4 (*C*(8a')), 146.8 (*C*(4')), 147.2 (*C*(2')), 158.3 (*C*(6')); *m/z* (ES$^+$) 397 ([M+H]$^+$, 100 %); HRMS (ES$^+$) $C_{23}H_{33}N_2O_2Si$ [M+H]$^+$ found 397.2308, requires 397.2306, (+0.6 ppm).

OTMS-Cinchonidine (17)

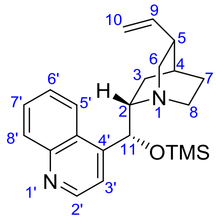

Trimethylsilyl chloride (100 μL, 1.02 mmol) and cinchonidine (250 mg, 0.85 mmol) in anhydrous DMF (10 mL) were reacted according to general procedure 1. The crude product was purified by column chromatography (CH_2Cl_2: MeOH, 95:5) to give the title compound **17** (102 mg, 33%) as a yellow oil with data in accordance with the literature[1]. $[\alpha]_D^{20}$ −114.6 (*c* 0.8, $CHCl_3$), {Lit.[1] $[\alpha]_D^{30}$ −110.6 (*c* 0.05, $CHCl_3$)}; ν_{max} (film) 1637 (C=C), 1509 (C=C), 1252 (Si-Me), 1003 (Si-OR); ^{1}H NMR (300 MHz, $CDCl_3$) δ_H: 0.01 (9H, s, (Si(CH_3)$_3$), 1.38–1.56 (2H, br. m, C(3)H^AH^B and C(7)H^AH^B), 1.71–1.81 (3H, br. m, C(3)H^AH^B, C(7)H^AH^B and C(4)*H*), 2.21–2.35 (1H, br. m, C(5)*H*), 2.65–2.77 (2H, br. m, C(6)H^AH^B and C(8)H^AH^B), 2.96–3.19 (2H, br. m, C(2)*H* and C(6)H^AH^B), 3.39–3.59 (1H, br. m, C(8)H^AH^B), 4.80–4.95 (2H, m, C(10)H_2), 5.54–5.69 (1H, m, C(9)*H*), 5.70–6.00 (1H, br. m, C(11)*H*), 7.42–7.49 (1H, m, C(3')*H*), 7.52–7.60 (1H, m, C(5')*H*), 7.63–7.71 (1H, m, C(7')*H*), 8.06–8.13 (1H, m, C(8')*H*), 8.15–8.23 (1H, m, C(6')*H*), 8.81–8.87 (1H, m, C(2')*H*).

OTMS-Cinchonine (18)

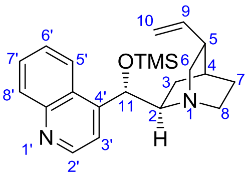

Trimethylsilyl chloride (100 μL, 1.02 mmol) and cinchonine (250 mg, 0.85 mmol) in anhydrous DMF (10 mL) were reacted according to general procedure 1. The crude product was purified by column chromatography (CH_2Cl_2: MeOH, 95:5) to give the title compound **18** (229 mg, 74%) as a yellow oil with data in accordance with the literature.[1] $[\alpha]_D^{20}$ +360.4 (*c* 0.5, $CHCl_3$), {Lit.[1] $[\alpha]_D^{30}$ +149.9 (*c* 0.04, $CHCl_3$)}; ν_{max} (film) 1637 (C=C), 1508 (C=C), 1254 (Si-Me), 1021 (Si-OR); ^{1}H NMR (400 MHz, $CDCl_3$) δ_H: 0.06 (9H, s, (Si(CH_3)$_3$), 1.19–1.25 (1H, br. m, C(3)H^AH^B , 1.41–1.58 (2H, br. m, C(7)H^AH^B and C(4)*H*), 1.74–1.79 (1H, br. m, C(3)H^AH^B), 2.01–2.06 (1H, br. m, C(7)H^AH^B), 2.19–2.27 (1H, m, C(5)*H*), 2.69–2.98 (4H, m, C(2)*H*, C(6)H_2 and C(8)*H*), 3.26–3.39 (1H, m, C(8)H^AH^B), 5.02–5.10 (2H, m, C(10)H_2), 5.60–5.85 (1H, br. m, C(11)*H*), 5.95–6.12 (1H, m, C(9)*H*), 7.46–7.73 (3H, m, C(3')*H*, C(5')*H* and C(7')*H*), 8.09–8.15 (2H, m, C(6')*H* and C(8')*H*), 8.87–8.91 (1H, m, C(2)*H*).

OTMS-Quinidine (19)

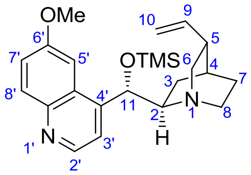

Trimethylsilyl chloride (370 μL, 3.67 mmol) and quinidine (1.00 g, 3.08 mmol) in anhydrous CH_2Cl_2 (40 mL) were reacted according to general procedure 1. The crude product was purified by column chromatography (CH_2Cl_2: MeOH, 95:5) to give the title compound **19** (918 mg, 75%) as a viscous clear oil with data in accordance with the literature.[2] $[\alpha]_D^{20}$ +181.9 (*c* 1.0, $CHCl_3$); ν_{max} (film) 1618 (C=C), 1505 (C=C), 1240 (Si-Me), 1007 (Si-OR); ^{1}H NMR (400 MHz, $CDCl_3$) δ_H: 0.12 (9H, s, (Si(C*H*$_3$)$_3$), 1.04–1.14 (1H, br. m, C(3)H^AH^B), 1.57–1.67 (1H, br. m, C(7)H^AH^B), 1.77–1.83 (1H, br. m, C(7)H^AH^B), 1.92–1.98 (1H, br. m, C(4)*H*), 2.36–2.46 (1H, br. m, C(3)H^AH^B), 2.48–2.57 (1H, br. m, C(5)*H*), 3.01–3.06 (1H, br. m, C(6)H^AH^B), 3.17–3.21 (1H, br. m, C(2)*H*), 3.23–3.34 (2H, br. m, C(6)H^AH^B and C(8)H^AH^B), 3.84–3.89 (1H, br. m, C(8)H^AH^B), 4.07 (3H, s, OC*H*$_3$), 5.13–5.24 (2H, m, C(10)*H*$_2$), 5.98 (1H, ddd, *J* 17.3, 10.2, 7.2, C(9)*H*), 6.68–6.81 (1H, br. m, C(11)*H*), 7.34 (1H, dd, *J* 9.2, 2.6, C(7')*H*), 7.43–7.48 (1H, m, C(3')*H*), 7.55–7.65 (1H, m, C(5')*H*), 7.94–8.00 (1H, d, *J* 9.2, C(8')*H*), 8.69 (1H, d, *J* 4.5, C(2')*H*); ^{13}C{^{1}H} NMR (100 MHz, $CDCl_3$) δ_C: 0.4 (Si(*C*H$_3$)$_3$), 18.2 (*C*(3)), 23.9 (*C*(7)), 27.7 (*C*(4)), 37.8 (*C*(5)), 48.0 (*C*(8)), 49.3 (*C*(6)), 57.5 (O*C*H$_3$), 60.3 (*C*(2)), 68.9 (*C*(11)), 100.7 (*C*(5')), 117.4 (*C*(10)), 118.7 (*C*(3')), 123.0 (*C*(7')), 125.8 (*C*(4a')), 131.8 (*C*(8')), 137.0 (*C*(9)), 144.5 (*C*(8a')), 146.8 (*C*(4')), 147.0 (*C*(2')), 159.1 (*C*(6')); *m/z* (ES$^+$) 397 ([M+H]$^+$, 100 %); HRMS (ES$^+$) $C_{23}H_{33}N_2O_2Si$ [M+H]$^+$ found 397.2308, requires 397.2306, (+0.6 ppm).

Data for Alkene-Acids

2-(But-3-en-1-yloxy)acetic acid (S1)

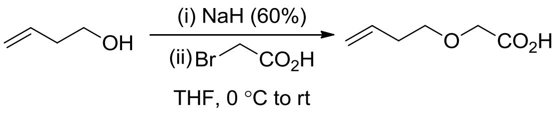

Sodium hydride (60% dispersion mineral oil, 6.70 g, 167 mmol) was suspended in anhydrous THF (400 mL) and cooled to 0 °C. A solution of 3-buten-1-ol (4.8 mL, 55.8 mmol) in anhydrous THF (20 mL) was added dropwise. After 15 min a solution of bromoacetic acid (3.88 g, 27.8 mmol) in THF (20 mL) was added and the reaction stirred overnight, allowing to warm to rt.

The reaction mixture was quenched by addition of Et_2O (400 mL) and MilliQ H_2O (400 mL). The layers were separated, the aqueous was washed with Et_2O (× 3) before being acidified with 8 M HCl and extracted with EtOAc (× 3). The combined organic extracts were dried over $MgSO_4$, filtered and concentrated *in vacuo* to afford the title compound **S1** (3.62 g, 99%) as a yellow oil with data in accordance with the literature.[3] ^{1}H NMR (500 MHz, $CDCl_3$) δ_H: 2.41 (2H, q, *J* 6.7, C(5)H_2), 3.62 (2H, t, *J* 6.7, C(4)H_2), 4.14 (2H, s, C(2)H_2), 5.10 (1H, d, *J* 10.2, C(7)$H^{cis}H^{trans}$), 5.12 (1H, dd, *J* 17.0, 1.5, C(7)$H^{cis}H^{trans}$), 5.81 (1H, ddt, *J* 17.0, 10.0, 6.8, C(6)*H*), 9.82 (1H, br. s, O*H*).

3-(Allyloxy)propanoic acid (S2)

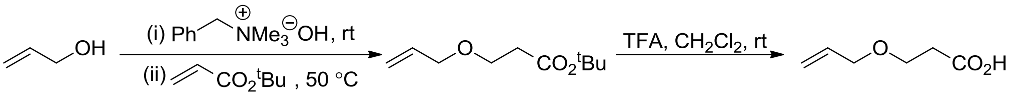

According to a literature procedure,[4] Triton B™ (40% in MeOH, 2.6 mL, 14.7 mmol) was placed in a flame-dried round-bottomed flask and the MeOH was removed *in vacuo* before allyl alcohol (5.0 mL, 73.5 mmol) was added. After stirring at rt for 15min, *tert*-butyl acrylate (9.6 mL, 66.1 mmol) was added and the reaction heated at 50 °C for 72 h. The mixture was cooled to rt and filtered through a short pad of silica and Celite®, washing with CH_2Cl_2. The resulting solution was concentrated *in vacuo* to give *tert*-butyl 3-(allyloxy)propanoate (10.2g, 75%) as a colourless oil with data in accordance with the literature.[4] ^{1}H NMR (300 MHz, $CDCl_3$) δ_H: 1.44 (9H, br. s, $CO_2(CH_3)_3$), 2.49 (2H, t, *J* 6.4, $CH_2CO_2(CH_3)_3$), 3.66 (2H, t, *J* 6.5, $OCH_2CH_2CO_2(CH_3)_3$), 3.98 (2H, d, *J* 5.6, $CHCH_2O$), 5.16 (1H, dd, *J* 10.4, 1.2, CH_2CHCH_2O), 5.26 (1H, dd, *J* 17.0, 1.2, CH_2CHCH_2O), 5.82–5.95 (1H, m, CH_2CHCH_2O).

TFA (1.32 mL, 17.0 mmol) was added to a solution of *tert*-butyl 3-(allyloxy)propanoate (508 mg, 2.73 mmol) in CH_2Cl_2 (6 mL) and stirred at rt for 5 h. The reaction mixture was concentrated *in vacuo* to give the title compound **S2** (236 mg, quant.) as a colourless oil. ^{1}H NMR (300 MHz, $CDCl_3$) δ_H:

2.64 (2H, t, *J* 6.3, CH_2CO_2H), 3.72 (2H, t, *J* 6.2, $OCH_2CH_2CO_2H$), 4.01 (2H, dt, *J* 5.7, 1.4, $CHCH_2O$), 5.19 (1H, dq, *J* 10.4, 1.5, CH_2CHCH_2O), 5.28 (1H, dq, *J* 17.2, 1.6, CH_2CHCH_2O), 5.90 (1H, ddt, *J* 17.2, 10.4, 5.6, CH_2CHCH_2O).

Data for Enone-Acids

(*E*)-2-(2-(3-Oxo-3-phenylprop-1-en-1-yl)phenoxy)acetic acid (S3)

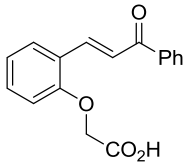

2-(Formylphenoxy)acetic acid (1.06 g, 5.86 mmol) and 1-phenyl-2-(triphenylphosphoranylidene)ethanone (2.67 g, 7.06 mmol) in $CHCl_3$ (30 mL) were reacted according to general procedure 2 to give the title compound **S3** (1.34 g, 81%) as a yellow solid with data in accordance with the literature.[5] mp 116–118 °C {Lit.[5] 126–127 °C }; ^{1}H NMR (300 MHz, $CDCl_3$) δ_H: 4.85 (2H, s, CH_2), 6.90 (1H, d, *J* 8.1, ArC(6)*H*), 7.11 (1H, d, *J* 7.5, ArC(4)*H*), 7.37–7.43 (1H, m, ArC(5)*H*), 7.47–7.60 (3H, m, C*H*COPh and Ph*H* (×2)), 7.68 (1H, dd, *J* 7.5, 1.5, ArC(3)*H*), 7.98 (1H, d, *J* 15.6, C*H*CHCOPh), 8.09–8.14 (3H, m, Ph*H* (×3)).

(*E*)-2-(2-(3-(4-Methoxyphenyl)-3-oxoprop-1-en-1-yl)phenoxy)acetic acid (S4)

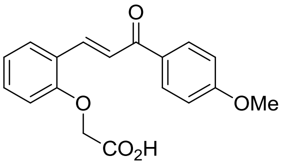

2-(Formylphenoxy)acetic acid (2.60 g, 14.2 mmol) and 1-(4-methoxyphenyl)-2-(triphenylphosphoranylidene)ethanone (7.00 g, 17.1 mmol) in anhydrous THF (80 mL) were reacted according to general procedure 2 to give the title compound **S4** (4.30 g, 98%) as a white solid. mp 140–142 °C; ν_{max} (KBr) 3465 (O-H), 2845 (C-H), 1743 (C=O), 1651 (C=O), 1591 (C=C); ^{1}H NMR (300 MHz, DMSO-d_6) δ_H: 3.86 (3H, s, OCH_3), 4.86 (2H, s, CH_2), 7.03–7.10 (4H, m, Ar*H* (×4)), 7.38–7.43 (1H, td, *J* 9.0, 1.8, Ar*H*), 7.87–7.92 (1H, m, Ar*H*), 7.99 (1H, d, *J* 15.8, C*H*COAr), 8.14–8.21 (3H, m, C*H*CHCOAr and Ar*H* (×2)); ^{13}C{^{1}H} NMR (75 MHz, DMSO-d_6) δ_C: 55.6 (OCH_3), 65.0 (CH_2), 112.6 (Ar*C*H), 114.0 (Ar*C*H (×2)), 121.2 (Ar*C*H), 122.7 (*C*HCOAr), 123.3 (Ar*C*(1)), 130.2 (Ar*C*H), 130.6 (Ar*C*), 130.9 (Ar*C*H (×2)), 131.8 (Ar*C*H), 138.5 (Ar*C*H), 156.8 (Ar*C*(2)), 163.2 (Ar*C*(4')), 170.1 (CO_2H), 187.6 (*C*OAr); *m/z* (ES$^+$) 625 ([2M+H]$^+$, 50%), 313 ([M+H]$^+$, 100%), HRMS (ES$^+$) $C_{18}H_{17}O_5$ [M+H]$^+$ found 313.1069, requires 313.1071 (−0.5 ppm).

(*E*)-2-(2-(3-Oxo-3-(*p*-tolyl)prop-1-en-1-yl)phenoxy)acetic acid (S5)

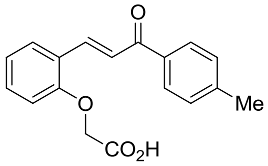

2-(Formylphenoxy)acetic acid (300 mg, 1.77 mmol) and 1-(*p*-tolyl)-2-(triphenylphosphoranylidene)ethanone (720 mg, 1.80 mmol) in $CHCl_3$ (20 mL) were reacted according to general procedure 2 to give the title compound **S5** (380 mg, 77%) as a white solid with data in accordance with the literature.[6] mp 132–134 °C {Lit.[6] 132–134 °C}; ^{1}H NMR (400 MHz, MeOD) δ_H: 2.44 (3H, s, C*H*$_3$), 4.82 (2H, s, C*H*$_2$), 7.01 (1H, d, *J* 8.4, ArC(6)*H*), 7.07 (1H, td, *J* 7.5, 0.8, ArC(4)*H*), 7.36 (2H, d, *J* 7.7, Ar(C3',5')*H*), 7.39–7.43 (1H, m, ArC(5)*H*), 7.72 (1H, dd, *J* 7.5, 1.6, ArC(3)*H*), 8.03–8.07 (3H, m, ArC(2',6')*H* and C*H*COAr), 8.12 (1H, d, *J* 15.8, C*H*CHCOAr).

(*E*)-2-(2-(3-Oxo-3-(4-(trifluoromethyl)phenyl)prop-1-en-1-yl)phenoxy)acetic acid (S6)

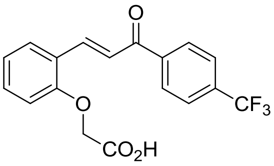

2-(Formylphenoxy)acetic acid (2.40 g, 13.5 mmol) and 1-(4-(trifluoromethyl)phenyl)-2-(triphenylphosphoranylidene)ethanone (7.20 g, 16.1 mmol) in anhydrous THF (80 mL) were reacted according to general procedure 2 to give the title compound **S6** (3.00 g, 64%) as an orange solid. mp 125–130 °C; ν_{max} (KBr) 2923 (O-H br.), 1739 (C=O), 1687 (C=O), 1323; ^{1}H NMR (300 MHz, DMSO-*d*$_6$) δ_H: 4.87 (2H, s, C*H*$_2$), 7.05–7.10 (2H, m, Ar*H* (×2)), 7.42–7.48 (1H, m, Ar*H*), 7.93 (3H, d, *J* 8.1, Ar*H* and Ar(C2',6')*H*), 8.06 (1H, d, *J* 16.0, C*H*COAr), 8.16 (1H, d, *J* 16.0, C*H*CHCOAr), 8.33 (2H, d, *J* 8.1, ArC(3',5')*H*); ^{13}C{^{1}H} NMR (100 MHz, DMSO-*d*$_6$) δ_C: 64.9 (*C*H$_2$), 112.7 (Ar*C*H), 121.2 (Ar*C*H), 122.3 (*C*HCOAr), 122.9 (Ar*C*), 124.1 (q, $^1J_{CF}$ 270, *C*F$_3$,), 125.7 (Ar*C*H (×2)), 129.2 (Ar*C*H (×2)), 130.3 (Ar*C*H), 132.4 (Ar*C*(4)CF$_3$), 132.5 (Ar*C*H), 140.3 (Ar*C*), 140.9 (Ar*C*H), 157.0 (Ar*C*), 169.9 (*C*O$_2$H), 188.7 (*C*OAr); *m/z* (ES$^-$) 349 ([M–H]$^-$, 100 %), HRMS (ES$^-$) $C_{18}H_{12}F_3O_4$ [M–H]$^-$ found 349.0680, requires 349.0688 (–2.2 ppm).

(*E*)-2-(2-(3-(4-Chlorophenyl)-3-oxoprop-1-en-1-yl)phenoxy)acetic acid (S7)

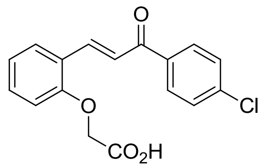

2-(Formylphenoxy)acetic acid (500 mg, 2.77 mmol) and 1-(4-chlorophenyl)-2-(triphenylphosphoranylidene)ethanone (1.30 g, 3.05 mmol) in $CHCl_3$ (20 mL) were reacted according to general procedure 2 to give the title compound **S7** (580 mg, 67%) as a yellow solid with data in accordance with the literature.[6] mp 167–168 °C {Lit.[6] 167–168 °C}; ^{1}H NMR (500 MHz, DMSO-*d*$_6$) δ_H: 4.85 (2H, s, C*H*$_2$), 7.04–7.07 (2H, m, ArC(4)*H* and ArC(6)*H*), 7.41–7.44 (1H, m, ArC(5)*H*), 7.63 (2H, d, *J* 8.5, ArC(3',5')*H*), 7.93 (1H, dd, *J* 7.6, 1.5, ArC(3)*H*), 8.04 (1H, d, *J* 15.7, C*H*COAr), 8.13 (1H, d, *J* 15.7, C*H*CHCOAr), 8.19 (2H, d, *J* 8.5, ArC(2',6')*H*).

(*E*)-2-(2-(3-(Naphthalen-2-yl)-3-oxoprop-1-en-1-yl)phenoxy)acetic acid (S8)

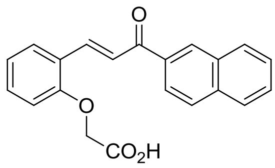

2-(Formylphenoxy)acetic acid (2.40 g, 13.5 mmol) and 1-(naphthalen-2-yl)-2-(triphenylphosphoranylidene)ethanone (7.00 g, 16.3 mmol) in anhydrous THF (80 mL) were reacted according to general procedure 2 to give the title compound **S8** (3.60 g, 80%) as an orange solid. mp 136–140 °C; ν_{max} (KBr) 3600–2500 (O-H), 1754 (C=O), 1637 (C=O), 1566 (C=C); ^{1}H NMR (400 MHz, DMSO-*d*$_6$) δ_H: 4.89 (2H, s, C*H*$_2$), 7.07–7.11 (2H, m, Ar*H* (×2)), 7.42–7.47 (1H, m, Ar*H*), 7.65–7.69 (2H, m, Ar*H* (×2)), 7.95–7.99 (1H, m, Ar*H*), 8.01–8.11 (3H, m, C*H*COAr and Ar*H* (×2)), 8.15–8.20 (2H, m, Ar*H* (×2)), 8.38 (1H, d, *J* 15.7, Ar*H*), 8.99 (1H, s, Ar*H*); ^{13}C{^{1}H} NMR (75 MHz, DMSO-*d*$_6$) δ_C: 65.0 (*C*H$_2$), 112.7 (Ar*C*H), 121.2 (Ar*C*H), 122.7 (C*H*COAr), 123.3 (Ar*C*(1)), 124.1 (Ar*C*H), 126.9 (Ar*C*H), 127.7 (Ar*C*H), 128.4 (Ar*C*H), 128.6 (Ar*C*H), 129.6 (Ar*C*H), 130.3 (Ar*C*H), 130.4 (Ar*C*H), 132.0 (Ar_{Naphth} *C*(2')), 132.4 (Ar*C*H), 135.0 (Ar_{Naphth}*C*(4a') and Ar_{Naphth}*C*(8a')), 139.1 (Ar*C*H), 156.9 (Ar*C*(2)), 170.0 (*C*O$_2$H), 189.0 (*C*OAr); *m/z* (ES$^+$) 333 ([M+H]$^+$, 100 %), HRMS (ES$^+$) $C_{21}H_{17}O_4$ [M+H]$^+$ found 313.1125, requires 313.1121 (+1.7 ppm).

(*E*)-2-(2-(3-Oxobut-1-en-1-yl)phenoxy)acetic acid (S9)

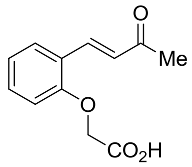

2-(Formylphenoxy)acetic acid (500 mg, 2.77 mmol) and 1-(triphenylphosphoranylidene)propan-2-one (970 mg, 3.05 mmol) in $CHCl_3$ (20 mL) were reacted according to general procedure 2 to give the title compound **S9** (440 mg, 60%) as a yellow solid with data in accordance with the literature.[6] mp 105–109 °C {Lit.[6] 105–109 °C}; ^{1}H NMR (300 MHz, $CDCl_3$) δ_H: 2.40 (3H, s, C*H*$_3$), 4.78 (2H, s, C*H*$_2$), 6.82–6.88 (2H, m, ArC(6)*H* and C*H*COMe), 7.05 (1H, t, *J* 7.5, ArC(4)*H*), 7.36 (1H, td, *J* 7.5, 1.5, ArC(5)*H*), 7.58 (1H, dd, *J* 7.5, 1.5, ArC(3)*H*), 7.95 (1H, d, *J* 16.5, C*H*CHCOMe).

(*E*)-2-((5-Oxo-5-phenylpent-3-en-1-yl)oxy)acetic acid (S10)

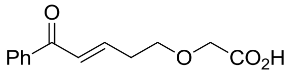

Alkene-acid **S1** (1.80 g, 13.8 mmol) and DMS (1.24 mL, 16.9 mmol) in CH_2Cl_2 (1.4 L) were reacted according to general procedure 3 followed by 1-phenyl-2-(triphenylphosphoranylidene)ethanone (5.26 g, 13.8 mmol) in THF (250 mL) to give the title compound **S10** (1.64 g, 50%) as a yellow oil. ν_{max} (film) 1732 (C=O, acid), 1668 (C=O, ketone), 1616 (C=C), 1130 (C-O, ether); ^{1}H NMR (500 MHz, $CDCl_3$) δ_H: 2.66 (2H, q, *J* 6.3, C*H*$_2$CH$_2$O), 3.76 (2H, t, *J* 6.4, CH$_2$C*H*$_2$O), 4.17 (2H, s, OC*H*$_2$CO$_2$H), 7.00–7.07 (2H, m, C*H*C*H*COPh), 7.46 (2H, td, *J* 6.7, 1.3 PhC(3,5)*H*), 7.55 (1H, td, *J* 7.4, 1.3, PhC(4)*H*), 7.93 (2H, dt, *J* 7.0, 1.3, PhC(2,6)*H*); ^{13}C{^{1}H} NMR (100 MHz, $CDCl_3$) δ_C: 33.0 (*C*H$_2$CH$_2$O), 67.9 (O*C*H$_2$CO$_2$H), 69.9 (*C*H$_2$O), 127.7 (CO*C*HCH), 128.6 (Ph*C*(4)), 128.7 (Ph*C*(3,5)), 132.9 (Ph*C*(2,6)), 137.7 (Ph*C*(1)), 145.4 (COCH*C*H), 174.9 (*C*O$_2$H), 190.9 (*C*OCHCH); *m/z* (ES$^+$) 486 ([2M+H$_2$O]$^+$, 87%), 235 ([M+H]$^+$, 100%); HRMS (ES$^+$) $C_{13}H_{15}O_4$ [M+H]$^+$ found 235.0963, requires 235.0965 (−0.8 ppm).

(*E*)-2-((5-(4-Methoxyphenyl)-5-oxopent-3-en-1-yl)oxy)acetic acid (S11)

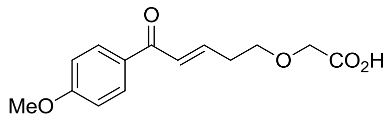

Alkene-acid **S1** (0.80 g, 6.15 mmol) and DMS (1.24 mL, 16.9 mmol) in CH_2Cl_2 (800 mL) were reacted according to general procedure 3 followed by 1-(4-methoxyphenyl)-2-(triphenylphosphoranylidene)ethanone (2.52 g, 6.15 mmol) in THF (150 mL) to give the title compound **S11** (1.15 g, 70%) as a yellow oil. ν_{max} (film) 1734 (C=O, acid), 1665 (C=O, ketone), 1595 (C=C); ^{1}H NMR (300 MHz, $CDCl_3$) δ_H: 2.61–2.67 (2H, m, C*H*$_2$CH$_2$O), 3.75 (2H, t, *J* 6.4, CH$_2$C*H*$_2$O),

3.86 (3H, s, OC*H*$_3$), 4.15 (2H, s, OC*H*$_2$CO$_2$H), 6.91–6.96 (2H, m, COC*H*C*H*), 7.01–7.04 (2H, m, ArC(3,5)*H*), 7.93–7.98 (2H, m, ArC(2,6)*H*); ^{13}C{^{1}H} NMR (75 MHz, CDCl$_3$) δ_C: 33.0 (*C*H$_2$CH$_2$O), 55.6 (O*C*H$_3$), 68.0 (O*C*H$_2$CO$_2$H), 70.1 (CH$_2$*C*H$_2$O), 113.9 (Ar*C*(3,5)), 127.4 (CO*C*HCH), 130.6 (Ar*C*(1)), 131.1 (Ar*C*(2,6)), 144.3 (COCH*C*H), 163.6 (Ar*C*(4)), 174.3 (*C*O$_2$H), 189.1 (*C*OCHCH); *m/z* (ES$^+$) 265 ([M+H]$^+$, 100%); HRMS (ES$^+$) $C_{14}H_{17}O_4$ [M+H]$^+$ found 265.1070, requires 265.1071 (−0.2 ppm).

(*E*)-2-((5-(4-Chlorophenyl)-5-oxopent-3-en-1-yl)oxy)acetic acid (S12)

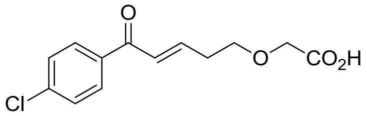

Alkene-acid **S1** (1.00 g, 7.68 mmol) and DMS (1.24 mL, 16.9 mmol) in CH$_2$Cl$_2$ (800 mL) were reacted according to general procedure 3 followed by 1-(4-chlorophenyl)-2-(triphenylphosphoranylidene)ethanone (3.19 g, 7.68 mmol) in THF (150 mL) to give the title compound **S12** (0.81 g, 39%) as a yellow oil. ν_{max} (film) 1730 (C=O, acid), 1668 (C=O, ketone), 1618 (C=C), 1587 (C=C), 1132 (C-O), 1089 (C-O), 827 (C-Cl); ^{1}H NMR (300 MHz, CDCl$_3$) δ_H: 2.66 (2H, q, *J* 5.8, C*H*$_2$CH$_2$O), 3.76 (2H, t, *J* 6.3, CH$_2$C*H*$_2$O), 4.17 (2H, s, OC*H*$_2$CO$_2$H), 6.97–7.10 (2H, m, COC*H*C*H*), 7.41–7.45 (2H, m, Ar(2,6)*H*), 7.86–7.91 (2H, m, Ar(3,5)*H*); ^{13}C{^{1}H} NMR (75 MHz, CDCl$_3$) δ_C: 33.0 (*C*H$_2$CH$_2$O), 67.9 (CH$_2$*C*H$_2$O), 69.9 (O*C*H$_2$CO$_2$H), 127.2 (COCH*C*H), 129.0 (Ar*C*(3,5)), 130.2 (Ar*C*(2,6)), 136.1 (Ar*C*(1)), 139.4 (Ar*C*(4)), 146.0 (CO*C*HCH), 175.1 (*C*O$_2$H), 189.5 (*C*OCHCH); *m/z* (ES$^-$) 535 ([2M−H]$^-$, 100%); HRMS (ES$^-$) $C_{13}H_{12}O_4Cl$ [M−H]$^-$ found 267.0427, requires 267.0430 (−1.0 ppm).

(*E*)-2-((5-Oxohex-3-en-1-yl)oxy)acetic acid (40)

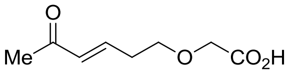

Alkene-acid **S1** (1.02 g, 7.80 mmol) and DMS (1.24 mL, 16.9 mmol) in CH$_2$Cl$_2$ (800 mL) were reacted according to general procedure 3 followed by 1-(triphenylphosphoranylidene)propan-2-one (2.98 g, 9.36 mmol) in THF (150 mL) to give the title compound **40** (0.82 g, 61%) as a yellow oil. ν_{max} (film) 1734 (C=O, acid), 1672 (C=O, ketone), 1625 (C=C); ^{1}H NMR (500 MHz, CDCl$_3$) δ_H: 2.24 (3H, s, COC*H*$_3$), 2.54 (2H, qd, *J* 6.5, 1.4, C*H*$_2$CH$_2$O), 3.68 (2H, t, *J* 6.3, CH$_2$C*H*$_2$O), 4.10 (2H, s, OC*H*$_2$CO$_2$H), 6.15 (1H, dt, *J* 16.1, 1.4, COC*H*CH), 6.84 (1H, dt, *J* 16.1, 6.8, COCHC*H*); ^{13}C{^{1}H} NMR (75 MHz, CDCl$_3$) δ_C: 26.9 (CO*C*H$_3$), 32.7 (*C*H$_2$CH$_2$O), 67.9 (O*C*H$_2$CO$_2$H), 69.8 (CH$_2$*C*H$_2$O), 132.8 (CO*C*HCH), 144.7 (COCH*C*H), 174.1 (*C*O$_2$H), 199.2 (*C*OCHCH); *m/z* (ES$^+$) 195 ([M+Na]$^+$, 100%); HRMS (ES$^+$) $C_8H_{12}O_4Na$ [M+Na]$^+$ found 195.0625, requires 195.0633 (−1.4 ppm).

(*E*)-2-((6,6-Dimethyl-5-oxohept-3-en-1-yl)oxy)acetic acid (S13)

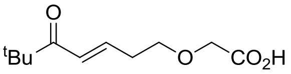

Alkene-acid **S1** (1.00 g, 7.68 mmol) and DMS (1.24 mL, 16.9 mmol) in CH_2Cl_2 (800 mL) were reacted according to general procedure 3 followed by 1-(4-tert-butyl)-2-(triphenylphosphoranylidene)ethanone (2.77 g, 7.68 mmol) in THF (150 mL) to give the title compound **S13** (0.49 g, 30%) as a yellow oil. ν_{max} (film) 1734 (C=O, acid), 1686 (C=O, ketone), 1622 (C=C), 1134 (C-O, ether); ^{1}H NMR (300 MHz, $CDCl_3$) δ_H: 1.15 (9H, s, C(C*H*$_3$)$_3$), 2.55 (2H, qd, *J* 6.6, 1.5, C*H*$_2$CH$_2$O), 3.69 (2H, t, *J* 6.5, CH$_2$C*H*$_2$O), 4.14 (2H, s, OC*H*$_2$CO$_2$H), 6.63 (1H, dt, *J* 15.3, 1.5, COC*H*CH), 6.91 (1H, dt, *J* 15.3, 6.8, COCHC*H*); ^{13}C{^{1}H} NMR (125 MHz, $CDCl_3$) δ_C: 26.2 (C(*C*H$_3$)$_3$), 32.7 (*C*H$_2$CH$_2$O), 43.1 (*C*(CH$_3$)$_3$), 68.0 (O*C*H$_2$CO$_2$H), 70.1 (CH$_2$*C*H$_2$O), 126.2 (CO*C*HCH), 142.9 (COCH*C*H), 174.4 (*C*O$_2$H), 204.4 (*C*OCHCH); *m/z* (ES$^-$) 427 ([2M−H]$^-$, 100%); HRMS (ES$^-$) $C_{11}H_{17}O_4$ [M−H]$^-$ found 213.1131, requires 213.1132 (−0.6 ppm).

(*E*)-3-((4-Oxo-4-phenylbut-2-en-1-yl)oxy)propanoic acid (S14)

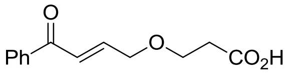

Alkene-acid **S2** (1.00 g, 7.68 mmol) and DMS (1.24 mL, 16.9 mmol) in CH_2Cl_2 (800 mL) were reacted according to general procedure 3 followed by 1-phenyl-2-(triphenylphosphoranylidene)ethanone (2.92 g, 7.68 mmol) in THF (250 mL) to give the title compound **S14** (96:4 *E*:*Z*, 1.03 g, 57%) as a yellow oil. ν_{max} (film) 1714 (C=O, acid), 1670 (C=O, ketone), 1624 (C=C), 1126 (C-O, ether); ^{1}H NMR (400 MHz, $CDCl_3$) δ_H: 2.71 (2H, t, *J* 6.1, OCH$_2$C*H*$_2$CO$_2$H), 3.82 (2H, t, *J* 6.1, OC*H*$_2$CH$_2$CO$_2$H), 4.28 (2H, dd, *J* 3.9, 1.9, OC*H*$_2$CH), 7.02 (1H, dt, *J* 15.5, 3.9, C*H*CHCOPh), 7.20 (1H, dt, *J* 15.5, 1.9, CHC*H*COPh), 7.44–7.48 (2H, m, Ph(3,5)*H*), 7.53–7.57 (1H, m, PhC(4)*H*), 7.95–7.97 (2H, m, PhC(2,6)*H*); ^{13}C{^{1}H} NMR (100 MHz, $CDCl_3$) δ_C: 35.0 (OCH$_2$*C*H$_2$CO$_2$H), 66.1 (O*C*H$_2$CH$_2$CO$_2$H), 70.2 (O*C*H$_2$CH), 124.9 (*C*HCHCOPh), 128.7 (Ph*C*(3,5)), 128.8 (Ph*C*(2,6)), 133.1 (Ph*C*(4)), 137.6 (Ph*C*(1)), 144.2 (CH*C*HCOPh), 177.5 (*C*O$_2$H), 190.5 (*C*OPh); *m/z* (APCI$^+$) 235 ([M+H]$^+$, 100%); HRMS (APCI$^+$) $C_{13}H_{15}O_4$ [M+H]$^+$ found 235.0960, requires 235.0965 (–2.1 ppm).

(*E*)-3-((4-(4-Methoxyphenyl)-4-oxobut-2-en-1-yl)oxy)propanoic acid (S15)

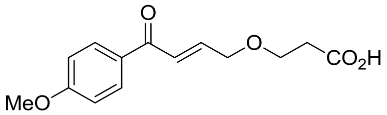

Alkene-acid **S2** (1.00 g, 7.68 mmol) and DMS (1.24 mL, 16.9 mmol) in CH_2Cl_2 (750 mL) were reacted according to general procedure 3 followed by 1-(4-methoxyphenyl)-2-(triphenylphosphoranylidene)ethanone (3.15 g, 7.68 mmol) in THF (150 mL) to give the title compound **S15** (96:4 *E*:*Z*, 1.10 g, 54%) as a pale yellow solid. mp 58–61 °C; ν_{max} (film) 2901, 1699, 1659, 1622, 1591, 1572, 1512, 1364, 1331, 1308, 1256, 1171; ^{1}H NMR (300 MHz, $CDCl_3$) δ_H: 2.72 (2H, t, *J* 6.1, C$H_2$$CO_2$H), 3.83 (2H, t, *J* 6.1, OC$H_2$$CH_2$), 3.87 (3H, s, OC$H_3$), 4.29 (2H, dd, *J* 4.0, 1.9, CHCH_2O), 6.92–7.04 (3H, m, C*H*CH_2 and ArC(3,5)*H*), 7.20 (1H, dt, *J* 15.5, 1.9, COC*H*CH), 7.98 (2H, d, *J* 8.9, ArC(2,6)*H*); ^{13}C{^{1}H} NMR (75 MHz, $CDCl_3$) δ_C: 35.0 (*C*$H_2$$CO_2$H), 55.6 (O*C*$H_3$), 66.1 (O*C*$H_2$$CH_2$), 70.4 (O*C*$H_2$CH), 113.9 (Ar*C*(3,5)H), 114.0 (*C*HCHCOAr), 124.8 (Ar*C*H), 131.2 (Ar*C*H), 143.1 (CH*C*HCOAr), 163.6 (Ar*C*(4)), 176.7 (*C*O_2H), 188.7 (*C*OAr); HRMS (NSI$^+$) $C_{14}H_{17}O_5$ $[M+H]^+$ found 265.1069, requires 265.1071 (–0.6 ppm).

(*E*)-3-((4-(4-Chlorophenyl)-4-oxobut-2-en-1-yl)oxy)propanoic acid (S16)

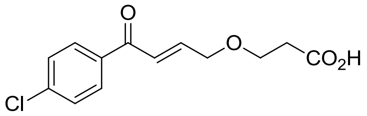

Alkene-acid **S2** (1.00 g, 7.70 mmol) and DMS (1.24 mL, 16.9 mmol) in CH_2Cl_2 (800 mL) were reacted according to general procedure 3 followed by 1-(4-chlorophenyl)-2-(triphenylphosphoranylidene)ethanone (3.19 g, 7.70 mmol) in THF (150 mL) to give the title compound **S16** (96:4 *E*:*Z*, 0.95 g, 46%) as a yellow solid. mp 60 °C; ν_{max} (film) 1710 (C=O, acid), 1674 (C=O, ketone), 1622 (C=C), 1142 (C-O, ether), 1088 (Ar-Cl); ^{1}H NMR (400 MHz, $CDCl_3$) δ_H: 2.71 (2H, t, *J* 6.0, OCH_2C$H_2$$CO_2$H), 3.82 (2H, t, *J* 6.0, OC$H_2$$CH_2CO_2$H), 4.28 (2H, dd, *J* 3.7, 1.9, OCH_2CH), 7.03 (1H, dt, *J* 15.4, 3.7, CHC*H*COAr), 7.17 (1H, dt, *J* 15.4, 2.0, C*H*CHCOAr), 7.41–7.45 (2H, m, ArC(3,5)*H*), 7.88–7.92 (2H, m, ArC(2,6)*H*); ^{13}C{^{1}H} NMR (100 MHz, $CDCl_3$) δ_C: 34.9 (*C*$H_2$$CO_2$H), 66.2 (O*C*$H_2$$CH_2$), 70.2 (O*C*$H_2$CH), 124.3 (*C*HCHCOAr), 129.0 (Ar*C*(3,5)), 130.2 (Ar*C*(2,6)), 136.0 (Ar*C*(1)), 139.5 (Ar*C*(4)), 144.7 (CH*C*HCOAr), 177.1 (*C*O_2H), 189.0 (*C*OAr); *m/z* (NSI$^-$) 267 ($[M–H]^-$, 100%); HRMS (NSI$^-$) $C_{13}H_{12}O_4Cl$ $[M–H]^-$ found 267.0425, requires 267.0430 (–1.7 ppm).

Synthesis of Dihydrobenzofuran and Tetrahydrofuran Derivatives

Data for syn-2,3-Dihydrobenzofurans

(2*R*,3*S*)-Methyl 3-(2-oxo-2-phenylethyl)-2,3-dihydrobenzofuran-2-carboxylate (*ent*-10)

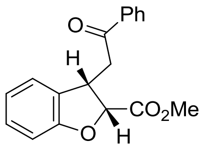

As reported previously,[6] enone-acid **S3** (51 mg, 0.18 mmol), $^{i}Pr_2NEt$ (37 μL, 0.20 mmol), $^{t}BuCOCl$ (26 μL, 0.22 mmol) in CH_2Cl_2 (3 mL) followed by (*S*)-(–)-tetramisole hydrochloride **5** (2.2 mg, 9.0 μmol) and $^{i}Pr_2NEt$ (76 μL, 0.45 mmol) were reacted according to general procedure 4 followed by addition of MeOH. The crude product (>99:1 $dr_{syn:anti}$) was purified by column chromatography (Petrol : EtOAc 80:20) to give the title compound *ent*-**10** (52 mg, 98%) as a white solid. mp 114–116 °C; $[\alpha]_D^{20}$ +21.4 (*c* 1.0 in $CHCl_3$); Chiral HPLC analysis, Chiralpak AD-H (85:15 hexane : IPA, flow rate 1.0 mLmin^{-1}, 254 nm) t_R (2*R*,3*S*): 14.8 min, t_R (2*S*,3*R*): 15.9 min, 94% ee; ν_{max} (KBr) 1740 (C=O), 1212 (C-O); ^{1}H NMR (400 MHz, $CDCl_3$) δ_H: 3.30 (1H, dd, *J* 18.0, 6.8, CH^AH^B), 3.37 (1H, dd, *J* 18.0, 7.6, CH^AH^B), 3.62 (3H, s, OC*H*$_3$), 4.48–4.56 (1H, m , C(3)*H*), 5.39 (1H, d, *J* 9.2, C(2)*H*), 6.87–6.95 (2H, m, Ar*H*), 7.15–7.21 (2H, m, Ar*H*), 7.46 (2H, t, *J* 8.0, PhC(3,5)*H*), 7.58 (1H, tt, *J* 7.4, 1.3, PhC(4)*H*), 7.90–7.95 (2H, m, PhC(2,6)*H*); $^{13}C\{^1H\}$ NMR (100 MHz, $CDCl_3$) δ_C: 40.3 (*C*(3)H), 40.4 (*C*H$_2$), 52.3 (CO_2CH_3), 82.8 (*C*(2)H), 110.2 (Ar*C*(7)), 121.7 (Ar*C*(5)), 124.5 (Ar*C*H), 128.1 (Ph*C*(3,5)), 128.7 (Ar*C*(3a)), 128.9 (Ph*C*(2,6)), 129.1 (Ar*C*H), 133.6 (Ar*C*H), 136.6 (Ph*C*(1)), 158.8 (Ar*C*(7a)), 170.1 (CO_2CH_3), 197.5 (*C*O); *m/z* (ES$^+$) 314 ([M+NH$_4$]$^+$, 67%), 297 ([M+H]$^+$, 100%); HRMS (ES$^+$) $C_{18}H_{17}O_4$ [M+H]$^+$ found 297.1125, requires 297.1126 (–0.5 ppm).

(2*R*,3*S*)-Methyl-3-(2-(4-methoxyphenyl)-2-oxoethyl)-2,3-dihydrobenzofuran-2-carboxylate (20)

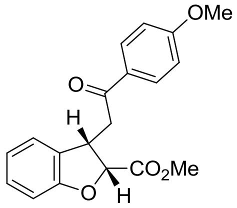

Enone-acid **S4** (2.00 g, 6.40 mmol), $^{i}Pr_2NEt$ (1.22 mL, 7.04 mmol), $^{t}BuCOCl$ (0.95 mL, 7.68 mmol) in CH_2Cl_2 (30 mL) followed by (*S*)-(–)-tetramisole hydrochloride **5** (15.4 mg, 64 μmol) and $^{i}Pr_2NEt$ (2.79 mL, 16.0 mmol) were reacted according to general procedure 4 followed by addition of MeOH. The crude product (>99:1 $dr_{syn:anti}$) was purified by column chromatography (Petrol : EtOAc 80:20) to give the title compound **20** (1.98 g, 95%) as white crystals. mp 98–100 °C; $[\alpha]_D^{20}$ +20.3 (*c* 0.6 in

CH_2Cl_2); Chiral HPLC analysis, Chiralpak AD-H (85:15 hexane : IPA, flow rate 0.5 mLmin^{-1}, 254 nm) t_R (2*R*,3*S*): 36.9 min, t_R (2*S*,3*R*): 42.9 min, 99% ee; ν_{max} (KBr) 1751 (C=O ester), 1671 (C=O ketone); ^{1}H NMR (300 MHz, $CDCl_3$) δ_H: 3.23 (1H, dd, *J* 17.5, 6.5 CH^AH^B), 3.31 (1H, dd, *J* 17.5, 7.5, CH^AH^B), 3.61 (3H, s, CO_2CH_3), 3.87 (3H, s, ArOCH_3), 4.44–4.57 (1H, m, C(3)*H*), 5.38 (1H, d, *J* 9.1, C(2)*H*), 6.83–6.98 (4H, m, Ar*H* (×4)), 7.11–7.22 (2H, m, ArC(3',5')*H*), 7.91 (2H, d, *J* 8.9, ArC(2',6')*H*); ^{13}C{^{1}H} NMR (100 MHz, $CDCl_3$) δ_C: 39.9 (*C*H_2), 40.3 (*C*(3)H), 52.3 (CO_2CH_3), 55.6 (O*C*H_3), 82.8 (*C*(2)H), 110.2 (Ar*C*H), 114.0 (Ar*C*(3,5)H), 121.6 (Ar*C*H), 124.6 (Ar*C*H), 128.8 (Ar*C*(3a)), 129.0 (Ar*C*H), 129.8 (Ar*C*(1)), 130.5 (Ar*C*(2,6)H), 158.8 (Ar*C*(7a)), 163.9 (Ar*C*(4)), 170.1 (*C*O_2CH_3), 195.9 (*C*O); *m/z* (ES^+) 327 ($[M+H]^+$, 100%); HRMS (ES^+) $C_{19}H_{19}O_5$ $[M+H]^+$ found 327.1231, requires 327.1227 (+1.2 ppm).

(2*R*,3*S*)-Methyl-3-(2-oxo-2-(*p*-tolyl)ethyl)-2,3-dihydrobenzofuran-2-carboxylate (21)

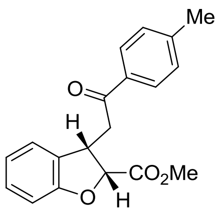

As reported previously,[6] enone-acid **S5** (53 mg, 0.18 mmol), $^iPr_2NEt$ (37 μL, 0.20 mmol), ^tBuCOCl (26 μL, 0.22 mmol) in CH_2Cl_2 (3 mL) followed by (*S*)-(–)-tetramisole hydrochloride **5** (2.2 mg, 9.0 μmol) and $^iPr_2NEt$ (76 μL, 0.45 mmol) were reacted according to general procedure 4 followed by addition of MeOH. The crude product (>99:1 $dr_{syn:anti}$) was purified by column chromatography (Petrol : EtOAc 80:20) to give the title compound **21** (53 mg, 95%) as a white solid, with data in accordance with the literature. mp 90–92 °C; $[\alpha]_D^{20}$ +18.0 (*c* 0.5 in $CHCl_3$); Chiral HPLC analysis, Chiralpak AD-H (85:15 hexane : IPA, flow rate 1.0 mLmin^{-1}, 254 nm) t_R (2*S*,3*R*): 12.9 min, t_R (2*R*,3*S*): 14.9 min, 94% ee; ν_{max} (KBr) 1749 (C=O ester), 1679 (C=O ketone); ^{1}H NMR (300 MHz, $CDCl_3$) δ_H: 2.41 (3H, s, ArC(4')CH_3), 3.26 (1H, dd, *J* 17.8, 6.5, CH^AH^B), 3.34 (1H, dd, *J* 17.8, 7.5, CH^AH^B), 3.61 (3H, s, CO_2CH_3), 4.46–4.56 (1H, m, C(3)*H*), 5.38 (1H, d, *J* 9.5, C(2)*H*), 6.84–6.94 (2H, m, Ar*H* (×2)), 7.12–7.29 (4H, m, Ar*H* (×4)), 7.81 (2H, d, *J* 8.2, ArC(2',6')*H*); ^{13}C{^{1}H} NMR (125 MHz, $CDCl_3$) δ_C: 21.8 (ArC(4)*C*H_3), 31.1 (*C*H_2), 40.3 (*C*(3)H), 52.3 (CO_2CH_3), 82.8 (*C*(2)H), 110.2 (Ar*C*H), 121.6 (Ar*C*H), 124.5 (Ar*C*H), 128.2 (Ar*C*(2',6')H), 128.7 (Ar*C*H), 129.1 (Ar*C*(3a)), 129.5 (Ar*C*(3',5')H), 134.2 (Ar*C*(1')), 144.5 (Ar*C*(4')), 158.8 (Ar*C*(7a)), 170.1 (*C*O_2CH_3), 197.1 (*C*O); *m/z* (ES^+) 333 ($[M+Na]^+$, 60%), 311 ($[M+H]^+$, 100%); HRMS (ES^+) $C_{19}H_{19}O_4$ $[M+H]^+$ found 311.1282, requires 311.1278 (+1.3 ppm).

(2*R*,3*S*)-Methyl3-(2-oxo-2-(4-(trifluoromethyl)phenyl)ethyl)-2,3-dihydrobenzofuran-2-carboxylate (22)

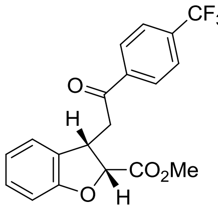

Enone-acid **S6** (62 mg, 0.18 mmol), $^{i}Pr_2NEt$ (35 μL, 0.19 mmol), $^{t}BuCOCl$ (33 μL, 0.27 mmol) in CH_2Cl_2 (3 mL) followed by (*S*)-(–)-tetramisole hydrochloride **5** (2.2 mg, 9.0 μmol) and $^{i}Pr_2NEt$ (78 μL, 0.45 mmol) were reacted according to general procedure 4 followed by addition of MeOH. The crude product (>99:1 $dr_{syn:anti}$) was purified by column chromatography (Petrol : EtOAc 80:20) to give the title compound **22** (40 mg, 64%) as a white solid. mp 110–112 °C; $[\alpha]_D^{20}$ –16.8 (*c* 0.25 in CH_2Cl_2); Chiral HPLC analysis, Chiralpak AD-H (97:3 hexane : IPA, flow rate 1.0 mLmin^{-1}, 211 nm, 30 °C) t_R (2*R*,3*S*): 27.2 min, t_R (2*S*,3*R*): 30.5 min, 94% ee; ν_{max} (film) 1749 (C=O), 1684 (C=O), 1327, 1173, 1132; ^{1}H NMR (500 MHz, $CDCl_3$) δ_H: 3.31 (1H, dd, *J* 18.0, 6.3, CH^AH^B), 3.40 (1H, dd, *J* 18.0, 7.9, CH^AH^B), 3.64 (3H, s, CO_2CH_3), 4.47–4.53 (1H, m, C(3)*H*), 5.39 (1H, d, *J* 9.0, C(2)*H*), 6.89–6.95 (2H, m, Ar*H* (×2)), 7.14–7.22 (2H, m, Ar*H* (×2)), 7.73 (2H, d, *J* 8.1, ArC(2',6')*H*), 8.03 (2H, d, *J* 8.1, ArC(3',5')*H*); $^{13}C\{^1H\}$ NMR (125 MHz, $CDCl_3$) δ_C: 40.2 (*C*(3)H), 40.7 (CH_2), 52.4 (CO_2CH_3), 82.6 (*C*(2)H), 110.4 (Ar*C*H), 121.8 (Ar*C*H), 123.6 (q, $^1J_{CF}$ 272.4, CF_3), 124.4 (Ar*C*H), 126.0 (q, $^3J_{CF}$ 3.6, Ar*C*(3',5')H), 128.3 (Ar*C*(3a)), 128.5 (Ar*C*(2',6')H), 129.3 (Ar*C*H), 134.9 (q, $^2J_{CF}$ 32.5, Ar*C*(4')), 139.2 (Ar*C*(1')), 158.8 (Ar*C*(7a)), 170.1 (CO_2CH_3), 196.7 (*C*O); $^{19}F\{^1H\}$ NMR (470 MHz, $CDCl_3$) δ_F: –63.2; *m/z* (ES^-) 363 ($[M–H]^-$, 100%); HRMS (ES^-) $C_{19}H_{14}F_3O_4$ $[M–H]^-$ found 363.0852, requires 363.0844 (+2.3 ppm).

(2*R*,3*S*)-Methyl-3-(2-(4-chlorophenyl)-2-oxoethyl)-2,3-dihydrobenzofuran-2-carboxylate (23)

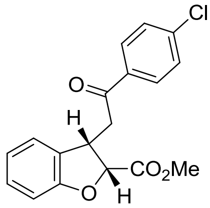

As reported previously,[6] enone-acid **S7** (57 mg, 0.18 mmol), $^{i}Pr_2NEt$ (37 μL, 0.20 mmol), $^{t}BuCOCl$ (26 μL, 0.22 mmol) in CH_2Cl_2 (3 mL) followed by (*S*)-(–)-tetramisole hydrochloride **5** (2.2 mg, 9.0 μmol) and $^{i}Pr_2NEt$ (76 μL, 0.45 mmol) were reacted according to general procedure 4 followed by addition of MeOH. The crude product (>99:1 $dr_{syn:anti}$) was purified by column chromatography (Petrol : EtOAc 80:20) to give the title compound **23** (37 mg, 63%) as a white solid, with data in

accordance with the literature. mp 120–122 °C; $[\alpha]_D^{20}$ +20.0 (*c* 0.5 in $CHCl_3$); Chiral HPLC analysis, Chiralpak AD-H (85:15 hexane : IPA, flow rate 1.0 $mLmin^{-1}$, 254 nm) t_R (2*R*,3*S*): 15.9 min, t_R (2*S*,3*R*): 19.2 min, 85% ee; ν_{max} (KBr) 1750 (C=O ester), 1677 (C=O ketone); ^1H NMR (400 MHz, $CDCl_3$) δ_H: 3.25 (1H, dd, *J* 17.8, 6.4, CH^AH^B), 3.33 (1H, dd, *J* 17.8, 7.8, CH^AH^B), 3.63 (3H, s, CO_2CH_3), 4.45-4.53 (1H, m, C(3)*H*), 5.38 (1H, d, *J* 9.0, C(2)*H*), 6.87-6.94 (2H, m, Ar*H* (×2)), 7.12-7.21 (2H, m, Ar*H* (×2)), 7.44 (2H, d, *J* 8.7, ArC(3',5')*H*), 7.86 (2H, d, *J* 8.7, ArC(2',6')*H*); $^{13}C\{^1H\}$ NMR (100 MHz, $CDCl_3$) δ_C: 40.2 (*C*H_2), 40.3 (*C*(3)H), 52.4 (CO_2CH_3), 82.7 (*C*(2)H), 110.3 (Ar*C*(7)H), 121.7 (Ar*C*(5)H), 124.5 (Ar*C*(4)H), 128.5 (Ar*C*(3a)), 129.2 (Ar*C*H (×3)), 129.6 (Ar*C*H (×2)), 135.0 (Ar*C*(1')), 140.1 (Ar*C*(4')), 158.8 (Ar*C*(7a)), 170.1 (*C*O_2CH_3), 196.3 (*C*O); *m/z* (ES^+) 353 ($[M(^{35}Cl)+Na]^+$, 90%), 331 ($[M(^{35}Cl)+H]^+$, 100%); HRMS (ES^+) $C_{18}H_{16}O_4{}^{35}Cl$ $[M+H]^+$ found 331.0745, requires 331.0737 (+1.6 ppm).

(2*R*,3*S*)-Methyl-3-(2-(naphthalen-2-yl)-2-oxoethyl)-2,3-dihydrobenzofuran-2-carboxylate (24)

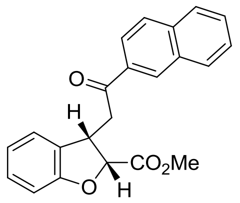

Enone-acid **S8** (60 mg, 0.18 mmol), $^iPr_2NEt$ (35 μL, 0.19 mmol), ^tBuCOCl (33 μL, 0.27 mmol) in CH_2Cl_2 (3 mL) followed by (*S*)-(–)-tetramisole hydrochloride **5** (2.2 mg, 9.0 μmol) and $^iPr_2NEt$ (78 μL, 0.45 mmol) were reacted according to general procedure 4 followed by addition of MeOH. The crude product (>99:1 $dr_{syn:anti}$) was purified by column chromatography (Petrol : EtOAc 80:20) to give the title compound **24** (40 mg, 64%) as a white solid. mp 114–116 °C; $[\alpha]_D^{20}$ –38.9 (*c* 0.5 in $CHCl_3$); Chiral HPLC analysis, Chiralpak AD-H (85:15 hexane : IPA, flow rate 1.0 $mLmin^{-1}$, 254 nm) t_R (2*S*,3*R*): 16.4 min, t_R (2*R*,3*S*): 18.2 min, 95% ee; ν_{max} (KBr) 1751 (C=O ester), 1677 (C=O ketone); ^1H NMR (300 MHz, $CDCl_3$) δ_H: 3.43 (1H, dd, *J* 17.8, 6.6 CH^AH^B), 3.52 (1H, dd, *J* 17.8, 7.7, CH^AH^B), 3.60 (3H, s, CO_2CH_3), 4.53–4.64 (1H, m, C(3)*H*), 5.43 (1H, d, *J* 9.1, C(2)*H*), 6.87–6.97 (2H, m, Ar*H* (×2)), 7.15–7.23 (2H, m, Ar*H* (×2)), 7.51–7.65 (2H, m, Ar*H* (×2)), 7.84–7.97 (3H, m, Ar*H* (×3)), 8.02 (1H, dd, *J* 8.6, 1.7, Ar*H*), 8.39–8.44 (1H, br. m, Ar*H*); $^{13}C\{^1H\}$ NMR (75 MHz, $CDCl_3$) δ_C: 40.4 (*C*(3)H and *C*H_2), 52.3 (CO_2CH_3), 82.7 (*C*(2)H), 110.2 (Ar*C*H), 121.6 (Ar*C*H), 123.7 (Ar*C*H), 124.5 (Ar*C*H), 127.0 (Ar*C*H), 127.9 (Ar*C*H), 128.7 (Ar*C*(3a)), 128.7 (Ar*C*H), 128.8 (Ar*C*H), 129.1 (Ar*C*H), 129.7 (Ar*C*H), 129.9 (Ar*C*H), 132.5 (Ar*C*), 133.9 (Ar*C*), 135.8 (Ar*C*(1')), 158.8 (Ar*C*(7a)), 170.1 (*C*O_2CH_3), 197.4 (*C*O); *m/z* (ES^+) 347 ($[M+H]^+$, 100%); HRMS (ES^+) $C_{22}H_{19}O_4$ $[M+H]^+$ found 347.1283, requires 347.1278 (+1.5 ppm).

(2*R*,3*S*)-Methyl 3-(2-oxopropyl)-2,3-dihydrobenzofuran-2-carboxylate (25)

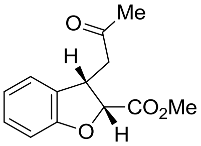

As reported previously,[6] enone-acid **S9** (40 mg, 0.18 mmol), $^{i}Pr_2NEt$ (37 μL, 0.20 mmol), ^{t}BuCOCl (26 μL, 0.22 mmol) in CH_2Cl_2 (3 mL) followed by (*S*)-(–)-tetramisole hydrochloride **5** (2.2 mg, 9.0 μmol) and $^{i}Pr_2NEt$ (76 μL, 0.45 mmol) were reacted according to general procedure 4 followed by addition of MeOH. The crude product (>99:1 $dr_{syn:anti}$) was purified by column chromatography (Petrol : EtOAc 80:20) to give the title compound **25** (37 mg, 87%) as a clear oil, with data in accordance with the literature. $[\alpha]_D^{20}$ + 35.5 (*c* 1.0 in $CHCl_3$); Chiral HPLC analysis, Chiralpak AD-H (85:15 hexane : IPA, flow rate 1.0 $mLmin^{-1}$, 254 nm) t_R (2*S*,3*R*): 7.8 min, t_R (2*R*,3*S*): 8.8 min, 95% ee; ν_{max} (film) 1751 (C=O ester), 1718 (C=O ketone), 1207 (C-O); ^{1}H NMR (300 MHz, $CDCl_3$) δ_H: 2.14 (3H, s, CH_2COCH_3), 2.77 (2H, d, *J* 7.2, CH_2), 3.76 (3H, s, CO_2CH_3), 4.25 (1H, dd, *J* 16.2, 7.2, C(3)*H*), 5.27 (1H, d, *J* 9.0, C(2)*H*), 6.83–6.92 (2H, m, Ar*H*), 7.06–7.20 (2H, m, Ar*H*); $^{13}C\{^1H\}$ NMR (75 MHz, $CDCl_3$) δ_C: 30.4 (CH_2COCH_3), 39.8 (*C*(3)H), 44.9 (CH_2), 52.3 (CO_2CH_3), 82.5 (*C*(2)H), 110.1 (Ar*C*(7)H), 121.6 (Ar*C*(5)H), 124.3 (Ar*C*(4)H), 128.5 (Ar*C*(3a)), 129.0 (Ar*C*(6)H), 158.6 (Ar*C*(7a)), 170.0 (CO_2CH_3), 205.9 (*C*O); *m/z* (ES^+) 252 ($[M+NH_4]^+$, 100%), 235 ($[M+H]^+$, 67%); HRMS (ES^+) $C_{13}H_{15}O_4$ $[M+H]^+$ found 235.0967, requires 235.0965 (+ 0.9 ppm).

Data for anti-2,3-Dihydrobenzofurans

(2*S*,3*S*)-Methyl 3-(2-oxo-2-phenylethyl)-2,3-dihydrobenzofuran-2-carboxylate (11)

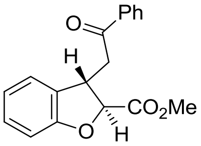

Enone-acid **S3** (30 mg, 0.11 mmol) in CH_2Cl_2 (2 mL), $^{i}Pr_2NEt$ (45 µL, 0.27 mmol), Mukaiyama derivative **8** (54 mg, 0.16 mmol) and OTMS-quinidine **19** (7.80 mg, 0.02 mmol) in CH_2Cl_2 (1 mL) were reacted according to general procedure 5 followed by addition of MeOH. The crude product (20:80 $dr_{syn:anti}$) was purified by column chromatography (Petrol: EtOAc, 90:10) to give:

Anti-(2*S*,3*S*) **11** (17.2 mg, 53%) as a colourless oil. $[\alpha]_D^{20}$ −81.1 (*c* 0.3, $CHCl_3$); Chiral HPLC analysis, Chiralpak AD-H (85:15 hexane : IPA, flow rate 1.0 $mLmin^{-1}$, 254 nm, 30 °C) t_R (2*R*,3*R*): 15.1 min, t_R (2*S*, 3*S*): 20.4 min, 98% ee; ν_{max} (KBr) 1740 (C=O, ester), 1680 (C=O, ketone); ^{1}H NMR (300 MHz, $CDCl_3$) δ_H: 3.35 (1H, dd, *J* 17.8, 7.3, CH^AH^B), 3.48 (1H, dd, *J* 17.8, 6.6, CH^AH^B), 3.76 (3H, s, OCH_3), 4.24 (1H, app. q, *J* 6.5, C(3)*H*), 4.84 (1H, d, *J* 5.9, C(2)*H*), 6.80–6.86 (2H, m, Ar*H*), 7.09–7.14 (2H, m, Ar*H*), 7.38–7.43 (2H, m, Ar*H*), 7.49–7.52 (1H, m, Ar*H*), 7.89–7.92 (2H, m, Ar*H*); $^{13}C\{^1H\}$ NMR (100 MHz, $CDCl_3$) δ_C: 42.0 (*C*(3)H), 44.4 (CH_2), 52.8 (OCH_3), 84.8 (*C*(2)H), 110.2 (Ar*C*H), 121.7 (Ar*C*H), 124.8 (Ar*C*H), 128.2 (Ar*C*(3',5')H), 128.4 (Ar*C*H), 128.9 (Ar*C*(2',6')H), 129.1 (Ar*C*(3a)), 133.7 (Ar*C*(4)H), 136.5 (Ar*C*(1')), 158.9 (Ar*C*(7a)), 171.1 (CO_2CH_3), 197.6 (*C*OAr); *m/z* (ES^+) 297 ($[M+H]^+$, 100 %), HRMS (ES^+) $C_{18}H_{17}O_4$ $[M+H]^+$ found 297.1124, requires 297.1121 (+0.9 ppm).

Syn-(2*S*,3*R*) **10** (2.7 mg, 9%) as a white solid with data in accordance with the literature.[6] mp 112–114 °C {Lit.[6] 114–116 °C}; $[\alpha]_D^{20}$ −5.2 (*c* 0.1, $CHCl_3$); {Lit.[6] (2*R*,3*S*) $[\alpha]_D^{20}$ +21.4 (*c* 1.0, $CHCl_3$)}; Chiral HPLC analysis, Chiralpak AD-H (85:15 hexane : IPA, flow rate 1.0 $mLmin^{-1}$, 254 nm, 30 °C) t_R (2*S*, 3*R*): 12.4 min, t_R (2*R*, 3*S*) 16.7 min, 63% ee; ^{1}H NMR (400 MHz, $CDCl_3$) δ_H: 3.22 (1H, dd, *J* 17.9, 6.5, CH^AH^B), 3.30 (1H, dd, *J* 17.9, 7.6, CH^AH^B), 3.55 (3H, s, OCH_3), 4.45 (1H, app. q, *J* 7.9 C(3)*H*), 5.32 (1H, d, *J* 9.2, C(2)*H*), 6.81–6.86 (2H, m, Ar*H*), 7.09–7.14 (2H, m, Ar*H*), 7.38–7.41 (2H, m, Ar*H*), 7.49–7.53 (1H, tt, *J* 7.8, 1.3 Ar*H*), 7.93 (2H, m, Ar*H*).

(2*S*,3*S*)-Methyl 3-(2-(4-methoxyphenyl)-2-oxoethyl)-2,3-dihydrobenzofuran-2-carboxylate (26)

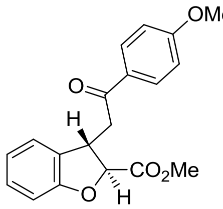

Enone-acid **S4** (2.00 g, 6.4 mmol) in CH_2Cl_2 (20 mL), $^iPr_2NEt$ (2.79 mL, 16.0 mmol), Mukaiyama derivative **8** (3.23 g, 9.6 mmol) and OTMS-quinidine **19** (0.51 g, 1.28 mmol) in CH_2Cl_2 (10 mL) were reacted according to general procedure 5 followed by addition of MeOH. The crude product (15:85 $dr_{syn:anti}$) was purified by column chromatography (Petrol: EtOAc, 90:10) to give:

Anti-(2*S*,3*S*) **26** (14:86 $dr_{syn:anti}$, 1.25 g, 60%) as a colourless oil. $[\alpha]_D^{20}$ +147.9 (*c* 0.5, $CHCl_3$); Chiral HPLC analysis, Chiralpak AD-H (85:15 hexane : IPA, flow rate 0.5 $mLmin^{-1}$, 254 nm, 30 °C) t_R (2*R*,3*R*): t_R (2*S*,3*S*): 48.5 min, t_R (2*R*,3*R*): 53.3 min, 99% ee; ν_{max} (KBr) 1751 (C=O, ester), 1673 (C=O, ketone); ^{1}H NMR (300 MHz, $CDCl_3$) δ_H: 3.34 (1H, dd, *J* 17.6, 7.6 CH^AH^B), 3.50 (1H, dd, *J* 17.6, 6.8, CH^AH^B), 3.82 (3H, s, CO_2CH_3), 3.86 (3H, s, OCH_3), 4.29 (1H, q, *J* 6.8, C(3)*H*), 4.90 (1H, d, *J* 5.6, C(2)*H*), 6.87–6.95 (4H, m, Ar*H*), 7.15–7.19 (2H, m, Ar*H*), 7.93–7.96 (2H, m, Ar*H*); $^{13}C\{^1H\}$ NMR (75 MHz, $CDCl_3$) δ_C: 42.1 (*C*(3)H), 44.1 (CH_2), 52.8 (OCH_3), 55.7 ($ArOCH_3$), 84.9 (*C*(2)H), 110.1 (Ar*C*H), 114.0 (*C*(3',5')H), 121.6 (Ar*C*H), 124.8 (Ar*C*H), 128.8 (Ar*C*(3a)), 129.0 (Ar*C*H), 129.7 (Ar*C*(1')CO), 130.5 (Ar*C*(2',6')H), 158.9 (Ar*C*(7a)), 163.9 (Ar*C*(4')) 171.1 (CO_2CH_3), 196.0 (*C*OAr); *m/z* (ES^+) 670 ($[2M+NH_4]^+$, 50%), 327 ($[M+H]^+$, 100%); HRMS (ES^+) $C_{19}H_{19}O_5$ $[M+H]^+$ found 327.1231, requires 327.1227 (+1.2 ppm);

Syn-(2*S*,3*R*) *ent*-**20** (0.21 g, 10%) as a white solid. mp 98–100 °C; $[\alpha]_D^{20}$ −23.0 (*c* 0.3, $CHCl_3$); Chiral HPLC analysis, Chiralpak AD-H (85:15 hexane : IPA, flow rate 0.5 $mLmin^{-1}$, 254 nm, 30 °C) t_R (2*R*,3*S*): 37.1 min, t_R (2*S*,3*R*): 42.8 min, 51% ee; ν_{max} (KBr) 1751 (C=O, ester), 1671 (C=O, ketone); ^{1}H NMR (300 MHz, $CDCl_3$) δ_H: 3.18-3.35 (2H, m, CH_2), 3.61 (3H, s, CO_2CH_3), 3.87 (3H, s, OCH_3), 4.50 (1H, app. q, *J* 7.2, C(3)*H*), 5.38 (1H, d, *J* 9.2, C(2)*H*), 6.86–6.95 (4H, m, Ar*H*), 7.15–7.20 (2H, m, Ar*H*), 7.88–7.93 (2H, m, Ar*H*); $^{13}C\{^1H\}$ NMR (75 MHz, $CDCl_3$) δ_C: 39.9 (CH_2), 40.3 (*C*(3)H), 52.3 (CO_2CH_3), 55.6 (OCH_3), 82.8 (*C*(2)H), 110.2 (Ar*C*H), 113.9 (Ar*C*(3',5')H), 121.6 (Ar*C*H), 124.5 (Ar*C*H), 128.8 (Ar*C*(3a)), 129.0 (Ar*C*H), 129.7 (Ar*C*(1')), 130.4 (Ar*C*(2',6')H), 158.8 (Ar*C*(7a)), 163.9 (Ar*C*(4')), 170.1 (CO_2CH_3), 195.9 (*C*OAr); *m/z* (ES^+) 670 ($[2M+NH_4]^+$, 15%), 349 ($[M+Na]^+$, 20%), 327 ($[M+H]^+$, 100%); HRMS (ES^+) $C_{19}H_{19}O_5$ $[M+H]^+$ found 327.1231, requires 327.1227 (+1.2 ppm);

(2*S*,3*S*)-Methyl 3-(2-oxo-2-(*p*-tolyl)ethyl)-2,3-dihydrobenzofuran-2-carboxylate (27)

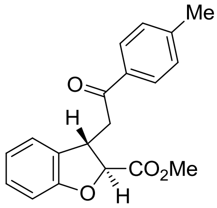

Enone-acid **S5** (150 mg, 0.51 mmol) in CH_2Cl_2 (2 mL), $^iPr_2NEt$ (220 μL, 1.27 mmol), Mukaiyama derivative **8** (255 mg, 0.76 mmol) and OTMS-quinidine **19** (40 mg, 0.10 mmol) in CH_2Cl_2 (1 mL) were reacted according to general procedure 5 followed by addition of MeOH. The crude product (18:82 $dr_{syn:anti}$) was purified by column chromatography (Petrol: EtOAc, 90:10) to give:

Anti-(2*S*,3*S*) **27** (72 mg, 45%) as a colourless oil. $[\alpha]_D^{20}$ +168.2 (*c* 0.6, $CHCl_3$); Chiral HPLC analysis, Chiralpak AD-H (85:15 hexane : IPA, flow rate 0.5 $mLmin^{-1}$, 254 nm, 30 °C) t_R (2*R*,3*R*): 29.4 min, t_R (2*S*,3*S*): 32.0 min, 98% ee; ν_{max} (KBr) 1751 (C=O, ester), 1680 (C=O, ketone); ^{1}H NMR (400 MHz, $CDCl_3$) δ_H: 2.41 (3H, s, C*H₃*), 3.37 (1H, dd, *J* 18.0, 7.2, CH^AH^B), 3.52 (1H, dd, *J* 18.0, 6.8, CH^AH^B), 3.82 (3H, s, OCH_3), 4.30 (1H, app. q, *J* 6.5, C(3)*H*), 4.90 (1H d, *J* 5.9, C(2)*H*), 6.87–6.92 (2H, m, Ar*H*), 7.15–7.19 (2H, m, Ar*H*), 7.25–7.27 (2H, m, Ar*H*), 7.86–7.88 (2H, m, Ar*H*); $^{13}C\{^1H\}$ NMR (100 MHz, $CDCl_3$) δ_C: 21.8 (CH_3), 42.1 (*C*(3)H), 44.3 (CH_2), 52.8 (OCH_3), 84.9 (*C*(2)H), 110.1 (Ar*C*H), 121.6 (Ar*C*H), 124.8 (Ar*C*H), 128.4 (Ar*C*(2',6')H), 128.5 (Ar*C*H), 129.1 (Ar*C*(3a)), 129.6 (Ar*C*(3',5')H), 134.1 (Ar*C*(1')), 144.6 (Ar*C*(4')CH_3), 158.9 (Ar*C*(7a)), 171.1 (CO_2CH_3), 197.2 (*C*OAr); *m/z* (ES^+) 311 ($[M+H]^+$, 100 %), HRMS (ES^+) $C_{19}H_{19}O_4$ $[M+H]^+$ found 311.1280, requires 311.1278 (+0.7 ppm);

Syn-(2*S*,3*R*) *ent*-**21** (23 mg, 15%) as a white solid with data in accordance with the literature.[6] mp 93 °C {Lit.[6] 90–92 °C};$[\alpha]_D^{20}$ −25.9 (*c* 0.4, $CHCl_3$) {Lit.[6] (2*R*,3*S*) $[\alpha]_D^{20}$ +18.0 (*c* 0.5, $CHCl_3$)}; Chiral HPLC analysis, Chiralpak AD-H (85:15 hexane : IPA, flow rate 0.5 $mLmin^{-1}$, 240 nm, 30 °C) t_R (2*R*,3*S*): 25.4 min, t_R (2*S*,3*R*): 28.1 min, 63% ee; ^{1}H NMR (300 MHz, $CDCl_3$) δ_H: 2.41 (3H, s, CH_3Ar), 3.27 (1H, dd, *J* 17.8, 6.5, CH^AH^B), 3.33 (1H, dd, *J* 17.8, 7.5, CH^AH^B), 3.61 (3H, s, OCH_3), 4.46–4.54 (1H, m, C(3)*H*), 5.39 (1H, d, *J* 9.5, C(2)*H*), 6.87–6.92 (2H, m, Ar*H*), 7.15–7.19 (2H, m, Ar*H*), 7.25–7.26 (2H, m, Ar*H*), 7.81–7.83 (2H, m, Ar*H*).

(2*S*,3*S*)-Methyl3-(2-oxo-2-(4-(trifluoromethyl)phenyl)ethyl)-2,3-dihydrobenzofuran-2-carboxylate (28)

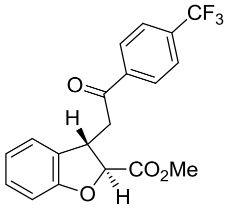

Enone-acid **S6** (50.0 mg, 0.14 mmol) in CH_2Cl_2 (1 mL), $^iPr_2NEt$ (62 µL, 0.35 mmol), Mukaiyama derivative **8** (71.9 mg, 0.21 mmol) and OTMS-quinidine **19** (11.3 mg, 0.03 mmol) in CH_2Cl_2 (2 mL) were reacted according to general procedure 5 followed by addition of MeOH. The crude product (37:63 $dr_{syn:anti}$) was purified by column chromatography (Petrol: EtOAc, 80:10) to give:

Anti-(2*S*,3*S*) **28** (13:87 $dr_{syn:anti}$, 22.5 mg, 44%) as a colourless oil. $[\alpha]_D^{20}$ +78.8 (*c* 0.3, $CHCl_3$); Chiral HPLC analysis, Chiralpak AD-H (90:10 hexane : IPA, flow rate 1.0 mLmin^{-1}, 254 nm, 30 °C) t_R (2*S*,3*S*): 19.1 min, t_R (2*R*,3*R*): 21.7 min, 94% ee; ν_{max} (KBr) 1751 (C=O, ester), 1677 (C=O, ketone); ^{1}H NMR (300 MHz, $CDCl_3$) δ_H: 3.37 (1H, dd, *J* 17.9, 7.2 CH^AH^B) 3.53 (1H, dd, *J* 17.9, 6.6, CH^AH^B), 3.77 (3H, s, CO_2CH_3), 4.25 (1H, app. q, *J* 6.4, C(3)*H*), 4.84 (1H, d, *J* 5.8, C(2)*H*), 6.83–6.87 (2H, m, Ar*H*), 7.10–7.13 (2H, m, Ar*H*), 7.66–7.69 (2H, m, Ar*H*), 7.99–8.02 (2H, m, ArH); ^{13}C{^{1}H} NMR (125 MHz, $CDCl_3$) δ_C: 41.8 (*C*(3)H), 44.6 (CH_2), 52.9 (CO_2CH_3), 84.6 (*C*(2)H), 110.2 (Ar*C*H), 121.6 (Ar*C*H), 123.5 (q, $^1J_{CF}$ 271.2, CF_3), 124.6 (Ar*C*H), 125.9 (Ar*C*H), 127.9 (Ar*C*(3a)), 128.5 (Ar*C*(2',6')H), 129.2 (Ar*C*H), 134.8 (q, $^2J_{CF}$ 32.8, *C*(4')CF_3), 138.9 (Ar*C*(1')), 158.7 (Ar*C*(7a)), 170.9 (CO_2CH_3), 196.6 (*C*OAr); ^{19}F{^{1}H} NMR (470 MHz, $CDCl_3$) δ_F: –63.2; *m/z* (ES^-) 363 ($[M-H]^-$, 100%); HRMS (ES^-) $C_{19}H_{14}F_3O_4$ $[M-H]^-$ found 363.0847, requires 363.0844 (+0.8 ppm);

Syn-(2*S*,3*R*) *ent*-**22** (6.1 mg, 11%) as a white solid. mp 110–112 °C; $[\alpha]_D^{20}$ +16.0 (*c* 0.05, CH_2Cl_2); Chiral HPLC analysis, Chiralpak AD-H (97:3 hexane : IPA, flow rate 1.0 mLmin^{-1}, 254 nm, 30 °C) t_R (2*S*,3*R*): 27.4 min, t_R (2*R*, 3*S*): 31.1 min, 48% ee; ν_{max} (KBr) 1751 (C=O, ester), 1677 (C=O, ketone); ^{1}H NMR (500 MHz, $CDCl_3$) δ_H: 3.22 (1H, dd, *J* 11.4, 6.6 CH^AH^B), 3.35 (1H, dd, *J* 11.4, 7.5, CH^AH^B), 3.57 (3H, s, CO_2CH_3), 4.43 (1H, q, *J* 7.8, C(3)*H*), 5.32 (1H, d, *J* 9.1, C(2)*H*), 6.82–6.87 (2H, m, Ar*H*), 7.08–7.19 (2H, m, Ar*H*), 7.66 (2H, d, *J* 8.2, Ar*H*), 7.96 (2H, d, *J* 8.2, Ar*H*); ^{13}C{^{1}H} NMR (125 MHz, $CDCl_3$) δ_C: 40.0 (*C*(3)H), 40.6 (CH_2), 52.3 (CO_2CH_3), 82.5 (*C*(2)H), 110.3 (Ar*C*H), 121.6 (Ar*C*H), 123.5 (q, $^1J_{CF}$ 271.2, CF_3), 124.3 (Ar*C*H), 125.8 (Ar*C*H), 128.2 (Ar*C*(3a)), 128.3 (Ar*C*(2',6')H), 129.2 (Ar*C*H), 134.7 (q, $^2J_{CF}$ 32.6, *C*(4')CF_3), 139.1 (Ar*C*(1')), 158.6 (Ar*C*(7a)), 169.9 (CO_2CH_3), 196.6 (*C*OAr); ^{19}F{^{1}H} NMR (470 MHz, $CDCl_3$) δ_F: –63.2; *m/z* (ES^-) 363 ($[M-H]^-$, 100%); HRMS (ES^-) $C_{19}H_{14}F_3O_4$ $[M-H]^-$ found 363.0852, requires 363.0844 (+2.3 ppm).

(2*S*,3*S*)-Methyl 3-(2-(4-chlorophenyl)-2-oxoethyl)-2,3-dihydrobenzofuran-2-carboxylate (29)

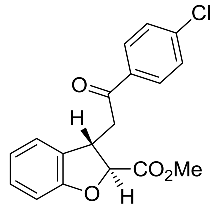

Enone-acid **S7** (150 mg, 0.51 mmol) in CH_2Cl_2 (1 mL), $^iPr_2NEt$ (200 μL, 1.18 mmol), Mukaiyama derivative **8** (238 mg, 0.71 mmol) and OTMS-quinidine **19** (37.6 mg, 0.09 mmol) in CH_2Cl_2 (2 mL) were reacted according to general procedure 5 followed by addition of MeOH. The crude product (26:74 $dr_{syn:anti}$) was purified by column chromatography (Petrol: EtOAc, 90:10) to give:

Anti-(2*S*,3*S*) **29** (72 mg, 43%) as a colourless oil. $[\alpha]_D^{20}$ +144.1 (*c* 0.6, $CHCl_3$); Chiral HPLC analysis, Chiralpak AD-H (85:15 hexane : IPA, flow rate 0.5 $mLmin^{-1}$, 254 nm, 30 °C) t_R (2*S*,3*S*): 34.3 min, t_R (2*R*,3*R*): 37.7min, 96% ee; ν_{max} (KBr) 1751 (C=O, ester), 1685 (C=O, ketone); ^{1}H NMR (300 MHz, $CDCl_3$) δ_H: 3.37 (1H, dd, *J* 18.0, 7.2 CH^AH^B), 3.51 (1H, dd, *J* 18.0, 6.3, CH^AH^B), 3.82 (3H, s, C*H*$_3$), 4.30 (1H, app. q, *J* 6.3, C(3)*H*), 4.89 (1H, d, *J* 6.0, C(2)*H*), 6.87–6.92 (2H, m, Ar*H*), 7.15–7.20 (2H, m, Ar*H*), 7.42–7.47 (2H, m, ArC(3',5')*H*), 7.88–7.92 (2H, m, ArC(2',6')*H*); $^{13}C\{^1H\}$ NMR (75 MHz, $CDCl_3$) δ_C: 41.9 (*C*(3)H), 44.3 (*C*H_2), 52.8 (O*C*H_3), 84.7 (*C*(2)H), 110.2 (Ar*C*H), 121.6 (Ar*C*H), 124.7 (Ar*C*H), 128.2 (Ar*C*(3a)), 129.2 (Ar*C*(3',5')H), 129.2 (Ar*C*H), 129.6 (Ar*C*(2',6')H), 134.8 (Ar*C*(1')), 140.1 (Ar*C*(4')), 158.8 (Ar*C*(7a)), 170.9 (*C*O_2CH_3), 196.3 (*C*OAr); *m/z* (ES^+) 683 ($[2M(^{35}Cl)+Na]^+$, 35 %), 348 ($[M(^{35}Cl)+NH_4]^+$, 50 %), 331 ($[M(^{35}Cl)+H]^+$, 100 %), HRMS (ES^+) $C_{18}H_{16}O_4{}^{35}Cl$ $[M+H]^+$ found 311.0736, requires 331.0732 (+1.3 ppm);

Syn-(2*S*,3*R*) *ent*-**23** (28 mg, 17%) as a white solid with data in accordance with the literature.[6] mp 118–120 °C {Lit.[6] 120–122 °C}; $[\alpha]_D^{20}$ −21.5 (*c* 0.5, $CHCl_3$) {Lit.[6] (2*R*,3*S*) $[\alpha]_D^{20}$ +20.0 (*c* 0.5, $CHCl_3$)}; Chiral HPLC analysis, Chiralpak AD-H (85:15 hexane : IPA, flow rate 0.5 $mLmin^{-1}$, 254 nm, 30 °C) t_R (2*R*,3*S*): 27.6 min, t_R (2*S*,3*R*): 32.6 min, 42% ee; ^{1}H NMR (400 MHz, $CDCl_3$) δ_H: 3.25 (1H, dd, *J* 17.8, 6.4, CH^AH^B), 3.34 (1H, dd, *J* 17.8, 7.8, CH^AH^B), 3.63 (3H, s, OC*H*$_3$), 4.48 (1H, app. q, *J* 7.8, C(3)*H*), 5.38 (1H, d, *J* 8.8, C(2)*H*), 6.88–6.93 (2H, m, Ar*H*), 7.13–7.21 (2H, m, Ar*H*), 7.43–7.45 (2H, m, ArC(3',5')*H*), 7.85–7.88 (2H, m, ArC(2',6')*H*).

(2*S*,3*S*)-Methyl 3-(2-(naphthalen-2-yl)-2-oxoethyl)-2,3-dihydrobenzofuran-2-carboxylate (30)

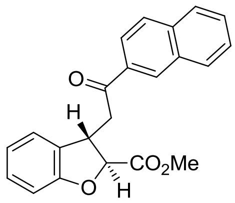

Enone-acid **S8** (150 mg, 0.45 mmol) in CH_2Cl_2 (1 mL), $^iPr_2NEt$ (190 μL, 1.13 mmol), Mukaiyama derivative **8** (227 mg, 0.68 mmol) and OTMS-quinidine **19** (35.8 mg, 0.10 mmol) in CH_2Cl_2 (2 mL) were reacted according to general procedure 5 followed by addition of MeOH. The crude product (23:77 $dr_{syn:anti}$) was purified by column chromatography (Petrol: EtOAc, 90:10) to give:

Anti-(2*S*,3*S*) **30** (9:91 $dr_{syn:anti}$, 53 mg, 34%) as a colourless oil. $[\alpha]_D^{20}$ +252.0 (*c* 0.3, $CHCl_3$); Chiral HPLC analysis, Chiralpak AD-H (85:15 hexane : IPA, flow rate 1.0 $mLmin^{-1}$, 254 nm, 30 °C) t_R (2*S*,3*S*): 23.5 min, t_R (2*R*,3*R*): 27.6 min), 97% ee; ν_{max} (KBr) 1748 (C=O, ester), 1678 (C=O, ketone); ^1H NMR (300 MHz, $CDCl_3$) δ_H: 3.55 (1H, dd, *J* 17.6, 7.2 CH^AH^B), 3.71 (1H, dd, *J* 17.6, 6.4, CH^AH^B), 3.84 (3H, s, CO_2CH_3), 4.37 (1H, q, *J* 6.4, C(3)*H*), 4.97 (1H, d, *J* 5.8, C(2)*H*), 6.89–6.94 (2H, m, Ar*H*), 7.17–7.24 (2H, m, Ar*H*), 7.56–7.62 (2H, m, Ar*H*), 7.87–7.96 (3H, m, ArH), 8.04–8.07 (1H, m, Ar*H*), 8.48 (1H, s, Ar*H*); $^{13}C\{^1H\}$ NMR (75 MHz, $CDCl_3$) δ_C: 42.2 (*C*(3)H), 44.5 (CH_2), 52.8 (CO_2CH_3), 84.9 (*C*(2)H), 110.2 (Ar*C*H), 121.7 (Ar*C*H), 123.8 (Ar*C*H), 124.8 (Ar*C*H), 127.1 (Ar*C*H), 128.0 (Ar*C*H), 128.5 (Ar*C*(3a)), 128.8 (Ar*C*H), 128.9 (Ar*C*H), 129.1 (Ar*C*H), 129.7 (Ar*C*H), 130.1 (Ar*C*H), 132.6 (Ar*C*), 133.9 (Ar*C*), 135.9 (Ar*C*(1')), 158.9 (Ar*C*(7a)), 171.1 (CO_2CH_3), 197.5 (*C*OAr); *m/z* (ES^+) 347 ($[M+H]^+$, 100%); HRMS (ES^+) $C_{22}H_{19}O_4$ $[M+H]^+$ found 347.1283, requires 347.1278 (+1.5 ppm);

Syn-(2*S*,3*R*) *ent*-**24** (34 mg, 22%) as a white solid. mp 112–116 °C; $[\alpha]_D^{20}$ +5.3 (*c* 0.5, $CHCl_3$); Chiral HPLC analysis, Chiralpak AD-H (85:15 hexane : IPA, flow rate 1.0 $mLmin^{-1}$, 254 nm, 30 °C) t_R (2*S*,3*R*): 16.8min, t_R (2*R*,3*S*): 18.9 min, 57% ee; ν_{max} (KBr) 1751 (C=O, ester), 1677 (C=O, ketone); ^1H NMR (300 MHz, $CDCl_3$) δ_H: 3.47 (1H, dd, *J* 17.8, 6.5 CH^AH^B), 3.50 (1H, dd, *J* 17.8, 7.7, CH^AH^B), 3.60 (3H, s, CO_2CH_3), 4.58 (1H, q, *J* 7.8, C(3)*H*), 5.43 (1H, d, *J* 9.1, C(2)*H*), 6.88–6.96 (2H, m, Ar*H*), 7.16–7.22 (2H, m, Ar*H*), 7.55–7.63 (2H, m, Ar*H*), 7.86–7.95 (3H, m, ArH), 8.00–8.05 (1H, dd, *J* 8.6, 1.7, Ar*H*), 8.42 (1H, s, Ar*H*); $^{13}C\{^1H\}$ NMR (75 MHz, $CDCl_3$) δ_C: 40.4 (*C*(3)H), 40.4(CH_2), 52.3 (CO_2CH_3), 82.7 (*C*(2)H), 110.2 (Ar*C*H), 121.6 (Ar*C*H), 123.7 (Ar*C*H), 124.4 (Ar*C*H), 127.0 (Ar*C*H), 127.9 (Ar*C*H), 128.6 (Ar*C*(3a)), 128.7 (Ar*C*H), 128.8 (Ar*C*H), 129.1 (Ar*C*H), 129.7 (Ar*C*H), 129.9 (Ar*C*H), 132.5 (Ar*C*), 133.9 (Ar*C*), 135.8 (Ar*C*(1')), 158.8 (Ar*C*(7a)), 170.1 (CO_2CH_3), 197.4 (*C*OAr); *m/z* (ES^+) 347 ($[M+H]^+$, 100%); HRMS (ES^+) $C_{22}H_{19}O_4$ $[M+H]^+$ found 347.1283, requires 347.1278 (+1.5 ppm).

(2*S*,3*S*)-Methyl 3-(2-oxopropyl)-2,3-dihydrobenzofuran-2-carboxylate (31)

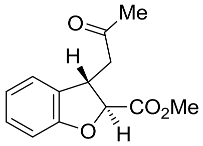

Enone-acid **S9** (150 mg, 0.68 mmol) in CH_2Cl_2 (2 mL), $^iPr_2NEt$ (300 μL, 1.70 mmol), Mukaiyama derivative **8** (342 mg, 1.02 mmol) and OTMS-quinidine **19** (54.0 mg, 0.14 mmol) in CH_2Cl_2 (1 mL) were reacted according to general procedure 5 followed by addition of MeOH (5 mL). The crude product (22:78 $dr_{syn:anti}$) was purified by column chromatography (Petrol: EtOAc, 90:10) to give the title compound (22:78 $dr_{syn:anti}$, 116 mg, 73%) as a clear oil. ν_{max} (film) 1747 (C=O, ester), 1714 (C=O, ketone);

*Data for major anti-(2S,3S) diastereoisomer **31***: Chiral HPLC analysis, Chiralpak AD-H (85:15 hexane : IPA, flow rate 1.0 mLmin^{-1}, 220 nm, 30 °C) t_R (2*S*,3*S*): 11.3 min, t_R (2*R*,3*R*): 12.0 min, 99% ee; ^{1}H NMR (400 MHz, $CDCl_3$) δ_H: 2.19 (3H, s, C*H₃*), 2.86 (1H, dd, *J* 17.9, 6.8, CH^AH^B), 2.97 (1H, dd, *J* 17.9, 7.0, CH^AH^B), 3.79 (3H, s, CO_2CH_3), 4.06 (1H, q, *J* 6.6, C(3)*H*), 4.77 (1H, d, *J* 6.1, C(2)*H*), 6.85–6.89 (2H, m, Ar*H* (×2)), 7.08–7.17 (2H, m, Ar*H* (×2)); ^{13}C{^{1}H} NMR (100 MHz, $CDCl_3$) δ_C: 30.3 (*C*H_3), 41.7 (*C*(3)H), 48.9 (*C*H_2), 52.7 (CO_2CH_3), 84.5 (*C*(2)H), 110.1 (Ar*C*(7)H), 121.6 (Ar*C*(5)H), 124.5 (Ar*C*(4)H), 128.2 (Ar*C*(3a)), 129.0 (Ar*C*(6)H), 158.6 (Ar*C*(7a)O), 171.0 (*C*O_2CH_3), 206.1 (*C*OCH_3);

*Data for minor syn-(2S,3R) diastereoisomer ent-**25***: Chiral HPLC analysis, Chiralpak AD-H (85:15 hexane : IPA, flow rate 1.0 mLmin^{-1}, 220 nm, 30 °C) t_R (2*S*,3*R*): 7.35 min, t_R (2*R*,3*S*): 8.44 min, 43% ee; ^{1}H NMR (400 MHz, $CDCl_3$) δ_H: 2.14 (3H, s, CH_3), 2.76 (2H, d, *J* 7.2, CH_2), 3.75 (3H, s, CH_3), 4.23 (1H, q, *J* 8.1, C(3)*H*), 5.25 (1H, d, *J* 9.0, C(2)*H*), 6.85–6.89 (2H, m, Ar*H*), 7.08–7.17 (2H, m Ar*H*); ^{13}C{^{1}H} NMR (100 MHz, $CDCl_3$) δ_C: 30.3 (*C*H_3), 39.8 (*C*(3)H), 44.8 (*C*H_2), 52.3 (CO_2CH_3), 82.4 (*C*(2)H), 110.0 (Ar*C*(7)H), 121.6 (Ar*C*(5)H), 124.3 (Ar*C*(4)H), 128.5 (Ar*C*(3a)), 129.0 (Ar*C*(6)H), 158.6 (*C*Ar(7a)O), 170.0 (*C*O_2CH_3), 205.9 (*C*OCH_3);

m/z (ES^+) 235 ($[M+H]^+$, 100%); HRMS (ES^+) $C_{13}H_{15}O_4$ $[M+H]^+$ found 235.0965, requires 235.0978 (+1.3ppm).

Data for syn-2,3-Tetrahydrofurans

(2*R*,3*R*)-*N*-Benzyl-3-(2-oxo-2-phenylethyl)tetrahydrofuran-2-carboxamide (32)

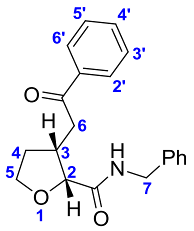

Enone-acid **S10** (90 mg, 0.38 mmol), $^{i}Pr_2NEt$ (100 μL, 0.58 mmol), $^{t}BuCOCl$ (95 μL, 0.77 mmol) in CH_2Cl_2 (3 mL) followed by (*S*)-(–)-tetramisole hydrochloride **5** (9 mg, 40 μmol) and $^{i}Pr_2NEt$ (167 μL, 0.96 mmol) were reacted according to general procedure 6 followed by addition of benzylamine (1.00 mL, 9.15mmol). The crude product (>99:1 $dr_{syn:anti}$) was purified by column chromatography (CH_2Cl_2 : EtOAc 90:10 to 70:30) to give the title compound **32** (124 mg, 70%) as a white solid. mp 118–120 °C; $[\alpha]_D^{20}$ –28.2 (*c* 1.0, CH_2Cl_2); Chiral HPLC analysis, Chiralpak IA (90:10 hexane : IPA, flow rate 1.0 mLmin^{-1}, 211 nm, 40 °C) t_R (2*R*,3*R*): 17.0 min, t_R (2*S*,3*S*): 21.1 min, >99% ee; ν_{max} (film) 3400 (N-H), 1682 (C=O, ketone), 1645 (C=O, amide), 1520 (C-N), 1082 (C-O, ether); ^{1}H NMR (300 MHz, $CDCl_3$) δ_H: 1.67 (1H, dq, *J* 12.7, 7.4, C(4)H^AH^B), 2.26 (1H, dtd, *J* 12.1, 7.0, 5.1, C(4)H^AH^B), 2.76 (1H, dd, *J* 17.1, 10.1, C(6)H^AH^B), 3.09–3.21 (1H, m, C(3)*H*), 3.38 (1H, dd, *J* 17.1, 4.3, C(6)H^AH^B), 3.88 (1H, dd, *J* 15.8, 7.6, C(5)H^AH^B), 4.07 (1H, ddd, *J* 8.3, 7.9, 4.9, C(5)H^AH^B), 4.37 (1H, dd, *J* 14.7, 5.7, C(7)H^AH^B), 4.51 (1H, d, *J* 7.6, C(2)*H*), 4.58 (1H, dd, *J* 14.7, 6.4, C(7)H^AH^B), 7.02–7.03 (1H, m, N*H*), 7.29–7.35 (5H, m, Ar*H*), 7.41–7.47 (2H, m, ArC(3')*H*), 7.56 (1H, tt, *J* 7.3, 1.3, ArC(4')*H*), 7.88 (2H, app. dt, *J* 8.4, 1.2, ArC(2')*H*); $^{13}C\{^{1}H\}$ NMR (75 MHz, $CDCl_3$) δ_C: 31.6 (*C*(4)H_2), 37.4 (*C*(3)H), 38.5 (*C*(6)H_2), 43.0 (*C*(7)H_2), 68.2 (*C*(5)H_2), 80.1 (*C*(2)H), 127.7 (Ar*C*(4")H), 128.0 (Ar*C*(2",6") H), 128.2 (Ar*C*(3",5")H), 128.7 (Ar*C*(3',5')H), 128.9 (Ar*C*(2',6')H), 133.3 (Ar*C*(4')H), 136.8 (Ar*C*(1')), 138.4 (Ar*C*(1")), 171.1 (*C*ON), 198.8 (*C*OAr); *m/z* (ES^+) 324 ($[M+H]^+$, 100%); HRMS (ES^+) $C_{20}H_{22}O_3N$ $[M+H]^+$ found 324.1598, requires 324.1594 (+1.2 ppm).

(2*R*,3*R*)-*N*-Benzyl-3-(2-(4-methoxyphenyl)-2-oxoethyl)tetrahydrofuran-2-carboxamide (33)

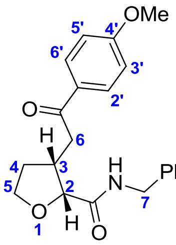

Enone-acid **S11** (100 mg, 0.38 mmol), $^{i}Pr_2NEt$ (99 μL, 0.57 mmol), $^{t}BuCOCl$ (93 μL, 0.76 mmol) in CH_2Cl_2 (3 mL) followed by (*S*)-(–)-tetramisole hydrochloride **5** (9 mg, 40 μmol) and $^{i}Pr_2NEt$ (165 μL, 0.95 mmol) were reacted according to general procedure 6 followed by addition of benzylamine (1.00 mL, 9.15mmol). The crude product (>99:1 $dr_{syn:anti}$) was purified by column chromatography (CH_2Cl_2 : EtOAc 90:10 to 70:30) to give the title compound **33** (83 mg, 62%) as a white solid. mp 119 °C; $[\alpha]_D^{20}$ −43.3 (*c* 0.5, CH_2Cl_2); Chiral HPLC analysis, Chiralpak IA (90:10 hexane : IPA, flow rate 1.0 $mLmin^{-1}$, 211 nm, 40 °C) t_R (2*R*,3*R*): 26.0 min, t_R (2*S*,3*S*): 31.3 min, 99% ee; ν_{max} (film) 3321 (N-H), 1678 (C=O, ketone), 1643 (C=O, amide), 1508 (C-N); ^{1}H NMR (400 MHz, $CDCl_3$) δ_H: 1.68 (1H, dq, *J* 12.6, 7.3, C(4)H^AH^B), 2.24 (1H, dtd, *J* 12.4, 7.1, 5.3, C(4)H^AH^B), 2.68 (1H, dd, *J* 16.7, 10.3, C(6)H^AH^B), 3.08-3.17 (1H, m, C(3)*H*), 3.33 (1H, dd, *J* 16.7, 4.2, C(6)H^AH^B), 3.84–3.90 (4H, m, C(5)H^AH^B and ArOCH_3), 4.06 (1H, td, *J* 8.0, 5.2, C(5)H^AH^B), 4.38 (1H, dd, *J* 14.7, 5.7, C(7)H^AH^B), 4.49 (1H, d, *J* 7.6, C(2)*H*), 4.57 (1H, dd, *J* 14.7, 6.4, C(7)H^AH^B), 6.89–6.93 (2H, m, ArC(3')*H*), 7.04 (1H, t, *J* 5.2, N*H*), 7.29–7.36 (5H, m, ArC*H*), 7.85– 7.88 (2H, m, ArC(2')*H*); $^{13}C\{^1H\}$ NMR (75 MHz, $CDCl_3$) δ_C: 31.5 (*C*(4)H_2), 37.6 (*C*(3)H), 38.1 (C(6)H_2), 42.9 (*C*(7)H_2), 55.6 (ArO*C*H_3), 68.2 (*C*(5)H_2), 80.6 (*C*(2)H), 113.8 (Ar*C*(3',5')H), 127.7 (Ar*C*(4")H), 127.9 (Ar*C*(3",5")H), 128.9 (Ar*C*(2",6")H), 129.9 (Ar*C*(1")), 130.5 (Ar*C*(2',6')H), 138.4 (Ar*C*(1')), 163.6 (Ar*C*(4')H), 171.1 (*C*ON), 197.4 (*C*OAr); *m/z* (ES^+) 354 ($[M+H]^+$, 100%); HRMS (ES^+) $C_{21}H_{24}O_4N$ $[M+H]^+$ found 354.1692, requires 354.1700 (−2.8 ppm).

(2*R*,3*R*)-*N*-Benzyl-3-(2-(4-chlorophenyl)-2-oxoethyl)tetrahydrofuran-2-carboxamide (34)

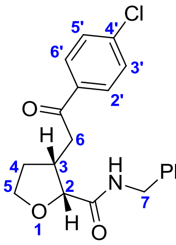

Enone-acid **S12** (100 mg, 0.37 mmol), $^{i}Pr_2NEt$ (97 μL, 0.57 mmol), $^{t}BuCOCl$ (92 μL, 0.74 mmol) in CH_2Cl_2 (3 mL) followed by (*S*)-(–)-tetramisole hydrochloride **5** (9 mg, 40 μmol) and $^{i}Pr_2NEt$ (162 μL, 0.93 mmol) were reacted according to general procedure 6 followed by addition of benzylamine (1.00

mL, 9.15mmol). The crude product (>99:1 $dr_{syn:anti}$) was purified by column chromatography (CH_2Cl_2 : EtOAc 90:10 to 70:30) to give the title compound **34** (97 mg, 73%) as a white solid. mp 139 °C; $[\alpha]_D^{20}$ −43.3 (*c* 0.5, CH_2Cl_2); Chiral HPLC analysis, Chiralpak IA (95:5 hexane : IPA, flow rate 1.0 $mLmin^{-1}$, 254 nm, 40 °C) t_R (2*R*,3*R*): 36.4 min, t_R (2*S*,3*S*): 41.3 min, 99% ee; ν_{max} (film) 3313 (N-H), 1683 (C=O, ketone), 1643 (C=O, amide), 1533 (C-N), 1085 (C-O, ether), 993 (C-Cl); ^1H NMR (400 MHz, $CDCl_3$) δ_H: 1.65 (1H, dq, *J* 13.2, 7.0, C(4)H^AH^B), 2.21–2.29 (1H, m, C(4)H^AH^B), 2.71 (1H, dd, *J* 17.2, 9.7, C(6)H^AH^B), 3.09–3.18 (1H, m, C(3)*H*), 3.34 (1H, dd, *J* 17.2, 4.6, C(6)H^AH^B), 3.88 (1H, q, *J* 7.9, C(5)H^AH^B), 4.06 (1H, td, *J* 7.9, 4.8, C(5)H^AH^B), 4.37 (1H, dd, *J* 14.6, 5.8, C(7)H^AH^B), 4.50 (1H, d, *J* 7.7, C(2)*H*), 4.56 (1H, dd, *J* 14.8, 6.4, C(7)H^AH^B), 7.05 (1H, s, N*H*), 7.33 (5H, m, Ar*H*), 7.40 (2H, d, *J* 8.5, ArC(3')*H*), 7.80 (2H, d, *J* 8.5, ArC(2')*H*); $^{13}C\{^1H\}$ NMR (100 MHz, $CDCl_3$) δ_C: 31.5 (*C*(4)H_2), 37.4 (*C*(3)H), 38.5 (*C*(6)H_2), 42.9 (*C*(7)H_2), 68.2 (*C*(5)H_2), 80.2 (*C*(2)H), 127.7 (Ar*C*(4")H), 127.9 (Ar*C*(3',5')H), 128.9 (Ar*C*(3",5")H), 129.0 (Ar*C*(2",6")H), 129.7 (Ar*C*(2',6')H), 135.1 (Ar*C*(4')H), 138.4 (Ar*C*(1")), 139.7 (Ar*C*(1')), 171.1 (*C*ON), 197.6 (*C*OAr); *m/z* (ES^+) 358 ($[M+H]^+$, 100%); HRMS (ES^+) $C_{20}H_{21}O_3NCl$ $[M+H]^+$ found 358.1198, requires 358.1204 (−1.8 ppm).

(2*R*,3*R*)-*N*-Benzyl-3-(2-oxopropyl)tetrahydrofuran-2-carboxamide (35)

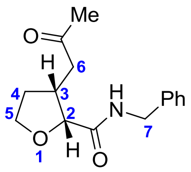

Enone-acid **40** (100 mg, 0.58 mmol), $^iPr_2NEt$ (152 μL, 0.87 mmol), ^tBuCOCl (143 μL, 1.16 mmol) in CH_2Cl_2 (3 mL) followed by (*S*)-(−)-tetramisole hydrochloride **5** (14 mg, 60 μmol) and $^iPr_2NEt$ (253 μL, 1.45 mmol) were reacted according to general procedure 6 followed by addition of benzylamine (1.00 mL, 9.15mmol). The crude product (73:27 $dr_{syn:anti}$) was purified by column chromatography (Petrol : EtOAc 90:10 to 70:30) to give the title compound **35** (76 mg, 50%) as a colourless oil. $[\alpha]_D^{20}$ +12.0 (*c* 0.5, CH_2Cl_2); Chiral HPLC analysis, Chiralpak IA (90:10 hexane : IPA, flow rate 1.0 $mLmin^{-1}$, 220 nm, 40 °C) t_R (2*R*,3*R*): 10.5 min, t_R (2*S*,3*S*): 12.4 min, 98% ee; ν_{max} (film) 3329 (N-H), 1713 (C=O, ketone), 1643 (C=O, amide), 1530 (C-N), 1078 (C-O, ether); ^1H NMR (300 MHz, $CDCl_3$) δ_H: 1.44–1.54 (1H, m, C(4)H^AH^B), 2.07–2.16 (4H, m, CH_3 and C(4)H^AH^B); 2.25 (1H, dd, *J* 17.6, 9.0, C(6)H^AH^B), 2.75 (1H, dd, *J* 17.6, 5.7, C(6)H^AH^B), 2.85–2.98 (1H, m, C(3)*H*), 3.75 (1H, td, *J* 8.5, 6.8, C(5)H^AH^B), 3.95 (1H, td, *J* 8.1, 4.1, C(5)H^AH^B), 4.31–4.45 (3H, m, C(7)H_2 and C(2)*H*), 6.89 (1H, s, N*H*), 7.19–7.30 (5H, m, Ar*H*); $^{13}C\{^1H\}$ NMR (125 MHz, $CDCl_3$) δ_C: 30.3 (*C*H_3), 31.5 (*C*(4)H_2), 37.0 (*C*(3)H), 43.0 (*C*(6)H_2), 43.4 (*C*(7)H_2), 68.2 (*C*(5)H_2), 79.8 (*C*(2)H), 127.7 (Ar*C*(4)H), 127.9 (Ar*C*(3,5)H), 128.9 (Ar*C*(2,6)H), 138.2 (Ar*C*(1)), 171.1 (*C*ON), 207.4 (*C*OCH_3); *m/z* (ES^+) 262 ($[M+H]^+$, 100%); HRMS (ES^+) $C_{15}H_{20}O_3N$ $[M+H]^+$ found 262.1439, requires 262.1438 (+0.5 ppm).

(2*R*,3*R*)-*N*-Benzyl-3-(3,3-dimethyl-2-oxobutyl)tetrahydrofuran-2-carboxamide (36)

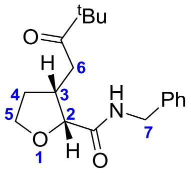

Enone-acid **S13** (100 mg, 0.47 mmol), $^{i}Pr_2NEt$ (122 μL, 0.70 mmol), $^{t}BuCOCl$ (115 μL, 0.93 mmol) in CH_2Cl_2 (3 mL) followed by (*S*)-(−)-tetramisole hydrochloride **5** (11 mg, 50 μmol) and $^{i}Pr_2NEt$ (203 μL, 1.17 mmol) were reacted according to general procedure 6 followed by addition of benzylamine (1.00 mL, 9.15mmol). The crude product (>99:1 $dr_{syn:anti}$) was purified by column chromatography (CH_2Cl_2 : EtOAc 90:10 to 70:30) to give the title compound **36** (77 mg, 69%) as a white solid. mp 64 °C; $[\alpha]_D^{20}$ +17.9 (*c* 0.5, CH_2Cl_2); Chiral HPLC analysis, Chiralpak AS-H (90:10 hexane : IPA, flow rate 1.0 mLmin^{-1}, 211 nm, 40 °C) t_R (2*S*,3*S*): 9.0 min, t_R (2*R*,3*R*): 13.2 min, 98% ee; ν_{max} (film) 3419 (N-H), 1695 (C=O, ketone), 1654 (C=O, amide), 1519 (C-N), 1089 (C-O, ether); ^{1}H NMR (500 MHz, $CDCl_3$) δ_H: 1.10 (9H, s, $(CH_3)_3$), 1.48 (1H, dq, *J* 12.5, 7.8, C(4)H^AH^B), 2.19 (1H, dtd, *J* 12.2, 7.1, 4.8, C(4)H^AH^B), 2.42 (1H, dd, *J* 18.0, 10.0, C(6)H^AH^B), 2.79 (1H, dd, J_{AB} 18.0, 4.5, C(6)H^AH^B), 2.94–3.01 (1H, m, C(3)*H*), 3.82 (1H, td, *J* 8.2, 7.1, C(5)H^AH^B), 4.00 (1H, td, *J* 8.0, 4.6, C(5)H^AH^B), 4.34 (1H, dd, *J* 14.7, 5.6, C(7)H^AH^B), 4.43 (1H, d, *J* 7.8, C(2)*H*), 4.59 (1H, dd, *J* 14.7, 6.5, C(7)H^AH^B), 6.96 (1H, s, N*H*), 7.28–7.30 (3H, m, Ar*H*), 7.32–7.35 (2H, m, Ar*H*); $^{13}C\{^1H\}$ NMR (75 MHz, $CDCl_3$) δ_C: 26.6 ($(CH_3)_3$), 31.7 ($C(4)H_2$), 36.5 ($C(6)H_2$), 37.0 (*C*(3)H), 42.9 ($C(7)H_2$), 44.2 ($C(CH_3)_3$), 68.0 ($C(5)H_2$), 80.2 (*C*(2)H), 127.6 (Ar*C*(4)H), 127.9 (Ar*C*(3,5)H), 128.8 (Ar*C*(2,6)H), 138.4 (Ar*C*(1)), 171.1 (*C*ON), 214.7 ($COC(H_3)_3$); *m/z* (ES$^+$) 304 ([M+H]$^+$, 100%); HRMS (ES$^+$) $C_{18}H_{26}O_3N$ [M+H]$^+$ found 304.1912, requires 304.1907 (+1.6 ppm).

(2*R*,3*R*)-Methyl 3-(2-oxo-2-phenylethyl)tetrahydrofuran-2-carboxylate (37)

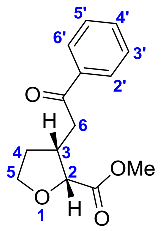

Enone-acid **S10** (100 mg, 0.43 mmol), $^{i}Pr_2NEt$ (116 μL, 0.64 mmol), $^{t}BuCOCl$ (105 μL, 0.85 mmol) in CH_2Cl_2 (3 mL) followed by (*S*)-(−)-tetramisole hydrochloride **5** (10 mg, 40 μmol) and $^{i}Pr_2NEt$ (186 μL, 1.07 mmol) were reacted according to general procedure 6. The reaction was concentrated *in vacuo* and diluted with MeOH (5 mL). The crude product (97:3 $dr_{syn:anti}$) was purified by column chromatography (Petrol : EtOAc 90:10 to 70:30) to give the title compound **37** (65 mg, 61%) as a light yellow oil. $[\alpha]_D^{20}$ −55.3 (*c* 0.3, CH_2Cl_2); Chiral HPLC analysis, Chiralpak IB (95:5 hexane : IPA, flow rate 1.0 mLmin^{-1}, 211 nm, 40 °C) t_R (2*S*,3*S*): 13.8 min, t_R (2*R*,3*R*): 15.9 min, >99% ee; ν_{max}

(film) 1743 (C=O, ester), 1683 (C=O, ketone), 1205 (C-O, ester), 1098 (C-O, ether); ^{1}H NMR (400 MHz, $CDCl_3$) δ_H: 1.78 (1H, dq, *J* 7.8, C(4)H^AH^B), 2.25 (1H, dtd, *J* 11.0, 7.1, 3.9, C(4)H^AH^B), 2.94–3.00 (1H, m, C(3)*H*), 3.08–3.19 (2H, m, C(6)H_2), 3.62 (3H, s, CO_2CH_3), 3.93 (1H, q, *J* 7.6, C(5)H^AH^B), 4.21 (1H, td, *J* 8.4, 3.9, C(5)H^AH^B), 4.65 (1H, d, *J* 7.4, C(2)*H*), 7.44–7.48 (2H, m, ArC(3',5')*H*), 7.57 (1H, tt, *J* 7.4, 1.3, ArC(4')*H*), 7.85–7.88 (2H, m, ArC(2',6')*H*); ^{13}C{^{1}H} NMR (100 MHz, $CDCl_3$) δ_C: 31.1 (*C*(4)H_2), 37.9 (*C*(3)H), 38.9 (*C*(6)H_2), 51.8 (CO_2CH_3), 68.5 (*C*(5)H_2), 79.3 (*C*(2)H), 128.0 (Ar*C*(3',5')H), 128.8 (Ar*C*(2',6')H), 133.4 (Ar*C*(4')H), 136.8 (Ar*C*(1')), 172.6 (*C*O_2CH_3), 198.8 (*C*OAr); *m/z* (ES^+) 249 ($[M+H]^+$, 100%); HRMS (ES^+) $C_{14}H_{17}O_4$ $[M+H]^+$ found 249.1122, requires 249.1121 (+0.3 ppm).

1-Phenyl-2-((2*R*,3*R*)-2-(pyrrolidine-1-carbonyl)tetrahydrofuran-3-yl)ethanone (38)

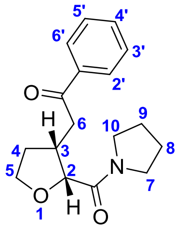

Enone-acid **S10** (100 mg, 0.43 mmol), ^{i}Pr$_2$NEt (112 μL, 0.64 mmol), ^{t}BuCOCl (105 μL, 0.85 mmol) in CH_2Cl_2 (3 mL) followed by (*S*)-(–)-tetramisole hydrochloride **5** (10 mg, 40 μmol) and ^{i}Pr$_2$NEt (185 μL, 1.07 mmol) were reacted according to general procedure 6 followed by addition of pyrrolidine (1.00 mL, 12.0 mmol). The crude product (>99:1 $dr_{syn:anti}$) was purified by column chromatography (CH_2Cl_2 : EtOAc 80:20 to 70:30) to give the title compound **38** (123 mg, 49%) as a white solid. mp 72–76 °C; $[\alpha]_D^{20}$ −67.5 (*c* 0.3, CH_2Cl_2); Chiral HPLC analysis, Chiralpak IA (90:10 hexane : IPA, flow rate 1.0 mLmin^{-1}, 211 nm, 40 °C) t_R (2*R*,3*R*): 17.3 min, t_R (2*S*,3*S*): 24.3 min, 99% ee; ν_{max} (film) 1682 (C=O, ketone), 1622 (C=O, amide), 1449 (C-N), 1051 (C-O, ether); ^{1}H NMR (300 MHz, $CDCl_3$) δ_H: 1.52-1.60 (1H, m, C(8)H^AH^B), 1.65–1.74 (1H, m, C(8)H^AH^B), 1.75–1.80 (1H, m, C(9)H^AH^B), 1.82–1.88 (1H, m, C(9)H^AH^B), 2.05 (1H, dq, *J* 11.9, 8.0, C(4)H^AH^B), 2.20 (1H, dtd, *J* 11.7, 7.6, 4.2, C(4)H^AH^B), 3.04–3.18 (3H, m, C(6)H^AH^B and C(3)*H* and C(7)H^AH^B), 3.35–3.51 (4H, m, C(6)H^AH^B and C(7)H^AH^B and C(10)H_2), 3.89 (1H, q, *J* 7.7, C(5)H^AH^B), 4.22 (1H, td, *J* 8.1, 4.4, C(5)H^AH^B), 4.79 (1H, d, *J* 7.2, C(2)*H*), 7.44 (2H, t, ArC(3',5')*H*), 7.55 (1H, t, *J* 7.4, ArC(4')*H*), 7.93 (2H, app. d, *J* 7.4, ArC(2',6')*H*); ^{13}C{^{1}H} NMR (75 MHz, $CDCl_3$) δ_C: 23.9 (*C*(8)H_2), 26.3 (*C*(9)H_2), 32.3 (*C*(4)H_2), 37.3 (*C*(3)H), 39.5 (*C*(6)H_2), 45.9 (*C*(7)H_2), 46.5 (*C*(10)H_2), 68.3 (*C*(5)H_2), 78.1 (*C*(2)H), 128.1 (Ar*C*(3',5')H), 128.7 (Ar*C*(2',6')H), 133.4 (Ar*C*(4')H), 136.7 (Ar*C*(1')), 169.5 (*C*ON), 199.3 (*C*OAr); *m/z* (ES^+) 575 ($[2M+H]^+$, 16%), 288 ($[M+H]^+$, 100%); HRMS (ES^+) $C_{17}H_{22}O_3N$ $[M+H]^+$ found 288.1597, requires 288.1594 (+1.0 ppm).

(2*R*,3*R*)-3-(2-Oxo-2-phenylethyl)tetrahydrofuran-2-carboxylic acid (39)

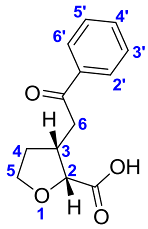

Enone-acid **S10** (100 mg, 0.43 mmol), $^{i}Pr_2NEt$ (116 μL, 0.64 mmol), $^{t}BuCOCl$ (105 μL, 0.85 mmol) in CH_2Cl_2 (3 mL) followed by (*S*)-(−)-tetramisole hydrochloride **5** (10 mg, 40 μmol) and $^{i}Pr_2NEt$ (186 μL, 1.07 mmol) were reacted according to general procedure 6. The reaction was concentrated *in vacuo*, diluted in EtOAc (3 mL) and 2 M NaOH (1.00 mL) was added. The crude product (>99:1 $dr_{syn:anti}$) was purified by column chromatography (CH_2Cl_2 : EtOAc 90:10 to 70:30) to give the title compound **39** (76 mg, 76%) as a white solid. mp 88–90 °C; $[\alpha]_D^{20}$ +3.1 (*c* 0.1, CH_2Cl_2); Chiral HPLC analysis, Chiralpak AS-H (90:10 hexane : IPA, flow rate 1.0 mLmin^{-1}, 211 nm, 40 °C) t_R (2*R*,3*R*): 6.3 min, t_R (2*S*,3*S*): 9.9 min, >99% ee; ν_{max} (film) 2922 (O-H, br.), 1732 (C=O, acid), 1687 (C=O, ketone), 1066 (C-O, ether), 1055 (C-O, ether); ^1H NMR (500 MHz, $CDCl_3$) δ_H: 1.74 (1H, dq, *J* 12.2, 8.8, C(4)H^AH^B), 2.29–2.35 (1H, m, C(4)H^AH^B), 3.02 (1H, dd, *J* 17.4, 8.3, C(6)H^AH^B), 3.16 (1H, dd, *J* 15.0, 7.3, C(3)*H*), 3.27 (1H, dd, *J* 17.4, 5.8, C(6)H^AH^B), 3.95 (1H, q., *J* 7.9, C(5)H^AH^B), 4.21 (1H, td, *J* 8.2, 3.6, C(5)H^AH^B), 4.67 (1H, d, *J* 7.9, C(2)*H*), 7.45 (2H, t, *J* 7.7, ArC(3',5')*H*), 7.56 (1H, t, *J* 7.4, ArC(4')*H*), 7.93 (2H, d, *J* 7.3, ArC(2',6')*H*); $^{13}C\{^1H\}$ NMR (75 MHz, $CDCl_3$) δ_C: 31.3 (*C*(4)H_2), 37.7 (*C*(3)H), 38.6 (*C*(6)H_2), 68.8 (*C*(5)H_2), 78.8 (*C*(2)H), 128.1 (Ar*C*(3',5')H), 128.8 (Ar*C*(2',6')H), 133.5 (Ar*C*(4')H), 136.6 (Ar*C*(1')), 175.2 (*C*O_2H), 198.2 (*C*OAr); *m/z* (ES^-) 467 ($[2M-H]^-$, 40%), 233 ($[M-H]^-$, 100%); HRMS (ES^-) $C_{13}H_{13}O_4$ $[M-H]^-$ found 233.0814, requires 233.0819 (−2.3 ppm).

Data for syn-3,4-Tetrahydrofurans

(3a*S*,7a*R*)-6-Phenyl-3,3a-dihydro-1*H*-furo[3,4-c]pyran-4(7a*H*)-one (43)

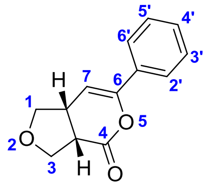

Enone-acid **S14** (100 mg, 0.43 mmol), $^{i}Pr_2NEt$ (112 μL, 0.64 mmol), $^{t}BuCOCl$ (79 μL, 0.64 mmol) in CH_2Cl_2 (2 mL) followed by (*S*)-(–)-tetramisole hydrochloride **5** (5 mg, 20 μmol) and $^{i}Pr_2NEt$ (185 μL, 1.07 mmol) were reacted according to general procedure 7. The crude product (>99:1 $dr_{syn:anti}$) was purified by column chromatography (petrol : EtOAc 90:10 to 70:30) to give the title compound **43** (61 mg, 66%) as a colourless oil. $[\alpha]_D^{20}$ +11.1 (*c* 1.4 in CH_2Cl_2); Chiral HPLC analysis, Chiralpak IA (95:5 hexane : IPA, flow rate 1.00 mLmin^{-1}, 211 nm, 40 °C) t_R (3a*R*,7a*S*): 18.9 min, t_R (3a*S*,7a*R*): 25.3 min, 98% ee; ν_{max} (film) 1757 (C=O), 1448 (C=C), 1211 (C-O), 1180 (C-O); ^{1}H NMR (300 MHz, $CDCl_3$) δ_H: 3.24–3.40 (2H, m, C(7a)*H* and C(3a)*H*), 3.71 (1H, dd, *J* 8.6, 5.4, C(1)H^AH^B), 4.13–4.30 (3H, m, C(1)H^AH^B and C(3)H_2), 5.67 (1H, d, *J* 4.2, C(7)*H*), 7.34–7.43 (3H, m, C(3',4',5')*H*), 7.59–7.67 (2H, m, C(2',6')*H*); $^{13}C\{^1H\}$ NMR (75 MHz, $CDCl_3$) δ_C: 37.5 (*C*(3a)), 42.0 (*C*(7a)), 71.0 (*C*(3)), 74.7 (*C*(1)), 99.4 (*C*(7)), 124.8 (*C*(3',5')), 128.6 (*C*(2',6')), 129.4 (*C*(4')), 132.1 (*C*(1')), 149.5 (*C*(6)), 168.3 (*C*(4)); *m/z* (CI^+) 217 ($[M+H]^+$, 63%), 145 ($[M–C_4H_6O]^+$, 100%); HRMS (CI^+) $C_{13}H_{13}O_3$ $[M+H]^+$ found 217.0866, requires 217.0865 (+0.6 ppm).

(3a*S*,7a*R*)-6-(4-Methoxyphenyl)-3,3a-dihydro-1*H*-furo[3,4-c]pyran-4(7a*H*)-one (44)

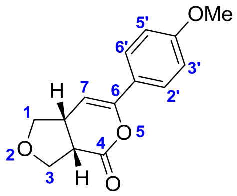

Enone-acid **S15** (100 mg, 0.38 mmol), $^{i}Pr_2NEt$ (99 μL, 0.57 mmol), $^{t}BuCOCl$ (93 μL, 0.76 mmol) in CH_2Cl_2 (2 mL) followed by (*S*)-(–)-tetramisole hydrochloride **5** (9.1 mg, 38 μmol) and $^{i}Pr_2NEt$ (160 μL, 0.95 mmol) were reacted according to general procedure 7. The crude product (>99:1 $dr_{syn:anti}$) was purified by column chromatography (neat hexane to 70:30 hexane : EtOAc, R_f 0.27) to give the title compound **44** (62 mg, 67%) as a white solid. mp 88–90 °C; $[\alpha]_D^{20}$ +14.1 (*c* 1.28 in $CHCl_3$); Chiral HPLC analysis, Chiralcel OD-H (90:10 hexane : IPA, flow rate 1.50 mLmin^{-1}, 211 nm, 40 °C) t_R (3a*S*,7a*R*): 11.3 min, t_R (3a*R*,7a*S*): 14.0 min, 99% ee; ν_{max} (film) 1751, 1672, 1607, 1510, 1464, 1348, 1285, 1250, 1213, 1175; ^{1}H NMR (300 MHz, $CDCl_3$) δ_H: 3.21–3.36 (2H, m, C(3a)*H* and C(7a)*H*), 3.68 (1H, dd, *J* 8.5, 5.4, C(1)H^AH^B), 3.82 (3H, s, OCH_3), 4.10–4.28 (3H, m, C(1)H^AH^B and C(3)H_2),

5.52 (1H, d, *J* 4.1, C(7)*H*), 6.89 (2H, d, *J* 8.9, C(3',5')*H*), 7.55 (2H, d, *J* 8.9, C(2',6')*H*); $^{13}C\{^1H\}$ NMR (75 MHz, $CDCl_3$) δ_C: 37.5 (*C*(3a)), 42.0 (*C*(7a)), 55.5 (O*C*H_3), 71.0 (*C*(3)H_2), 74.8 (*C*(1)H_2), 97.4 (*C*(7)H), 114.0 (Ar*C*(3',5')H), 124.7 (Ar*C*(1')), 126.3 (Ar*C*(2',6')H), 149.4 (*C*(6)), 160.5 (*C*(4')), 168.4 (*C*(4)); HRMS (NSI^+) $C_{21}H_{23}O_4NNa$ $[M+BnNH_2+Na]^+$ found 376.1510, requires 376.1525 (–2.5 ppm).

(3a*S*,7a*R*)-6-(4-Chlorophenyl)-3,3a-dihydro-1*H*-furo[3,4-c]pyran-4(7a*H*)-one (45)

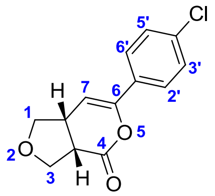

Enone-acid **S16** (100 mg, 0.37 mmol), $^iPr_2NEt$ (97 μL, 0.56 mmol), ^tBuCOCl (69 μL, 0.56 mmol) in CH_2Cl_2 (2 mL) followed by (*S*)-(–)-tetramisole hydrochloride **5** (5 mg, 20 μmol) and $^iPr_2NEt$ (162 μL, 0.93 mmol) were reacted according to general procedure 7. The crude product (>99:1 $dr_{syn:anti}$) was purified by column chromatography (CH_2Cl_2 neat to CH_2Cl_2 : Et_2O 90:10) to give the title compound **45** (48 mg, 52%) as a light yellow oil $[\alpha]_D^{20}$ +14.3 (*c* 1.0 in CH_2Cl_2); Chiral HPLC analysis, Chiralpak IA (95:5 hexane : IPA, flow rate 1.50 mLmin^{-1}, 211 nm, 40 °C) t_R (3a*R*,7a*S*): 13.7min, t_R (3a*S*,7a*R*): 16.5min, 94% ee; ν_{max} (film) 1757 (C=O), 1490 (C=C), 1213 (C-O), 1180 (C-O), 1092 (C-Cl); ^1H NMR (400 MHz, $CDCl_3$) δ_H: 3.25–3.38 (2H, m, C(7a)*H* and C(3a)*H*), 3.71 (1H, dd, *J* 8.6, 5.4, C(1)H^AH^B), 4.12–4.28 (3H, m, C(1)H^AH^B and C(3)H_2), 5.65 (1H, d, *J* 4.2, C(7)*H*), 7.35 (2H, d, *J* 8.7, C(2',6')*H*), 7.55 (2H, d, *J* 8.7, ArC(3',5')*H*); $^{13}C\{^1H\}$ NMR (100 MHz, $CDCl_3$) δ_C: 37.6 (*C*(3a)H), 41.9 (*C*(7a)H), 71.1 (*C*(3)H_2), 74.6 (*C*(1)H_2), 99.8 (*C*(7)H), 126.1 (Ar*C*(2',6')H), 128.9 (Ar*C*(3',5')H), 130.6 (Ar*C*(4')), 135.4 (Ar*C*(1')), 148.7 (*C*(6)), 168.0 (*C*(4)); HRMS (NSI^+) $C_{20}H_{21}O_3N^{35}Cl$ $[M+BnNH_2+H]^+$ found 358.1208, requires 358.1204 (+1.0 ppm).

(3*S*,4*R*)-*N*-Benzyl-4-(2-oxo-2-phenylethyl)tetrahydrofuran-3-carboxamide (46)

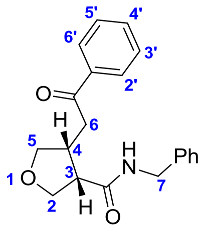

Enone-acid **S14** (100 mg, 0.43 mmol), $^iPr_2NEt$ (112 μL, 0.64 mmol), ^tBuCOCl (79 μL, 0.64 mmol) in CH_2Cl_2 (2 mL) followed by (*S*)-(–)-tetramisole hydrochloride **5** (5 mg, 30 μmol) and $^iPr_2NEt$ (185 μL, 1.07mmol) were reacted according to general procedure 7 followed by addition of benzylamine (1.00 mL, 9.15mmol). The crude product (>99:1 $dr_{syn:anti}$) was purified by column chromatography (90:10

CH_2Cl_2 : Et_2O to 70:30 CH_2Cl_2 : Et_2O) to give the title compound **46** (95 mg, 69%) as a white solid. mp 90–91 °C; $[\alpha]_D^{20}$ −11.8 (*c* 0.5 in CH_2Cl_2); Chiral HPLC analysis, Chiralpak IA (90:10 hexane : IPA, flow rate 1.5mLmin^{-1}, 254 nm, 40 °C) t_R (3*S*,4*R*): 14.0min, t_R (3*R*,4*S*): 20.6min, 96% ee; ν_{max} (film) 3238 (N-H), 1680 (C=O, ketone), 1643 (C=O, amide), 1051 (C-O); ^{1}H NMR (400 MHz, $CDCl_3$) δ_H: 2.97–3.11 (2H, m, C(4)*H* and C(6)H^AH^B), 3.14–3.20 (1H, m, C(3)*H*), 3.20–3.30 (1H, m, C(6)H^AH^B), 3.60 (1H, t, *J* 8.5, C(5)H^AH^B), 4.02 (1H, dd, *J* 9.0, 6.6, C(2)H^AH^B), 4.13 (1H, dd, *J* 8.3, 7.4, C(5)H^AH^B), 4.18 (1H, dd, *J* 9.0, 4.0, C(2)H^AH^B), 4.25 (1H, dd, *J* 14.5, 5.7, C(7)H^AH^B), 4.34 (1H, dd, *J* 14.5, 5.7, C(7)H^AH^B), 6.16 (1H, t, *J* 5.4, N*H*), 7.11–7.20 (5H, m, C(7)Ph*H*), 7.42–7.48 (2H, m, C(3',5')*H*), 7.58 (1H, tt, *J* 7.4, 1.5, C(4')*H*), 7.83–7.87 (2H, m, C(2',6')*H*); ^{13}C{^{1}H} NMR (100 MHz, $CDCl_3$) δ_C: 37.7 (*C*(6)H_2), 38.3 (*C*(4)H), 43.7 (*C*(7)H_2), 48.4 (*C*(3)H), 70.9 (*C*(2)H_2), 72.7 (*C*(5)H_2), 127.6 (Ar*C*H), 128.0 (Ar*C*H (×2)), 128.2 (Ar*C*H), 128.8 (Ar*C*(3')H), 133.5 (Ar*C*(4')H), 136.5 (Ar*C*(1')), 138.1 (Ar*C*), 172.3 (*C*ON), 199.3 (*C*OAr); *m/z* (NSI$^+$) 324 ([M+H]$^+$, 100%); HRMS (NSI$^+$) $C_{20}H_{22}O_3N$ [M+H]$^+$ found 324.1596, requires 324.1594 (+0.6 ppm).

(3*S*,4*R*)-*N*-Benzyl-4-(2-(4-methoxyphenyl)-2-oxoethyl)tetrahydrofuran-3-carboxamide (47)

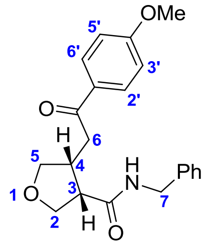

Enone-acid **S15** (106 mg, 0.40 mmol), ^{i}Pr$_2$NEt (100 µL, 0.60 mmol), ^{t}BuCOCl (100 µL, 0.80 mmol) in CH_2Cl_2 (2 mL) followed by (*S*)-(–)-tetramisole hydrochloride **5** (9.6 mg, 40 µmol) and ^{i}Pr$_2$NEt (170 µL, 1.00 mmol) were reacted according to general procedure 7 followed by addition of benzylamine (220 µL, 2.0 mmol) and DMAP (cat). The crude product (>99:1 $dr_{syn:anti}$) was purified by column chromatography (neat hexane to 40:60 hexane : EtOAc, R_f 0.20) to give the title compound **47** (91 mg, 64%) as a white solid. mp 100–101 °C; $[\alpha]_D^{20}$ −8.4 (*c* 1.02 in $CHCl_3$); Chiral HPLC analysis, Chiralcel OJ-H (90:10 hexane : IPA, flow rate 1.50 mLmin^{-1}, 211 nm, 40 °C) t_R (3*S*,4*R*): 22.5 min, t_R (3*R*,4*S*): 28.2 min, 97% ee; ν_{max} (film) 3331, 1668, 1639, 1597, 1545, 1508, 1454, 1263, 1236, 1167; ^{1}H NMR (300 MHz, $CDCl_3$) δ_H: 2.91–3.06 (2H, m, C(4)*H* and C(6)H^AH^B), 3.09–3.20 (2H, m, C(3)*H* and C(6)H^AH^B), 3.57 (1H, t, *J* 8.4, C(5)H^AH^B), 3.86 (3H, s, OCH_3), 3.98 (1H, dd, *J* 8.9, 6.7, C(2)H^AH^B), 4.07 (1H, dd, *J* 8.4, 7.3, C(5)H^AH^B), 4.14 (1H, dd, *J* 8.9, 4.1, C(2)H^AH^B), 4.22 (1H, dd, *J* 14.6, 5.8, C(7)H^AH^B), 4.30 (1H, dd, *J* 14.6, 5.8, C(7)H^AH^B), 6.38 (1H, t, *J* 5.7, N*H*), 6.89 (2H, d, *J* 8.9 C(3',5')*H*), 7.09–7.21 (5H, m, C(7)Ph*H*), 7.81 (2H, d, *J* 8.9, C(2',6')*H*); ^{13}C{^{1}H} NMR (75 MHz, $CDCl_3$) δ_C: 37.1 (*C*(6)H_2), 38.3 (*C*(4)H), 43.6 (*C*(7)H_2), 48.2 (*C*(3)H), 55.6 (O*C*H_3), 70.7 (*C*(2)H_2), 72.6 (*C*(5)H_2), 113.8 (Ar*C*(3',5')H), 127.4 (Ar*C*H), 127.9 (Ar*C*H), 128.6 (Ar*C*H), 129.6 (Ar*C*H),

130.4 (Ar*C*H), 138.1 (Ar*C*), 163.7 (Ar*C*(4')), 172.3 (*C*ON), 197.7 (*C*OAr); HRMS (NSI^+) $C_{21}H_{23}O_4NNa$ $[M+Na]^+$ found 376.1510, requires 376.1525 (–2.5 ppm).

(3*S*,4*R*)-*N*-Benzyl-4-(2-(4-chlorophenyl)-2-oxoethyl)tetrahydrofuran-3-carboxamide (48)

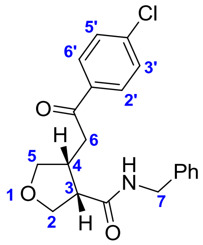

Enone-acid **S16** (100 mg, 0.37 mmol), $^iPr_2NEt$ (97 μL, 0.56 mmol), ^tBuCOCl (69 μL, 0.56 mmol) in CH_2Cl_2 (2 mL) followed by(*S*)-(–)-tetramisole hydrochloride **5** (5 mg, 20 μmol) and $^iPr_2NEt$ (162 μL, 0.93 mmol) were reacted according to general procedure 7. followed by addition of benzylamine (1.0 mL, 9.15 mmol) and DMAP (cat.). The crude product (>99:1 $dr_{syn:anti}$) was purified by column chromatography (90:10 CH_2Cl_2 : Et_2O to 70:30 CH_2Cl_2 : Et_2O) to give the title compound **48** (89 mg, 67%) as a white solid. mp 100–102 °C; $[\alpha]_D^{20}$ –1.2 (*c* 0.6 in CH_2Cl_2); Chiral HPLC analysis, Chiralpak IA (90:10 hexane : IPA, flow rate 1 $mLmin^{-1}$, 254 nm, 40 °C) t_R (3*S*,4*R*): 18.1min, t_R (3*R*,4*S*): 25.0min, 98% ee; ν_{max} (film) 3302 (N-H), 1684 (C=O, ketone), 1645 (C=O, amide), 1092 (C-O); ^1H NMR (500 MHz, $CDCl_3$) δ_H: 2.93 (1H, dd, *J* 18.0, 6.2, C(6)H^AH^B), 2.97–3.05 (1H, m, C(4)*H*), 3.11–3.21 (2H, m, C(3)*H* and C(6)H^AH^B), 3.56 (1H, t, *J* 8.3, C(5)H^AH^B), 3.98 (1H, dd, *J* 8.8, 6.6, C(2)H^AH^B), 4.08 (1H, t, *J* 7.9, C(5)H^AH^B), 4.13 (1H, dd, *J* 9.0, 4.1, C(2)H^AH^B), 4.27 (2H, d, *J* 5.9, C(7)CH_2), 6.51 (1H, br. s, N*H*), 7.10–7.20 (5H, m, C(7)Ph*H*), 7.38 (2H, d, *J* 8.5, ArC(3',5')*H*), 7.73 (2H, d, *J* 8.5, ArC(2',6')*H*); $^{13}C\{^1H\}$ NMR (125 MHz, $CDCl_3$) δ_C: 37.6 (*C*(6)), 38.0 (*C*(4)), 43.6 (*C*(7)), 48.1 (*C*(3)), 70.7 (*C*(2)), 72.6 (*C*(5)), 127.5 (Ar*C*H), 127.9 (Ar*C*H), 128.7 (Ar*C*H), 129.0 (Ar*C*H), 129.5 (Ar*C*H), 134.8 (Ar*C*(4')), 138.1 (Ar*C*), 139.8 (Ar*C*(1')), 172.2 (*C*ON), 197.9 (*C*OAr); HRMS (NSI^+) $C_{20}H_{21}O_3N^{35}Cl$ $[M+H]^+$ found 358.1208, requires 358.1204 (+1.0 ppm).

Data for anti-3,4-Tetrahydrofurans

(3a*R*,7a*R*)-6-Phenyl-3,3a-dihydro-1*H*-furo[3,4-c]pyran-4(7a*H*)-one (49)[7]

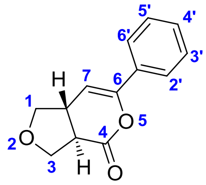

Enone-acid **S14** (100 mg, 0.43 mmol), OTMS-quinidine **19** (34 mg, 80 μmol), Mukaiyama derivative (215 mg, 0.64 mmol), and $^{i}Pr_2NEt$ (185 μL, 1.07 mmol) in CH_2Cl_2 (3 mL) were reacted according to general procedure 8. The crude product (16:84 $dr_{syn:anti}$) was purified by column chromatography (90:10 petrol : EtOAc) to give the title compound **49** (>1:99 $dr_{syn:anti}$, 53 mg, 58%) as a colourless oil. $[\alpha]_D^{20}$ +16.6 (*c* 0.2 in CH_2Cl_2); Chiral HPLC analysis, Chiralpak IA (95:5 hexane : IPA, flow rate 1.70 mLmin^{-1}, 211 nm, 40 °C) t_R (3a*S*,7a*S*): 23.4 min, t_R (3a*R*,7a*R*): 27.0 min, >99% ee; ν_{max} (film) 1759 (C=O, ester), 1448 (C=C), 1152(C-O), 1080 (C-O); ^1H NMR (500 MHz, $CDCl_3$) δ_H: 2.94 (1H, ddd, *J* 14.8, 10.4, 7.6, C(3a)*H*), 3.16–3.23 (1H, m, C(7a)*H*), 3.65 (1H, dd, *J* 11.0, 7.5, C(1)H^AH^B), 3.95 (1H, dd, *J* 10.3, 8.3, C(3)H^AH^B), 4.21 (1H, t, *J* 7.9, (3)H^AH^B), 4.26 (1H, *J* 7.4, C(1)H^AH^B), 6.08 (1H, d, *J* 2.3, C(7)*H*), 7.35–7.43 (3H, m, ArC(3',5')*H* and ArC(4')*H*), 7.60–7.64 (2H, m, ArC(2',6')*H*); $^{13}C\{^1H\}$ NMR (125 MHz, $CDCl_3$) δ_C: 39.5 (*C*(7a)H), 46.6 (*C*(3a)H), 65.4 (*C*(3)H_2), 70.0 (*C*(1)H_2), 101.5 (*C*(7)H), 124.8 (Ar*C*(2',6')H), 128.8 (Ar*C*(3',5')H), 129.6 (Ar*C*(4')H), 131.9 (Ar*C*(1')H), 153.8 (*C*(6)), 167.6 (CO_2),

*Data for minor syn-(3aR,7aS)-diastereoisomer ent-**43**:* Chiral HPLC analysis, Chiralpak IA (95:5 hexane : IPA, flow rate 1.70 mLmin^{-1}, 211 nm, 40 °C) t_R (3a*R*,7a*S*): 18.5 min, t_R (3a*S*,7a*R*): 25.0 min, 78% ee;

HRMS (ES$^+$) $C_{20}H_{22}NO_3$ [M+BnNH$_2$+H]$^+$ found 324.1590, requires 324.1600 (–1.3 ppm).

(3a*R*,7a*R*)-6-(4-Methoxyphenyl)-3,3a-dihydro-1*H*-furo[3,4-c]pyran-4(7a*H*)-one (50)

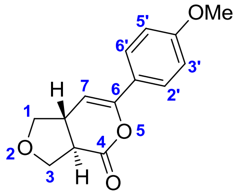

Enone-acid **S15** (102 mg, 0.39 mmol), OTMS-quinidine **19** (30.6 mg, 77 μmol), Mukaiyama derivative **8** (212 mg, 0.58 mmol), and $^{i}Pr_2NEt$ (170 μL, 0.96 mmol) in CH_2Cl_2 (2 mL) were reacted according to general procedure 8. The crude product (15:85 $dr_{syn:anti}$) was purified by column chromatography (neat hexane to 70:30 hexane : EtOAc, R_f 0.26) to give the title compound (15:85

dr$_{syn:anti}$,55 mg, 58%) as a white solid. mp 129–132 °C; $[\alpha]_D^{20}$ +26.1 (*c* 0.60 in $CHCl_3$); ν_{max} (film) 1769, 1609, 1512, 1256, 1179, 1152;

Data for major anti-(3aR,7aR)-diastereoisomer ***50****:* Chiral HPLC analysis, Chiralcel OD-H (90:10 hexane : IPA, flow rate 1.50 mLmin^{-1}, 211 nm, 40 °C) t_R (3a*R*,7a*R*): 13.1 min, t_R (3a*S*,7a*S*): 31.5 min, >99% ee; ^{1}H NMR (300 MHz, $CDCl_3$) δ_H: 2.87–2.95 (1H, m, C(3a)*H*), 3.12–3.21 (1H, m, C(7a)*H*), 3.63 (1H, dd, *J* 11.0, 7.5, C(1)H^AH^B), 3.83 (3H, s, OCH_3), 3.94 (1H, dd, *J* 10.4, 8.3, C(3)H^AH^B), 4.18–4.28 (2H, m, C(1)H^AH^B and C(3)H^AH^B), 5.93 (1H, d, *J* 2.3, C(7)*H*), 6.90 (2H, d, *J* 8.9, C(3',5')*H*), 7.54 (2H, d, *J* 8.9, C(2',6')*H*); ^{13}C{^{1}H} NMR (75 MHz, $CDCl_3$) δ_C: 39.6 (*C*(7a)H), 46.7 (*C*(3a)H), 55.5 (O*C*H_3), 65.4 (*C*(3)H_2), 70.1 (*C*(1)H_2), 99.6 (*C*(7)H), 114.1 (Ar*C*(3',5')H), 124.5 (Ar*C*(1')H), 126.3 (Ar*C*(2',6')H), 153.6 (*C*(6)), 160.7 (Ar*C*(4')), 167.8 (*C*(4));

*Data for minor syn-(3aR,7aS)-diastereoisomer ent-****44****:* Chiral HPLC analysis, Chiralcel OD-H (90:10 hexane : IPA, flow rate 1.50 mLmin^{-1}, 211 nm, 40 °C) t_R (3a*S*,7a*R*): 11.4 min, t_R (3a*R*,7a*S*): 14.2 min, 77% ee; ^{1}H NMR (300 MHz, $CDCl_3$) δ_H: 3.24–3.36 (2H, m, C(3a)*H* and C(7a)*H*), 3.69 (1H, dd, *J* 8.6, 5.5, C(1)H^AH^B), 3.83 (3H, s, OCH_3), 4.13 (1H, dd, *J* 8.6, 6.6, C(1)H^AH^B), 4.18–4.28 (2H, m, C(3)H_2), 5.52 (1H, d, *J* 4.1, C(7)*H*), 6.87–6.93 (2H, m, C(3',5')*H*), 7.52–7.58 (2H, m, C(2',6')*H*); ^{13}C{^{1}H} NMR (75 MHz, $CDCl_3$) (*selected*) δ_C: 37.6 (*C*(3a)H), 42.1 (*C*(7a)H), 71.0 (*C*(3)H_2), 74.8 (*C*(1)H_2), 97.4 (*C*(7)H), 168.4 (*C*(4));

HRMS (ES$^+$) $C_{21}H_{23}NNaO_4$ [M+BnNH_2+Na]$^+$ found 376.1508, requires 376.1525 (–3.0 ppm).

(3a*R*,7a*R*)-6-(4-Chlorophenyl)-3,3a-dihydro-1*H*-furo[3,4-c]pyran-4(7a*H*)-one (51)

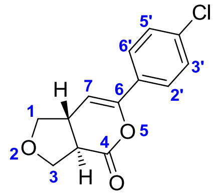

Enone-acid **S16** (107 mg, 0.40 mmol), OTMS-quinidine **19** (31.7 mg, 80 μmol), Mukaiyama derivative **8** (202 mg, 0.60 mmol), and ^{i}Pr$_2$NEt (170 μL, 1.00 mmol) in CH_2Cl_2 (2 mL) were reacted according to general procedure 8. The crude product (18:82 dr$_{syn:anti}$) was purified by column chromatography (neat hexane to 70:30 hexane : EtOAc, R_f 0.33) to give the title compound (18:82 dr$_{syn:anti}$, 58 mg, 58%) as a pale orange solid. mp 119–122 °C; $[\alpha]_D^{20}$ +18.9 (*c* 0.44 in $CHCl_3$); ν_{max} (film) 1767, 1686, 1639, 1589, 1491, 1402, 1381, 1271, 1153;

Data for major anti-(3aR,7aR)-diastereoisomer ***51****:* Chiral HPLC analysis, Chiralcel OD-H (90:10 hexane : IPA, flow rate 1.50 mLmin^{-1}, 211 nm, 40 °C) t_R (3a*R*,7a*R*): 11.1 min, t_R (3a*S*,7a*S*): 26.0 min, >99% ee; ^{1}H NMR (300 MHz, $CDCl_3$) δ_H: 2.87–2.98 (1H, m, C(3a)*H*), 3.12–3.24 (1H, m, C(7a)*H*),

3.65 (1H, dd, *J* 10.9, 7.5, C(1)H^AH^B), 3.95 (1H, dd, *J* 10.2, 8.4, C(3)H^AH^B), 4.18–4.30 (2H, m, C(1)H^AH^B and C(3)H^AH^B), 6.07 (1H, d, *J* 2.3, C(7)*H*), 7.36 (2H, d, *J* 8.8, C(2',6')*H*), 7.51–7.58 (2H, m, C(3',5')*H*); $^{13}C\{^1H\}$ NMR (125 MHz, $CDCl_3$) δ_C: 39.5 (*C*(7a)H), 46.4 (*C*(3a)H), 65.3 (*C*(3)H_2), 69.8 (*C*(1)H_2), 102.0 (*C*(7)H), 126.1 (Ar*C*(3',5')H), 129.0 (Ar*C*(2',6')H), 130.3 (Ar*C*(4')), 135.4 (Ar*C*(1')), 152.7 (*C*(6)), 167.2 (*C*(4));

*Data for minor syn-(3aR,7aS)-diastereoisomer ent-**45**:* Chiral HPLC analysis, Chiralcel OD-H (90:10 hexane : IPA, flow rate 1.50 mLmin^{-1}, 211 nm, 40 °C) t_R (3a*S*,7a*R*): 8.9 min, t_R (3a*R*,7a*S*): 9.6 min, 75% ee; ^1H NMR (300 MHz, $CDCl_3$) δ_H: 3.26–3.39 (2H, m, C(3a)*H* and C(7a)*H*), 3.71 (1H, dd, *J* 8.6, 5.4, C(1)H^AH^B), 4.14 (1H, dd, *J* 8.6, 6.6, C(1)H^AH^B), 4.18–4.30 (2H, m, C(3)H_2), 5.65 (1H, d, *J* 4.1, C(7)*H*), 7.32–7.39 (2H, m, C(2',6')*H*), 7.51–7.58 (2H, m, C(3',5')*H*); $^{13}C\{^1H\}$ NMR (125 MHz, $CDCl_3$) (*selected*) δ_C: 37.6 (*C*(3a)H), 41.9 (*C*(7a)H), 71.0 (*C*(3)H_2), 74.6 (*C*(1)H_2), 99.8 (*C*(7)H), 126.1 (Ar*C*(2'6')H), 128.9 (Ar*C*(3',5')H);

HRMS (ES^+) $C_{20}H_{20}{}^{35}ClNNaO_3$ [M+$BnNH_2$+Na]$^+$ found 380.1018, requires 380.1029 (–1.6 ppm).

(3*R*,4*R*)-*N*-Benzyl-4-(2-oxo-2-phenylethyl)tetrahydrofuran-3-carboxamide (52)

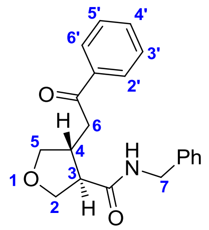

Enone-acid **S14** (89 mg, 0.38 mmol), OTMS-quinidine **19** (30.1 mg, 76 μmol), Mukaiyama derivative **8** (192 mg, 0.57 mmol), and $^iPr_2NEt$ (170 μL, 0.95 mmol) in CH_2Cl_2 (2 mL) were reacted according to general procedure 8 followed by addition of benzylamine (80 μL, 0.76 mmol). The crude product (20:80 $dr_{syn:anti}$) was purified by column chromatography (neat hexane to 50:50 hexane : EtOAc, R_f 0.20) to give the title compound (20:80 $dr_{syn:anti}$,69 mg, 56%) as a white solid. mp 102–104 °C; $[\alpha]_D^{20}$ +34.9 (*c* 0.51 in $CHCl_3$); ν_{max} (film) 3302, 1678, 1639, 1531, 1447, 1244, 1221;

*Data for major anti-(3R,4R)-diastereoisomer **52**:* Chiral HPLC analysis, Chiralpak IB (92.5:7.5 hexane : IPA, flow rate 1.50 mLmin^{-1}, 211 nm, 40 °C) t_R (3*R*,4*R*): 29.7 min, t_R (3*S*,4*S*): 32.5 min, >99% ee; ^1H NMR (300 MHz, $CDCl_3$) δ_H: 2.62–2.68 (1H, m, C(3)*H*), 2.86–2.99 (1H, m, C(4)*H*), 3.08 (1H, dd, *J* 18.1, 5.2, C(6)H^AH^B), 3.27 (1H, dd, *J* 18.1, 9.3, C(6)H^AH^B), 3.51 (1H, dd, *J* 8.9, 4.0, C(5)H^AH^B), 3.90 (1H, dd, *J* 8.9, 7.8, C(2)H^AH^B), 3.98 (1H, dd, *J* 8.9, 6.7, C(5)H^AH^B), 4.16 (1H, dd, *J* 9.0, 5.7, C(2)H^AH^B), 4.44 (2H, d, *J* 5.9, C(7)H_2), 7.04–7.30 (6H, m, C(7)Ph*H* and N*H*), 7.35–7.44 (2H, m, C(3',5')*H*), 7.47–7.57 (1H, m, C(4')*H*), 7.85–7.90 (2H, m, C(2',6')*H*); $^{13}C\{^1H\}$ NMR (75

MHz, $CDCl_3$) δ_C: 40.5 (*C*(4)H), 43.2 (*C*(6)H_2), 43.7 (*C*(7)H_2), 50.8 (*C*(3)H), 69.6 (*C*(2)H_2), 73.2 (*C*(5)H_2), 127.4 (Ar*C*H), 127.7 (Ar*C*H), 128.1 (Ar*C*H), 128.8 (Ar*C*H), 128.9 (Ar*C*H), 133.8 (Ar*C*H), 136.5 (Ar*C*), 138.6 (Ar*C*), 172.8 (*C*ON), 199.9 (*C*OAr);

*Data for minor syn-(3R,4S)-diastereoisomer ent-**46**:* Chiral HPLC analysis, Chiralpak IB (92.5:7.5 hexane : IPA, flow rate 1.50 mLmin^{-1}, 211 nm, 40 °C) t_R (3*R*,4*S*): 17.3 min, t_R (3*S*,4*R*): 19.5 min, 81% ee; ^{1}H NMR (300 MHz, $CDCl_3$) δ_H: 2.86–3.19 (4H, m, C(3)*H*, C(4)*H* and C(6)*H*$_2$), 3.57–3.65 (1H, m, C(5)H^AH^B), 3.91–4.05 (2H, m, C(2)H^AH^B and C(5)H^AH^B), 4.10 (1H, dd, *J* 9.1, 4.1, C(2)H^AH^B), 4.14–4.29 (2H, m, C(7)*H*$_2$), 6.18 (1H, t, *J* 5.4, N*H*), 7.04–7.30 (5H, m, C(7)Ph*H*), 7.35–7.44 (2H, m, C(3',5')*H*), 7.47–7.57 (1H, m, C(4')*H*), 7.75–7.80 (2H, m, C(2',6')*H*);^{13}C{^{1}H} NMR (75 MHz, $CDCl_3$) (*selected*) δ_C: 37.6 (*C*(6)H_2), 38.2 (*C*(4)H), 43.6 (*C*(7)H_2), 48.3 (*C*(3)H), 70.8 (*C*(2)H_2), 72.7 (*C*(5)H_2), 127.5 (Ar*C*H), 128.0 (Ar*C*H), 133.5 (Ar*C*H), 138.1 (Ar*C*), 172.2 (*C*ON), 199.2 (*C*OAr);

HRMS (ES^+) $C_{20}H_{22}NO_3$ $[M+H]^+$ found 324.1590, requires 324.1600 (–1.3 ppm).

(3*R*,4*R*)-*N*-Benzyl-4-(2-(4-methoxyphenyl)-2-oxoethyl)tetrahydrofuran-3-carboxamide (53)

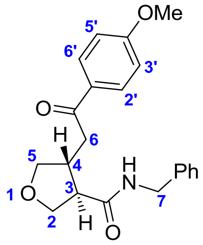

Enone-acid **S15** (106 mg, 0.40 mmol), OTMS-quinidine **19** (31.7 mg, 80 μmol), Mukaiyama derivative **8** (202 mg, 0.60 mmol), and ^{i}Pr$_2$NEt (170 μL, 1.00 mmol) in CH_2Cl_2 (2 mL) were reacted according to general procedure 8 followed by addition of benzylamine (220 μL, 2.00 mmol) and DMAP (cat.). The crude product (10:90 dr$_{syn:anti}$) was purified by column chromatography (neat hexane to 40:60 hexane : EtOAc, R_f 0.24) to give the title compound (10:90 dr$_{syn:anti}$,65 mg, 47%) as a white solid. mp 117–119 °C; $[\alpha]_D^{20}$ +37.0 (*c* 0.50 in $CHCl_3$); ν_{max} (film) 3298, 1668, 1632, 1597, 1528, 1510, 1248, 1228, 1167;

*Data for major anti-(3R,4R)-diastereoisomer **53**:* Chiral HPLC analysis, Chiralcel OJ-H (90:10 hexane : IPA, flow rate 1.50 mLmin^{-1}, 211 nm, 40 °C) t_R (3*S*,4*S*): 53.6 min, t_R (3*R*,4*R*): 57.1 min, >99% ee; ^{1}H NMR (300 MHz, $CDCl_3$) δ_H: 2.61–2.67 (1H, m, C(3)*H*), 2.82–2.94 (1H, m, C(4)*H*), 3.02 (1H, dd, *J* 17.8, 5.0, C(6)H^AH^B), 3.20 (1H, dd, *J* 17.8, 9.5, C(6)H^AH^B), 3.49 (1H, dd, *J* 8.9, 4.0, C(5)H^AH^B), 3.79 (3H, s, OC*H*$_3$), 3.87 (1H, dd, *J* 8.8, 7.9, C(2)H^AH^B), 3.95 (1H, dd, *J* 8.9, 6.7, C(5)H^AH^B), 4.16 (1H, dd, *J* 9.0, 5.7, C(2)H^AH^B), 4.42 (2H, d, *J* 5.8, C(7)*H*$_2$), 6.85 (2H, d, *J* 8.9,

C(3',5')*H*), 7.15–7.36 (6H, m, C(7)Ph*H* and N*H*), 7.84 (2H, d, *J* 8.9, C(2',6')*H*); ^{13}C{^{1}H} NMR (75 MHz, $CDCl_3$) δ_C: 40.6 (*C*(4)H), 42.9 (*C*(6)H_2), 43.7 (*C*(7)H_2), 50.8 (*C*(3)H), 55.6 (O*C*H_3), 69.5 (*C*(2)H_2), 73.1 (*C*(5)H_2), 114.0 (Ar*C*(3',5')H), 127.4 (Ar*C*H), 127.7 (Ar*C*H), 128.7 (Ar*C*H), 129.6 (Ar*C*), 130.5 (Ar*C*H), 138.7 (Ar*C*), 164.0 (Ar*C*(4')), 172.9 (*C*ON), 198.4 (*C*OAr);

*Data for minor syn-(3R,4S)-diastereoisomer ent-**47**:* Chiral HPLC analysis, Chiralcel OJ-H (90:10 hexane : IPA, flow rate 1.50 mLmin^{-1}, 211 nm, 40 °C) t_R (3*S*,4*R*): 23.0 min, t_R (3*R*,4*S*): 28.0 min, 80% ee; ^{1}H NMR (300 MHz, $CDCl_3$) δ_H: 2.82–3.11 (4H, m, C(3)*H*, C(4)*H* and C(6)*H*$_2$), 3.50–3.56 (1H, m, C(5)H^AH^B), 3.80 (3H, s, OC*H*$_3$), 3.95–4.05 (2H, m, C(2)H^AH^B and C(5)H^AH^B), 4.09 (1H, dd, *J* 8.9, 4.0, C(2)H^AH^B), 4.17–4.28 (2H, m, C(7)*H*$_2$), 6.21 (1H, t, *J* 5.6, N*H*), 6.81–6.88 (2H, m, C(3',5')*H*), 7.04–7.12 (5H, m, C(7)Ph*H*), 7.75 (2H, d, *J* 8.9, C(2',6')*H*); ^{13}C{^{1}H} NMR (75 MHz, $CDCl_3$) (*selected*) δ_C: 37.1 (*C*(6)H_2), 38.3 (*C*(4)H), 48.3 (*C*(3)H), 70.8 (*C*(2)H_2), 72.7 (*C*(5)H_2), 113.8 (Ar*C*(3',5')H), 127.5 (Ar*C*H), 128.0 (Ar*C*H), 129.7 (Ar*C*H), 130.4 (Ar*C*H), 138.2 (Ar*C*), 163.8 (Ar*C*(4')), 172.3 (*C*ON), 197.7 (*C*OAr);

HRMS (ES$^+$) $C_{21}H_{23}NNaO_4$ [M+Na]$^+$ found 376.1508, requires 376.1525 (–3.0 ppm).

(3*R*,4*R*)-*N*-Benzyl-4-(2-(4-chlorophenyl)-2-oxoethyl)tetrahydrofuran-3-carboxamide (54)

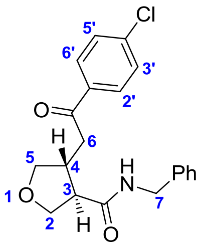

Enone-acid **S16** (107 mg, 0.40 mmol), OTMS-quinidine **19** (31.7 mg, 80 μmol), Mukaiyama derivative **8** (202 mg, 0.60 mmol), and ^{i}Pr$_2$NEt (170 μL, 1.00 mmol) in CH_2Cl_2 (2 mL) were reacted according to general procedure 8 followed by addition of benzylamine (220 μL, 2.00 mmol) and DMAP (cat.). The crude product (20:80 dr$_{syn:anti}$) was purified by trituration from Et2O and hexane to give two crops of the title compound, 1st crop (>1:99 dr$_{syn:anti}$, 35 mg, 24%) 2nd crop (15:75 dr$_{syn:anti}$, 52 mg, 36%), as a beige solid.

*Data for major anti-(3R,4R)-diastereoisomer **54**:* mp 143–144 °C; $[\alpha]_D^{20}$ +38.0 (*c* 0.75 in $CHCl_3$); ν_{max} (film) 3300, 1676, 1637, 1589, 1528, 1398, 1246, 1219; Chiral HPLC analysis, Chiralpak AD-H (95:5 hexane : IPA, flow rate 1.25 mLmin^{-1}, 211 nm, 40 °C) t_R (3*R*,4*R*): 55.3 min, t_R (3*S*,4*S*): 59.7 min, >99% ee; ^{1}H NMR (300 MHz, $CDCl_3$) δ_H: 2.67–2.73 (1H, m, C(3)*H*), 2.92–3.02 (1H, m, C(4)*H*), 3.12 (1H, dd, *J* 18.1, 5.2, C(6)H^AH^B), 3.31 (1H, dd, *J* 18.1, 9.2, C(6)H^AH^B), 3.58 (1H, dd, *J* 9.0, 4.1, C(5)H^AH^B), 3.98 (1H, dd, *J* 8.9, 7.9, C(2)H^AH^B), 4.05 (1H, dd, *J* 8.9, 6.6, C(5)H^AH^B), 4.22 (1H, dd, *J*

9.0, 5.8, C(2)H^AH^B), 4.51 (2H, d, *J* 5.8, C(7)H_2), 7.06 (1H, t, *J* 4.8, N*H*), 7.25–7.36 (5H, m, C(7)Ph*H*), 7.44 (2H, d, *J* 8.7, C(2',6')*H*), 7.88 (2H, d, *J* 8.7, C(3',5')*H*); $^{13}C\{^1H\}$ NMR (75 MHz, $CDCl_3$) δ_C: 40.5 (*C*(4)H), 43.3 (*C*(6)H_2), 43.8 (*C*(7)H_2), 50.9 (*C*(3)H), 69.7 (*C*(2)H_2), 73.2 (*C*(5)H_2), 127.5 (Ar*C*H), 127.8 (Ar*C*H), 128.8 (Ar*C*H), 129.2 (Ar*C*H), 129.6 (Ar*C*H), 134.8 (Ar*C*), 138.6 (Ar*C*), 140.4 (Ar*C*), 172.7 (*C*ON), 198.7 (*C*OAr);

*Data for minor syn-(3R,4S)-diastereoisomer ent-**48**:* Chiral HPLC analysis, Chiralpak AD-H (95:5 hexane : IPA, flow rate 1.25 $mLmin^{-1}$, 211 nm, 40 °C) t_R (3*S*,4*R*): 28.1 min, t_R (3*R*,4*S*): 38.4 min, 76% ee.

HRMS (ES^+) $C_{20}H_{20}{}^{35}ClNNaO_3$ $[M+Na]^+$ found 380.1018, requires 380.1029 (–1.6 ppm).

Conformation of Stereochemistry

The relative and absolute configuration of the *syn*-2,3-dihydrobenzofuran products has previously been confirmed through X-ray crystallographic analysis.[6] The relative and absolute configuration of the *anti*-2,3-dihydrobenzofuran products was confirmed through epimerisation (see below). The relative and absolute configuration of the *syn*-2,3-THF, *syn*-3,4-THF and *anti*-3,4-THF products was assigned by analogy.

Epimerisation of 20

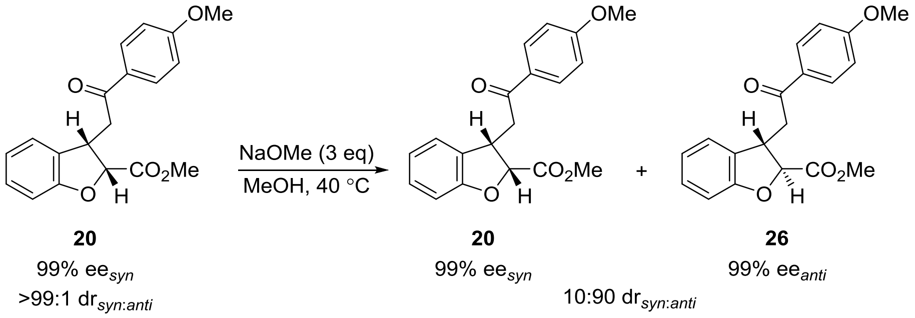

syn-2,3-Dihydrobenzofuran **20** (>99:1 $dr_{syn:anti}$, 46 mg, 0.14 mmol) was suspended in MeOH (0.7 mL) under an atmosphere of N_2. Freshly prepared NaOMe (0.5 M in MeOH, 0.85 mL, 0.42 mmol) was added and the reaction was stirred at 40 °C for 16 h before being quenched with 1 M HCl (5 mL). The solution was diluted with EtOAc (20 mL), the layers separated and the organics washed with 1 M HCl (10 mL) and brine (2 × 10 mL) before being dried over $MgSO_4$, filtered and concentrated. The crude product was purified by column chromatography (hexane : EtOAc 80:20, R_f 0.20) to give *anti*-2,3-dihydropyranone **26** (10:90 $dr_{syn:anti}$, 23 mg, 49%) as a colourless oil with data in accordance with the compound obtained from cyclisation using OTMS-quinidine **19** as the catalyst. Chiral HPLC analysis, Chiralpak AD-H (85:15 hexane : IPA, flow rate 0.5 mLmin^{-1}, 254 nm).

HPLC traces overleaf.

syn-2,3-Dihydrobenzofuran **20** (>99:1 dr$_{syn:anti}$, >99% ee$_{syn}$) from reaction with (*S*)-(–)-tetramisole hydrochloride **5**.

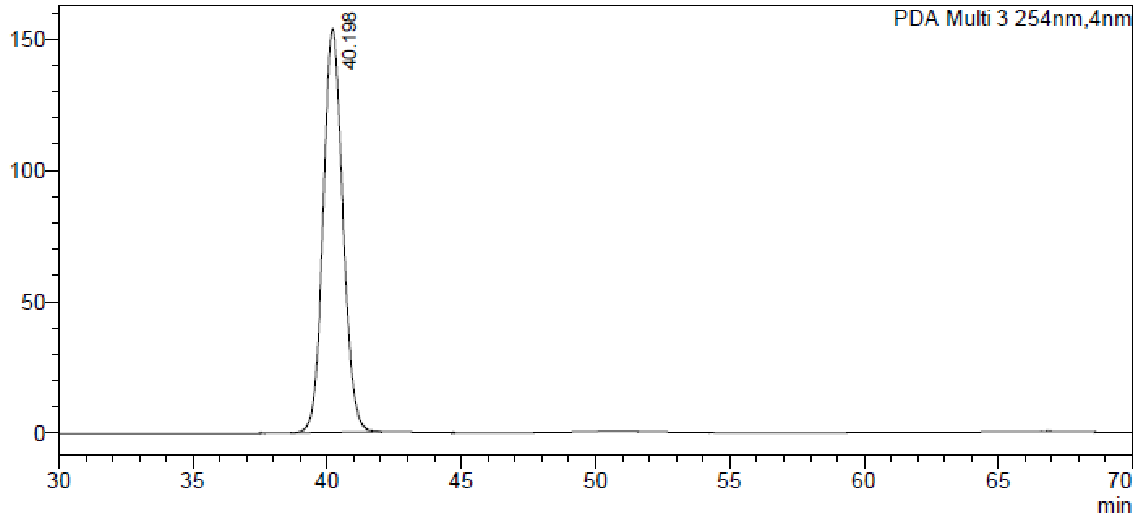

anti-2,3-Dihydrobenzofuran **26** (10:90 dr$_{syn:anti}$, >99% ee$_{anti}$) from epimerisation of **20**.

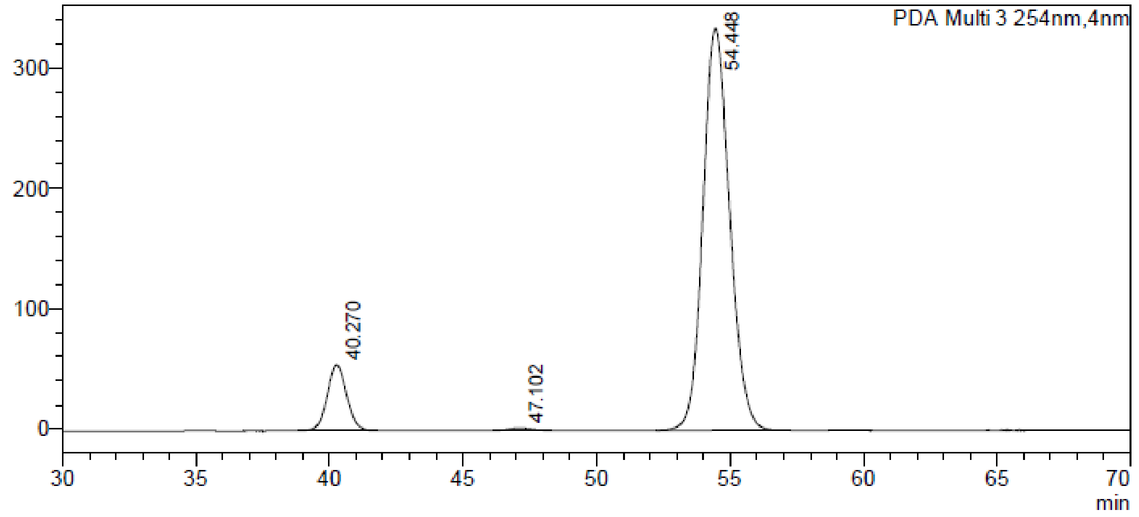

anti-2,3-Dihydrobenzofuran **26** (12:88 dr$_{syn:anti}$, 97% ee$_{anti}$, 60% ee$_{syn}$) from reaction with OTMS-quinidine **19**.

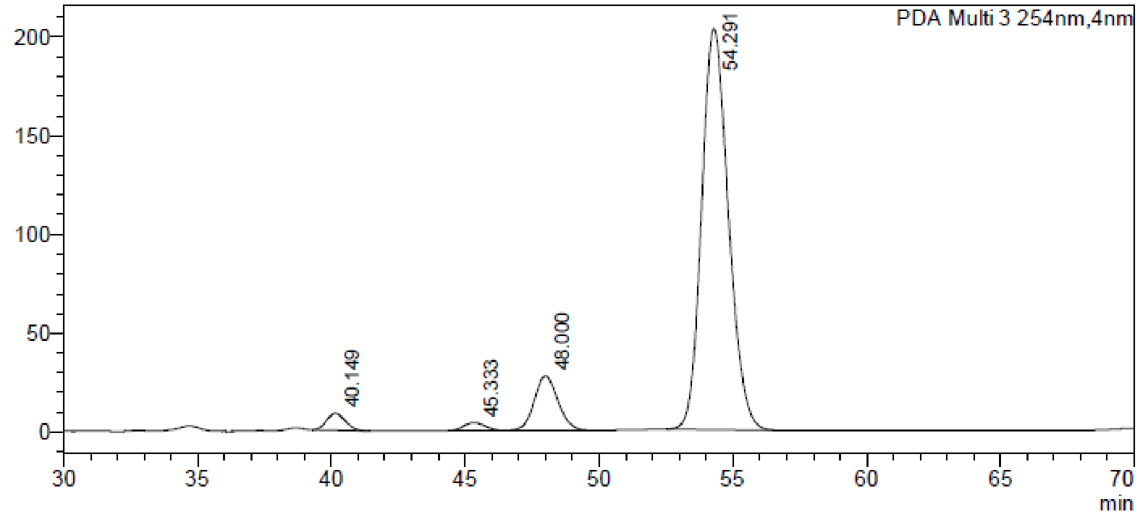

References

[1] I. Busygin, V. Nieminen, A. Taskinen, J. Sinkkonen, E. Toukoniitty, R. Sillanpää, D. Y. Murzin, R. Leino, *J. Org. Chem*. **2008**, *73*, 6559-6569.

[2] D. Belmessieri, D. B. Cordes, A. M. Z. Slawin, A. D. Smith, *Org. Lett.* **2013**, *15*, 3472-3475.

[3] M. B. Gravestock, D. W. Knight, J. S. Lovell, S. R. Thornton, *J. Chem. Soc., Perkin Trans. 1* **1999**, 3143-3155.

[4] B. Simonot, G. Rousseau, *Synth. Commun.* **1993**, *23*, 549-560.

[5] S. A. Ahmad-Junan, A. J. Walkington, D. A. Whiting, *J. Chem. Soc., Perkin Trans. 1* **1992**, 2313-2320.

[6] D. Belmessieri, L. C. Morrill, C. Simal, A. M. Z. Slawin, A. D. Smith, *J. Am. Chem. Soc.* **2011**, *133*, 2714-2720.

[7] An authentic scalemic mixture of both **49** and *ent*-**43** was prepared using a 50:50 mixture of OTMS-quinine **16** and pseudoenantiomeric OTMS-quinidine **19**.

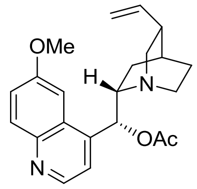

14

^1H, $CDCl_3$, 300 MHz

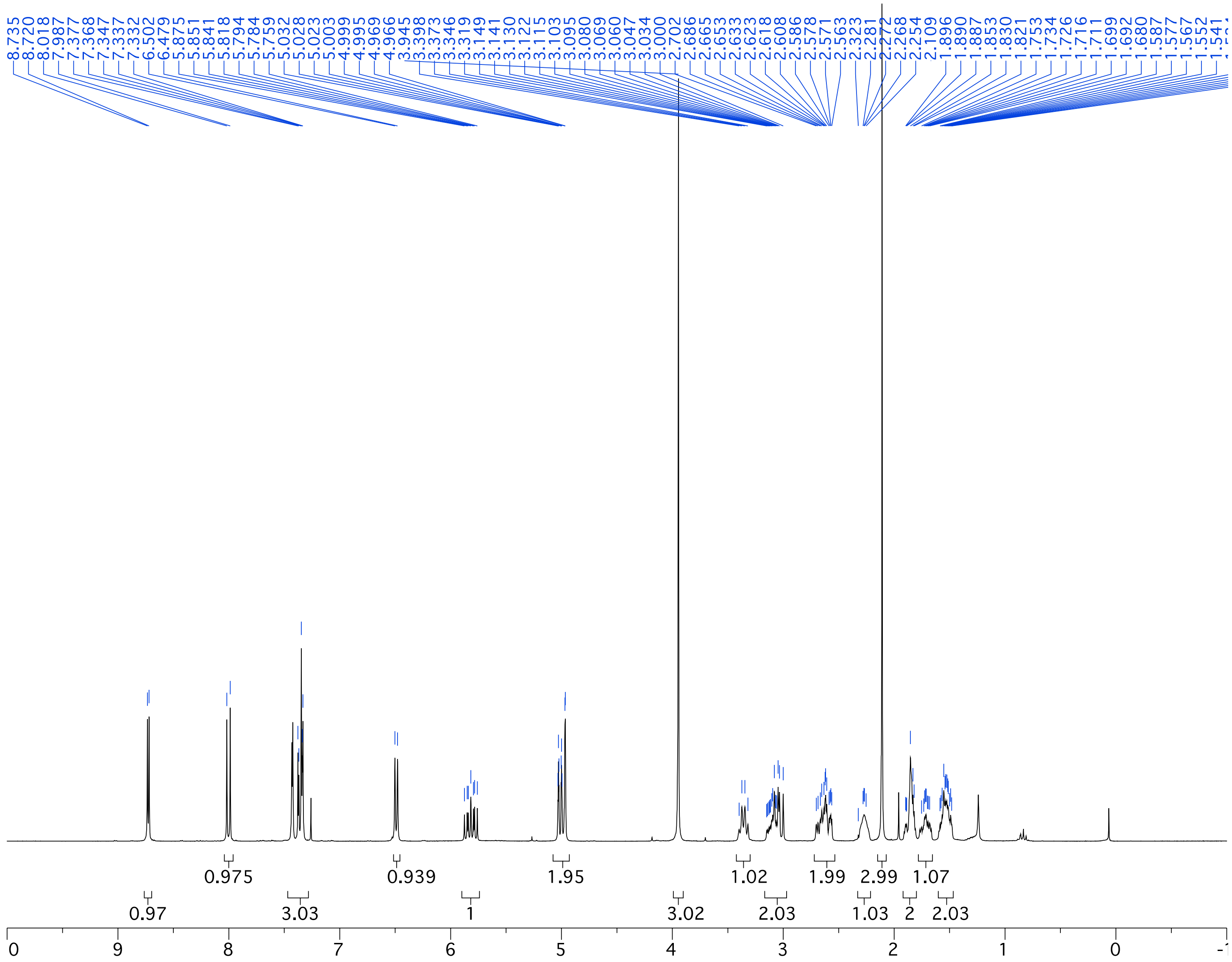

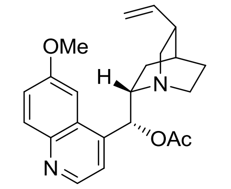

14

$^{13}C\{^{1}H\}$, $CDCl_3$, 75 MHz

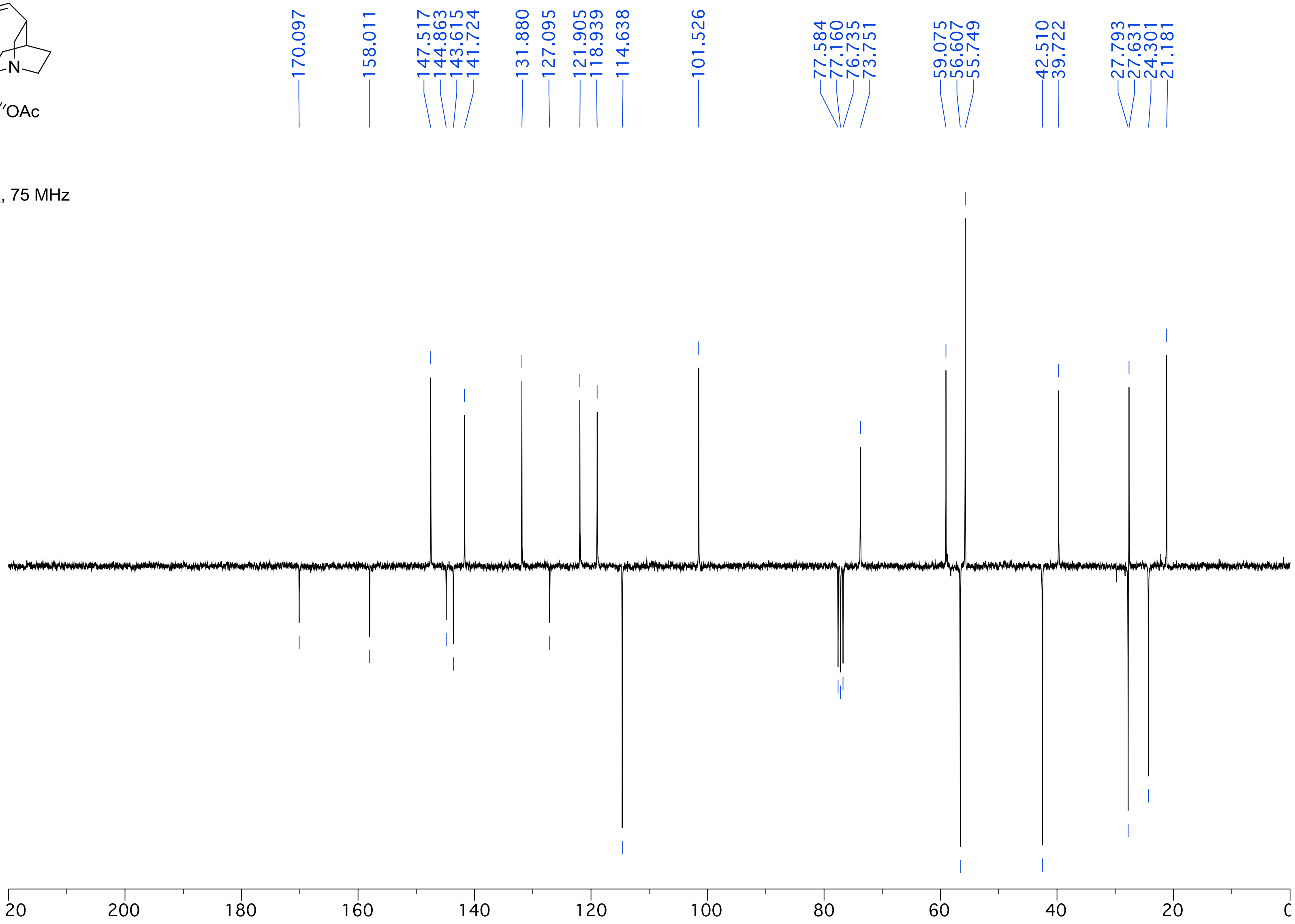

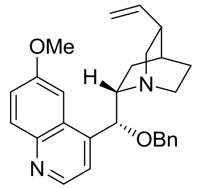

15

^{1}H, $CDCl_3$, 300 MHz

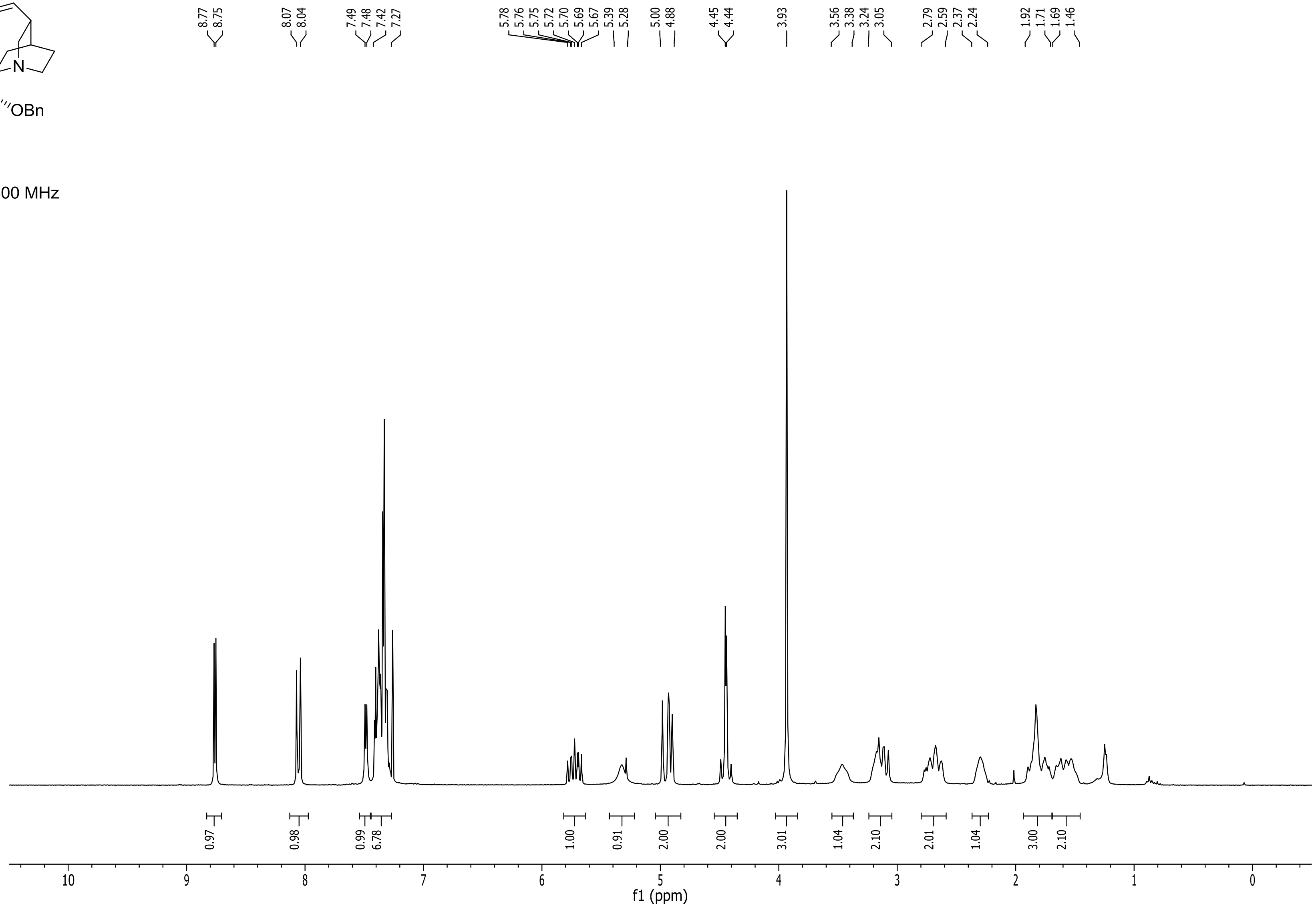

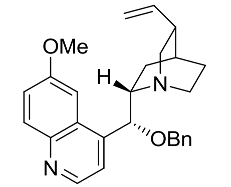

15

$^{13}C\{^{1}H\}$, $CDCl_3$, 75 MHz

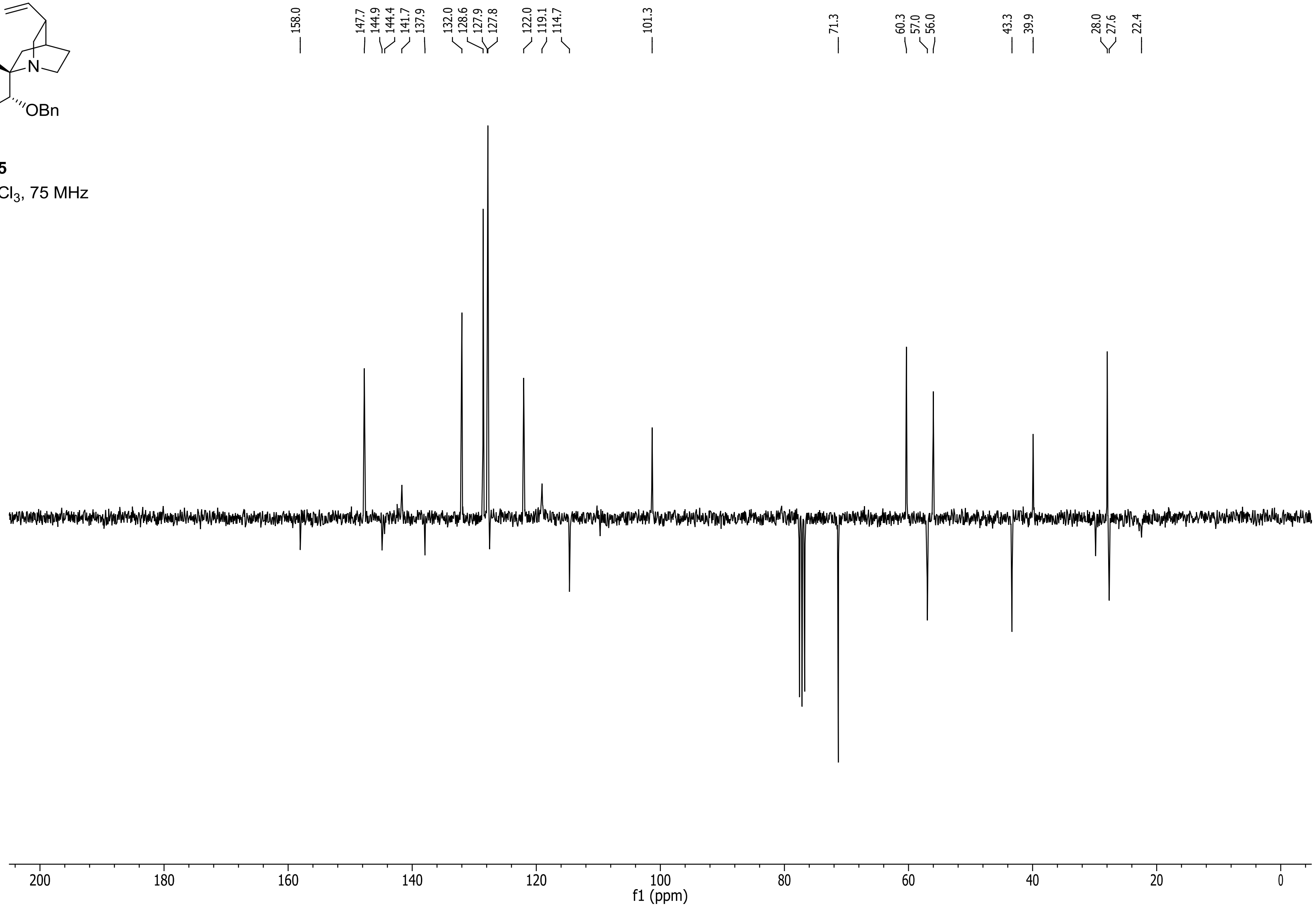

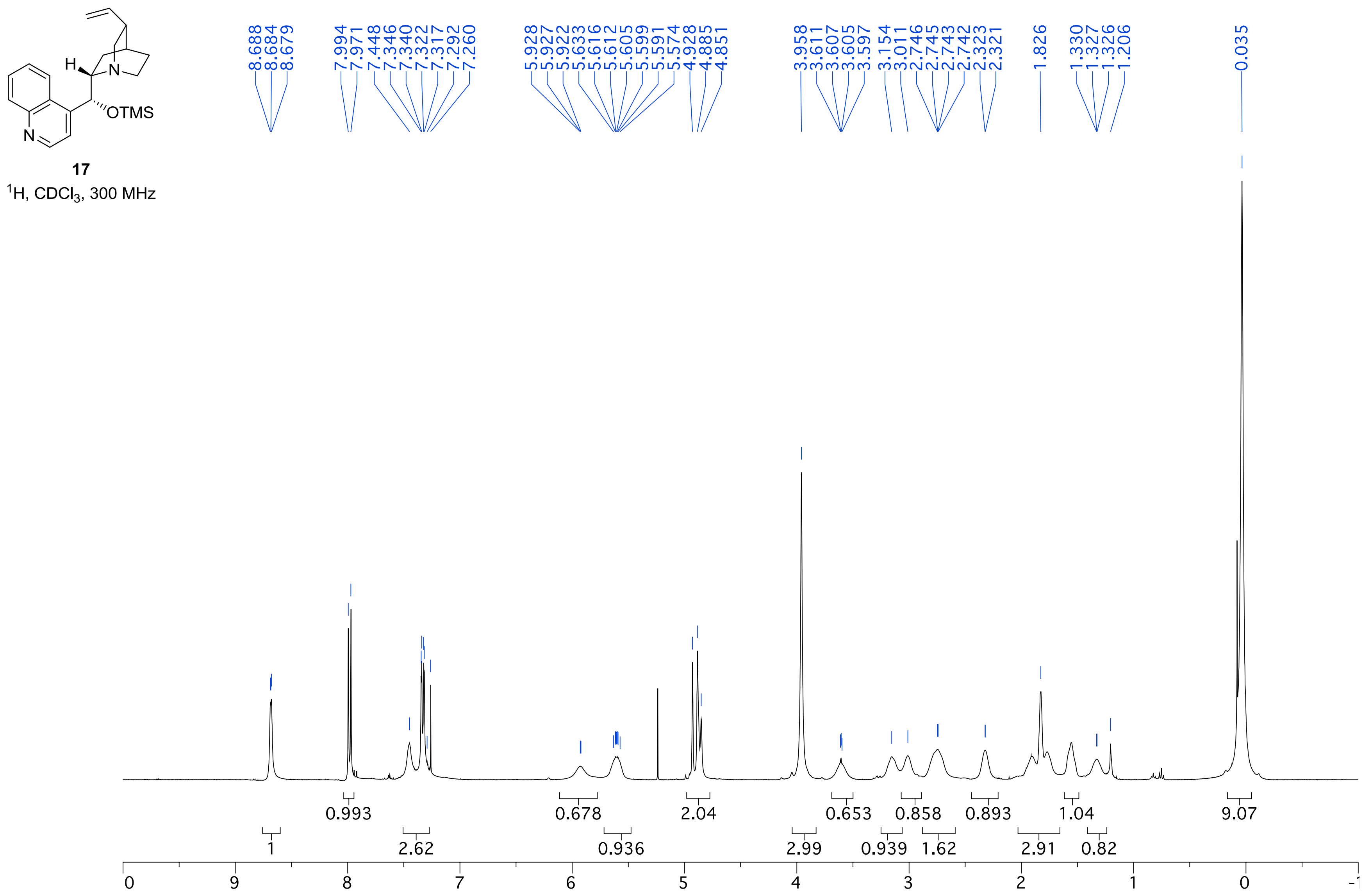

17
^{1}H, $CDCl_3$, 300 MHz

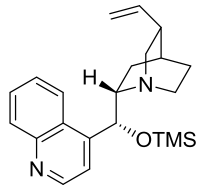

17

$^{13}C\{^{1}H\}$, $CDCl_3$, 100 MHz

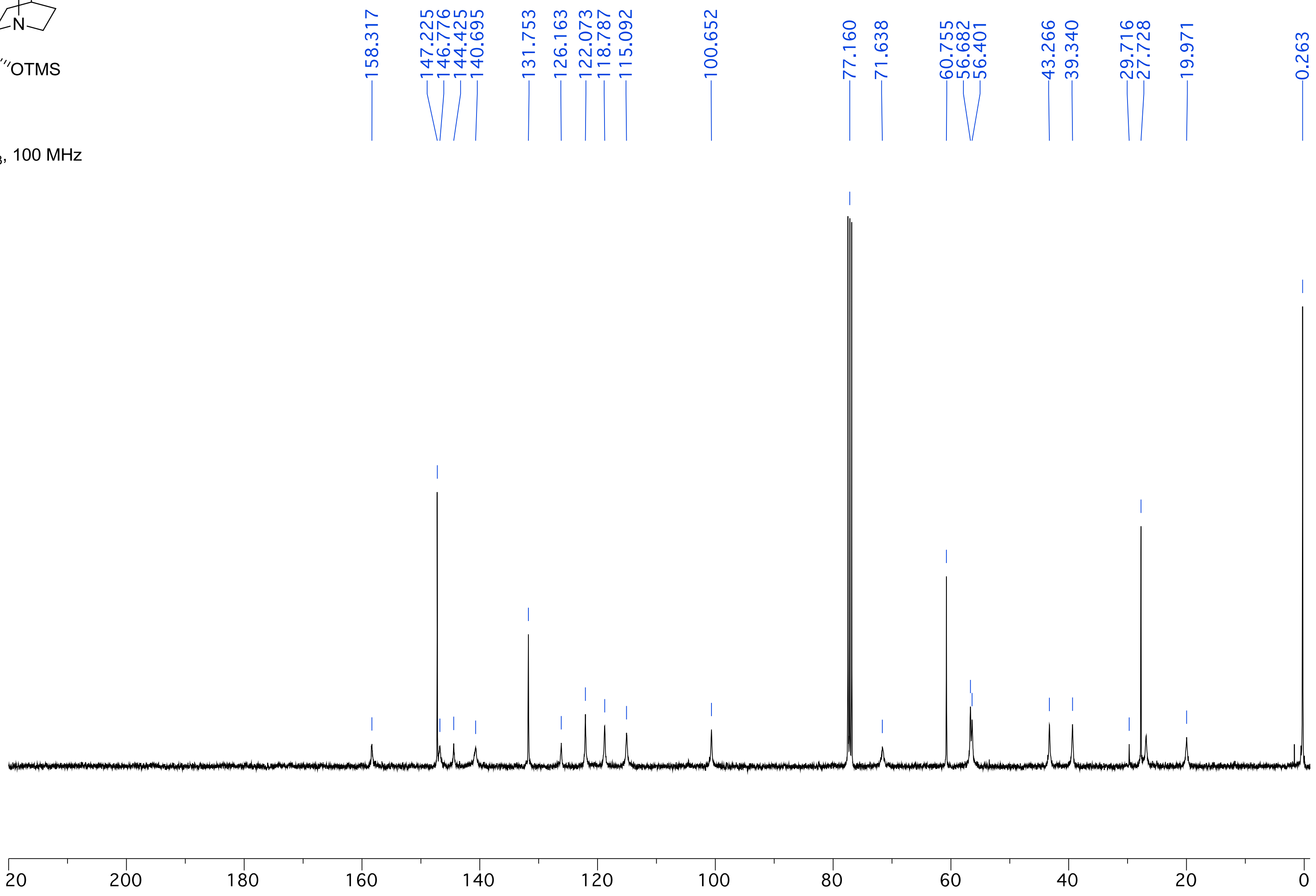

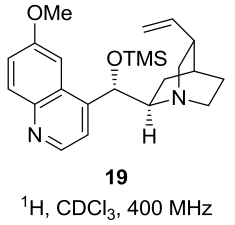
OMe
OTMS
N
N
H
19
1H, CDCl3, 400 MHz

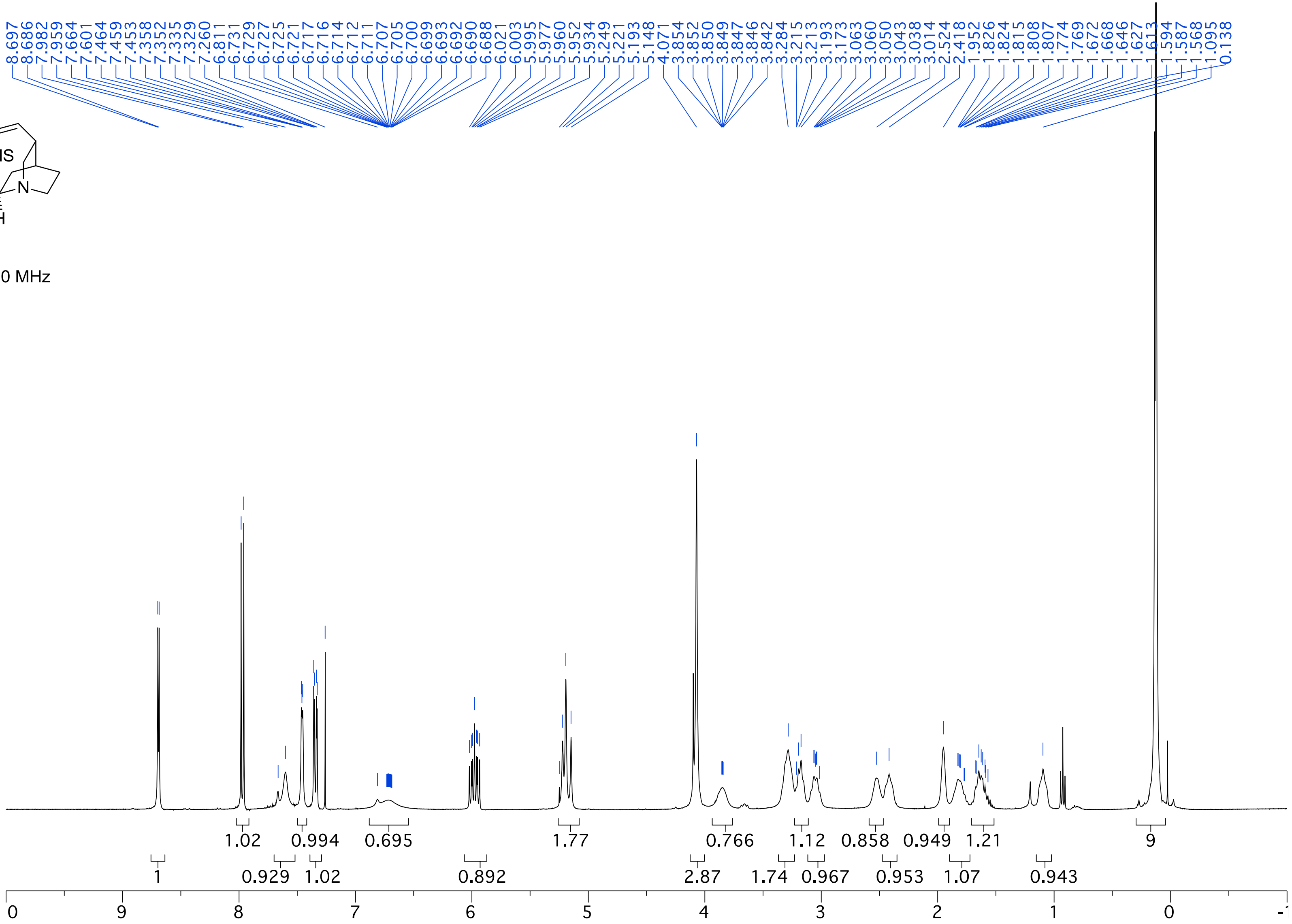

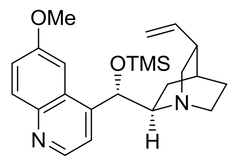

19

$^{13}C\{^{1}H\}$, $CDCl_3$, 100 MHz

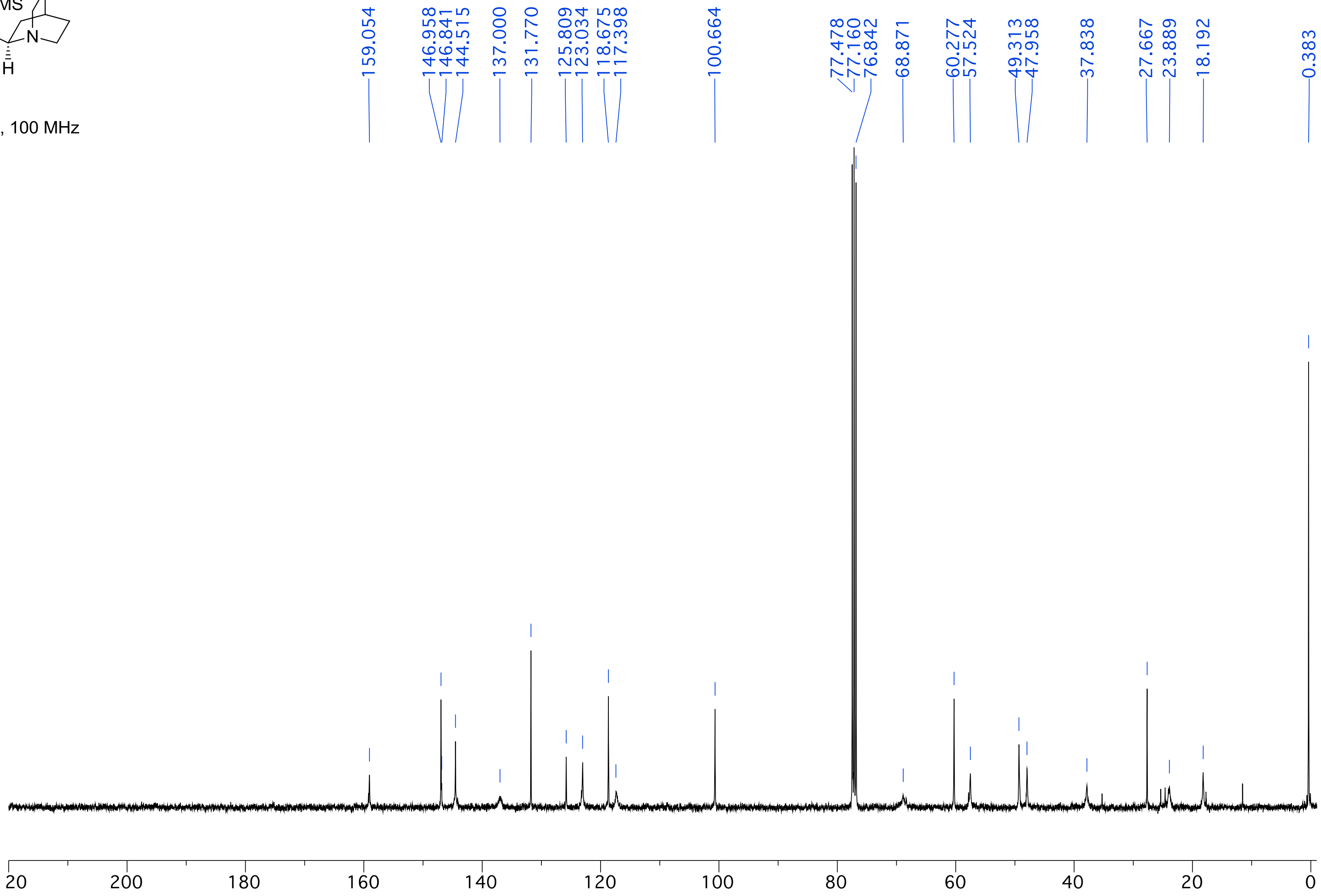

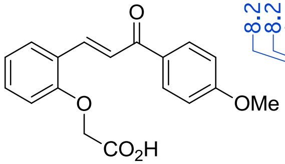

S4

^{1}H, DMSO-d_6, 300 MHz

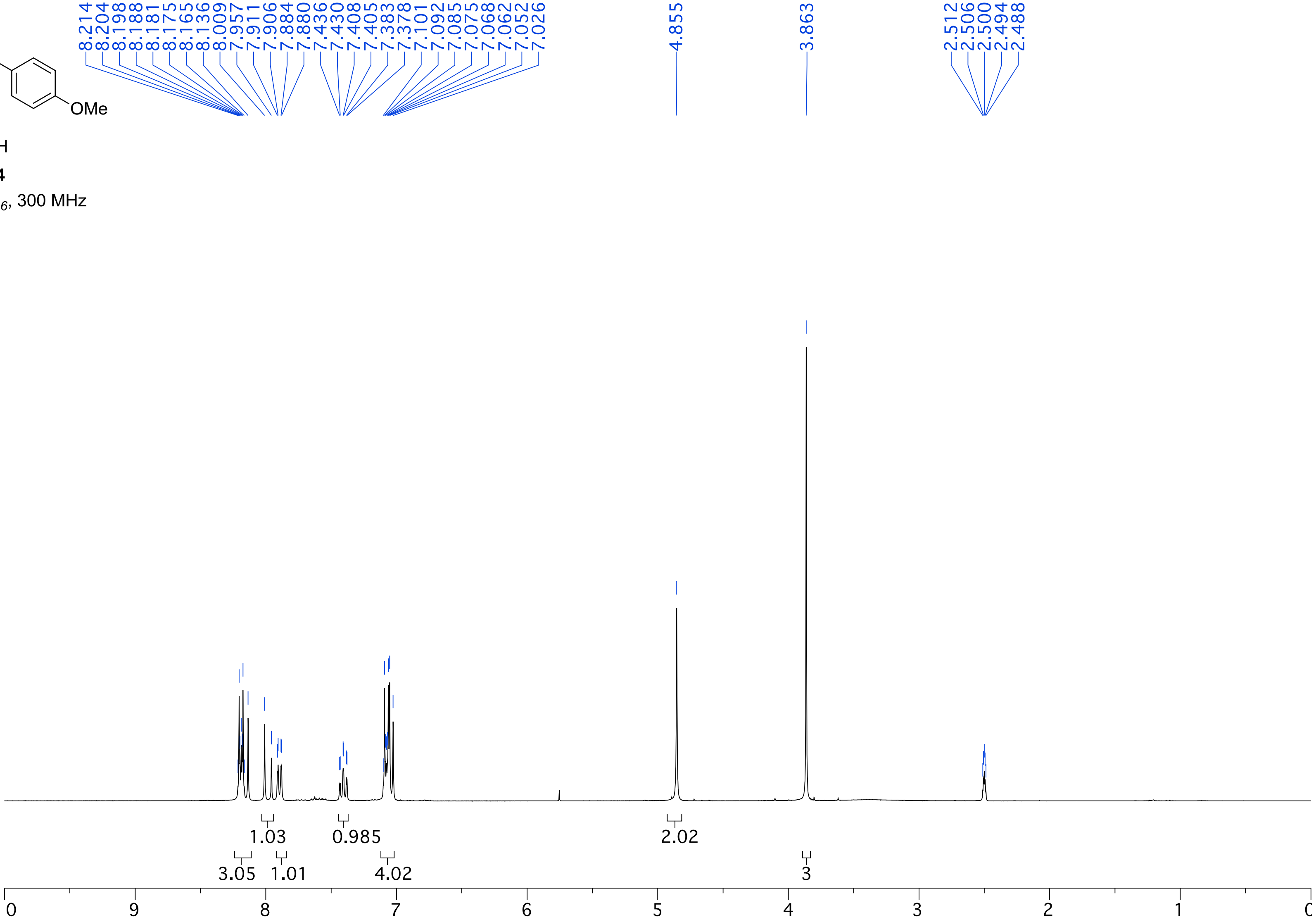

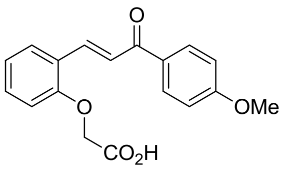

S4

$^{13}C\{^1H\}$, DMSO-d_6, 75 MHz

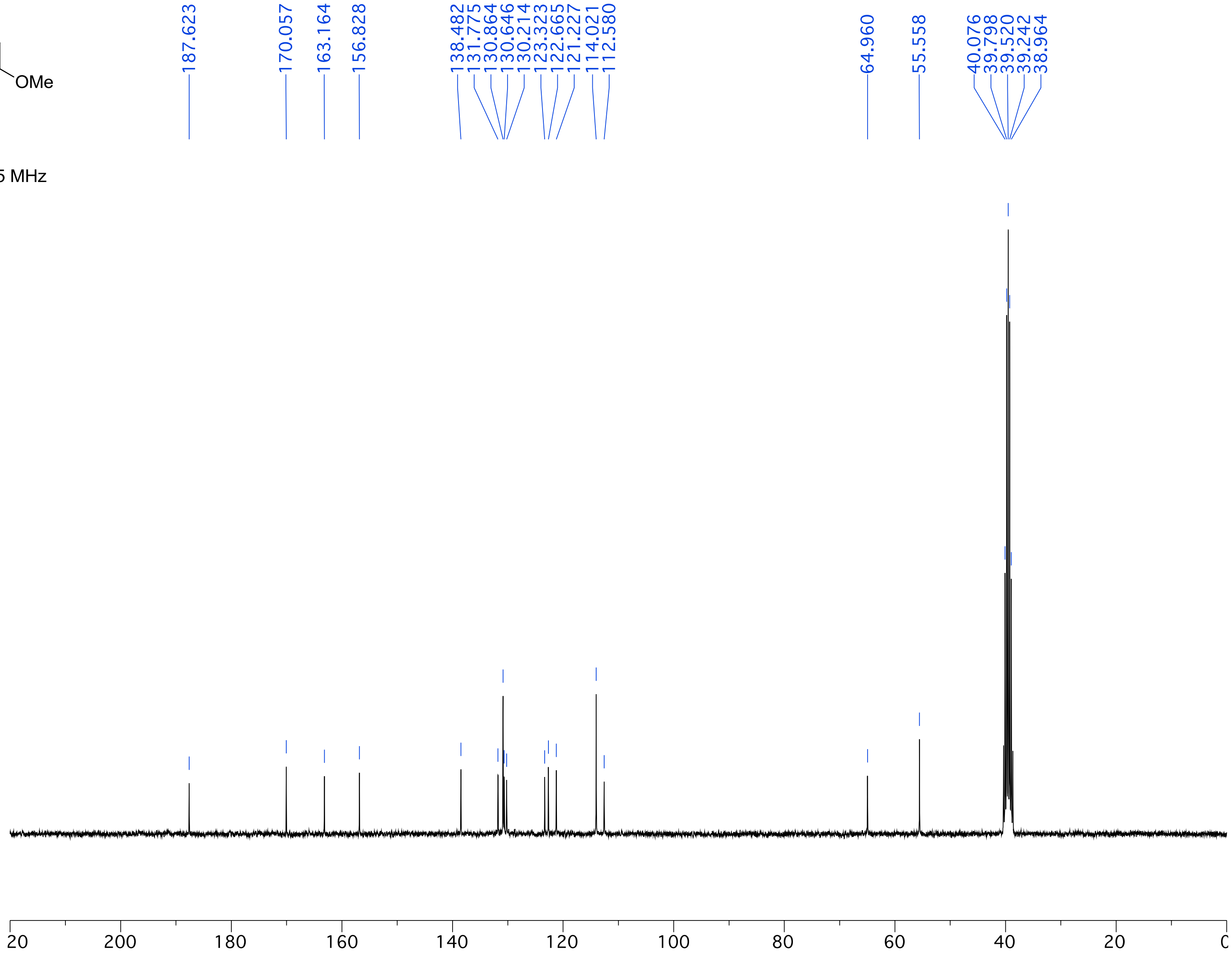

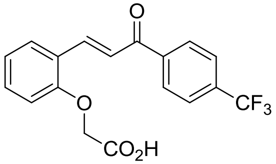

S6

^1H, DMSO-d_6, 300 MHz

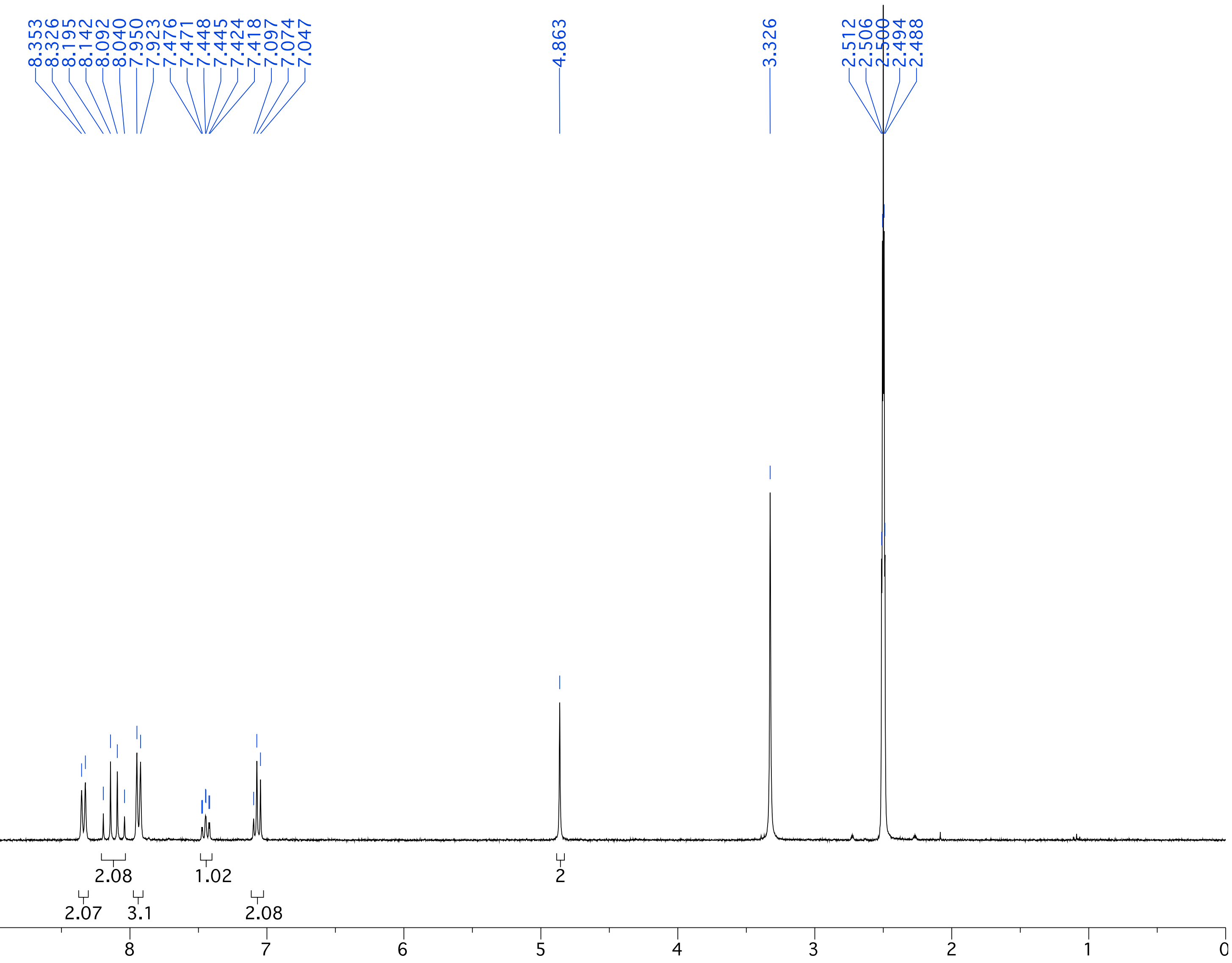

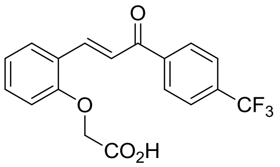

S6

$^{13}C\{^{1}H\}$, DMSO-d_6, 100 MHz

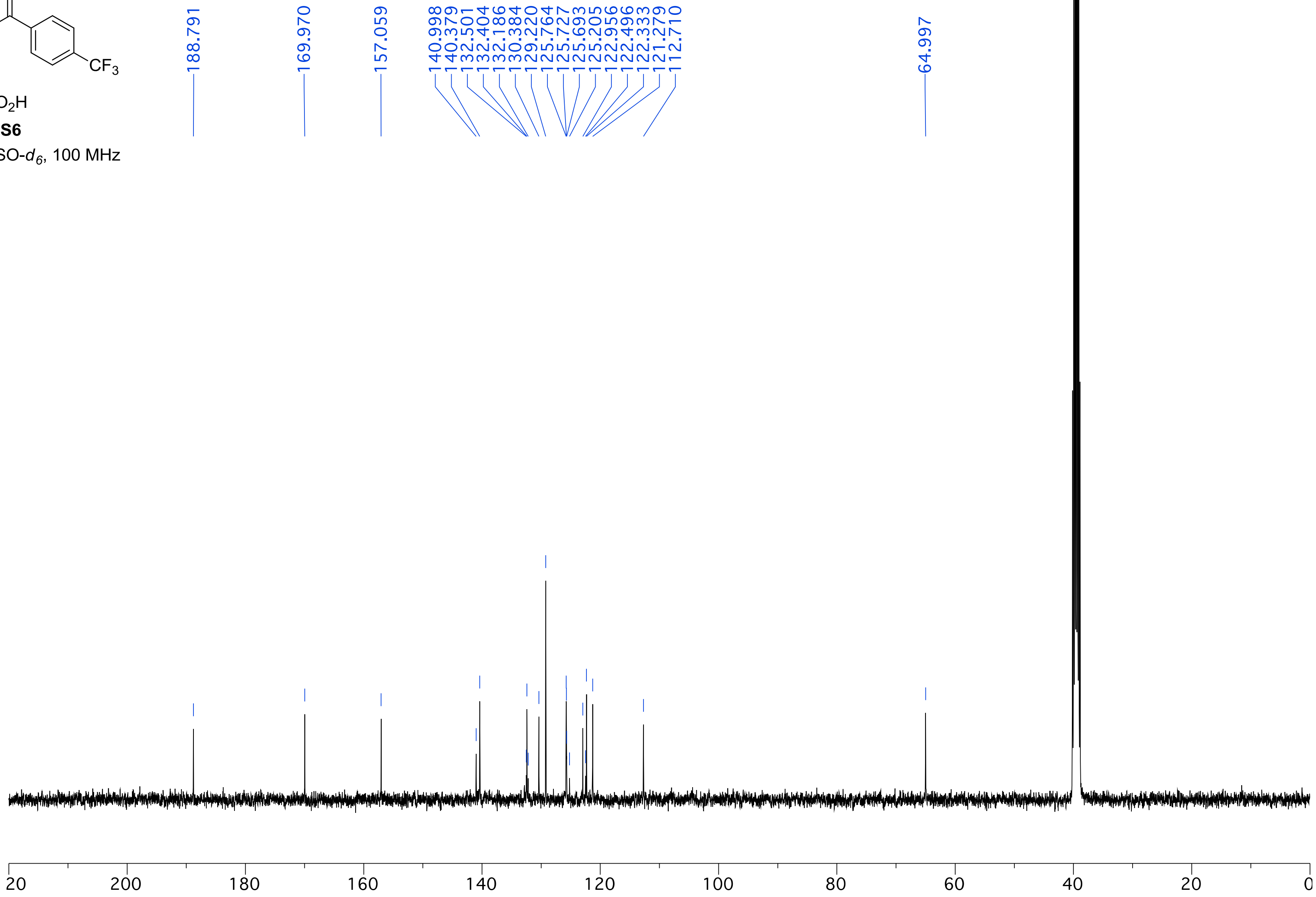

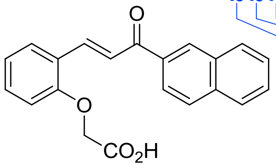

S8

^{1}H, DMSO-d_6, 400 MHz

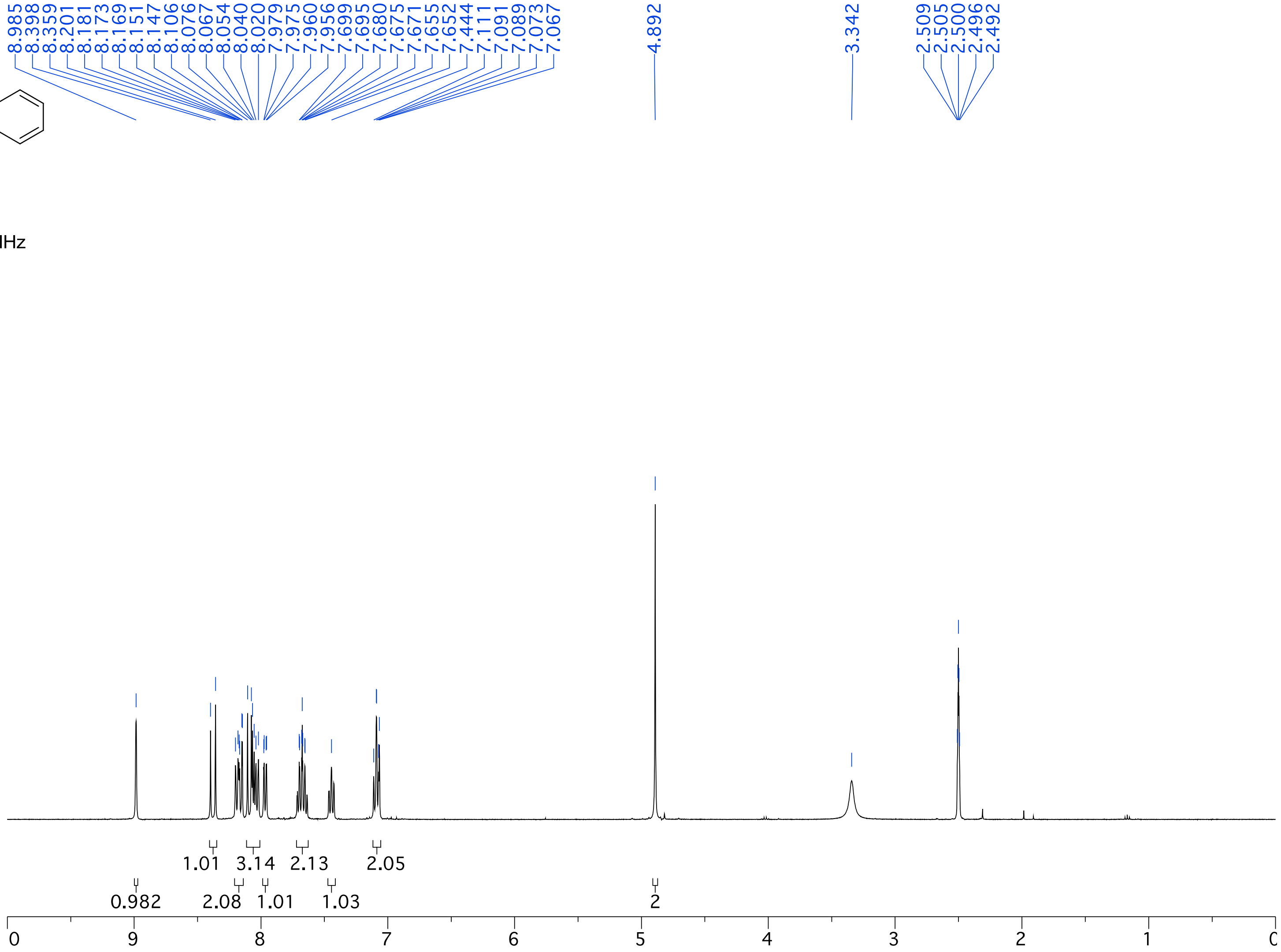

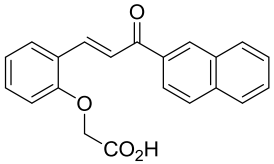

S8

$^{13}C\{^{1}H\}$, DMSO-d_6, 75 MHz

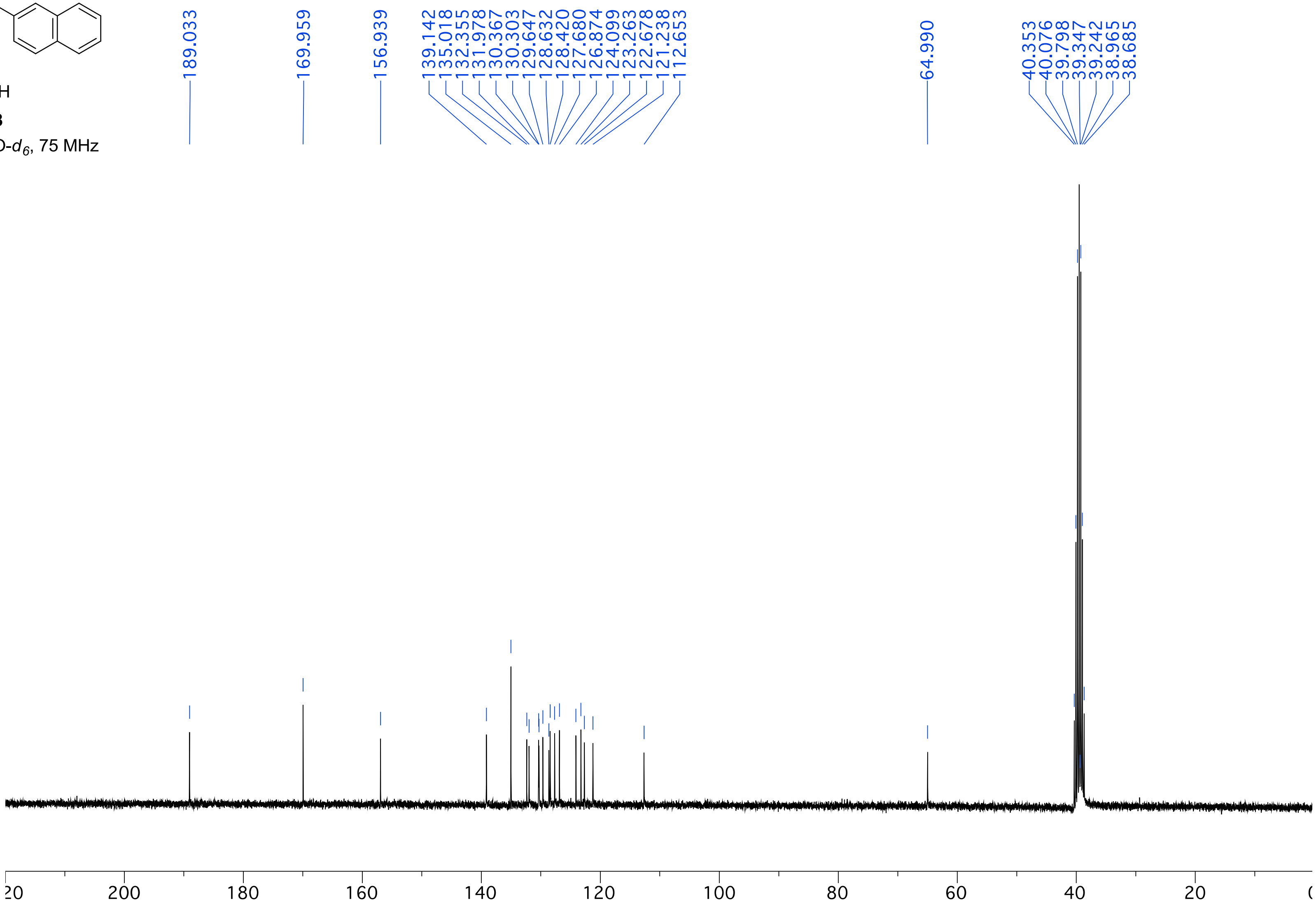

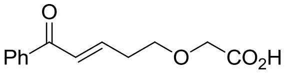

S10

^{1}H, $CDCl_3$, 500 MHz

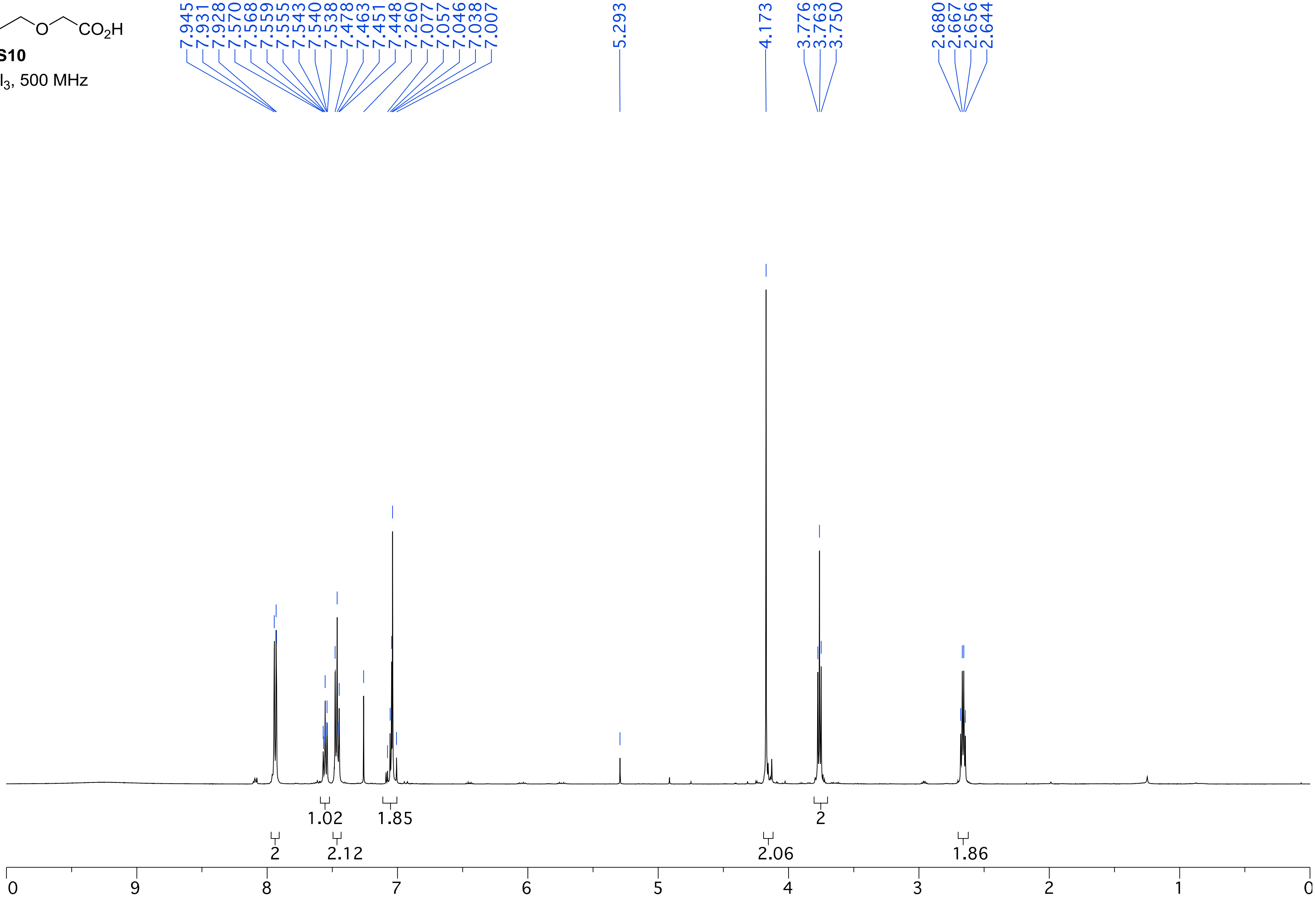

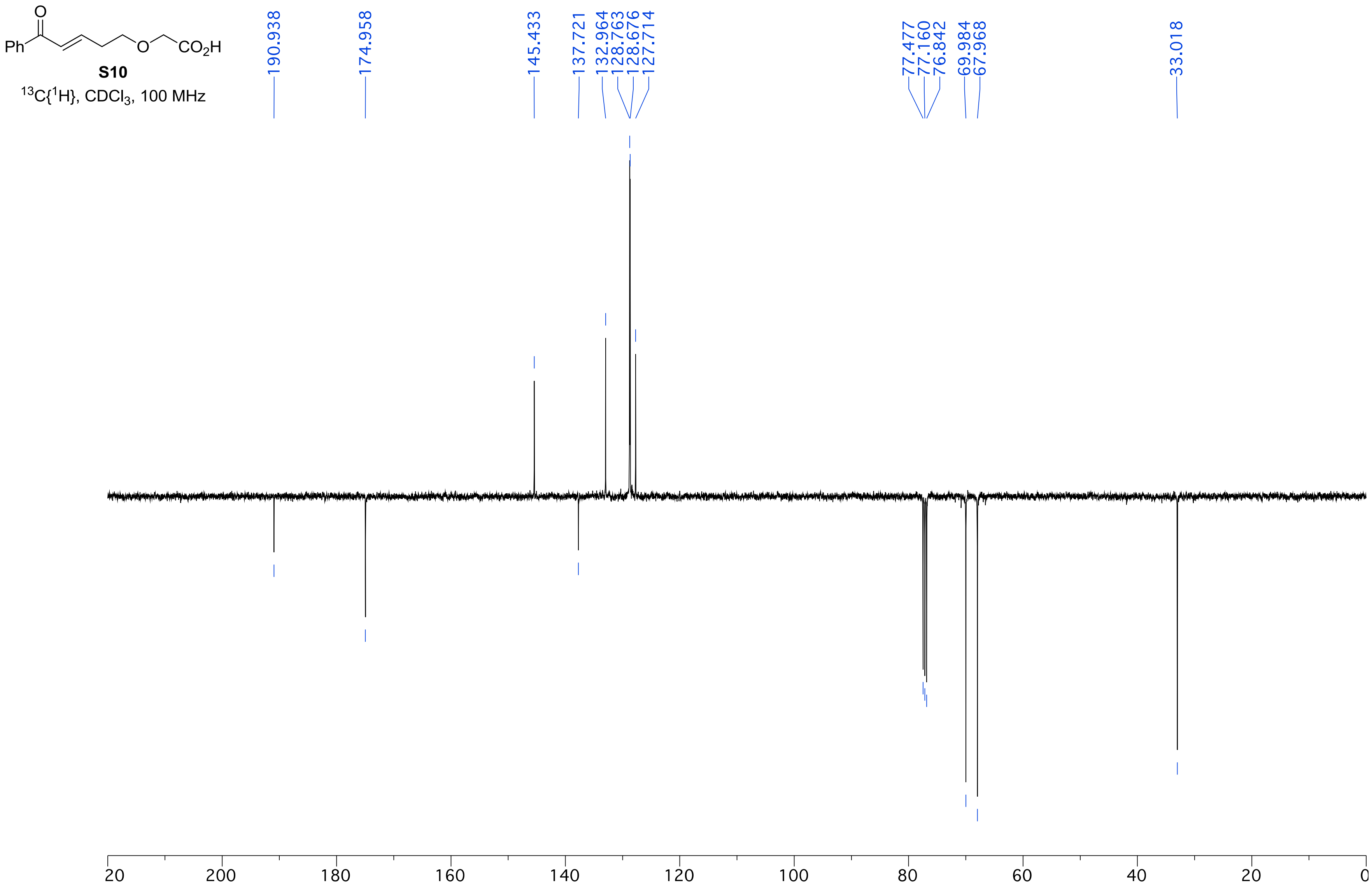

O
Ph
CO2H
S10
13C{1H}, CDCl3, 100 MHz
190.938
174.958
145.433
137.721
132.964
128.763
128.676
127.714
77.477
77.160
76.842
69.984
67.968
33.018
20
200
180
160
140
120
100
80
60
40
20
0

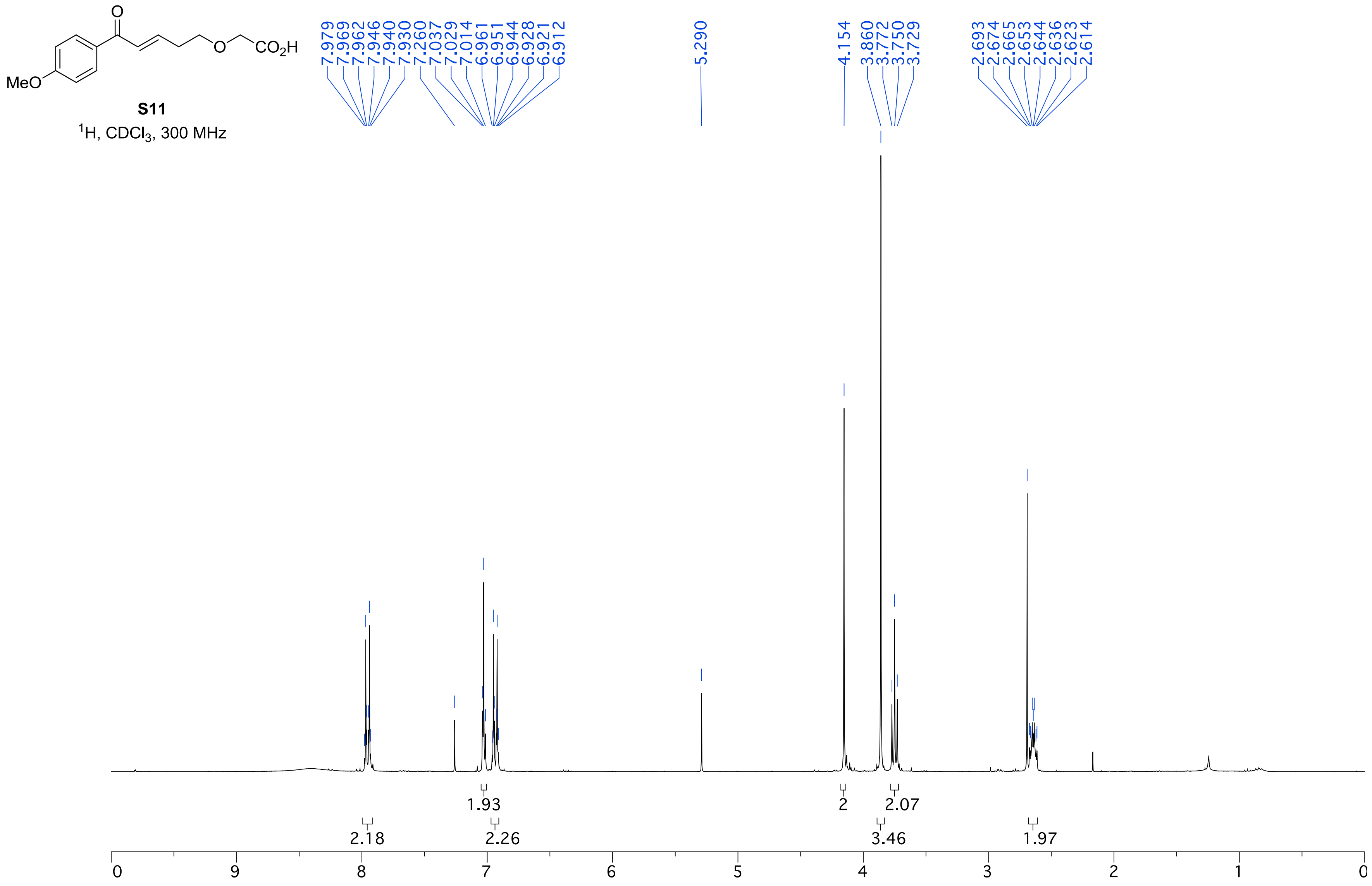
MeO
O
CO2H
S11
1H, CDCl3, 300 MHz
7.979 7.969 7.962 7.946 7.940 7.930 7.260 7.037 7.029 7.014 6.961 6.951 6.944 6.928 6.921 6.912
5.290
4.154 3.860 3.772 3.750 3.729
2.693 2.674 2.665 2.653 2.644 2.636 2.623 2.614
1.93 2 2.07
2.18 2.26 3.46 1.97
10 9 8 7 6 5 4 3 2 1 0

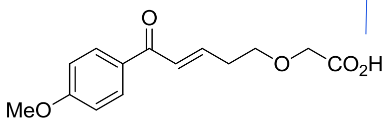

S11

$^{13}C\{^{1}H\}$, $CDCl_3$, 75 MHz

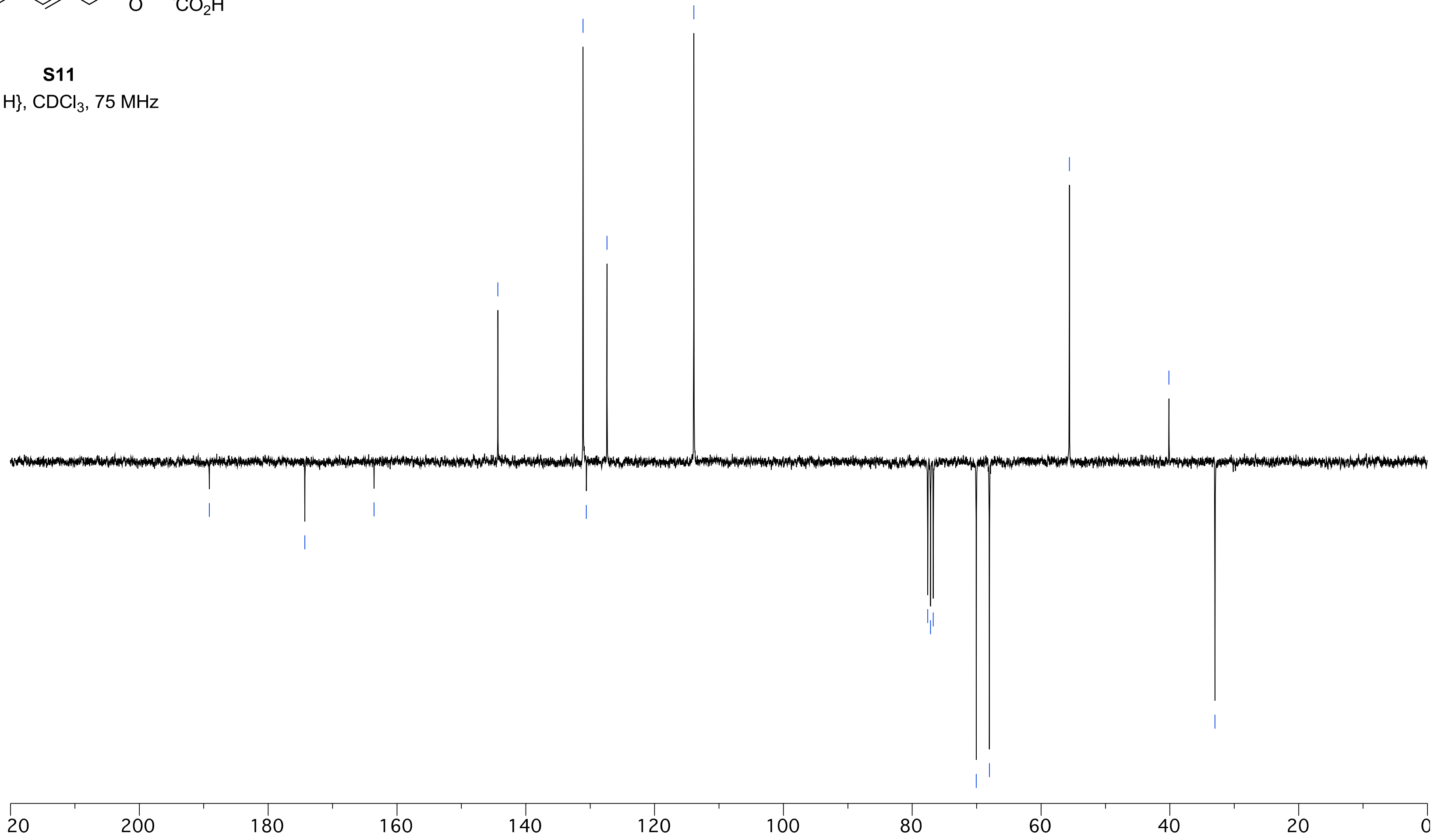

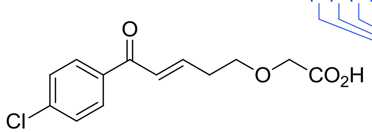

S12

^{1}H, $CDCl_3$, 300 MHz

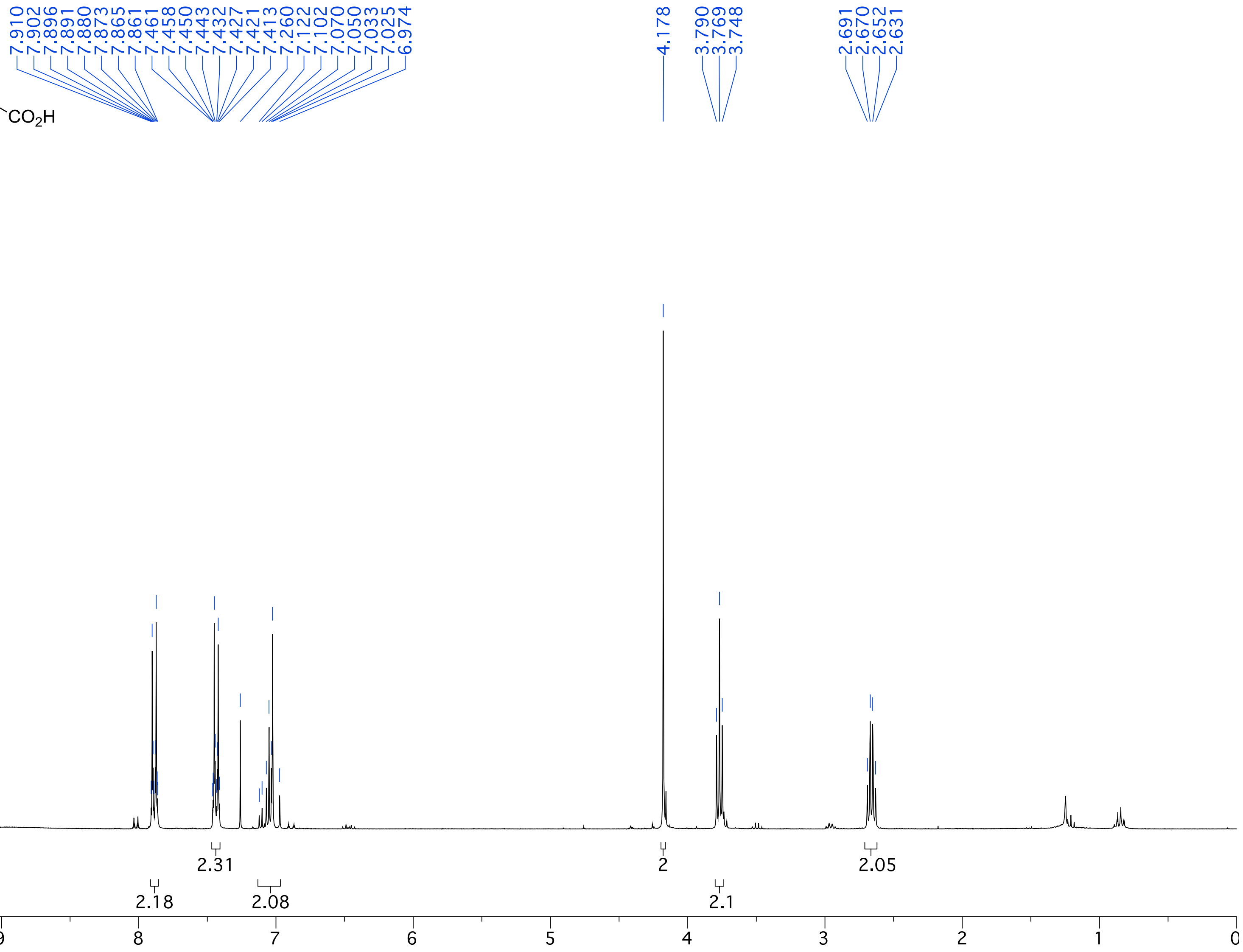

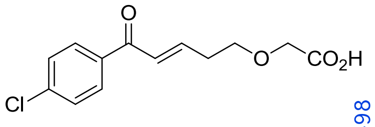

S12

$^{13}C\{^{1}H\}$, $CDCl_3$, 75 MHz

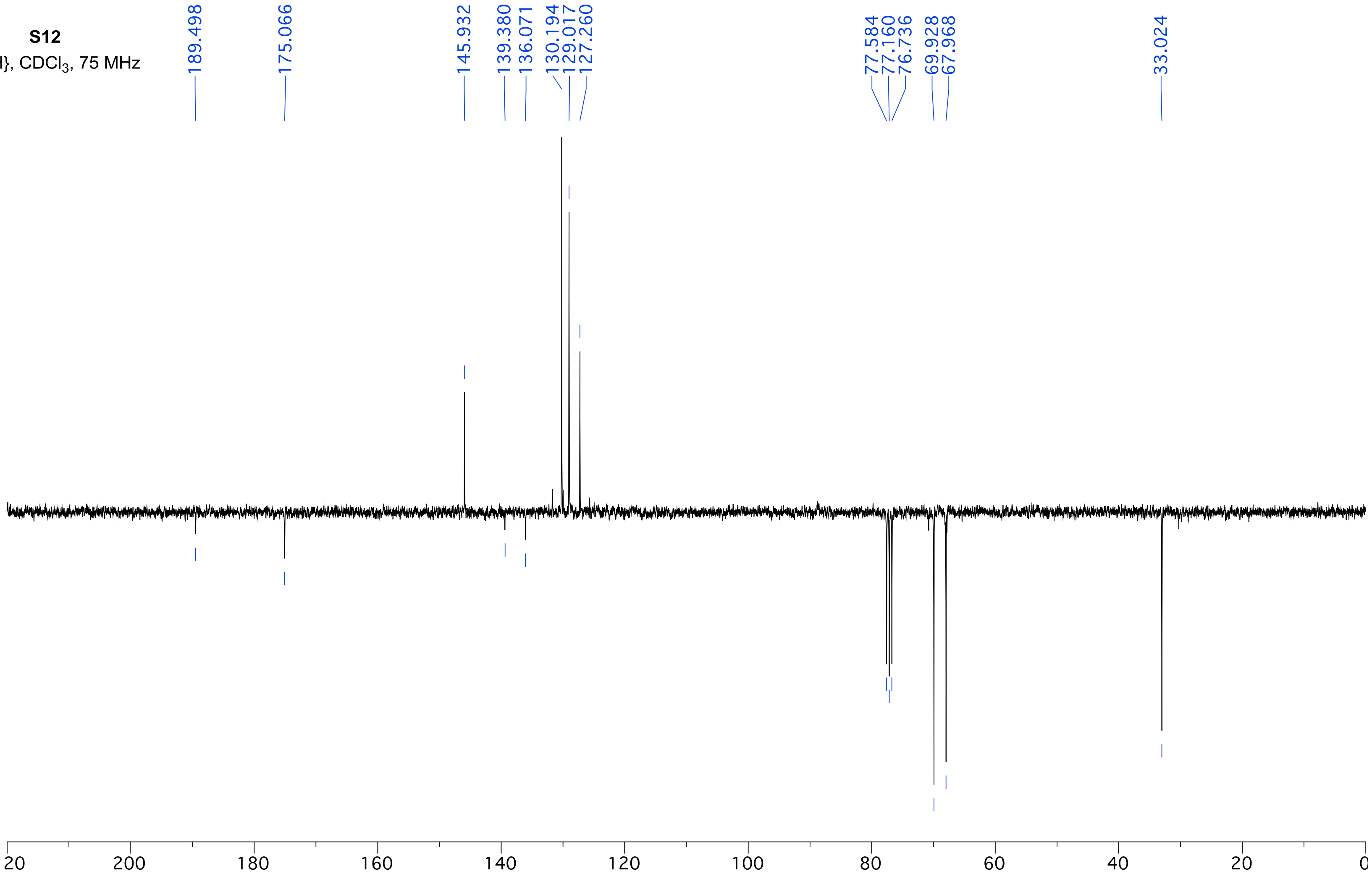

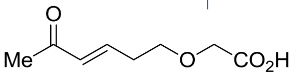

40

^{1}H, $CDCl_3$, 500 MHz

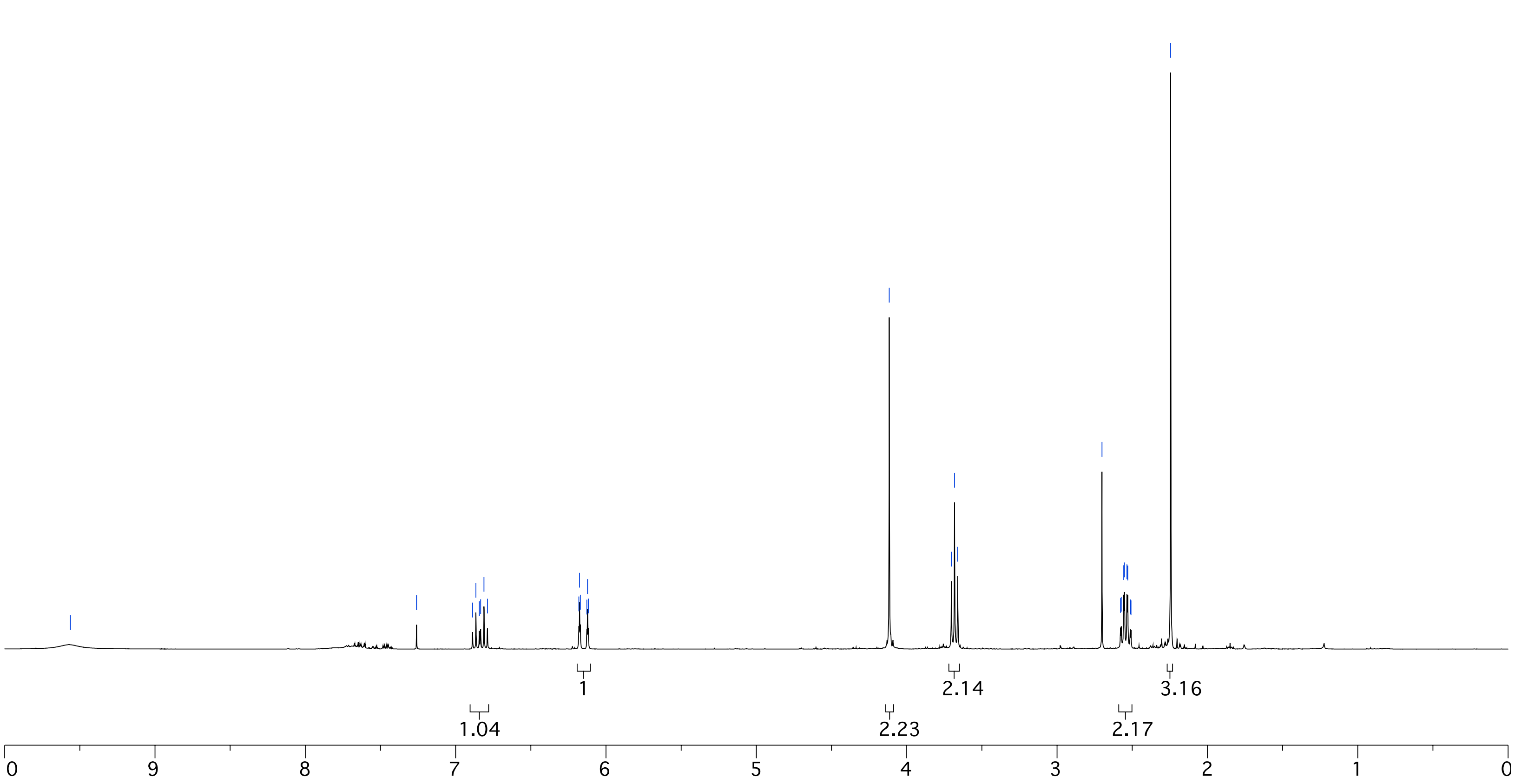

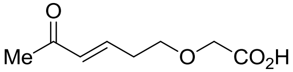

40

$^{13}C\{^{1}H\}$, $CDCl_3$, 75 MHz

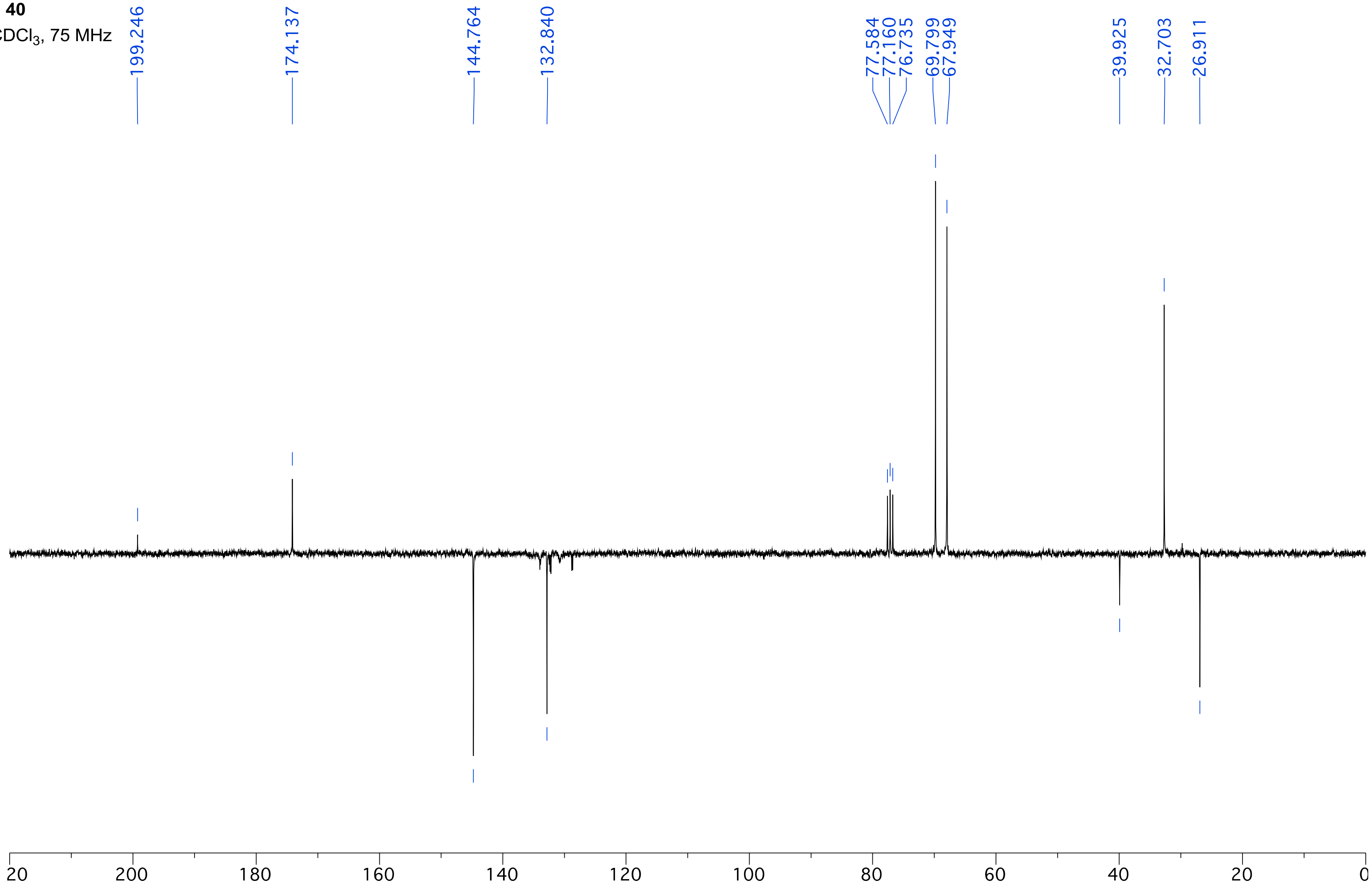

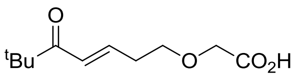

S13

^{1}H, $CDCl_3$, 300 MHz

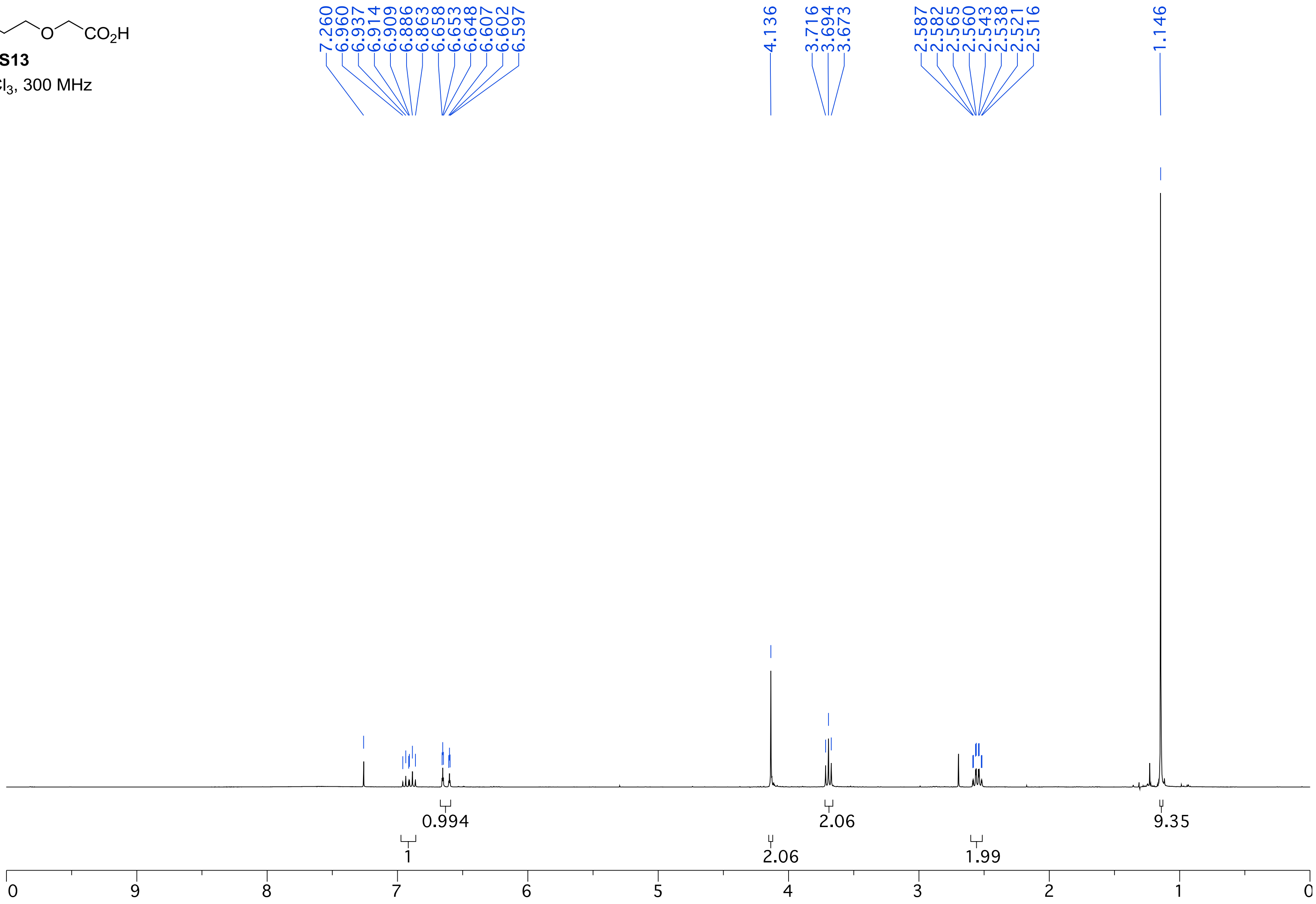

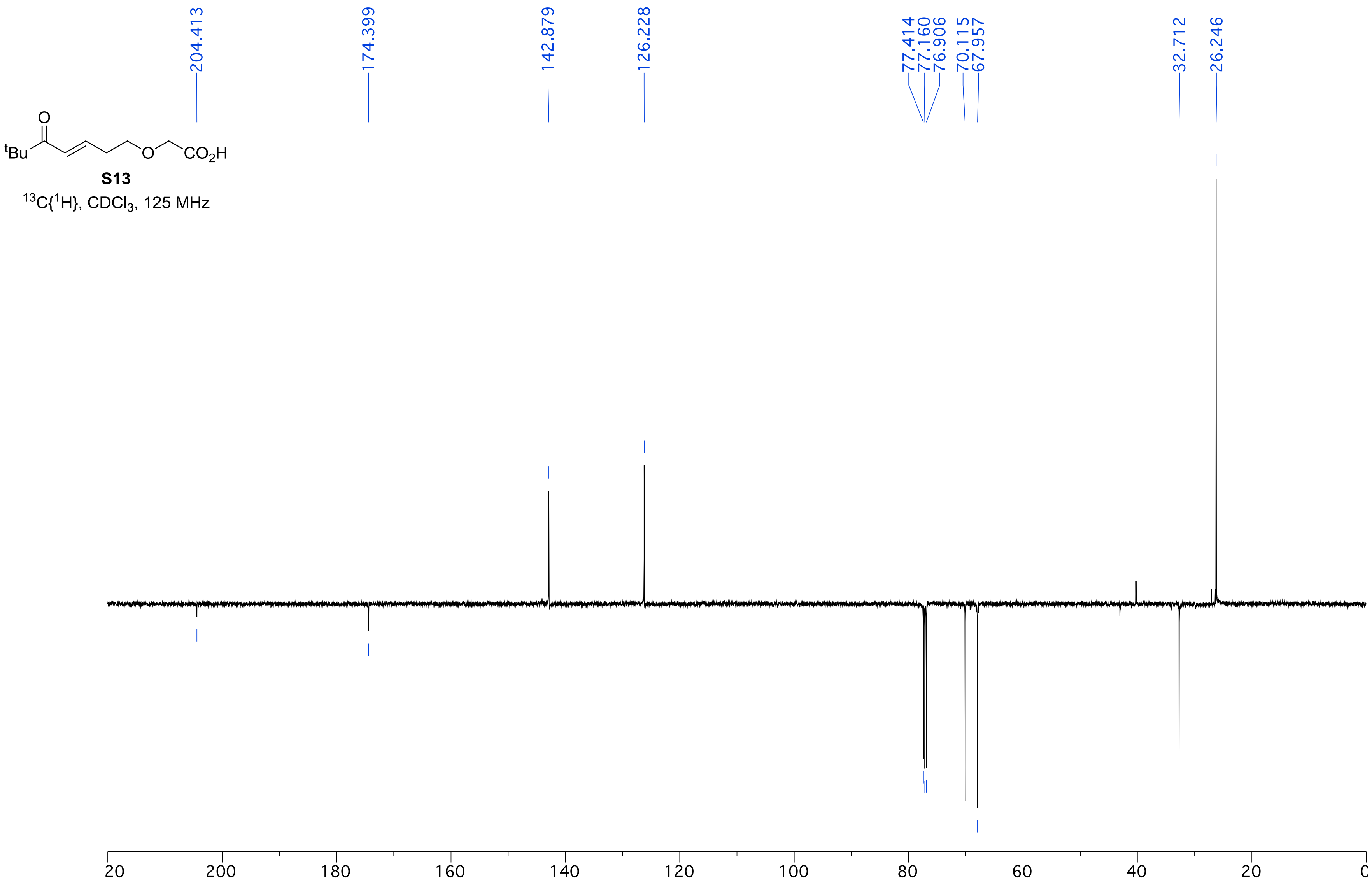
O
tBu
CO2H
S13
13C{1H}, CDCl3, 125 MHz
204.413
174.399
142.879
126.228
77.414
77.160
76.906
70.115
67.957
32.712
26.246
20
200
180
160
140
120
100
80
60
40
20
0

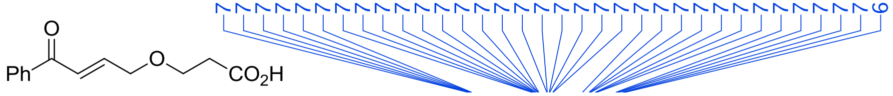

S14

^{1}H, $CDCl_3$, 400 MHz

96:4 *E*:*Z*

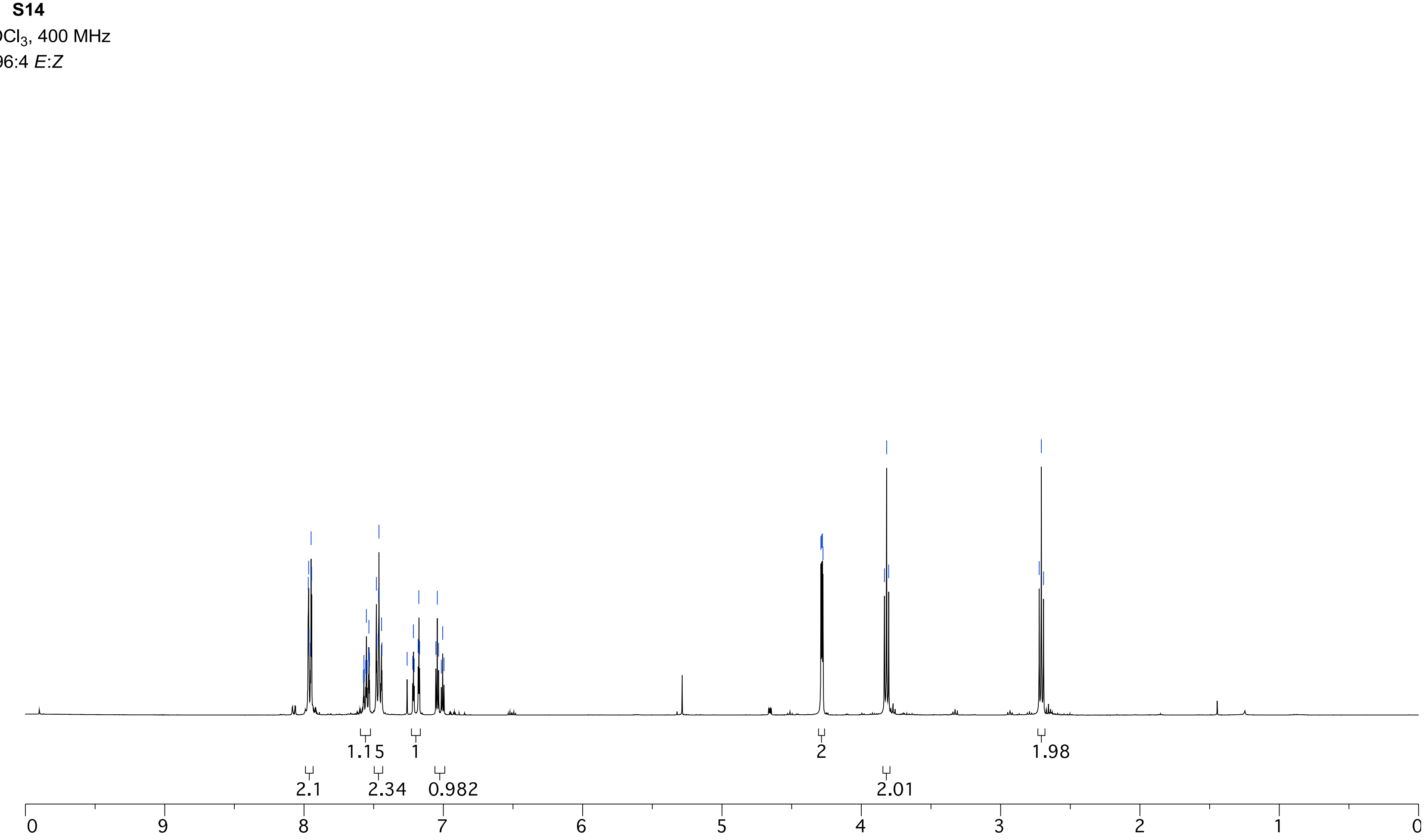

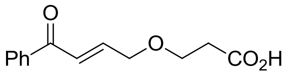

S14

$^{13}C\{^{1}H\}$, $CDCl_3$, 100 MHz

96:4 *E*:*Z*

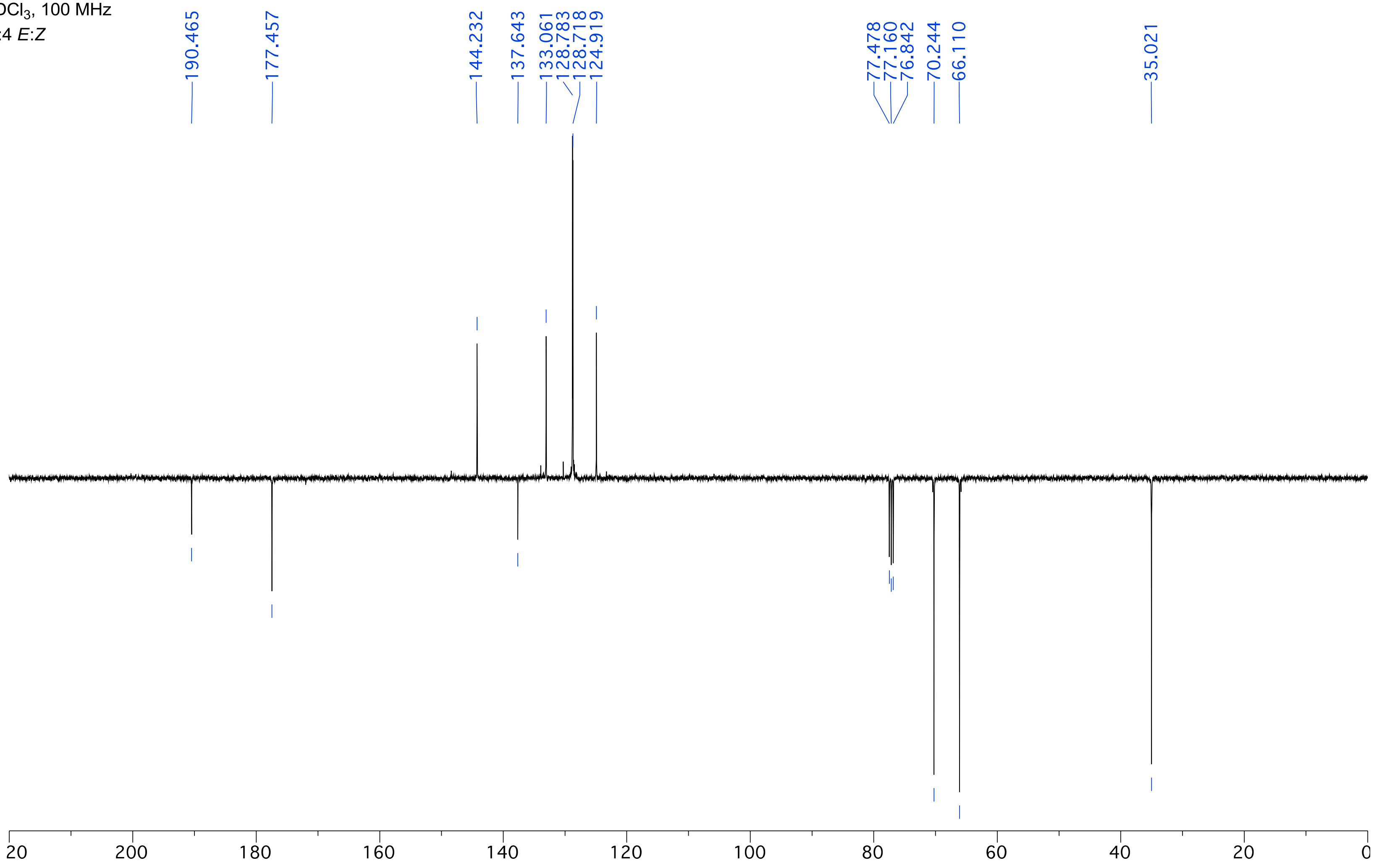

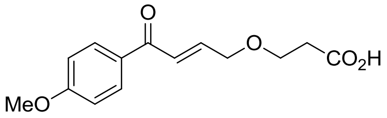

S15

^{1}H, $CDCl_3$, 300 MHz

96:4 *E*:*Z*

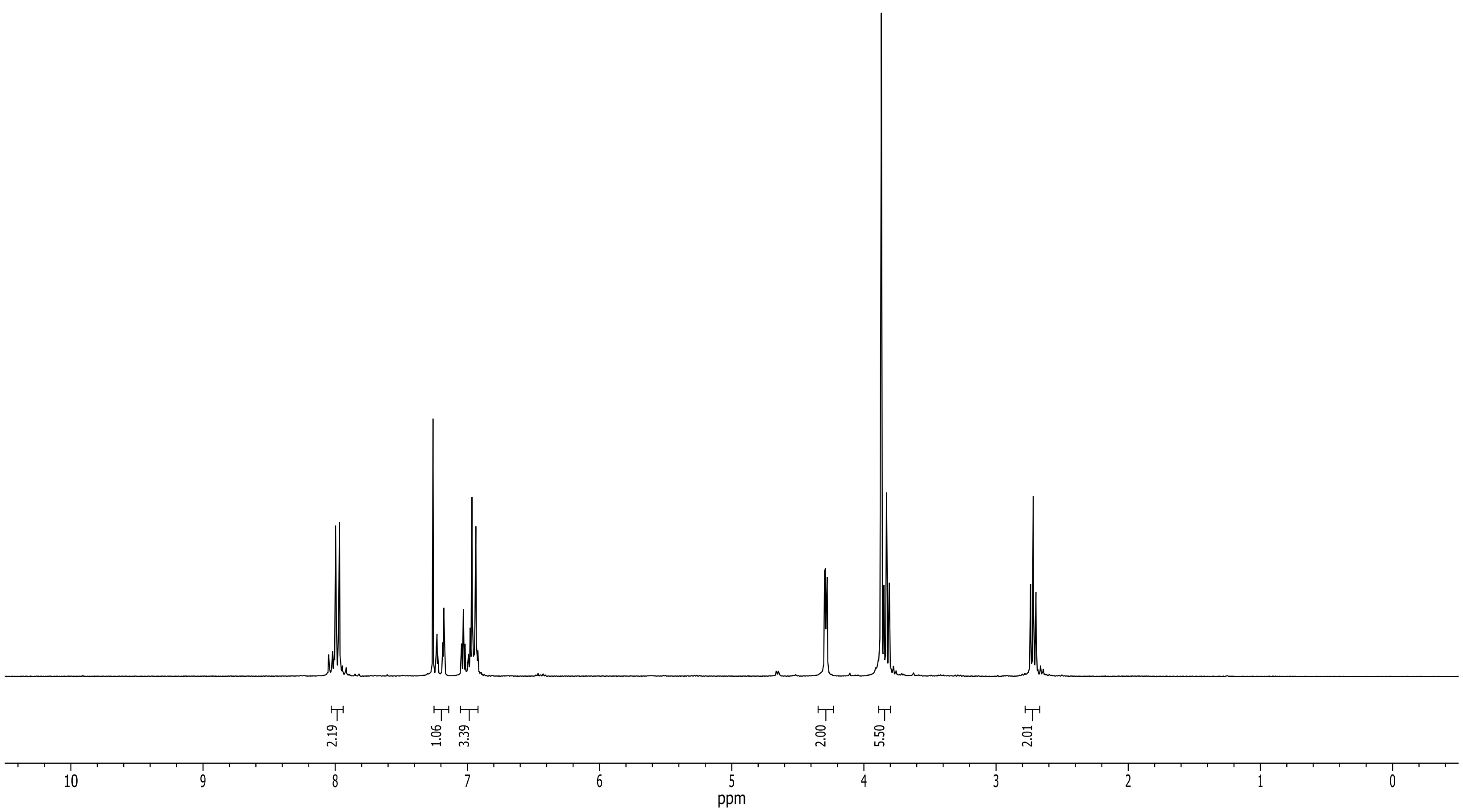

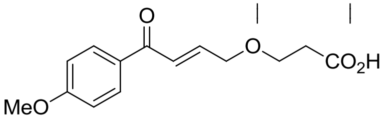

S15

$^{13}C\{^{1}H\}$, $CDCl_3$, 75 MHz

96:4 *E*:*Z*

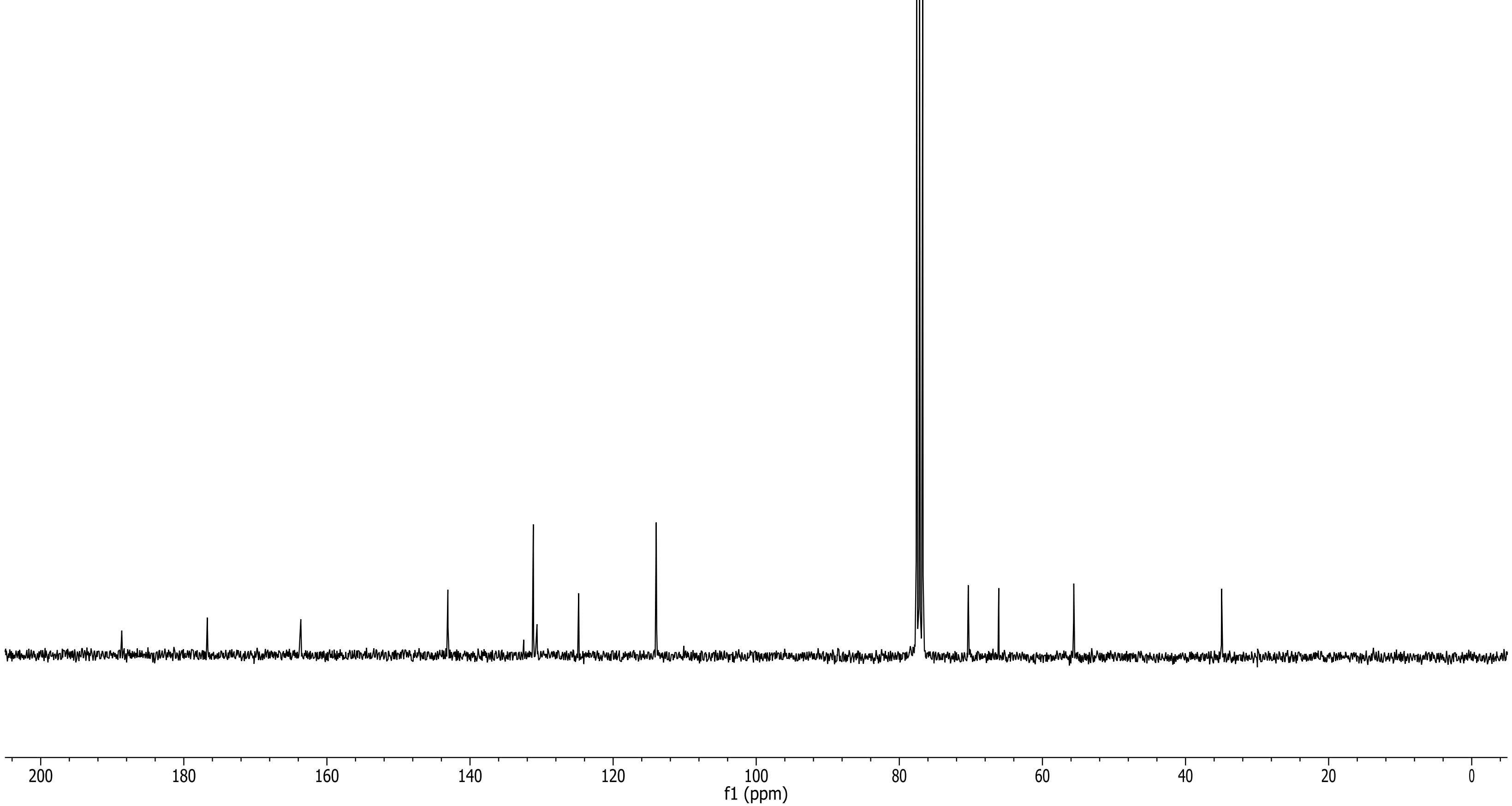

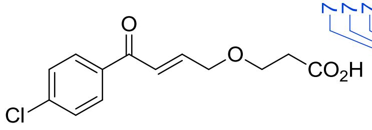

S16

^{1}H, $CDCl_3$, 400 MHz

96:4 *E*:*Z*

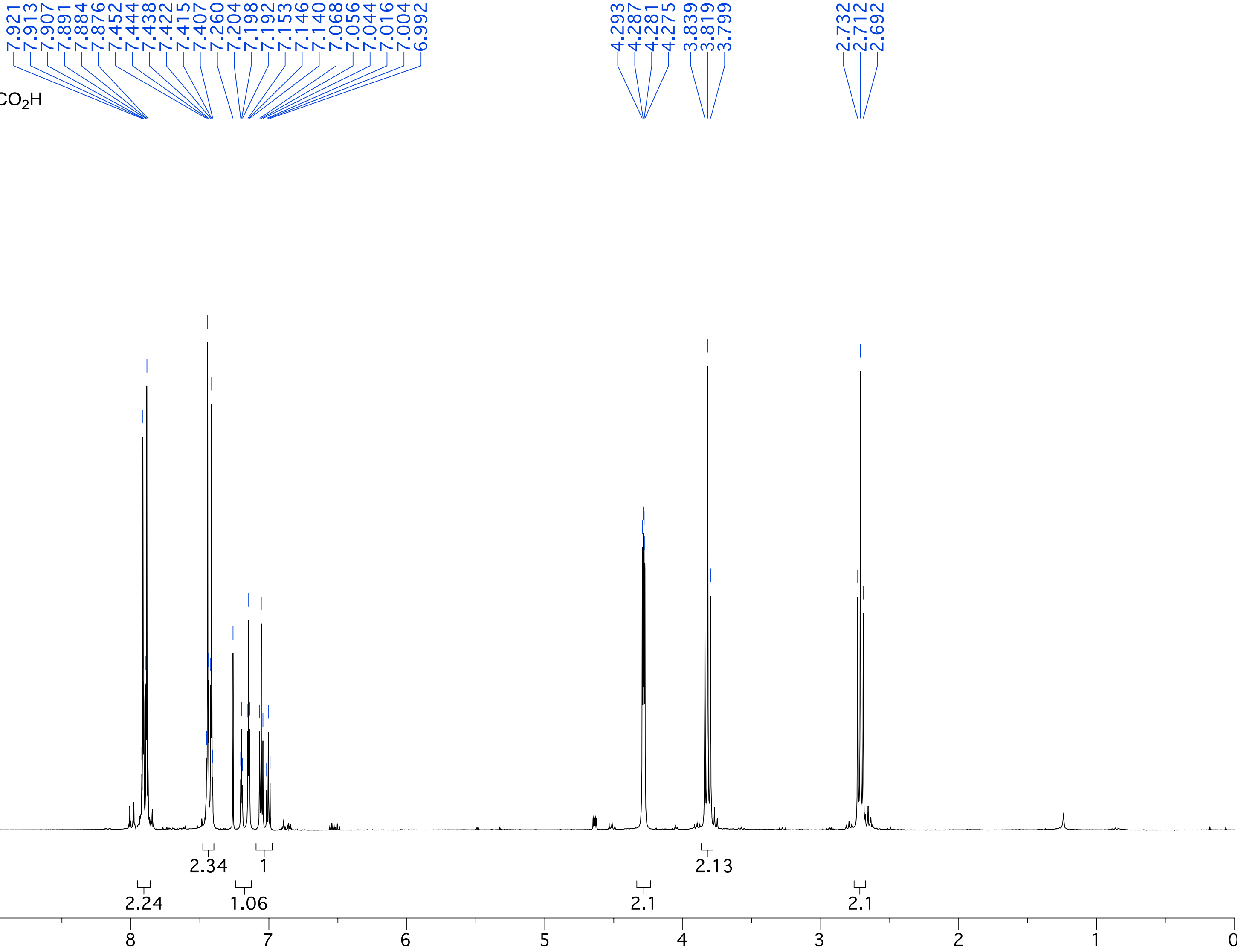

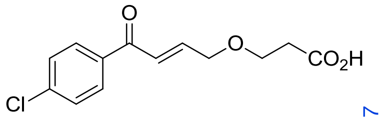

S16

$^{13}C\{^{1}H\}$, $CDCl_3$, 100 MHz

96:4 *E*:*Z*

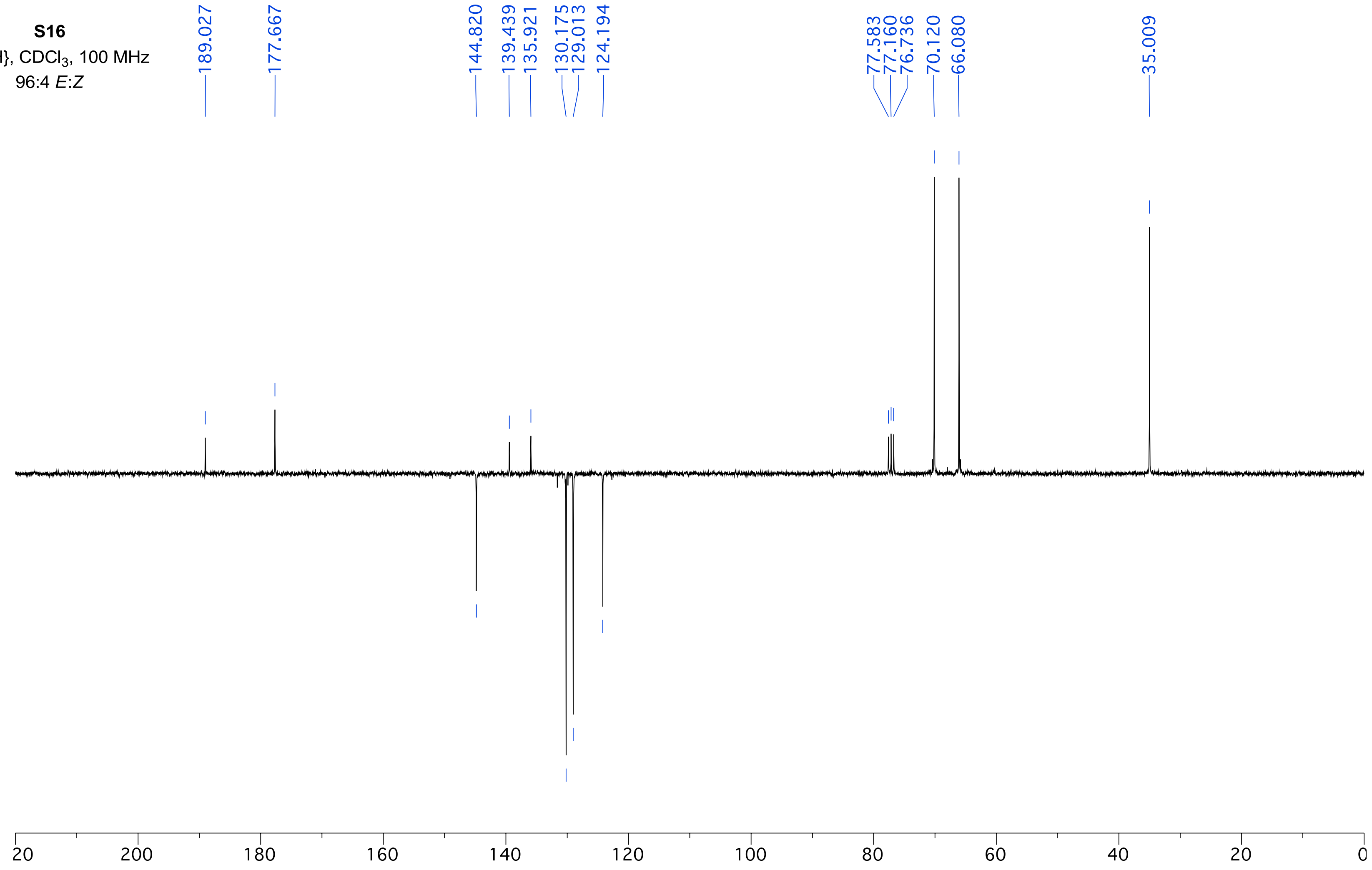

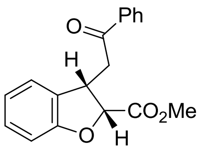

ent-**10**
^{1}H, $CDCl_3$, 400 MHz
>99:1 $dr_{syn:anti}$

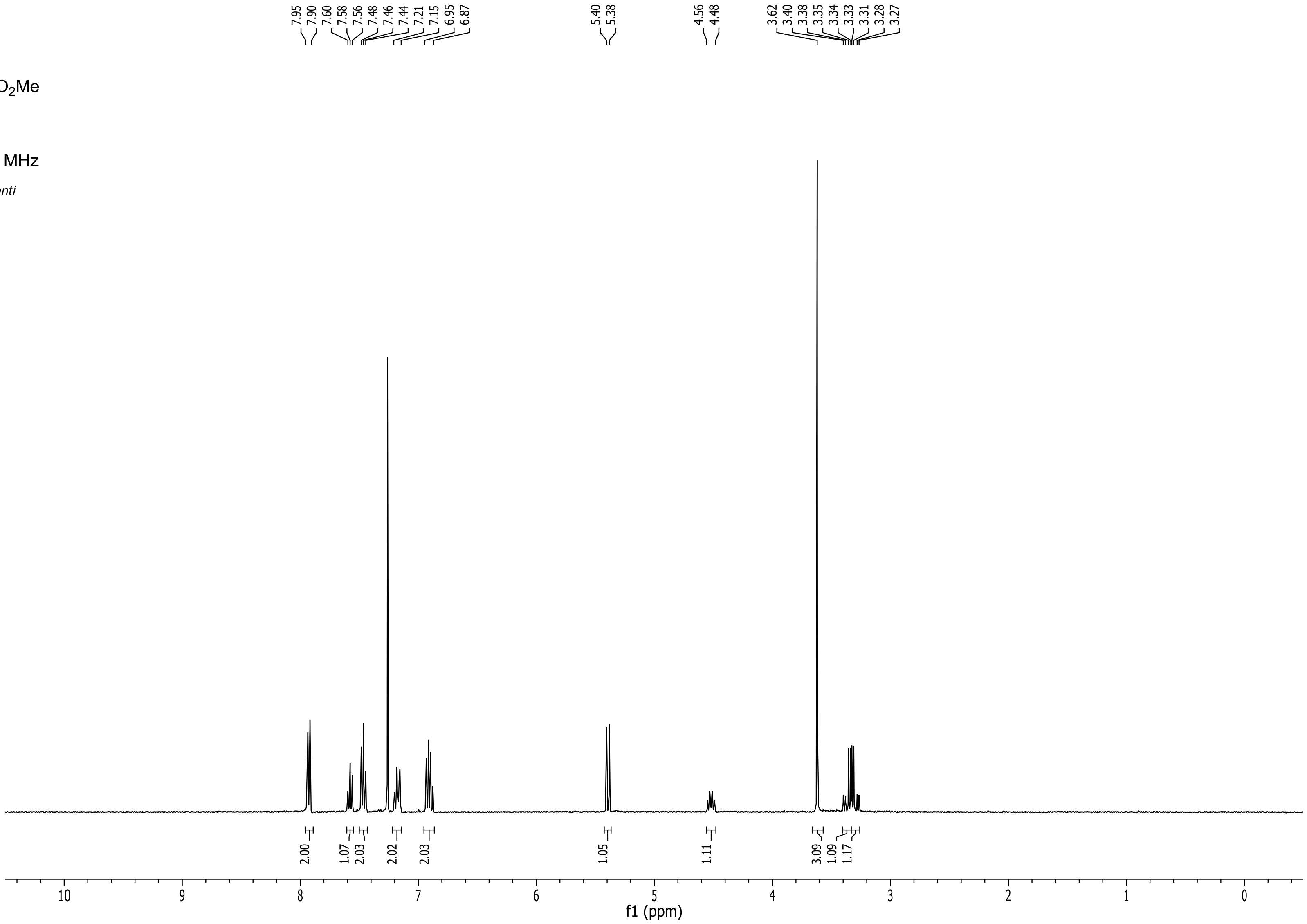

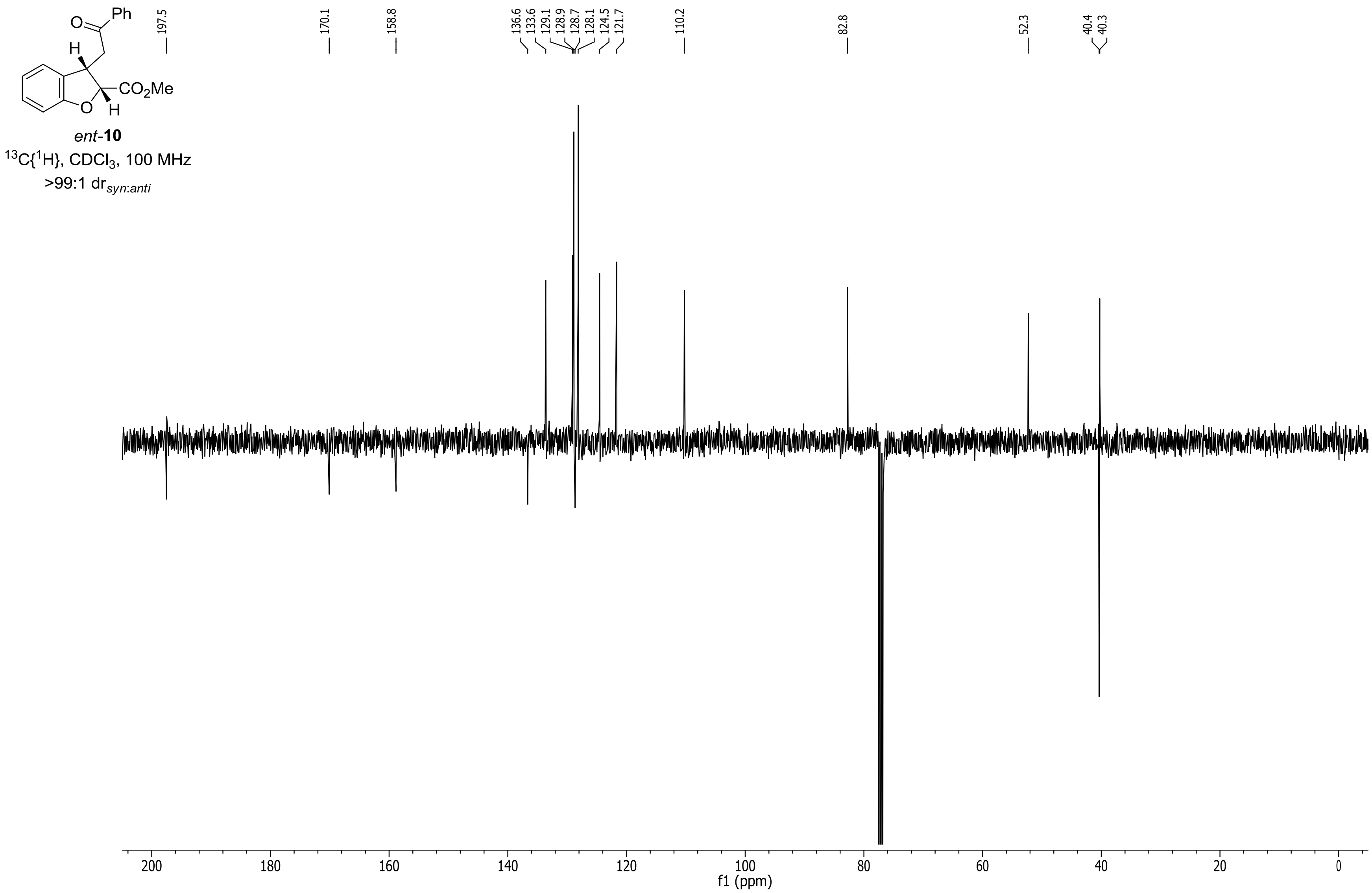

Ph
O
H
CO2Me
O
H
ent-10
13C{1H}, CDCl3, 100 MHz
>99:1 dr syn:anti
197.5
170.1
158.8
136.6
133.6
129.1
128.9
128.7
128.1
124.5
121.7
110.2
82.8
52.3
40.4
40.3
200
180
160
140
120
100
80
60
40
20
0
f1 (ppm)

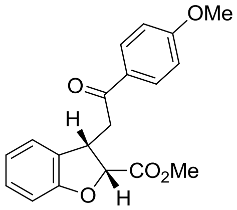

20

^{1}H, $CDCl_3$, 300 MHz

>99:1 $dr_{syn:anti}$

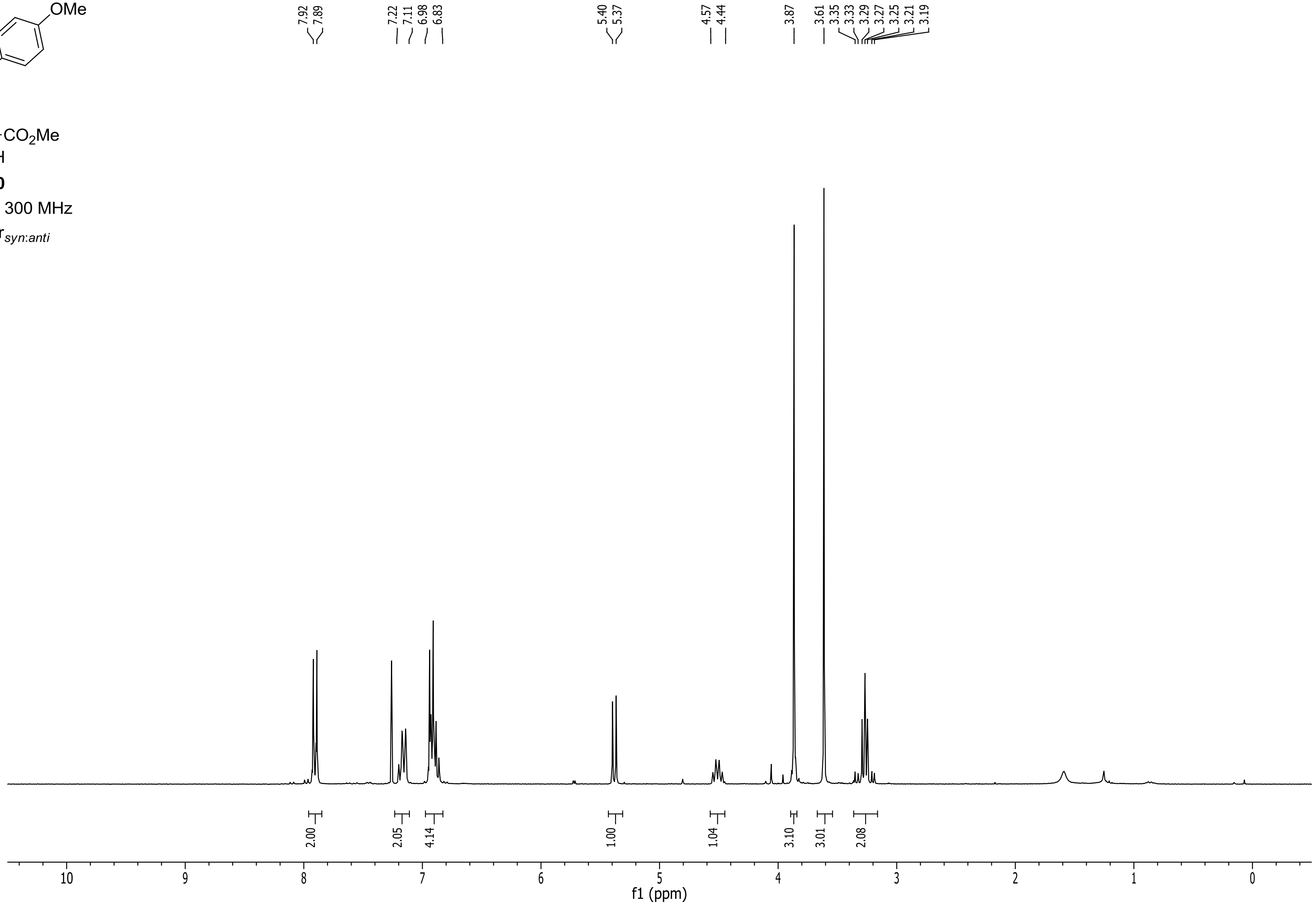

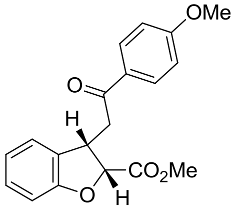

20

$^{13}C\{^{1}H\}$, $CDCl_3$, 100 MHz

>99:1 $dr_{syn:anti}$

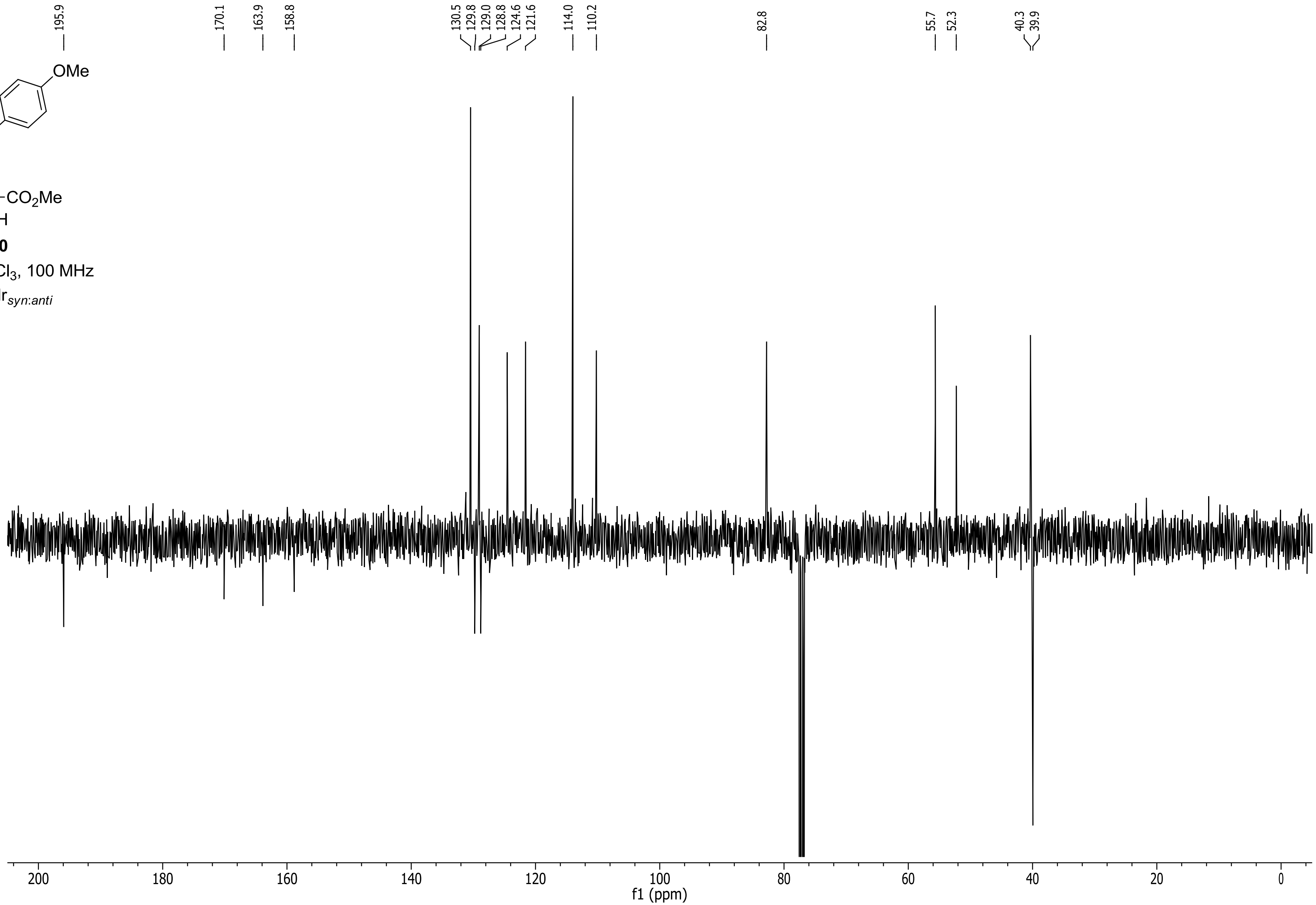

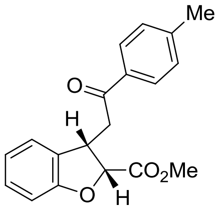

21

^{1}H, $CDCl_3$, 300 MHz

>99:1 $dr_{syn:anti}$

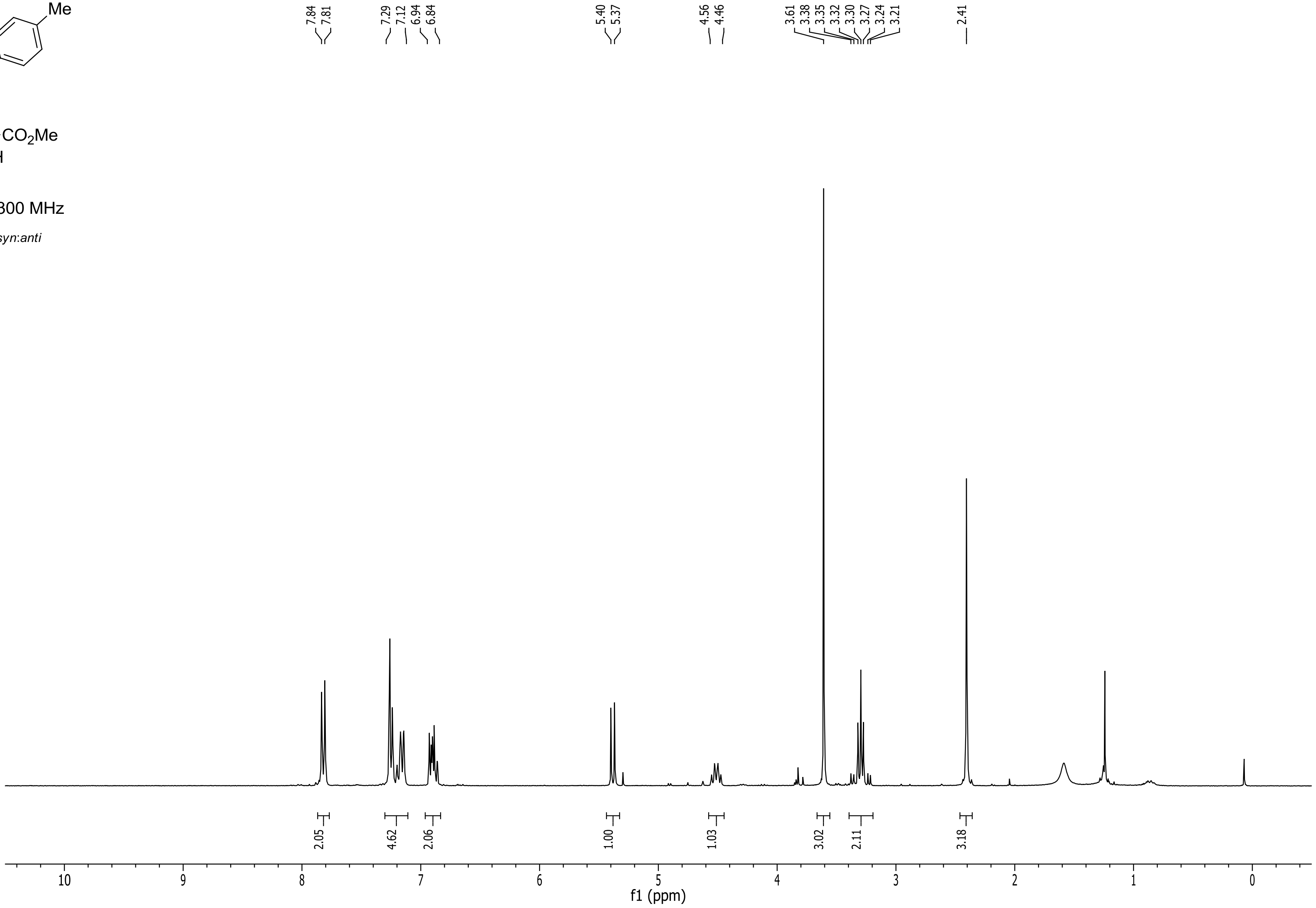

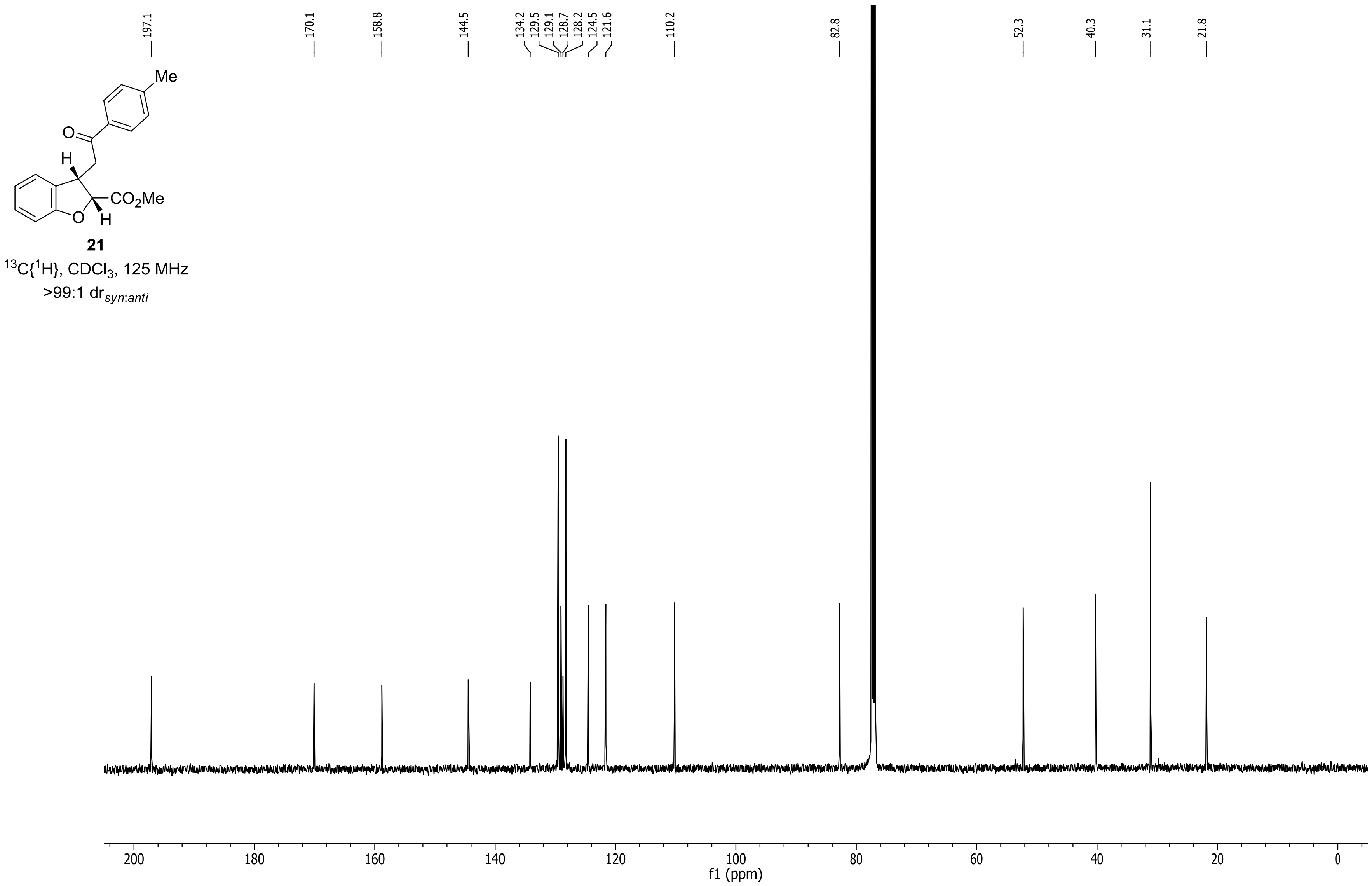

21

$^{13}C\{^{1}H\}$, $CDCl_3$, 125 MHz

>99:1 $dr_{syn:anti}$

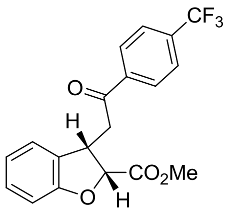

22

^{1}H, $CDCl_3$, 500 MHz

>99:1 $dr_{syn:anti}$

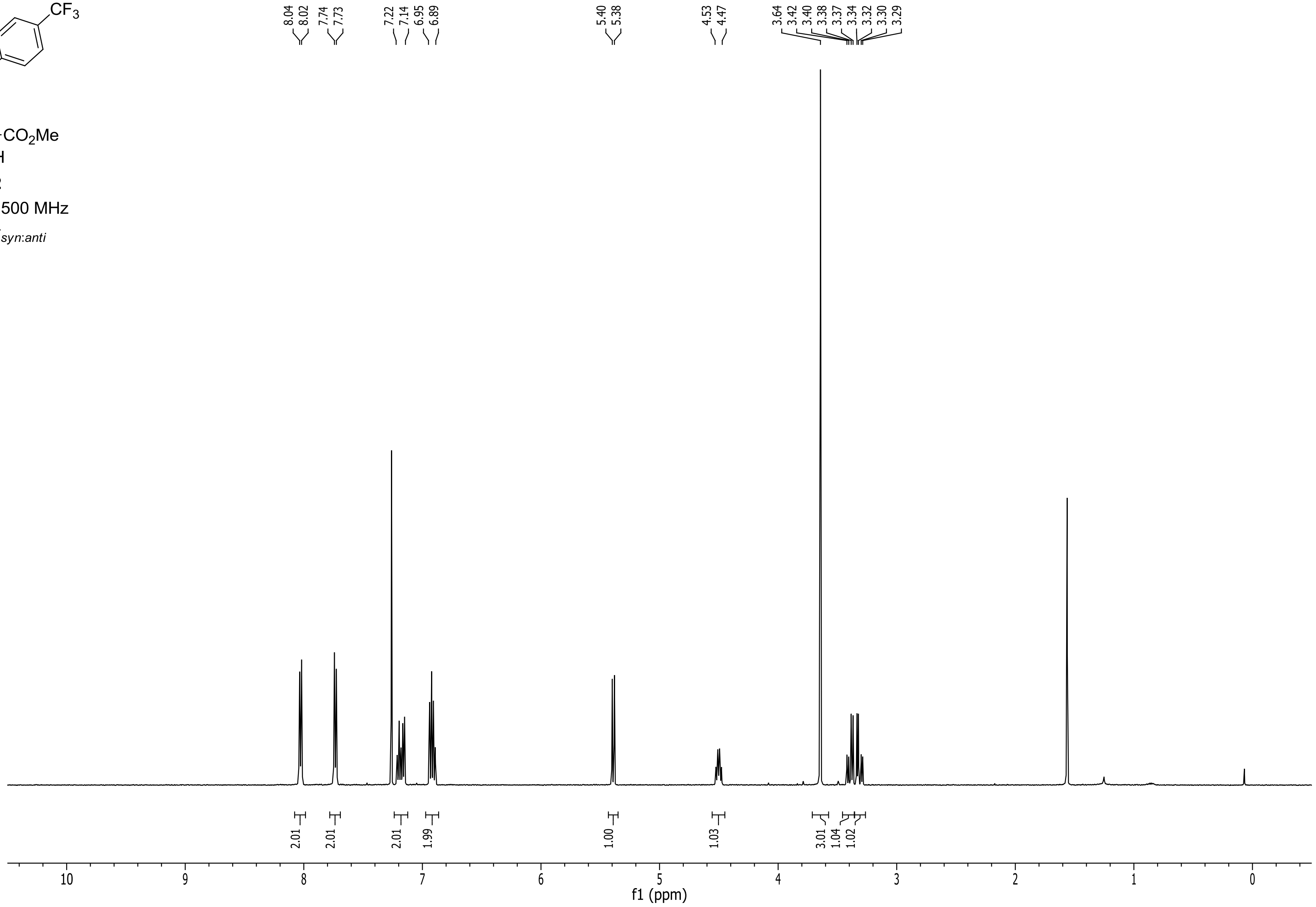

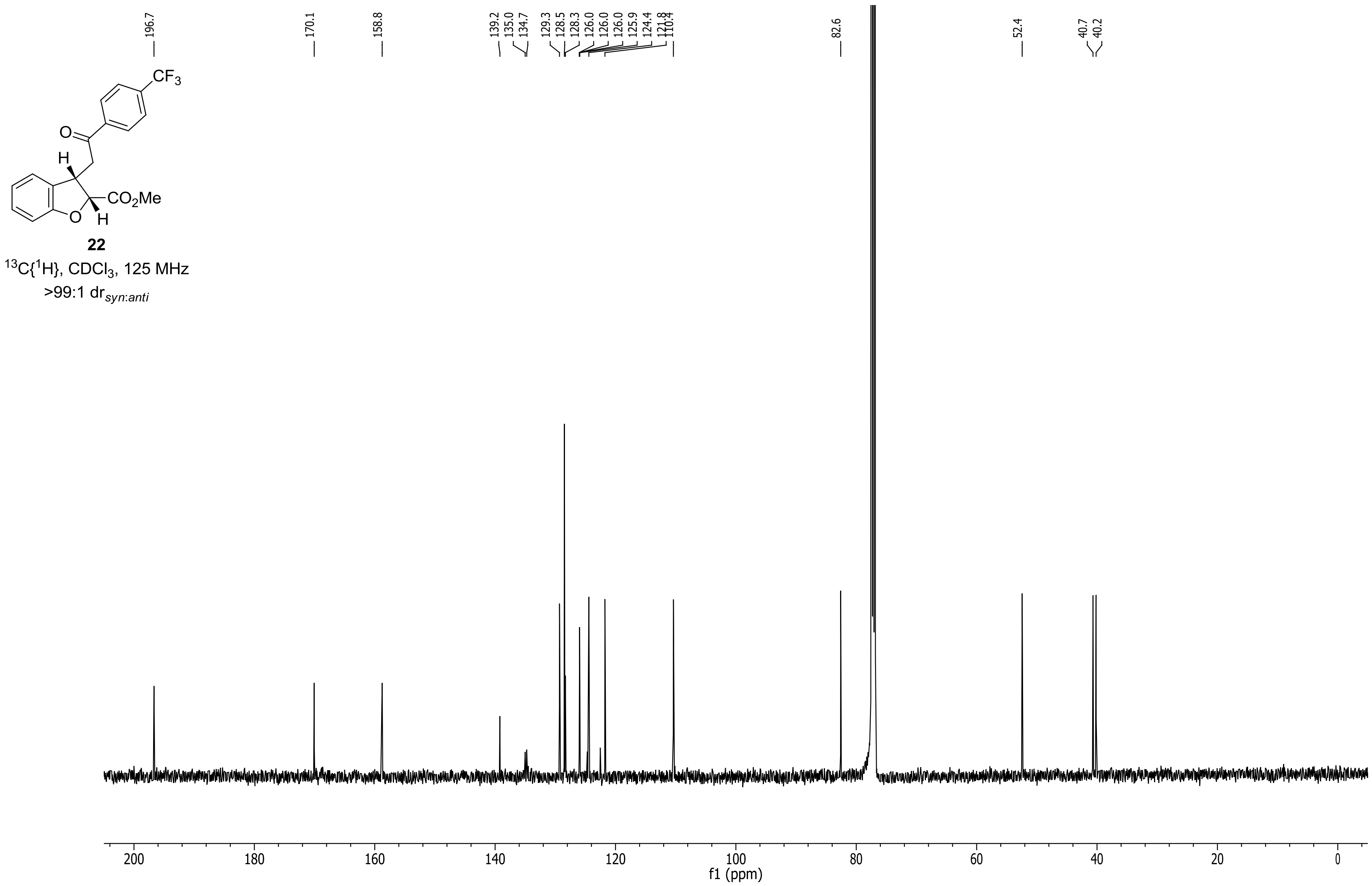

22

$^{13}C\{^{1}H\}$, $CDCl_3$, 125 MHz

>99:1 $dr_{syn:anti}$

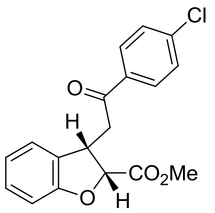

23

^{1}H, CDCl$_3$, 400 MHz

>99:1 dr$_{syn:anti}$

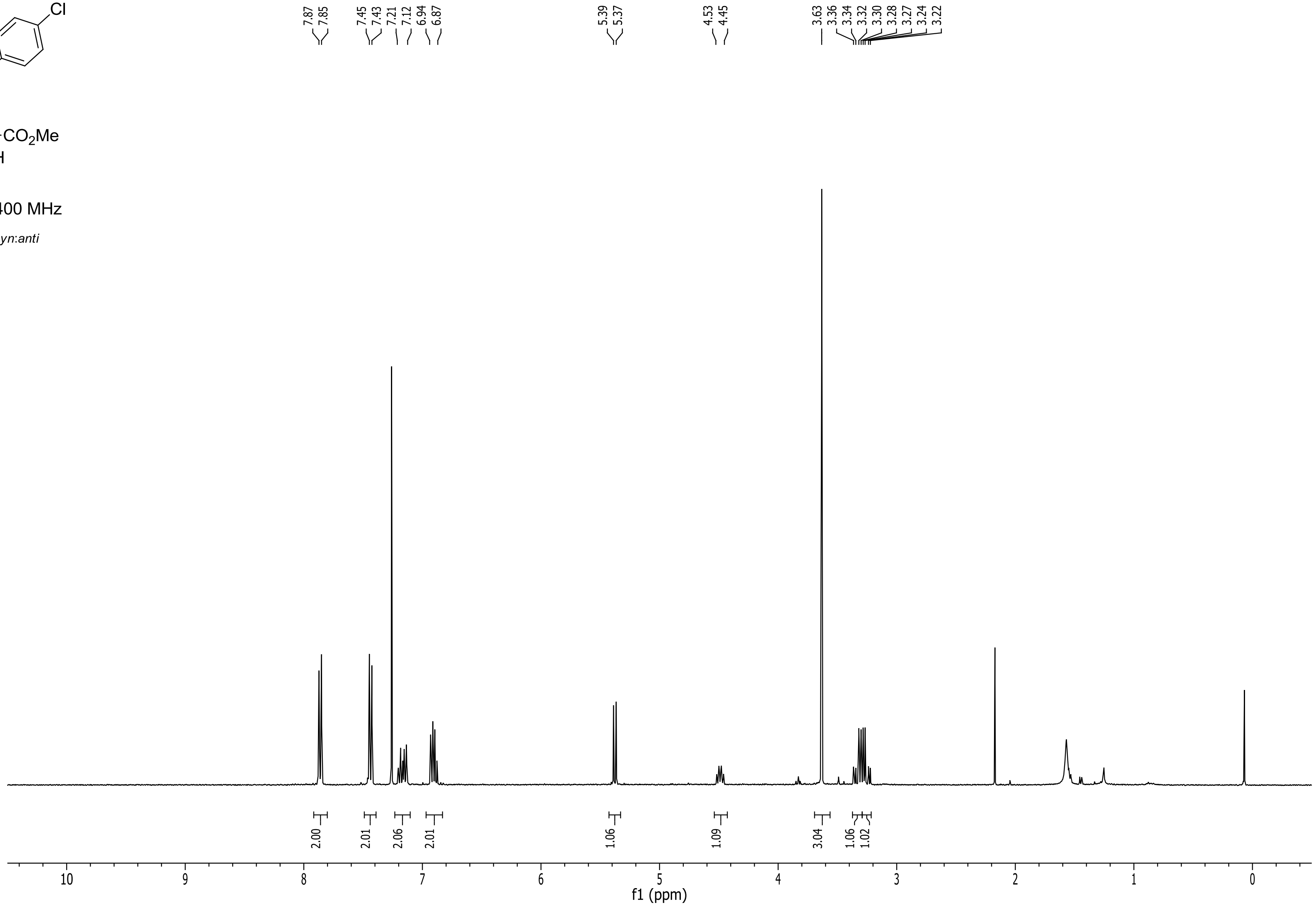

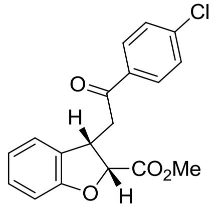

23

$^{13}C\{^{1}H\}$, $CDCl_3$, 100 MHz

>99:1 $dr_{syn:anti}$

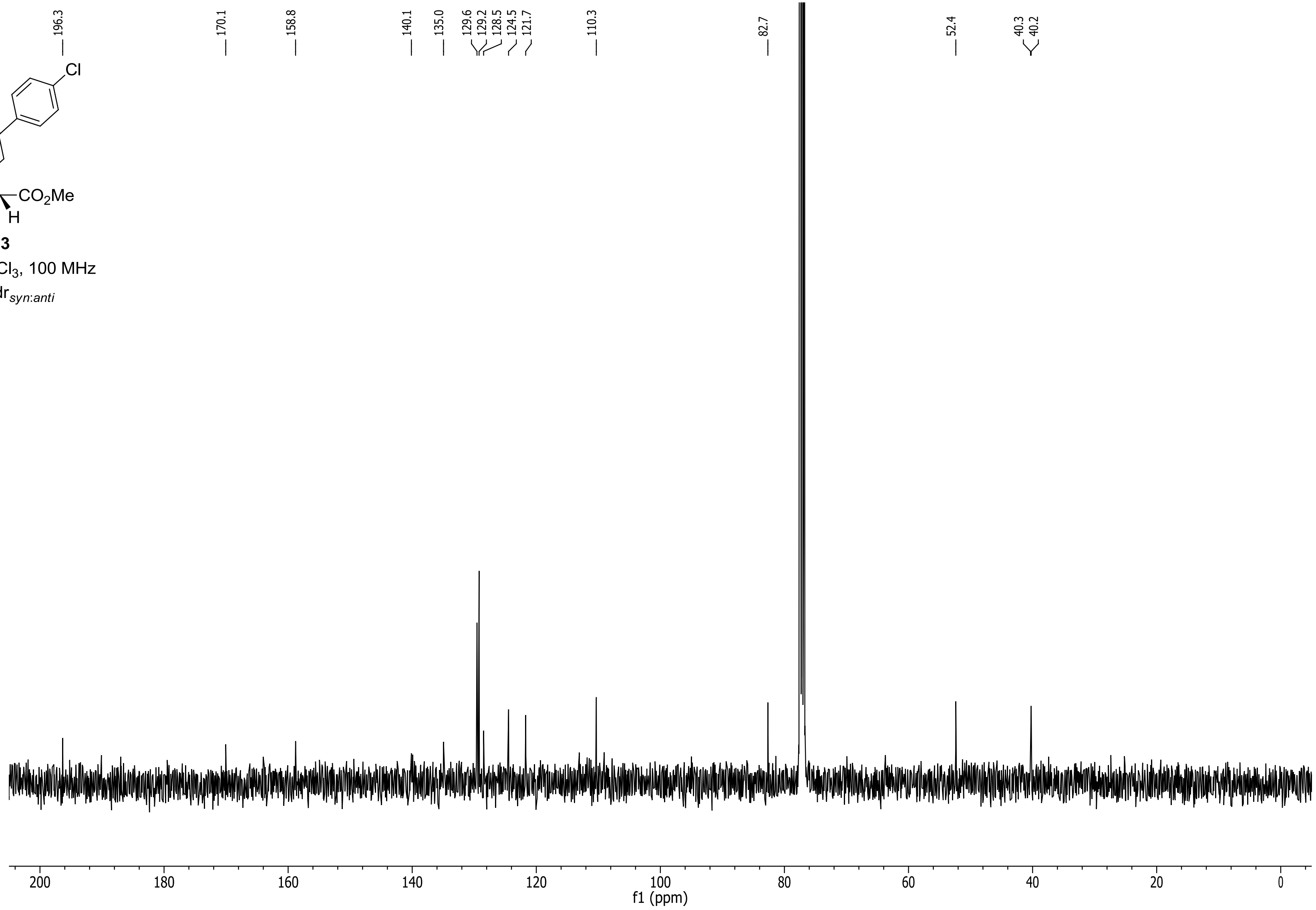

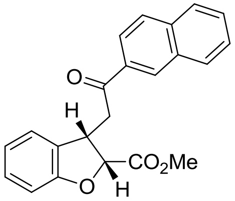

24

^1H, $CDCl_3$, 300 MHz

>99:1 dr$_{syn:anti}$

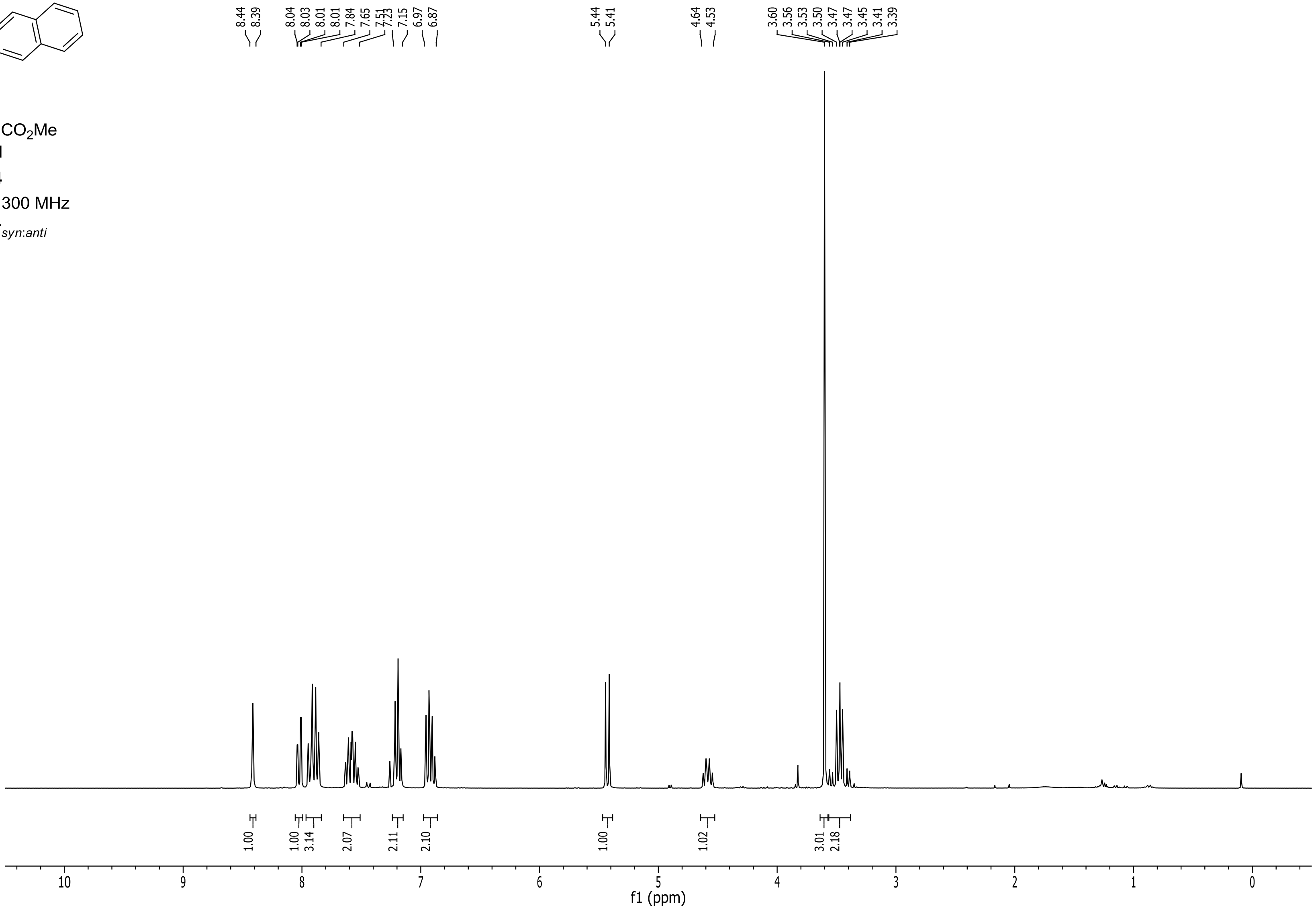

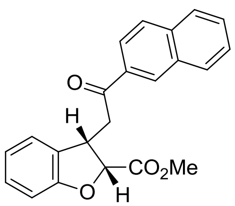

24

$^{13}C\{^{1}H\}$, $CDCl_3$, 75 MHz

>99:1 $dr_{syn:anti}$

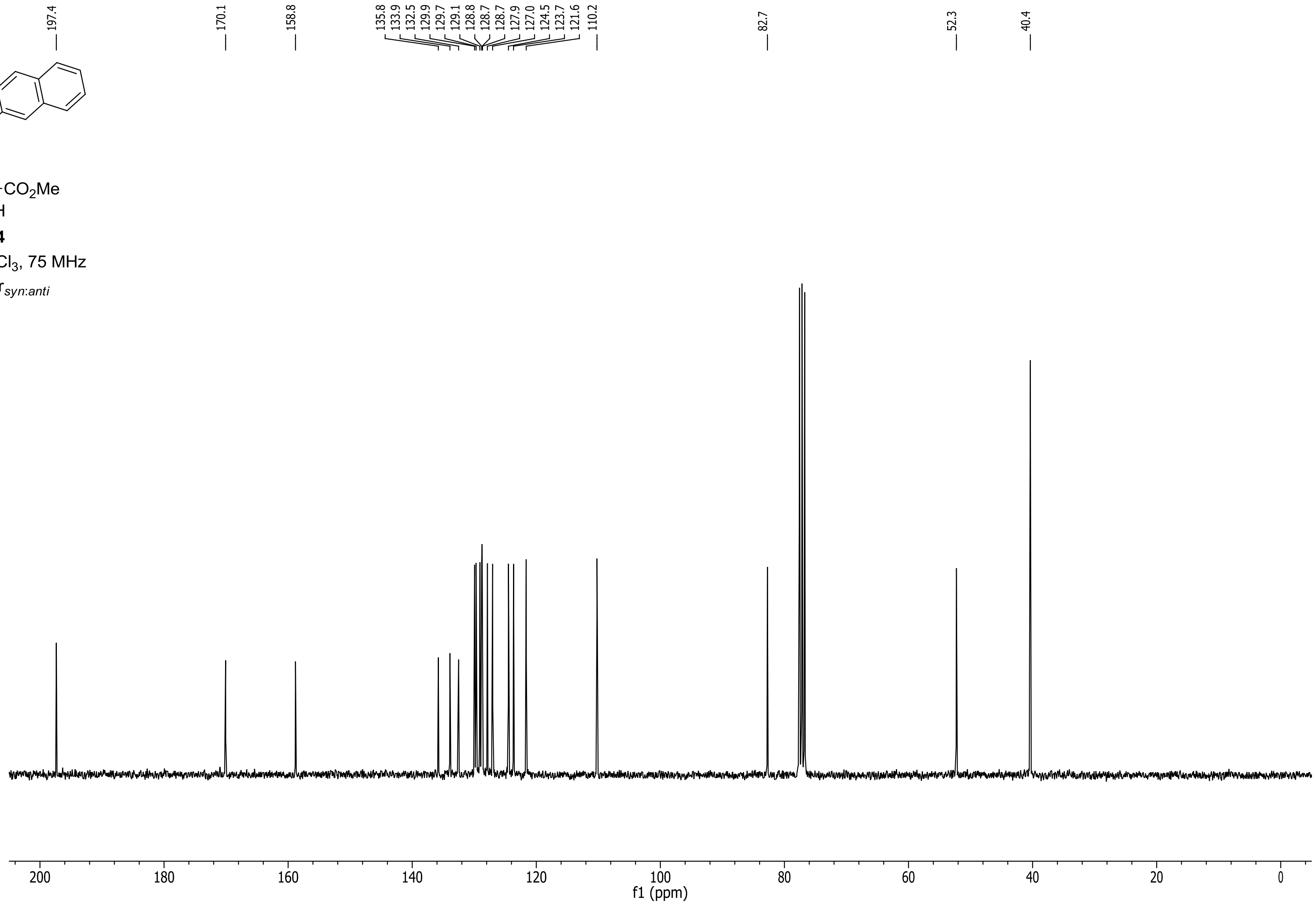

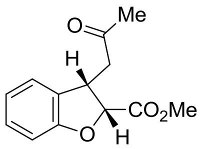

25

^{1}H, $CDCl_3$, 300 MHz

>99:1 $dr_{syn:anti}$

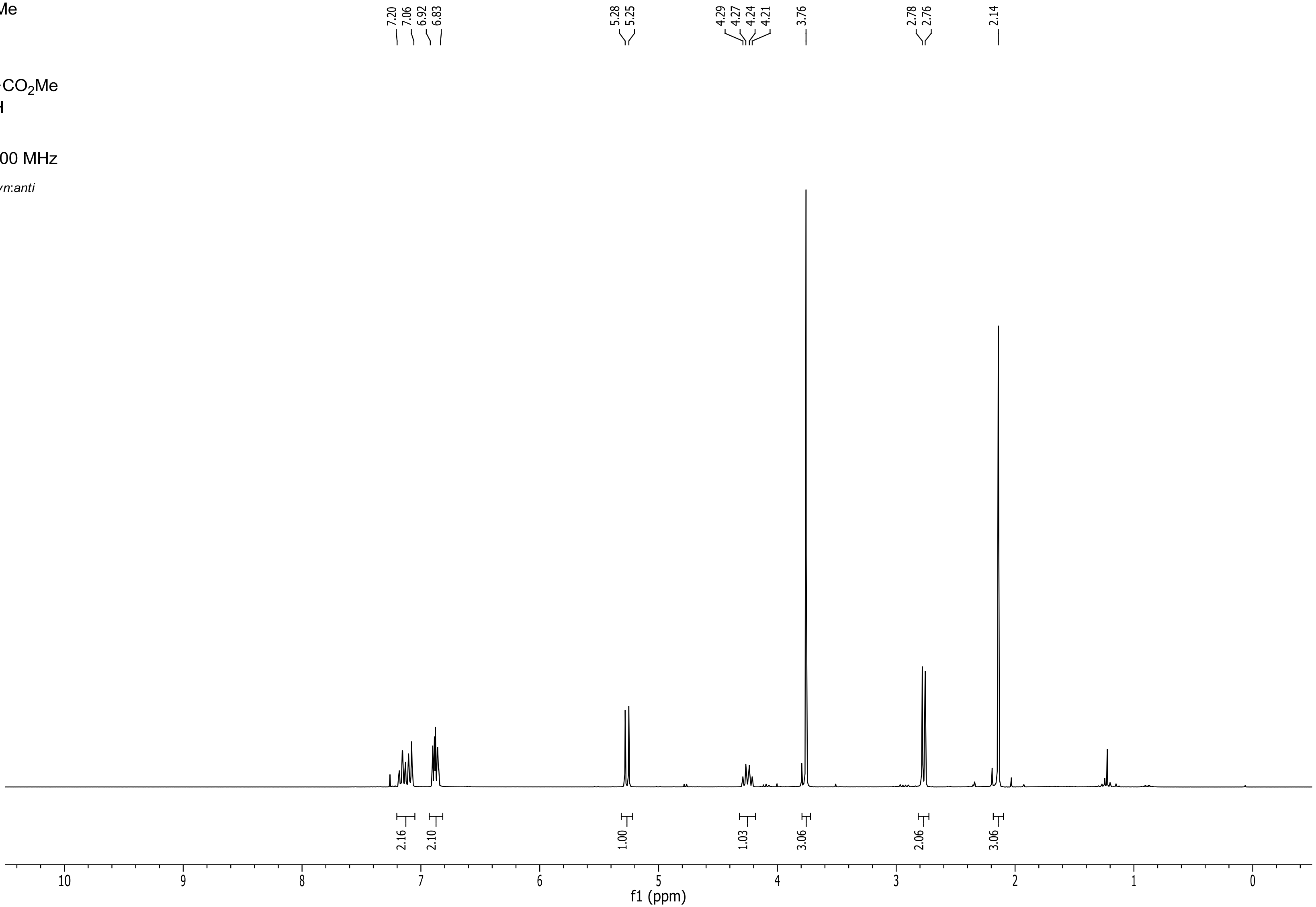

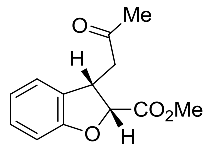

25

$^{13}C\{^1H\}$, $CDCl_3$, 75 MHz

>99:1 dr$_{syn:anti}$

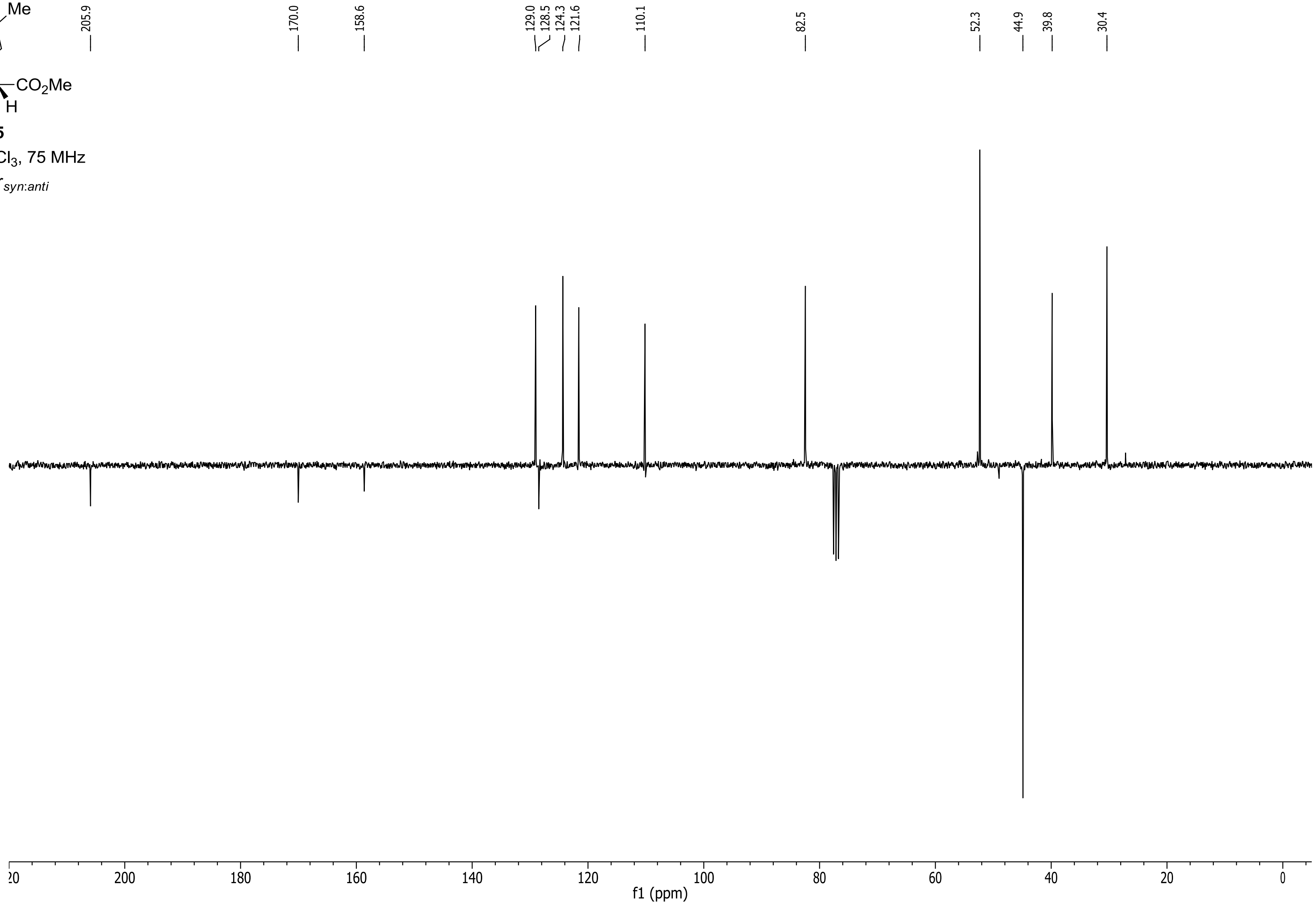

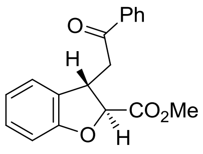

11

^{1}H, $CDCl_3$, 300 MHz

>1:99 $dr_{syn:anti}$

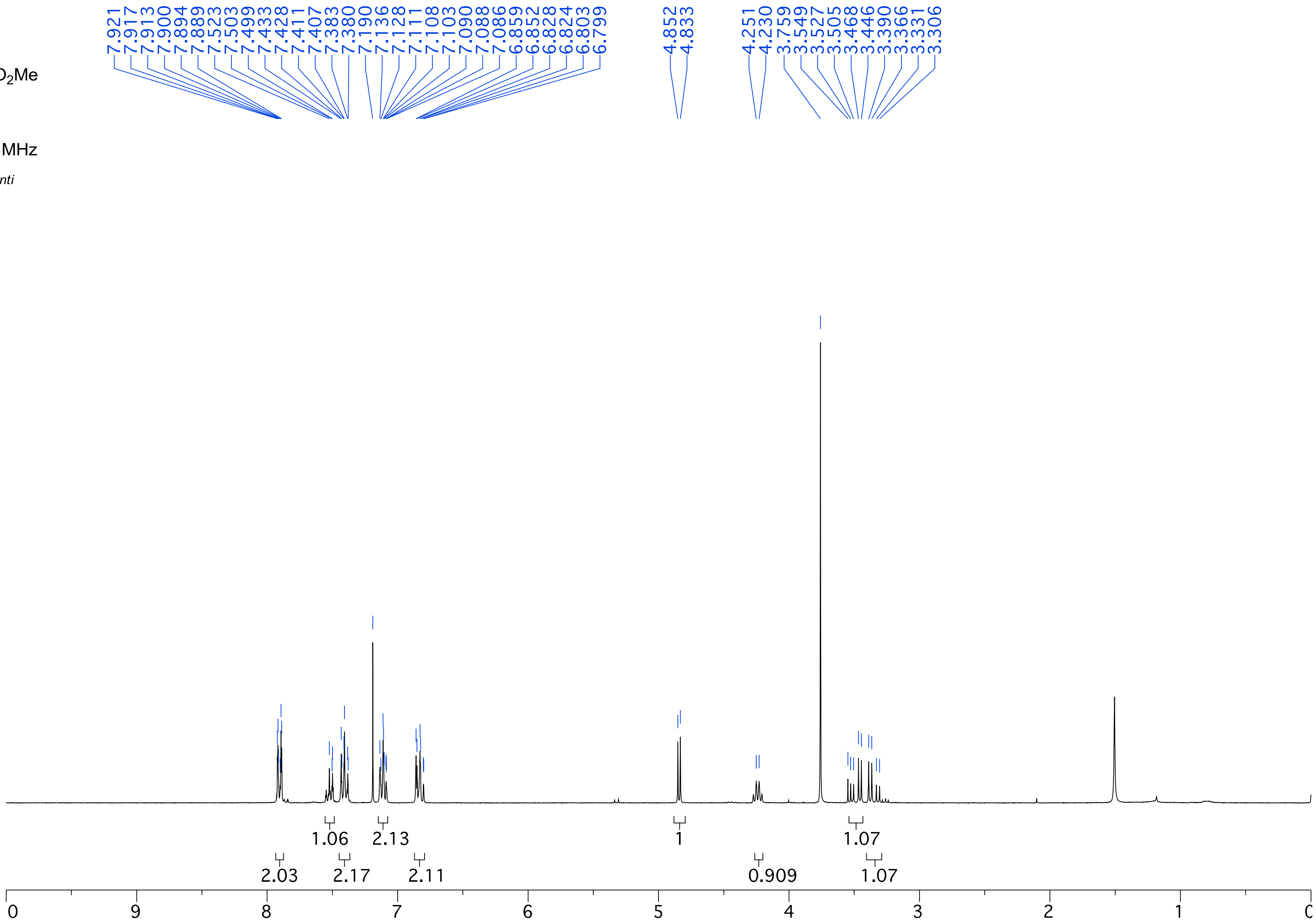

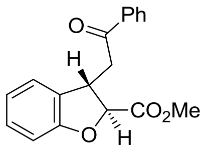

11

$^{13}C\{^1H\}$, $CDCl_3$, 100 MHz

>1:99 $dr_{syn:anti}$

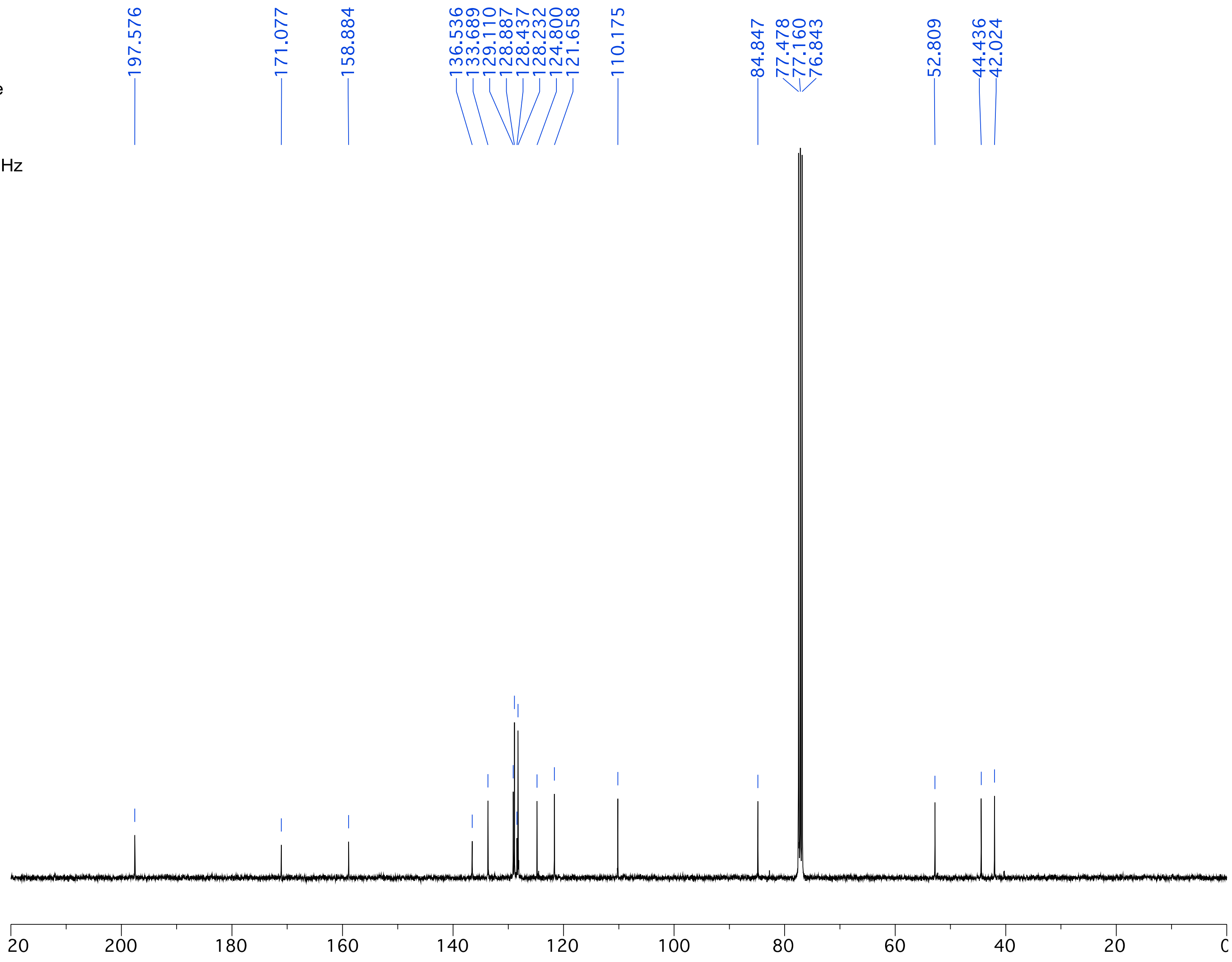

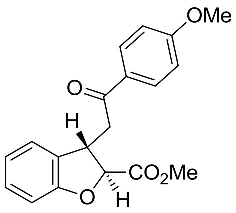

26

^1H, $CDCl_3$, 300 MHz

14:86 $dr_{syn:anti}$

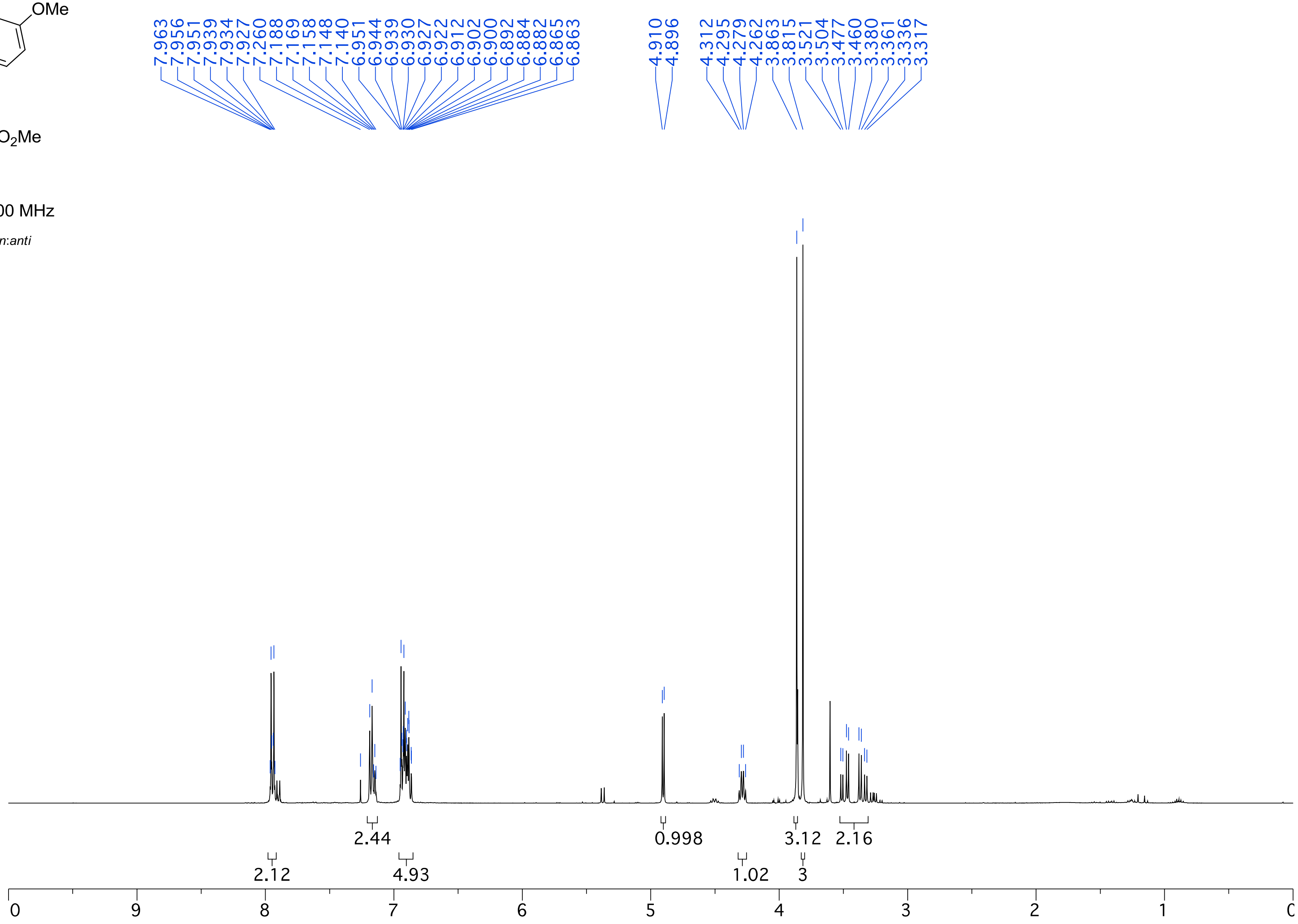

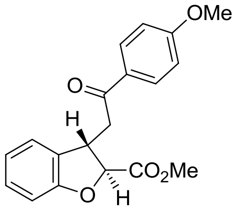

26

$^{13}C\{^{1}H\}$, $CDCl_3$, 75 MHz

14:86 $dr_{syn:anti}$

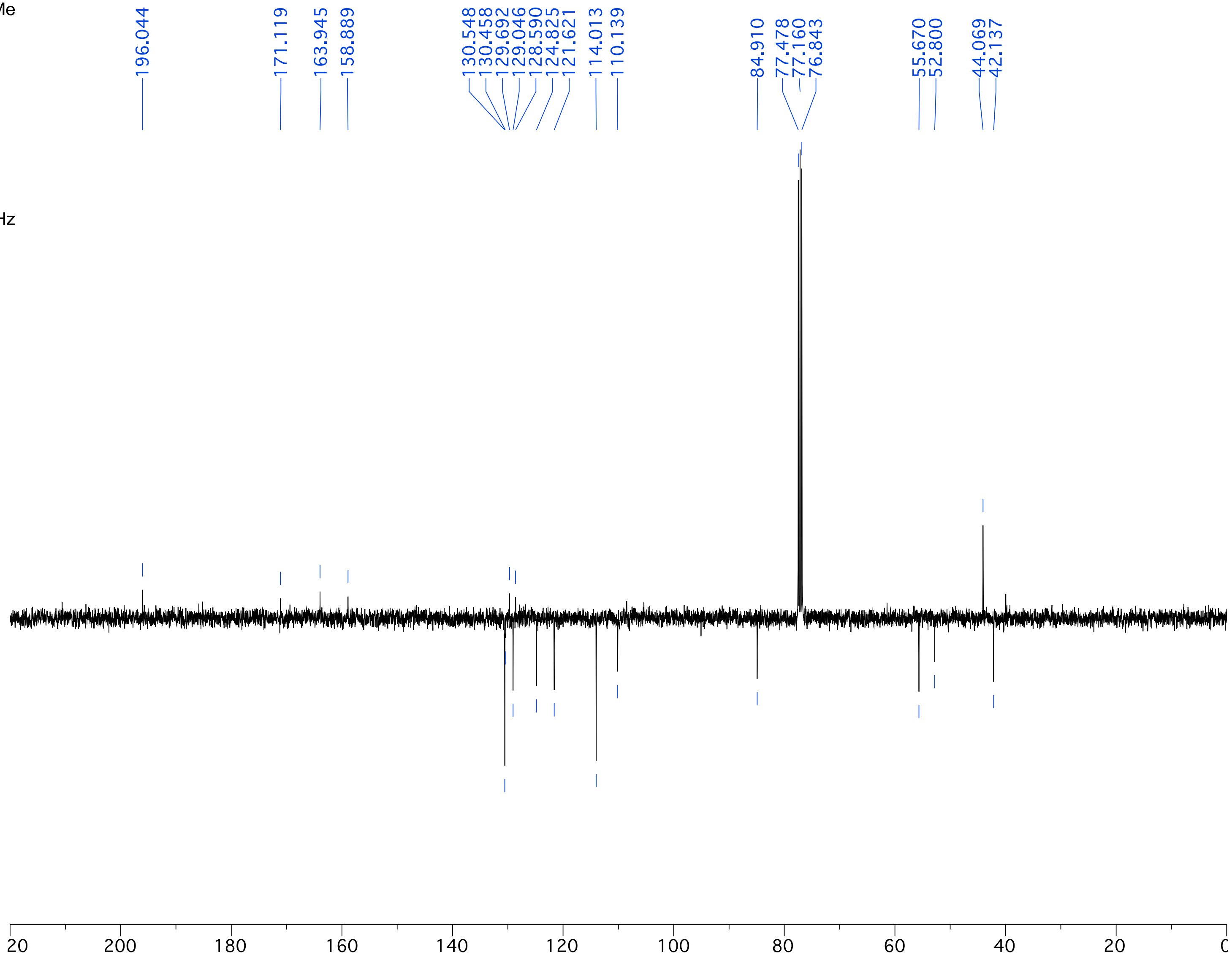

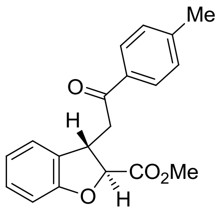

27

^{1}H, $CDCl_3$, 400 MHz

>1:99 $dr_{syn:anti}$

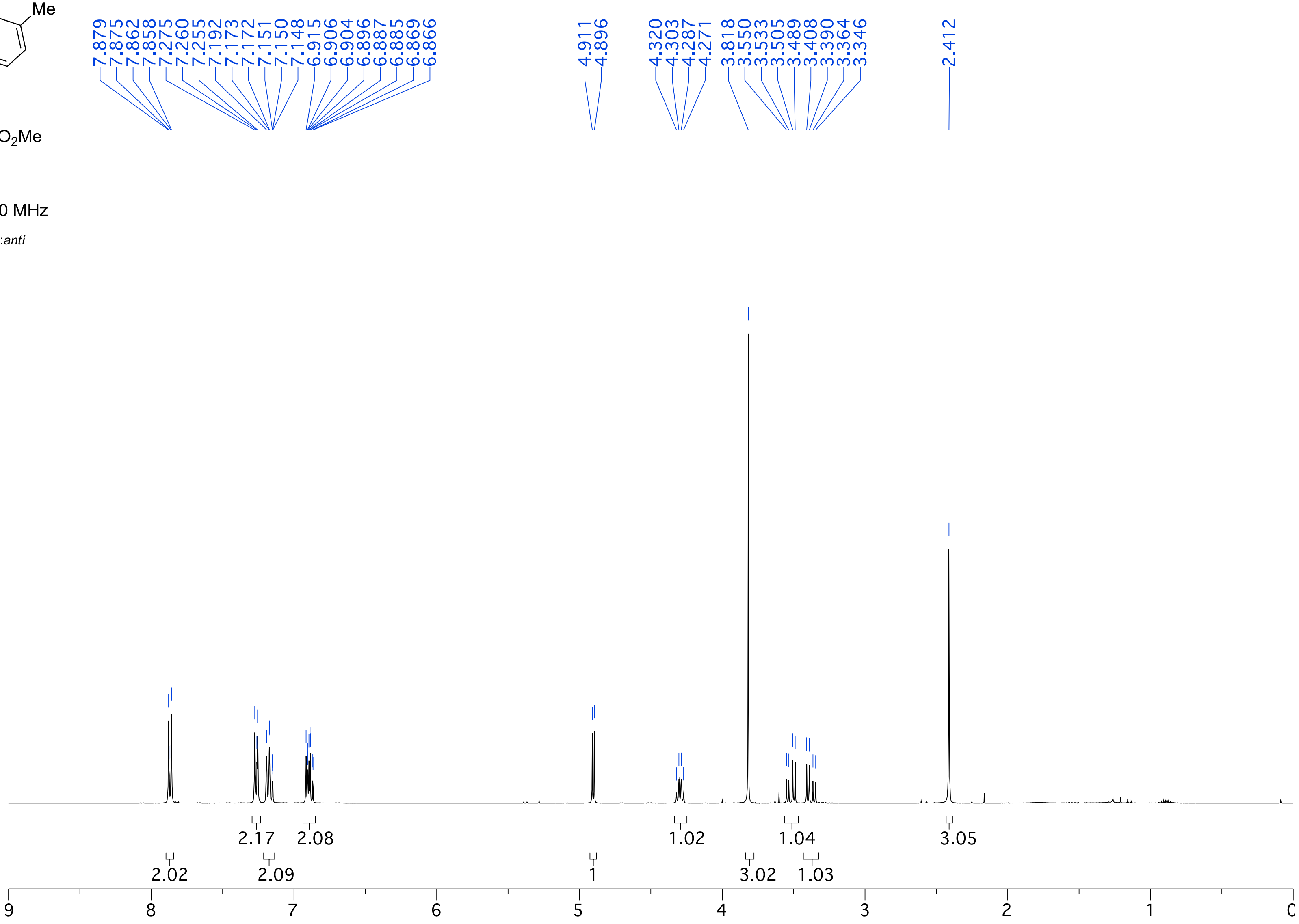

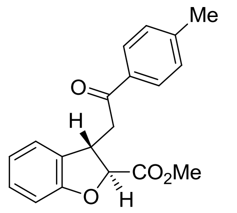

27

$^{13}C\{^{1}H\}$, $CDCl_3$, 100 MHz

>1:99 $dr_{syn:anti}$

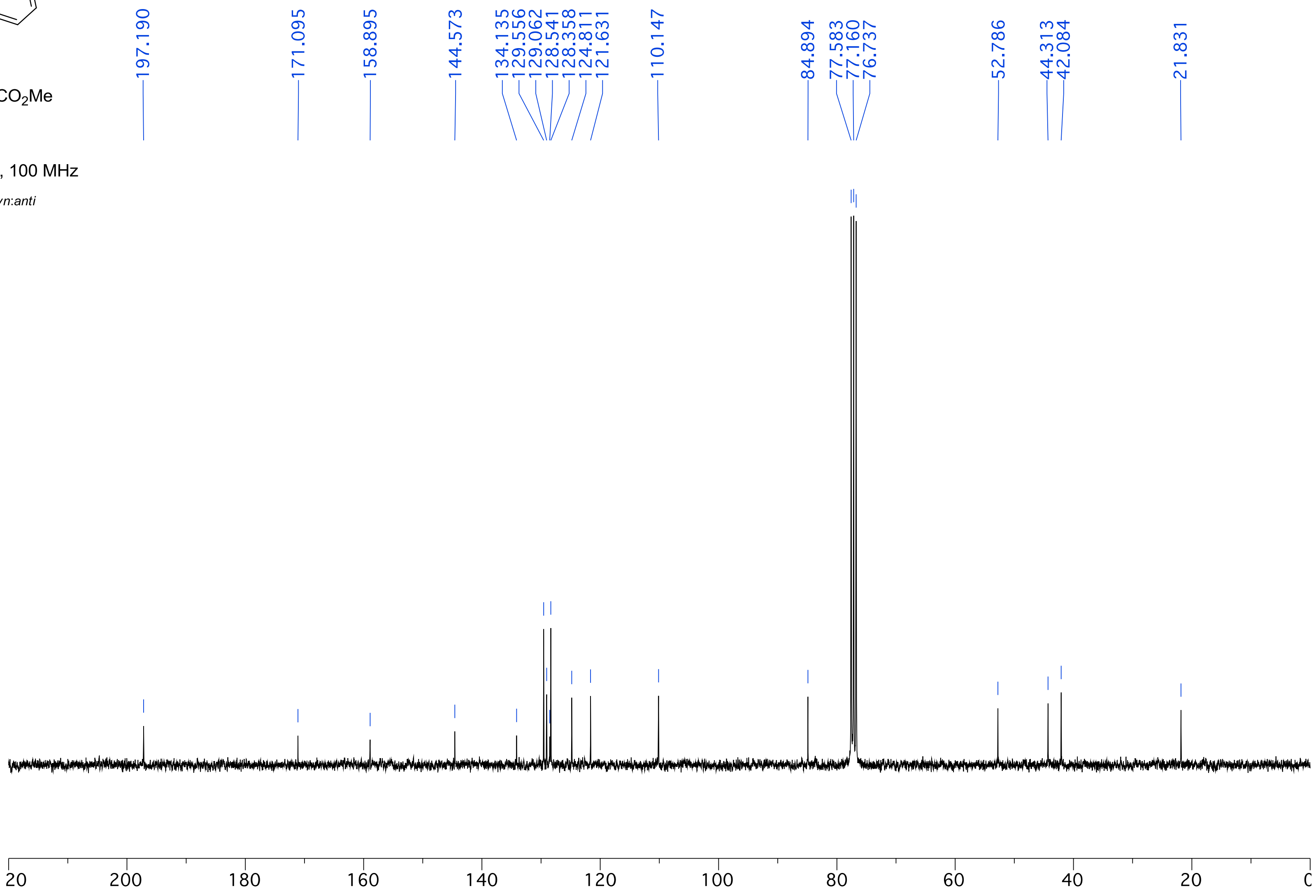

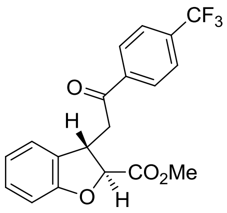

28

^1H, $CDCl_3$, 300 MHz

13:87 $dr_{syn:anti}$

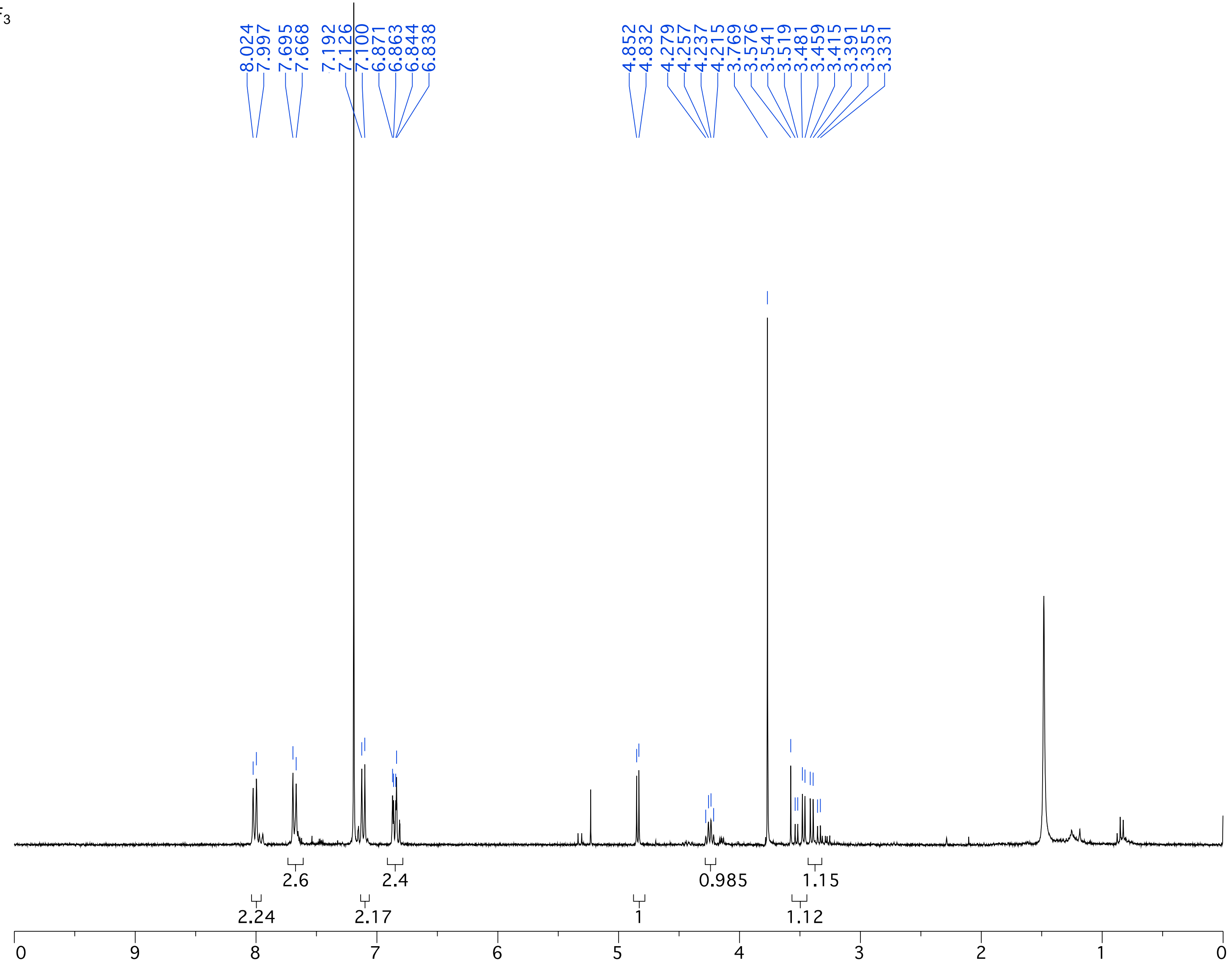

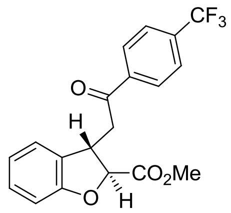

28

$^{13}C\{^{1}H\}$, $CDCl_3$, 125 MHz

13:87 $dr_{syn:anti}$

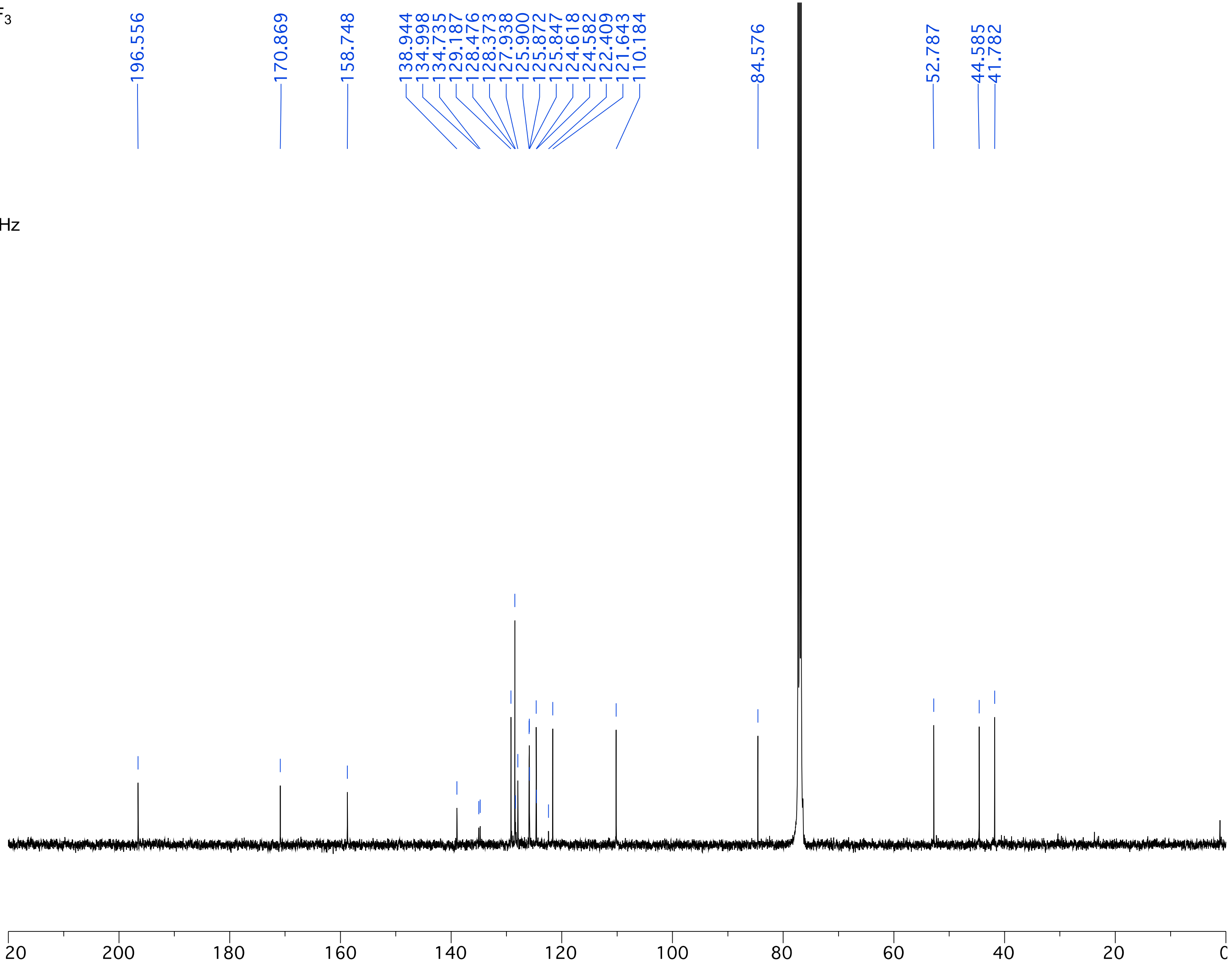

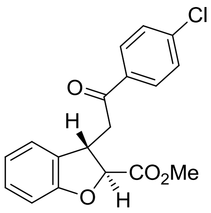

29

^{1}H, $CDCl_3$, 300 MHz

>1:99 $dr_{syn:anti}$

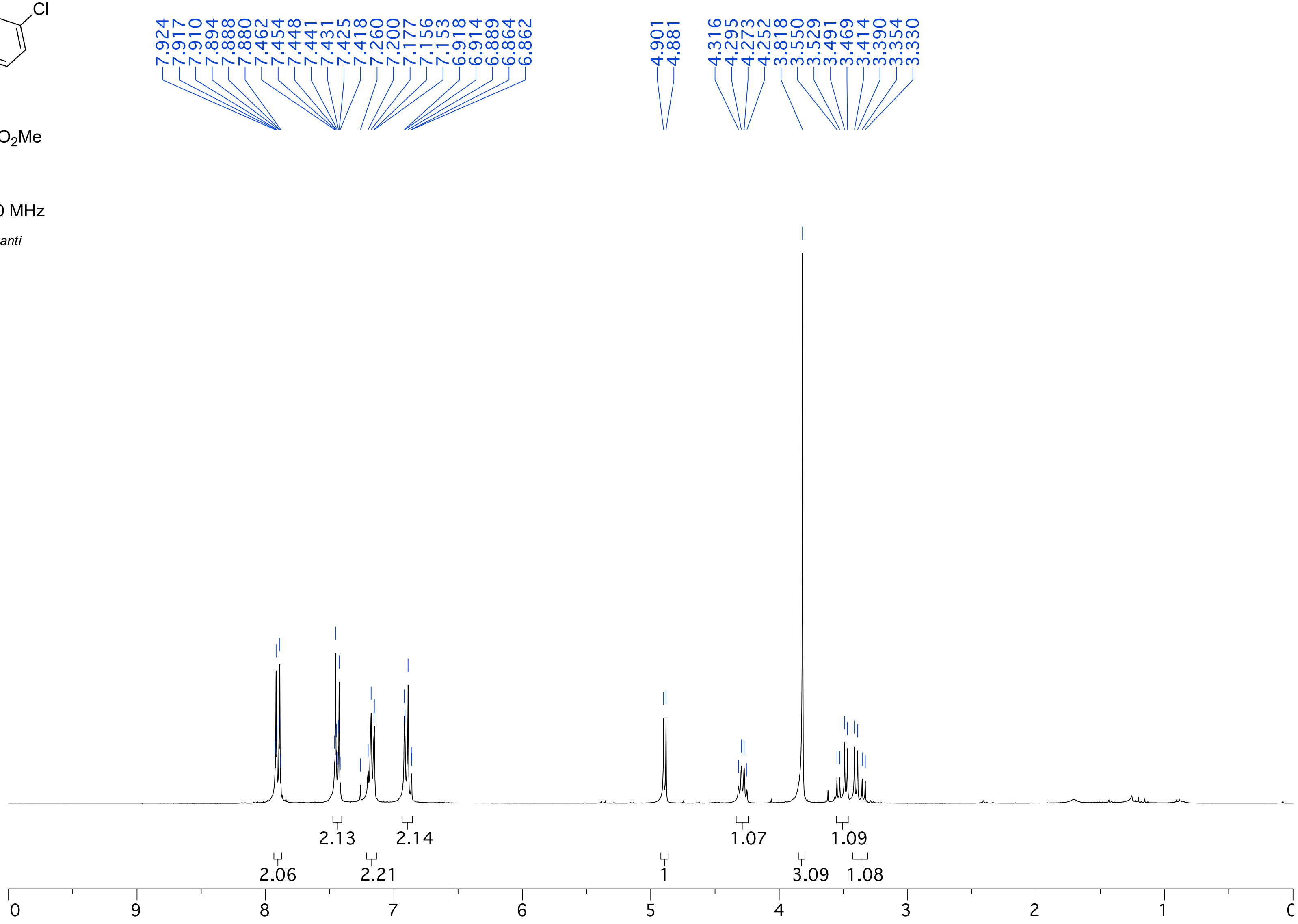

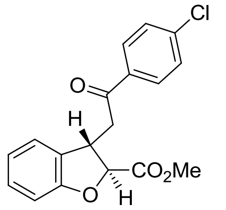

29

$^{13}C\{^1H\}$, $CDCl_3$, 75 MHz

>1:99 $dr_{syn:anti}$

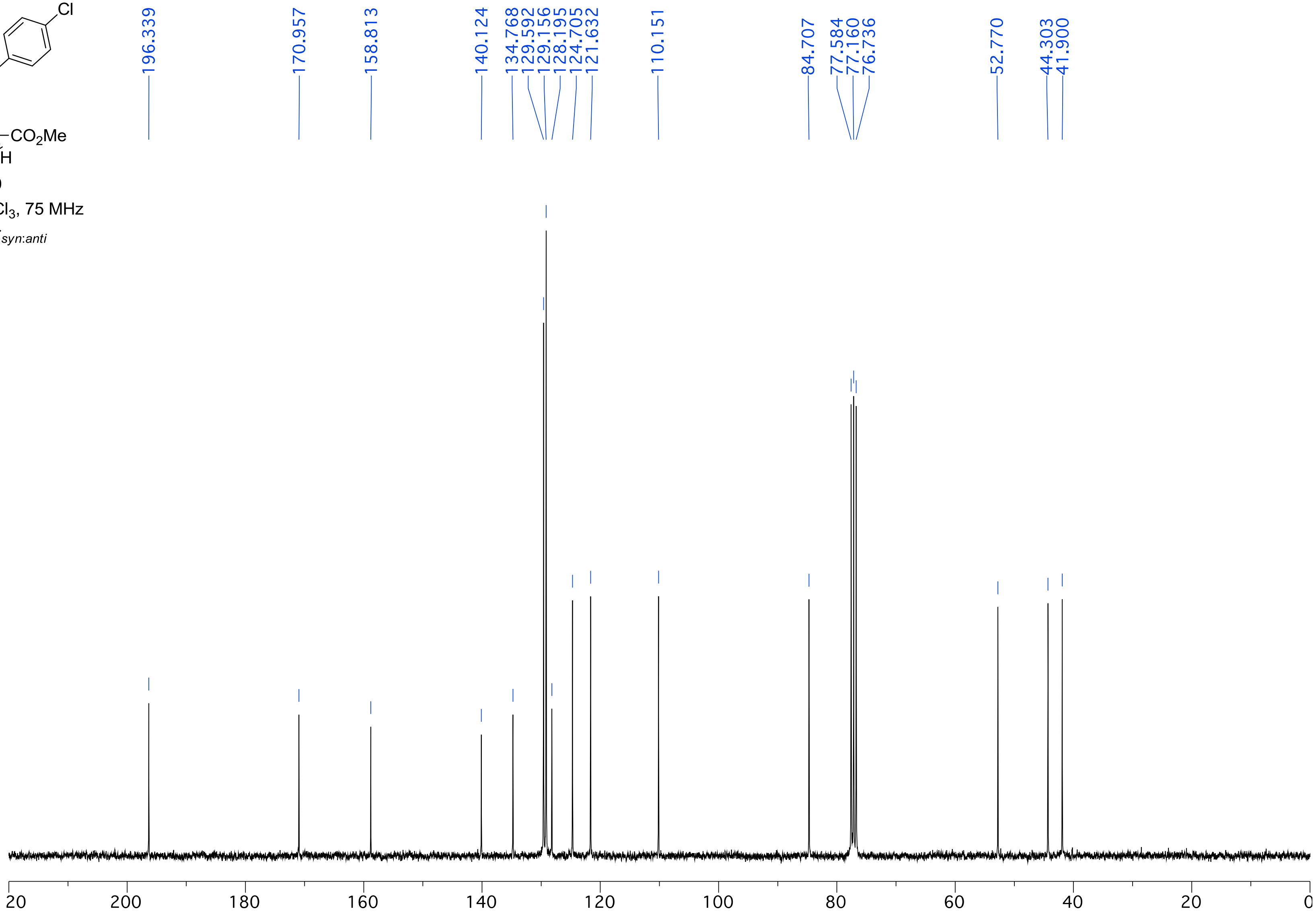

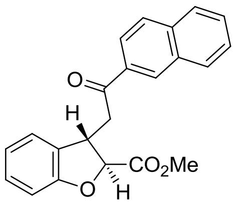

30

^1H, $CDCl_3$, 300 MHz

9:91 $dr_{syn:anti}$

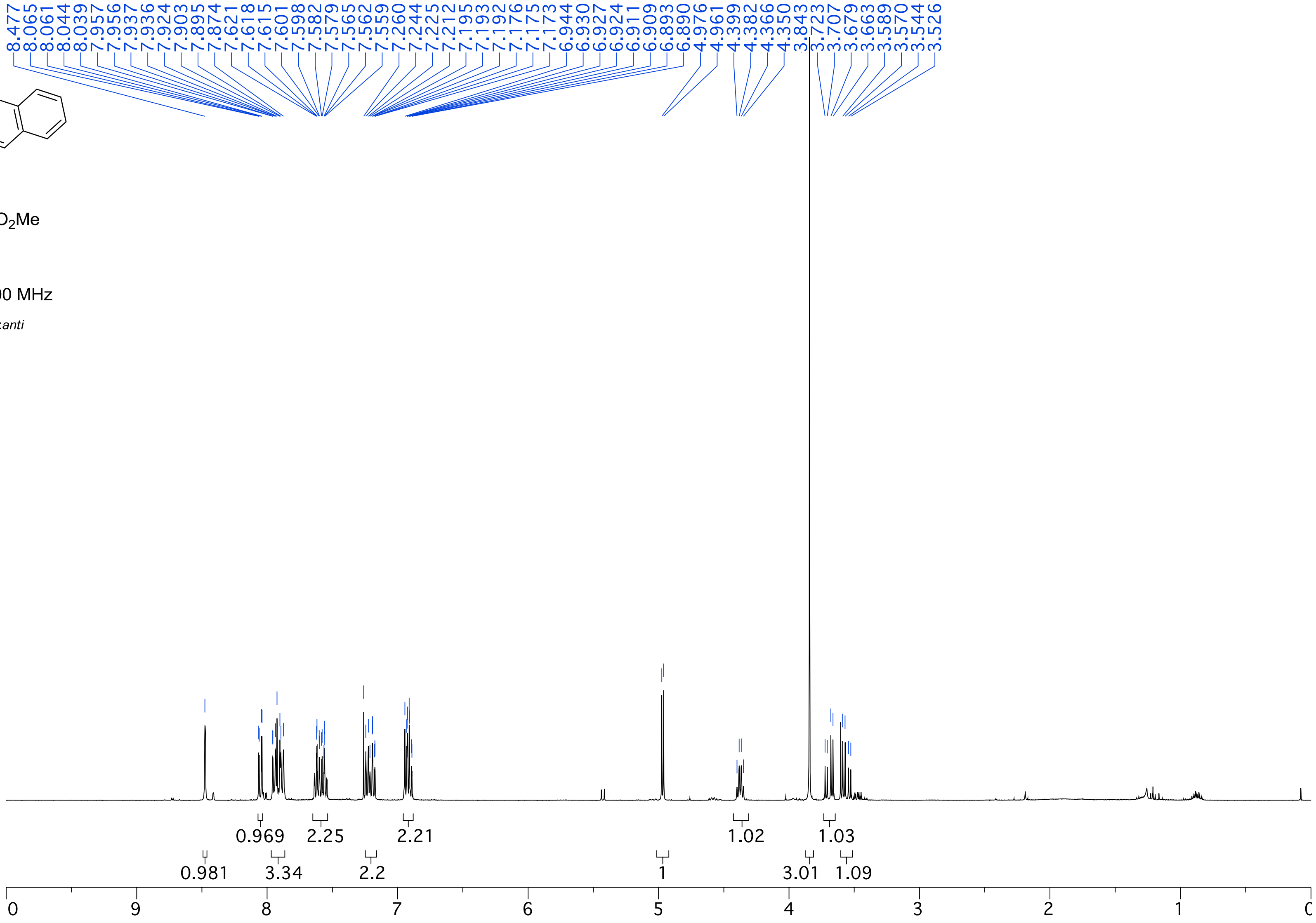

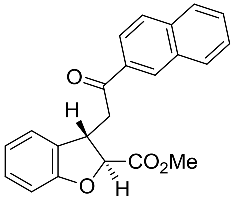

30

$^{13}C\{^{1}H\}$, $CDCl_3$, 75 MHz

9:91 dr$_{syn:anti}$

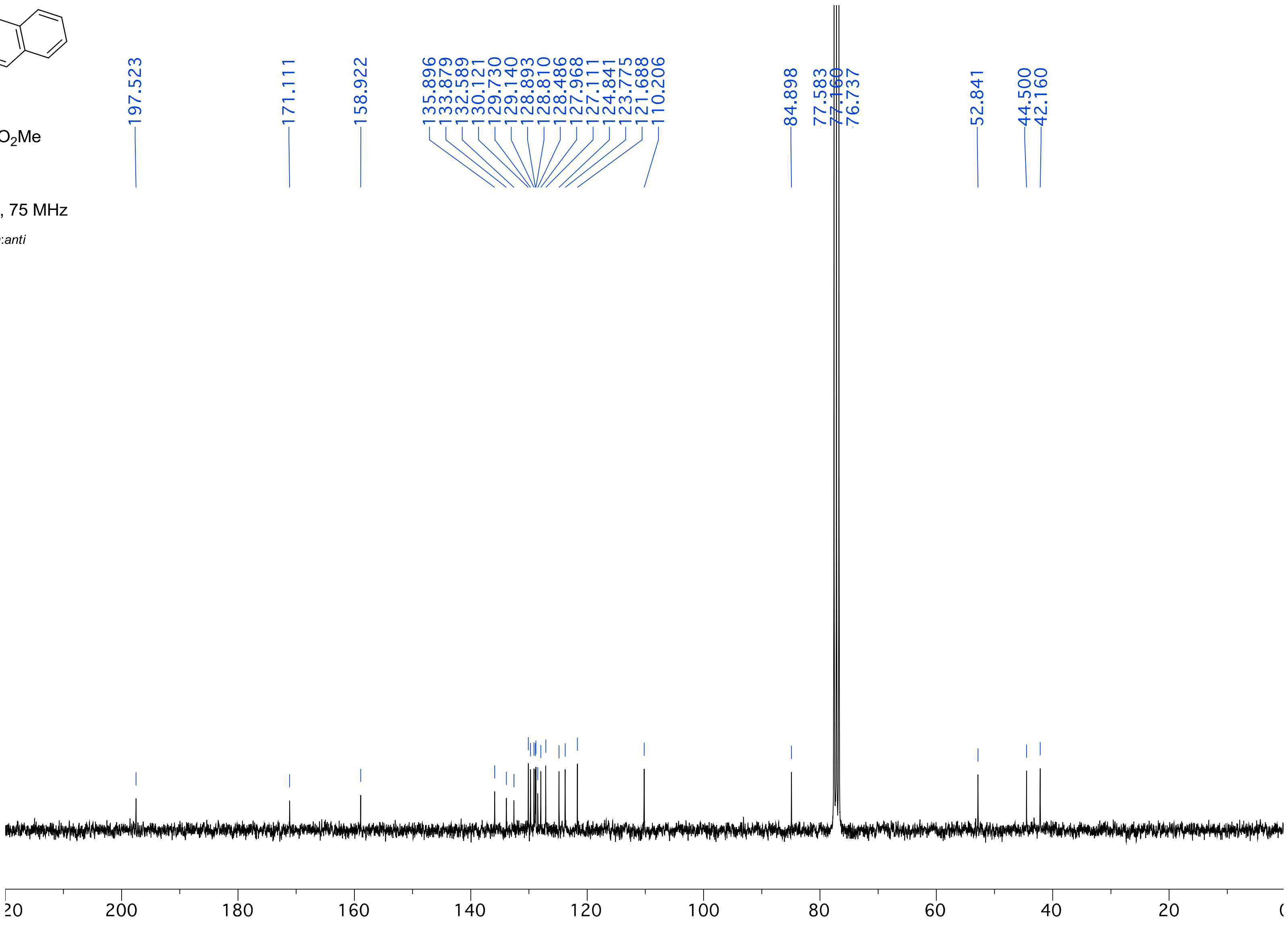

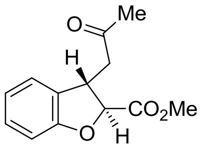

31

^{1}H, $CDCl_3$, 400 MHz

22:78 dr$_{syn:anti}$

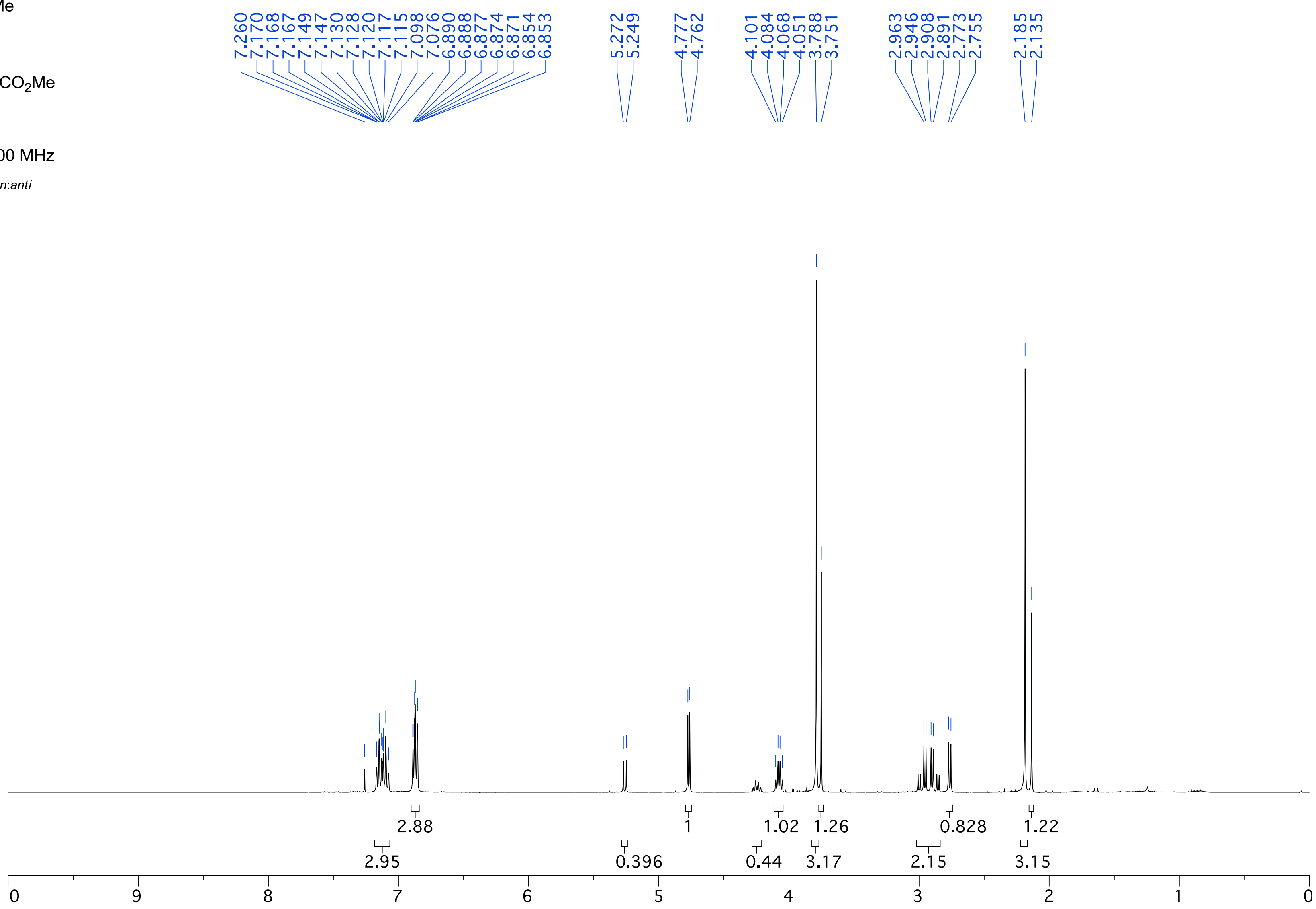

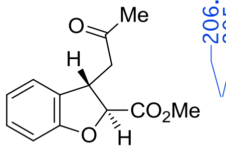

31

$^{13}C\{^{1}H\}$, $CDCl_3$, 100 MHz

22:78 $dr_{syn:anti}$

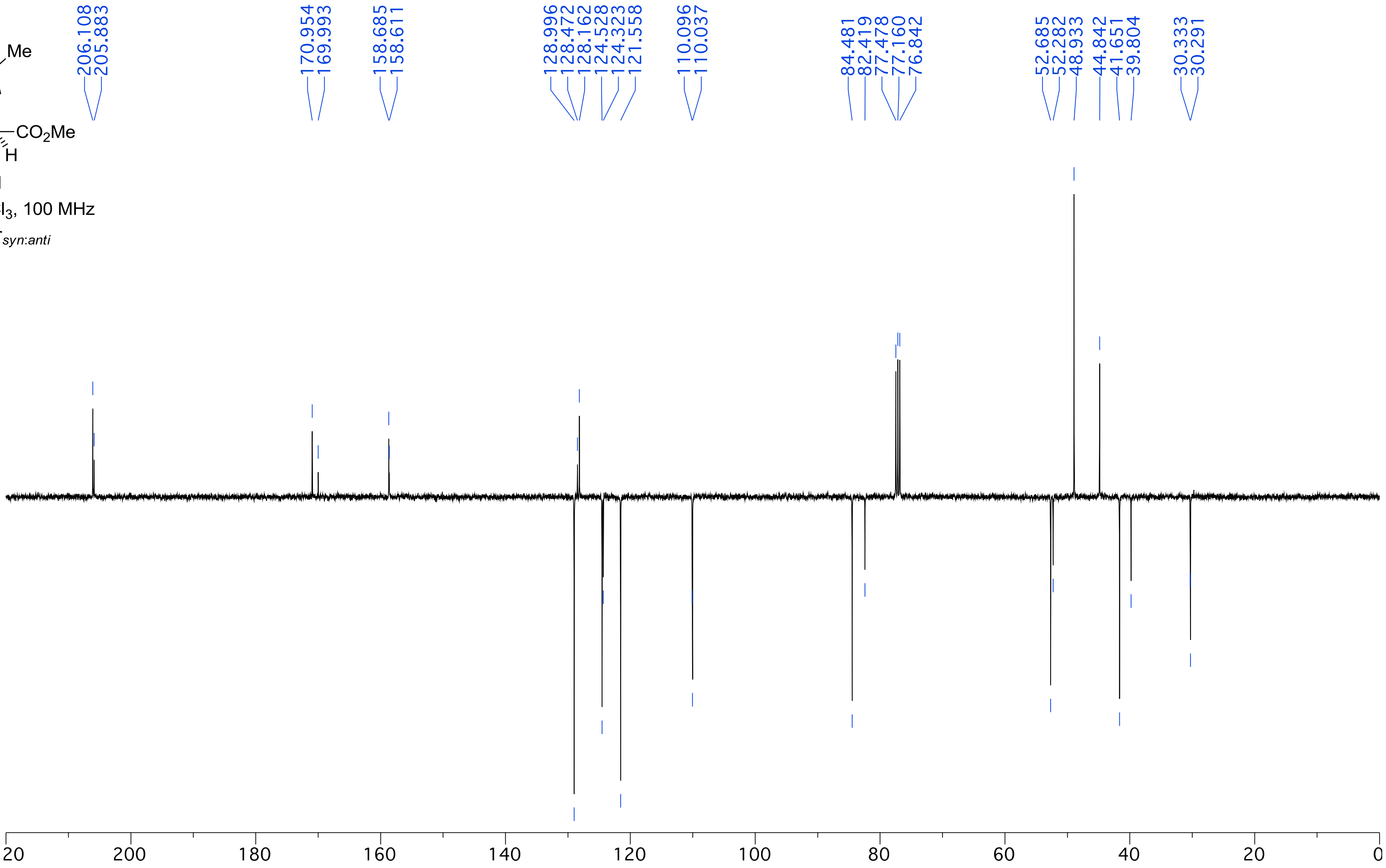

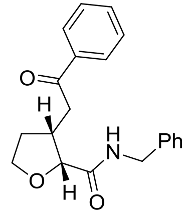

32

^{1}H, $CDCl_3$, 300 MHz

>99:1 $dr_{syn:anti}$

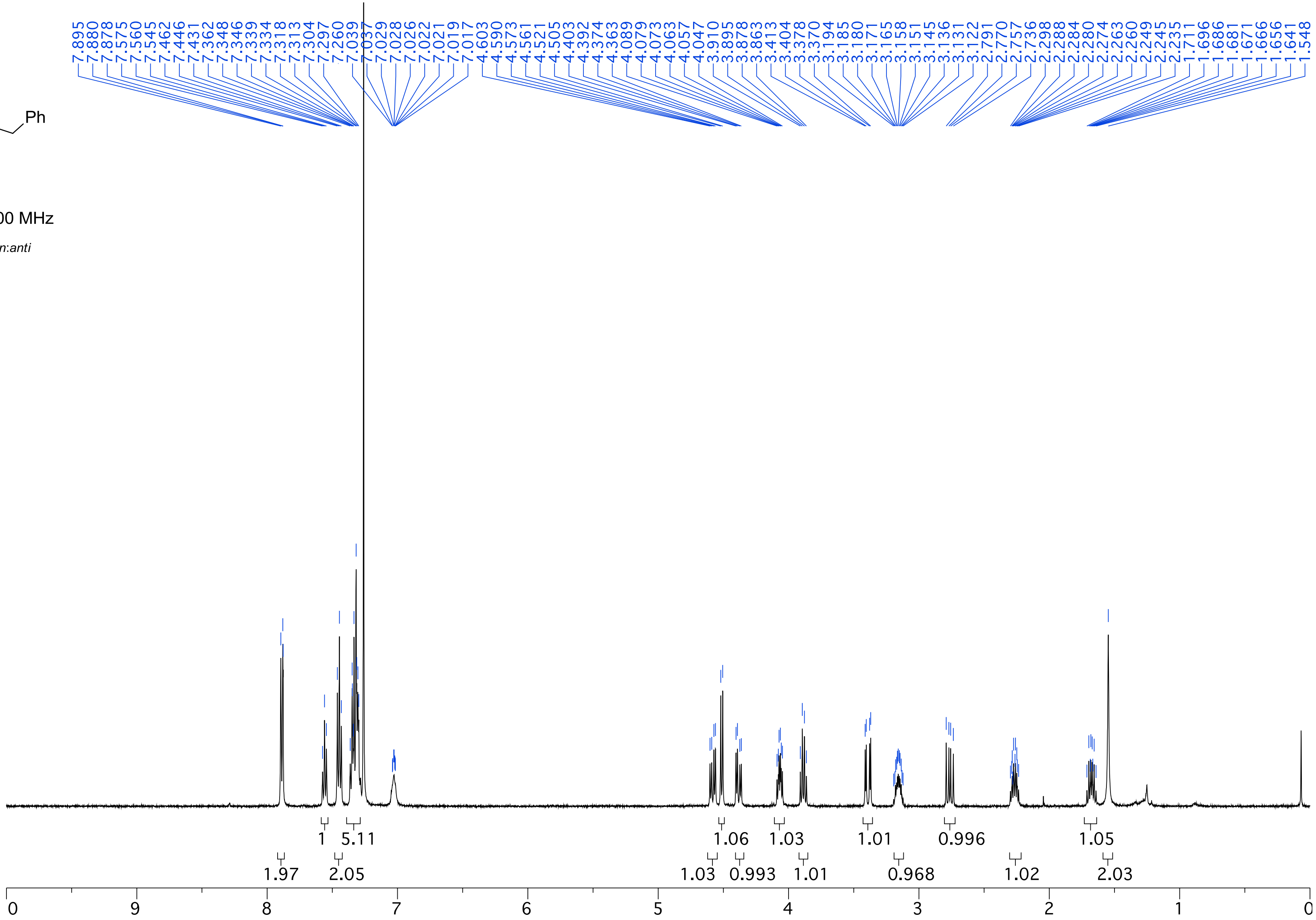

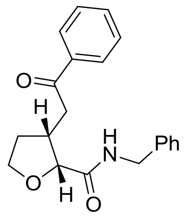

32

$^{13}C\{^{1}H\}$, $CDCl_3$, 75 MHz

>99:1 $dr_{syn:anti}$

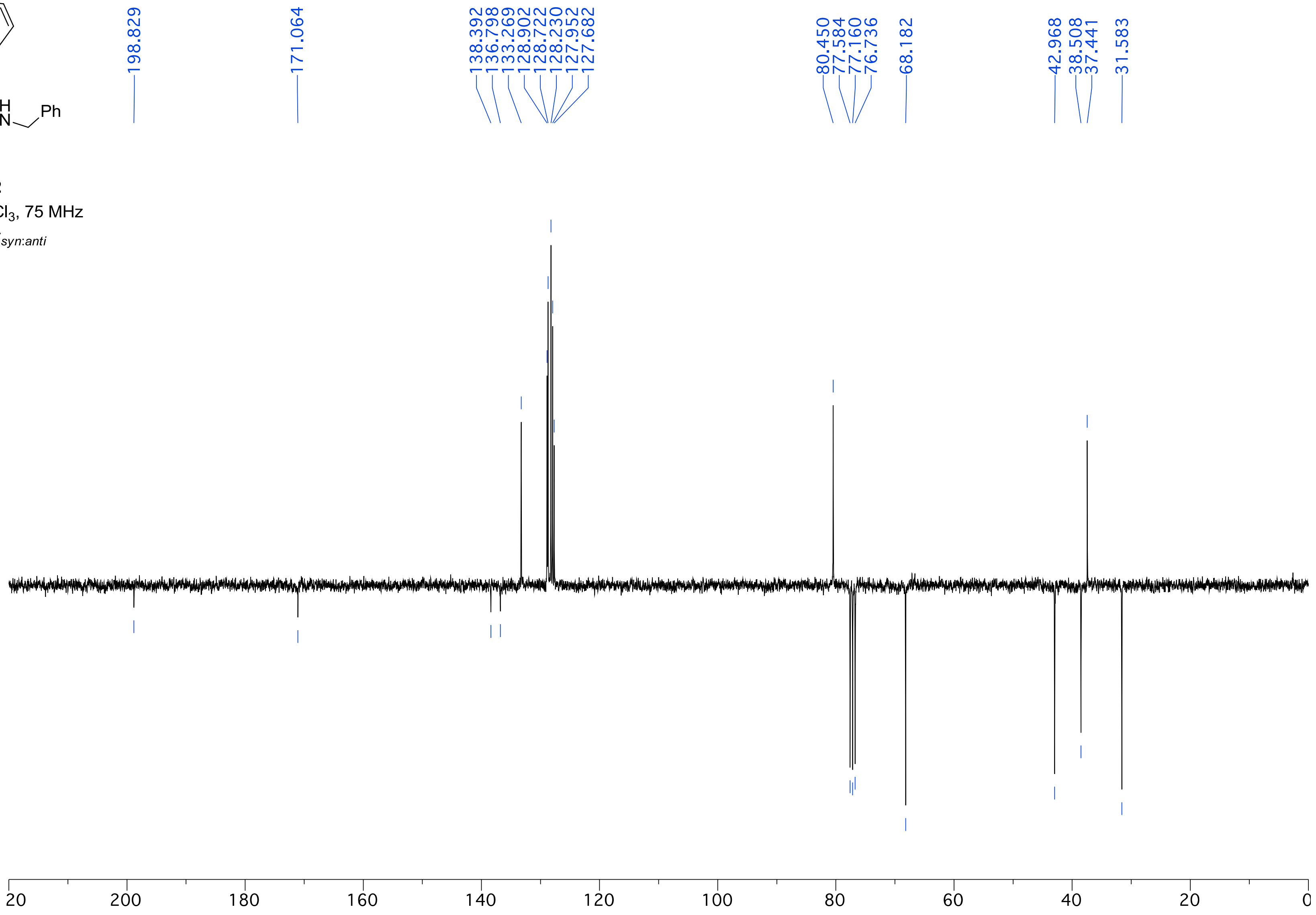

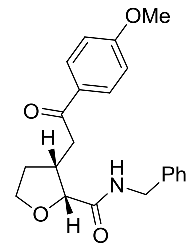

33

^{1}H, $CDCl_3$, 400 MHz

>99:1 $dr_{syn:anti}$

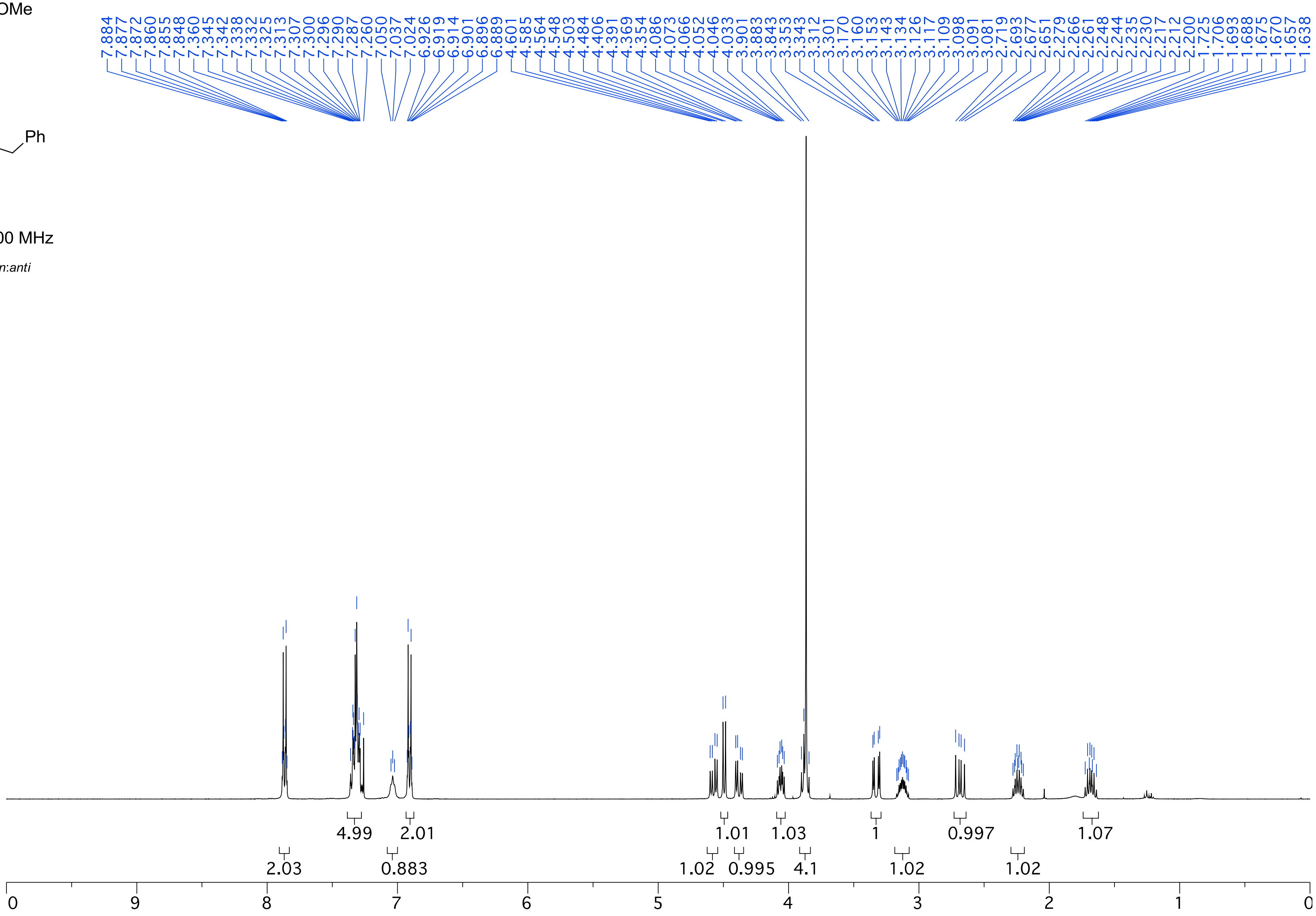

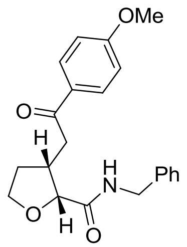

33

$^{13}C\{^{1}H\}$, $CDCl_3$, 75 MHz

>99:1 $dr_{syn:anti}$

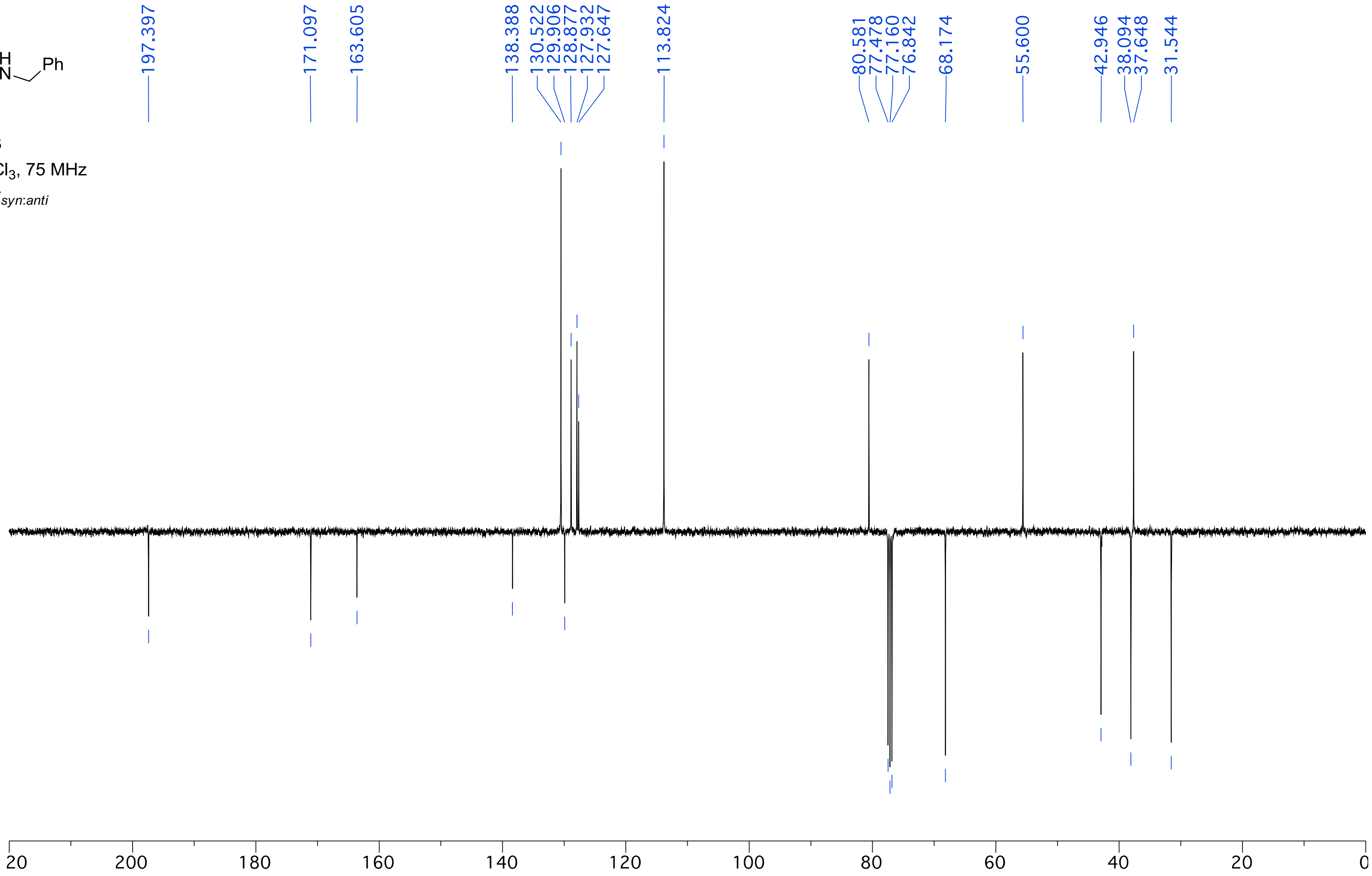

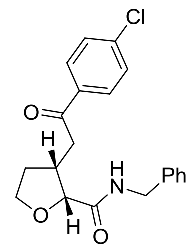

34

^{1}H, $CDCl_3$, 400 MHz

>99:1 dr$_{syn:anti}$

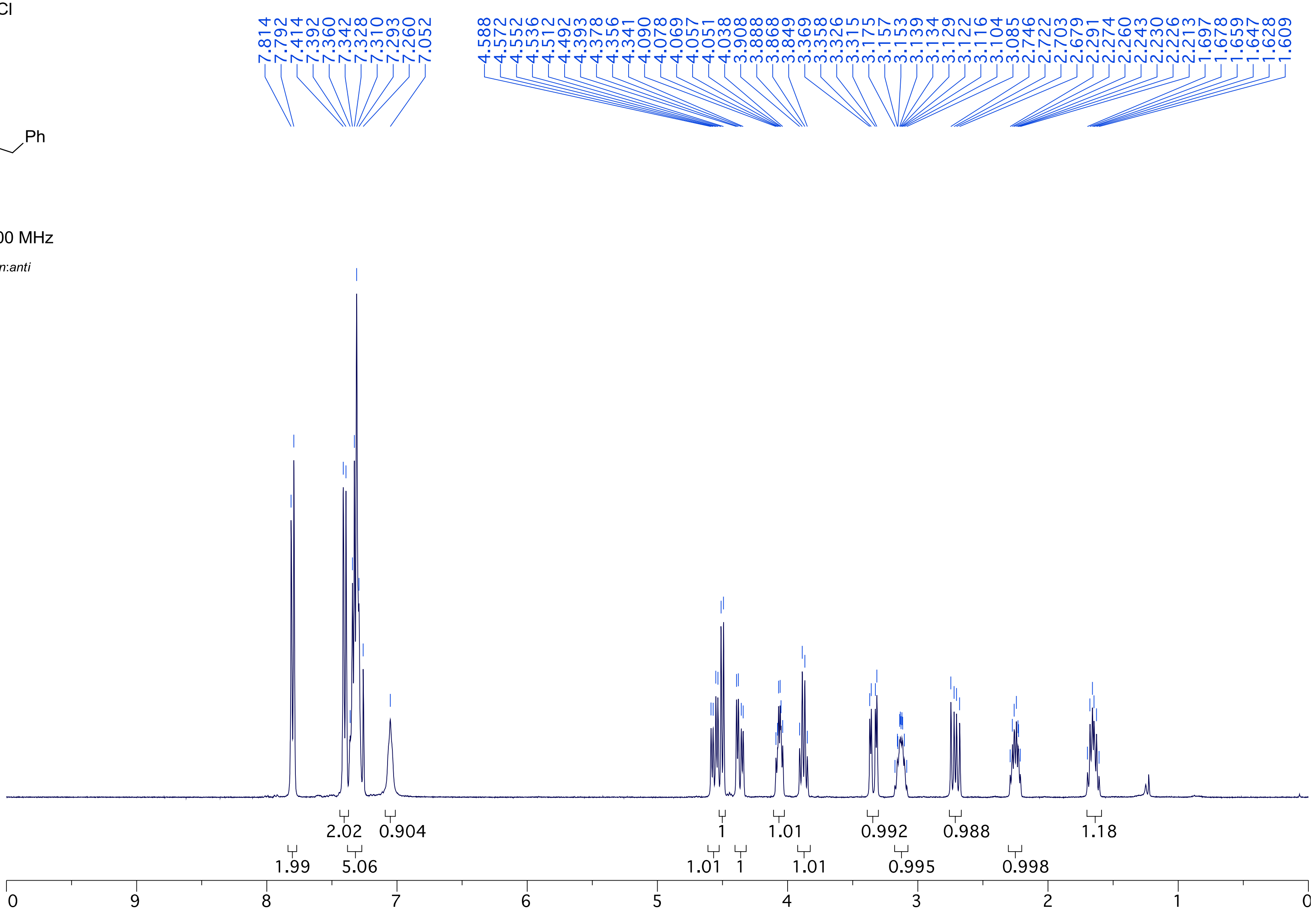

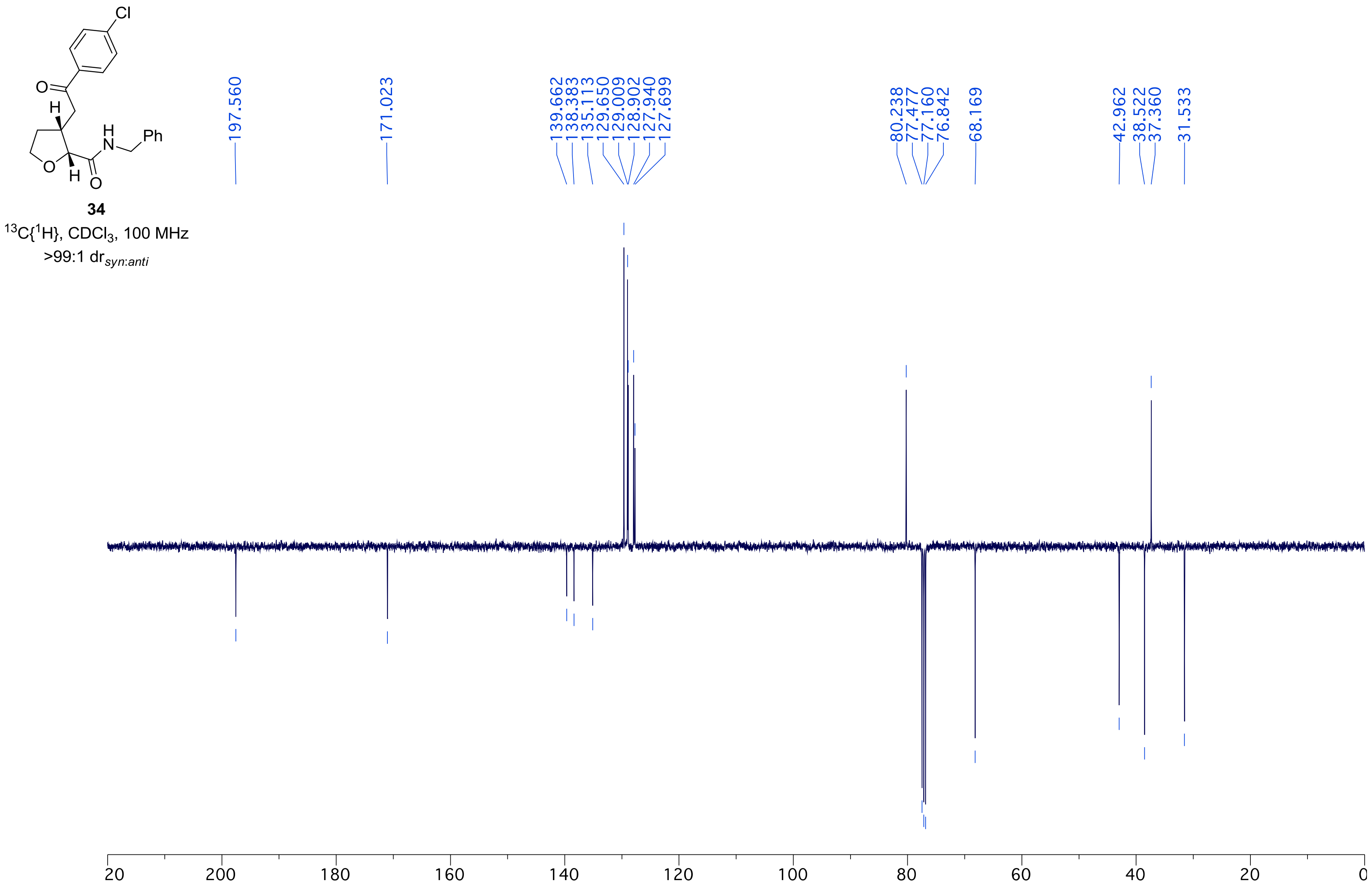

34

$^{13}C\{^{1}H\}$, $CDCl_3$, 100 MHz

>99:1 $dr_{syn:anti}$

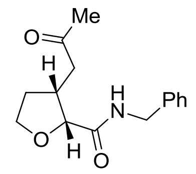

35

^1H, $CDCl_3$, 300 MHz

>99:1 $dr_{syn:anti}$

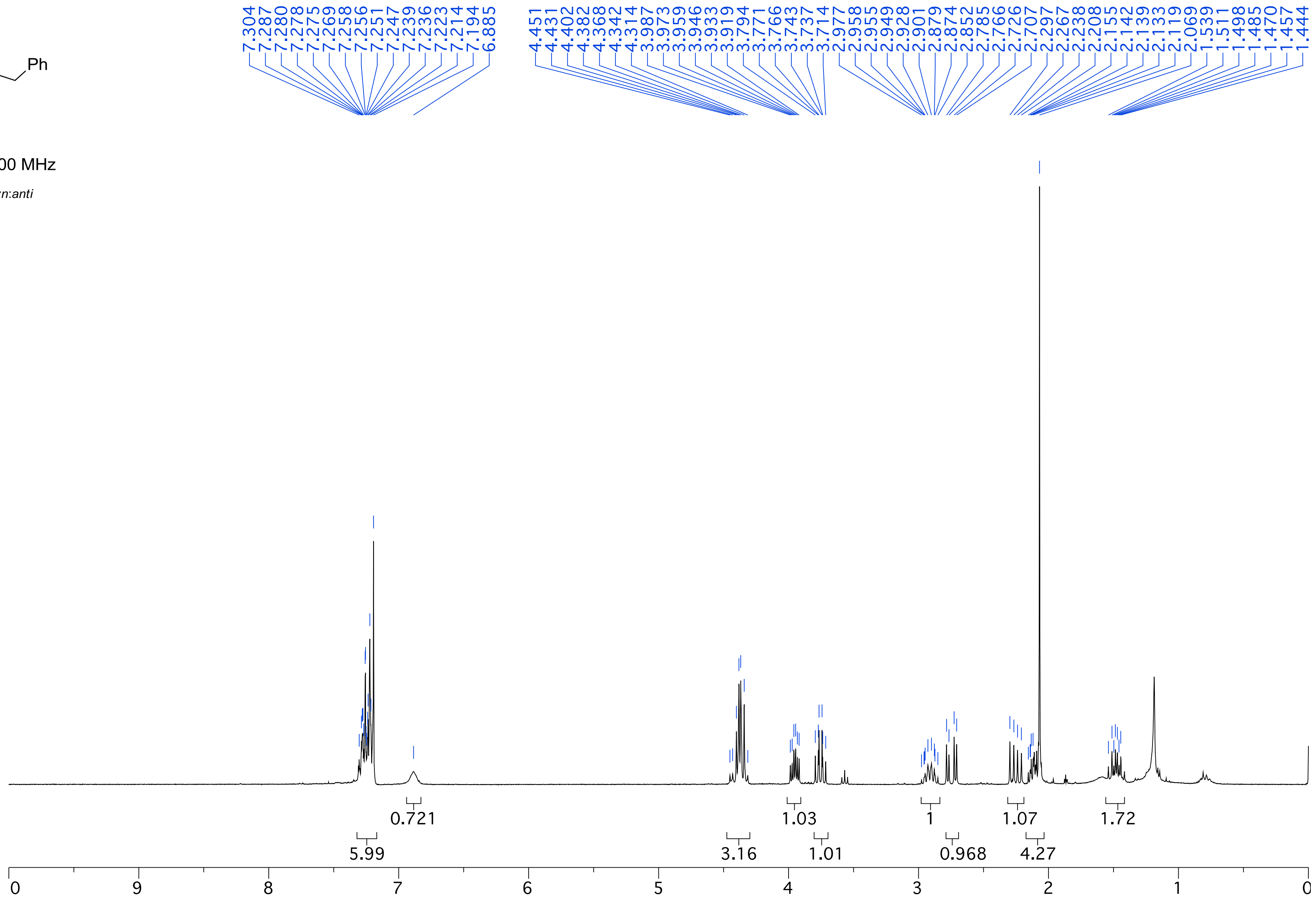

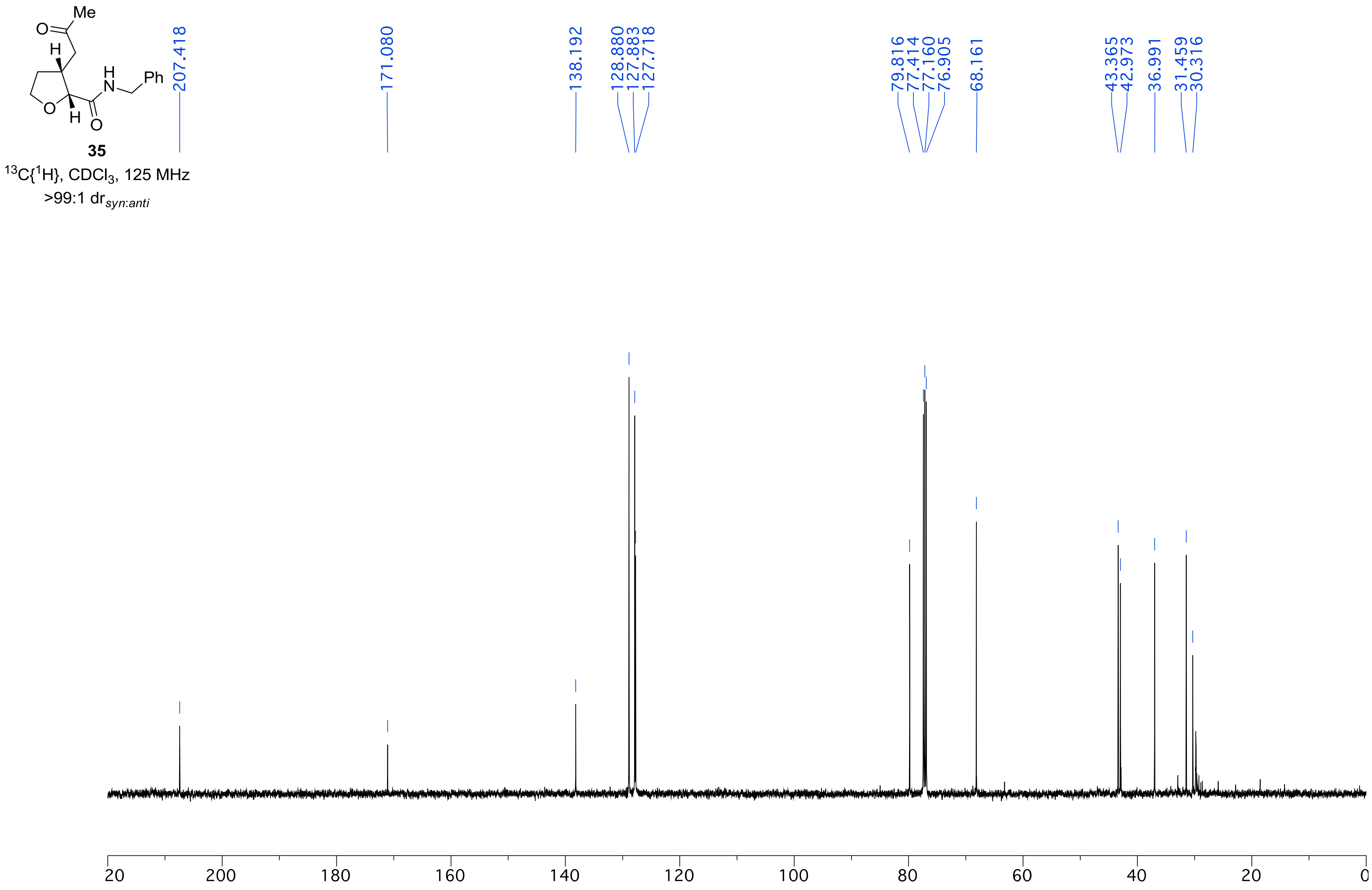
Me
O
H
H
N
Ph
O
H
O
35
$^{13}C\{^1H\}$, $CDCl_3$, 125 MHz
>99:1 $dr_{syn:anti}$
207.418
171.080
138.192
128.880
127.883
127.718
79.816
77.414
77.160
76.905
68.161
43.365
42.973
36.991
31.459
30.316
220
200
180
160
140
120
100
80
60
40
20
0

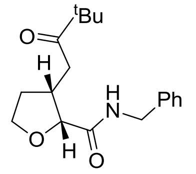

36

^{1}H, $CDCl_3$, 500 MHz

>99:1 $dr_{syn:anti}$

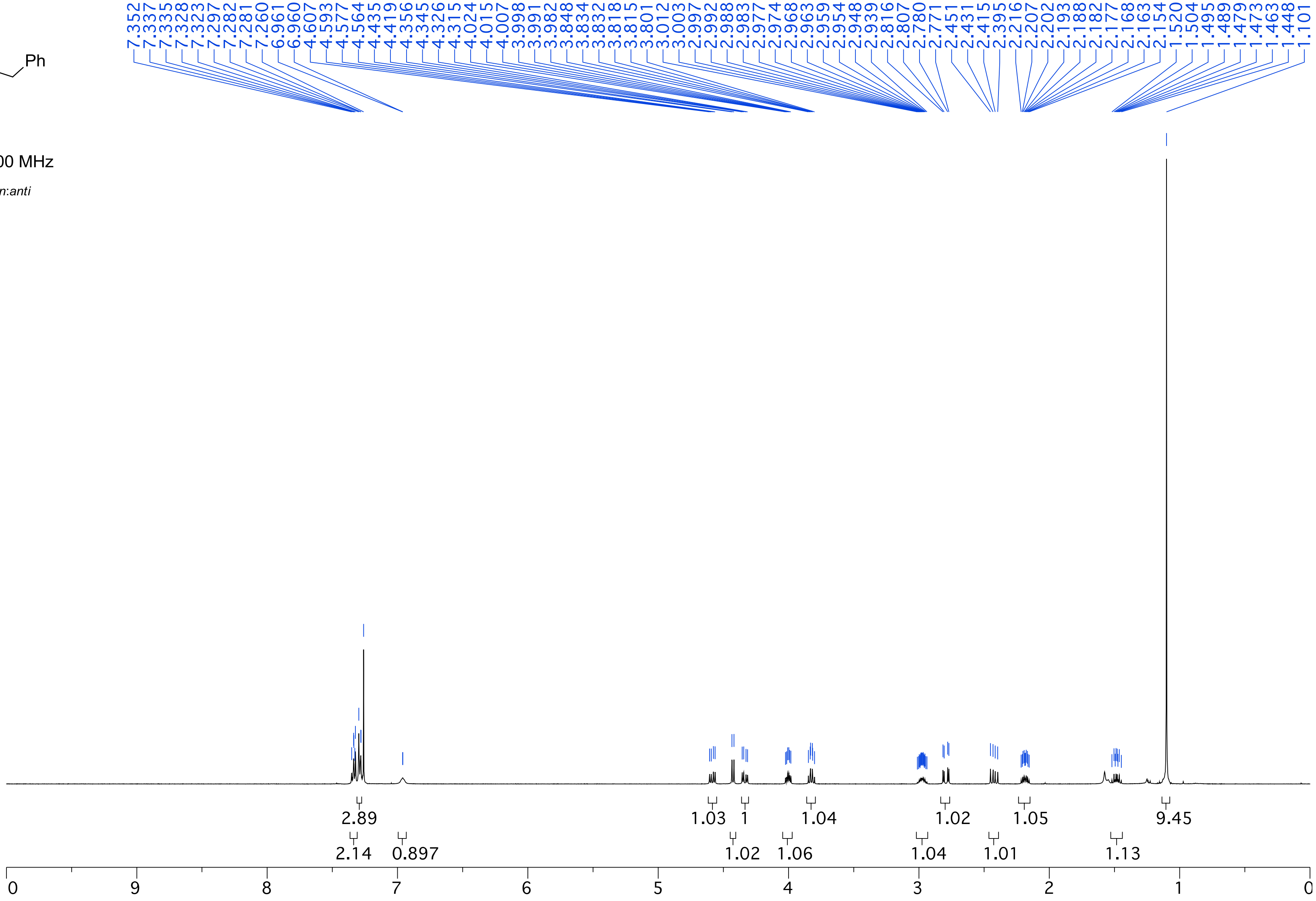

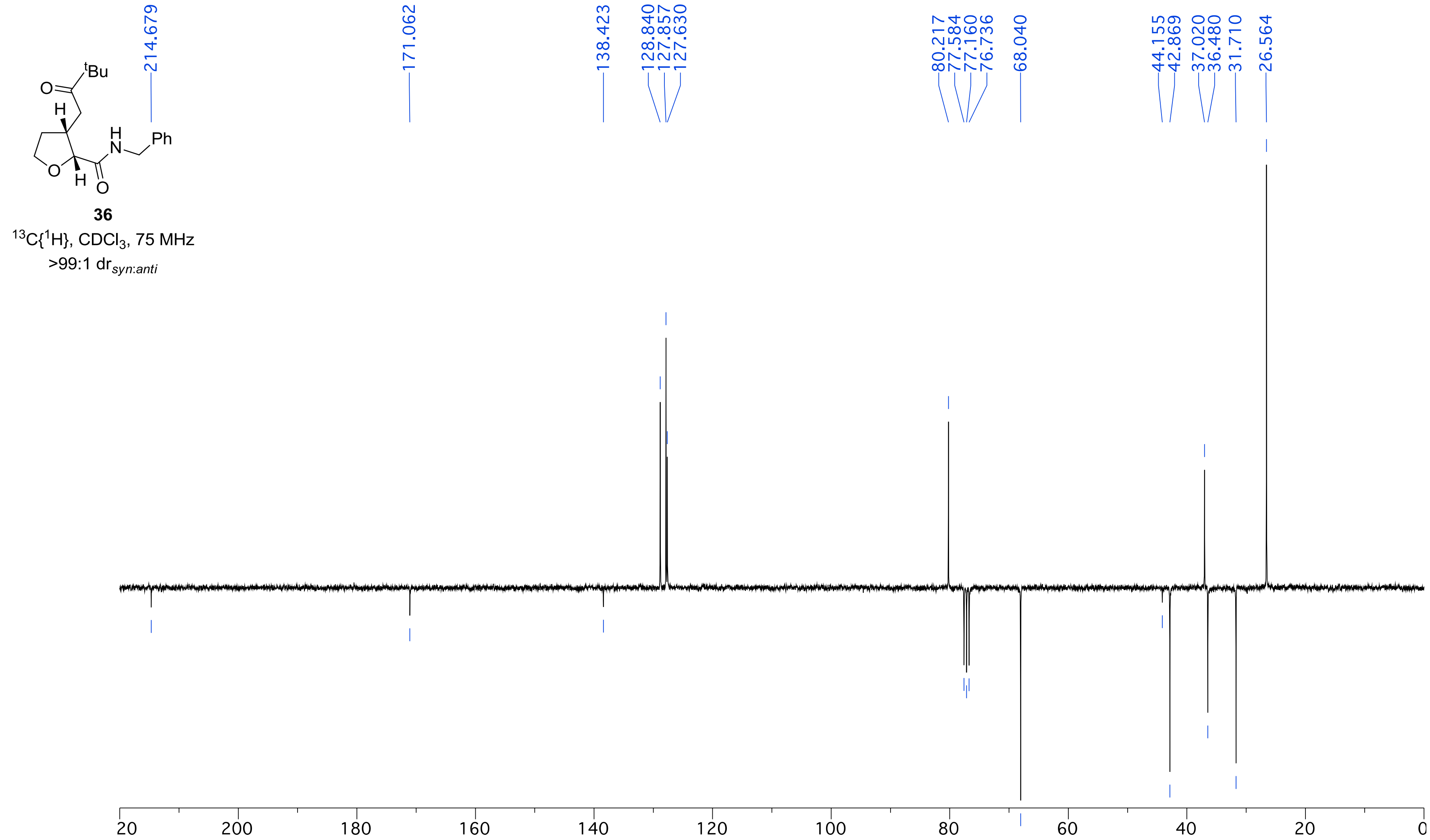

tBu
O
H
H
N
Ph
O
H
O
36
13C{1H}, CDCl3, 75 MHz
>99:1 dr syn:anti
214.679
171.062
138.423
128.840
127.857
127.630
80.217
77.584
77.160
76.736
68.040
44.155
42.869
37.020
36.480
31.710
26.564
20
200
180
160
140
120
100
80
60
40
20
0

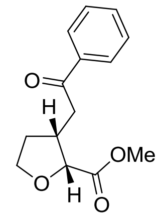

37

^{1}H, $CDCl_3$, 400 MHz

>99:1 $dr_{syn:anti}$

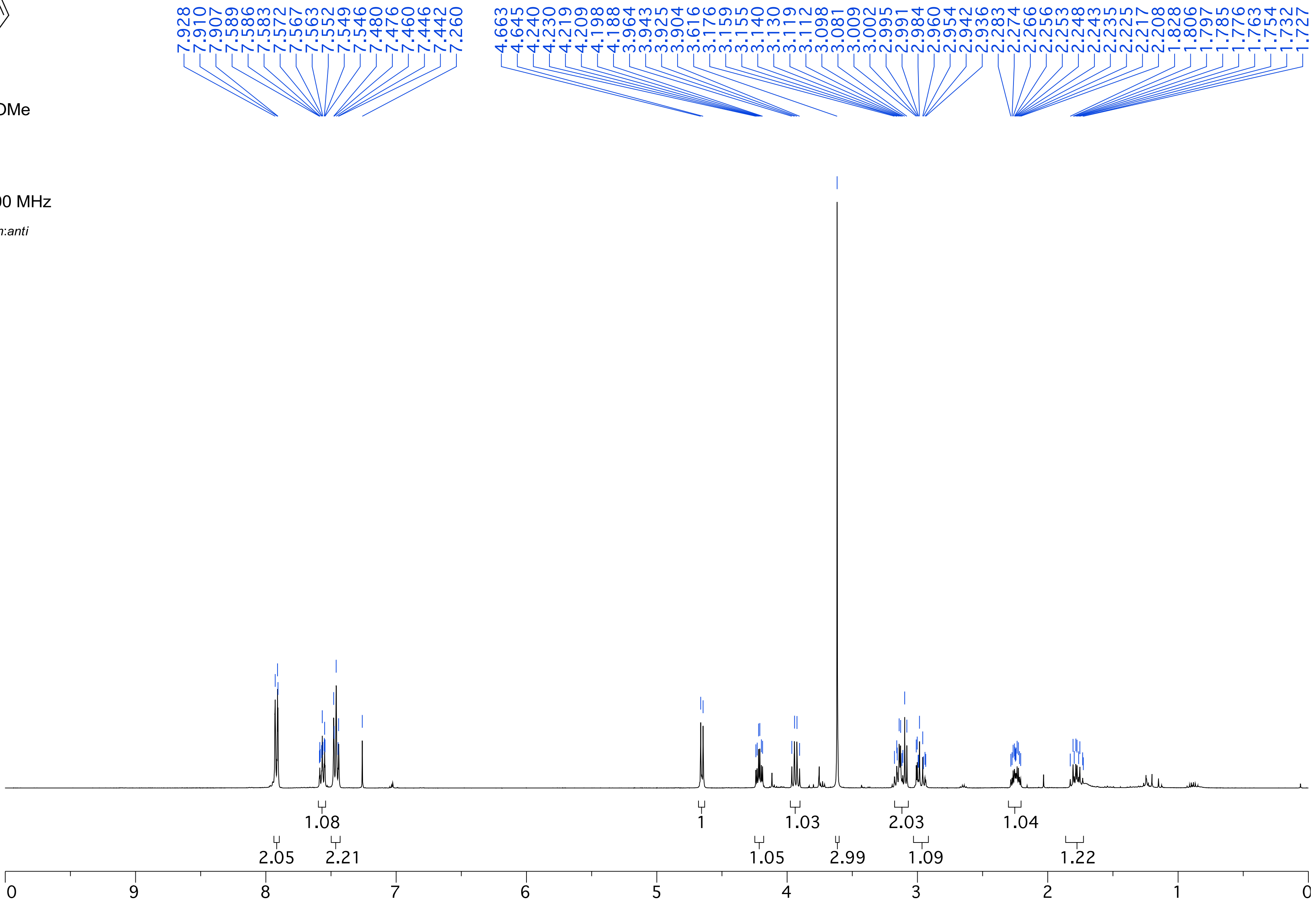

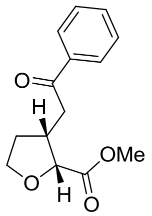

37

$^{13}C\{^1H\}$, $CDCl_3$, 100 MHz

>99:1 $dr_{syn:anti}$

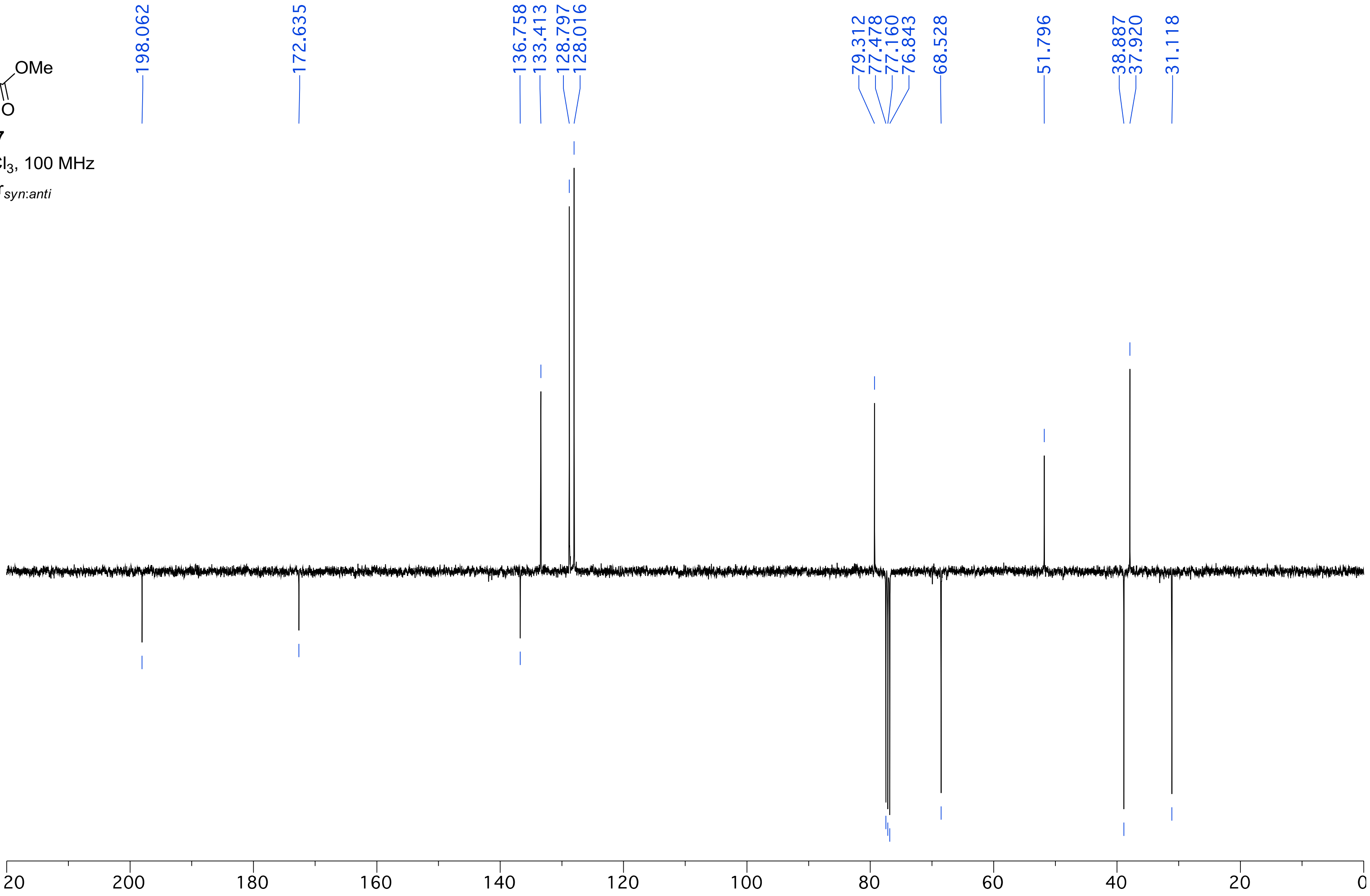

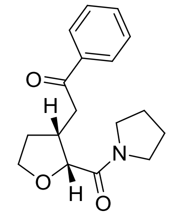

38

^{1}H, $CDCl_3$, 300 MHz

>99:1 $dr_{syn:anti}$

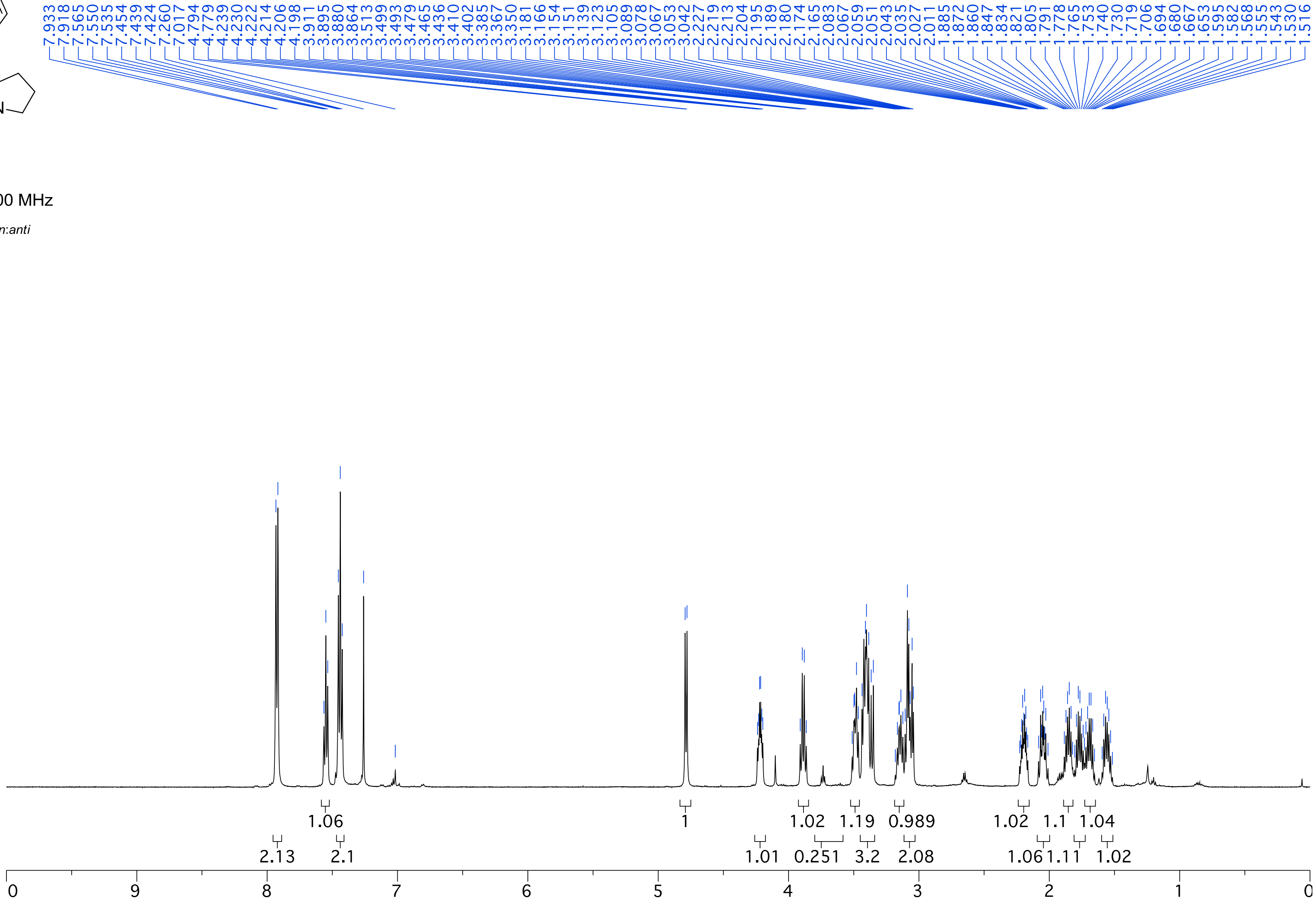

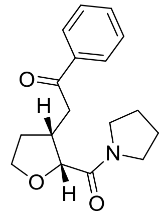

38

$^{13}C\{^{1}H\}$, $CDCl_3$, 75 MHz

>99:1 $dr_{syn:anti}$

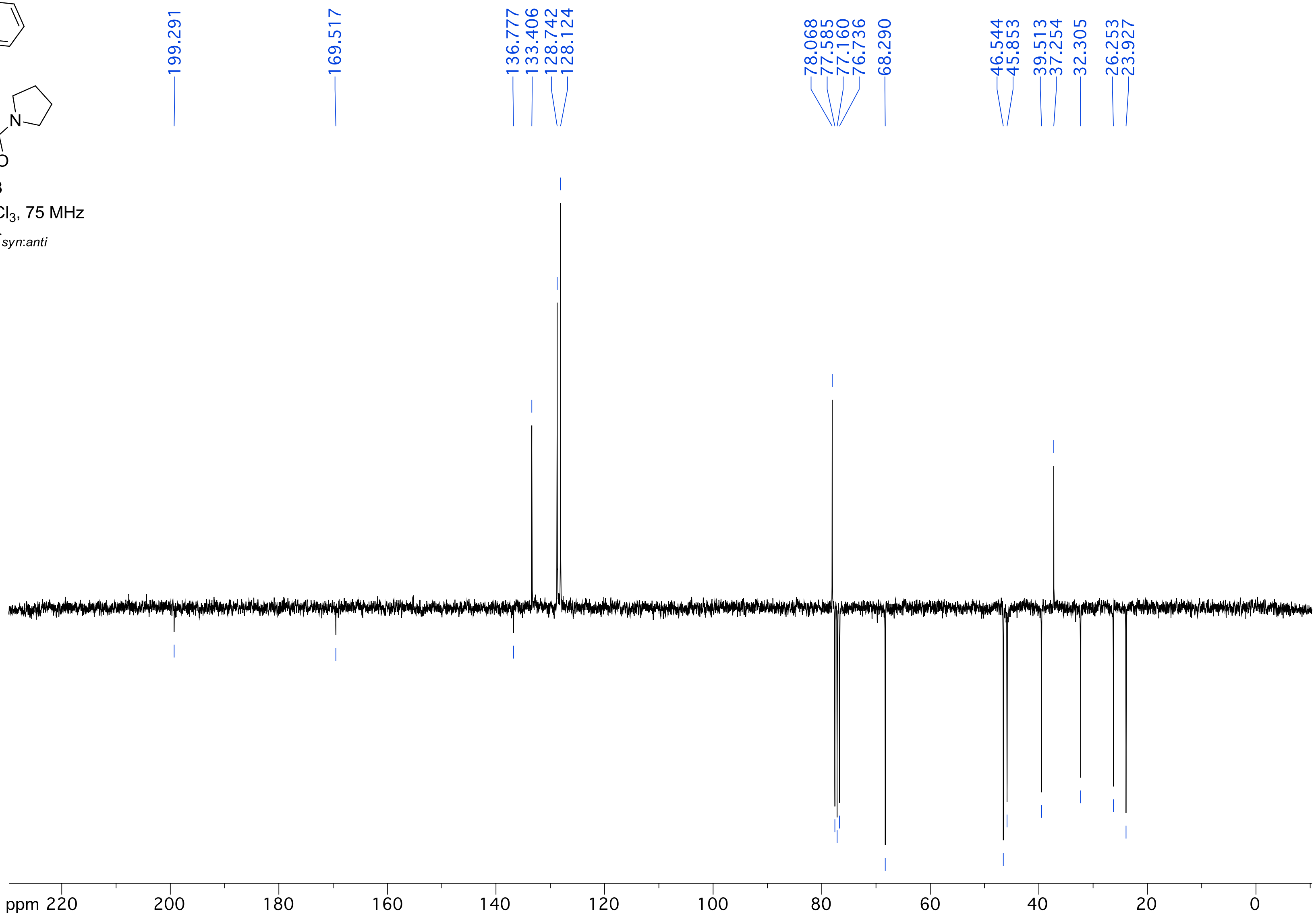

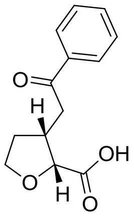

39

^{1}H, $CDCl_3$, 500 MHz

>99:1 $dr_{syn:anti}$

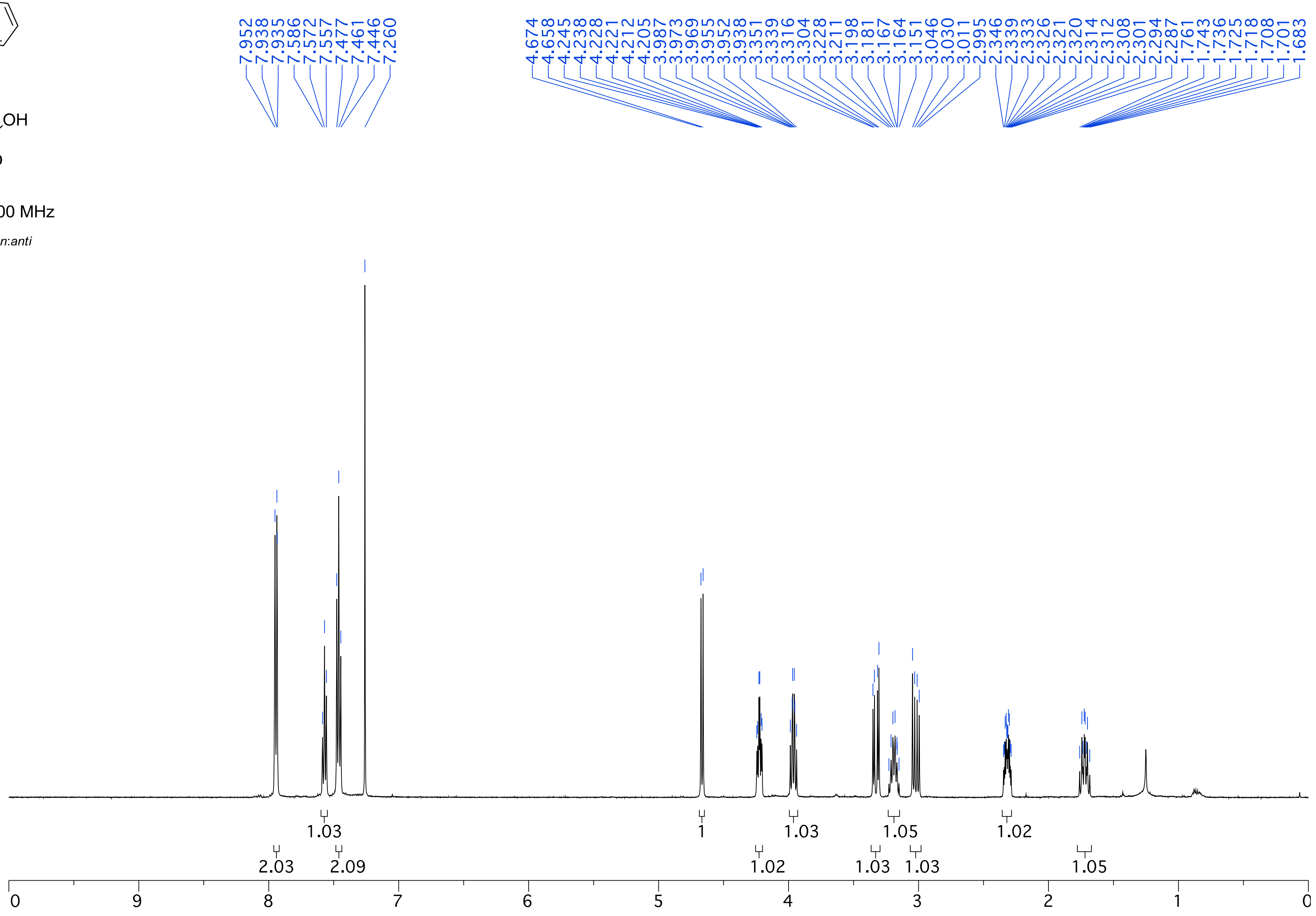

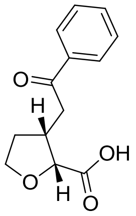

39

$^{13}C\{^{1}H\}$, $CDCl_3$, 75 MHz

>99:1 $dr_{syn:anti}$

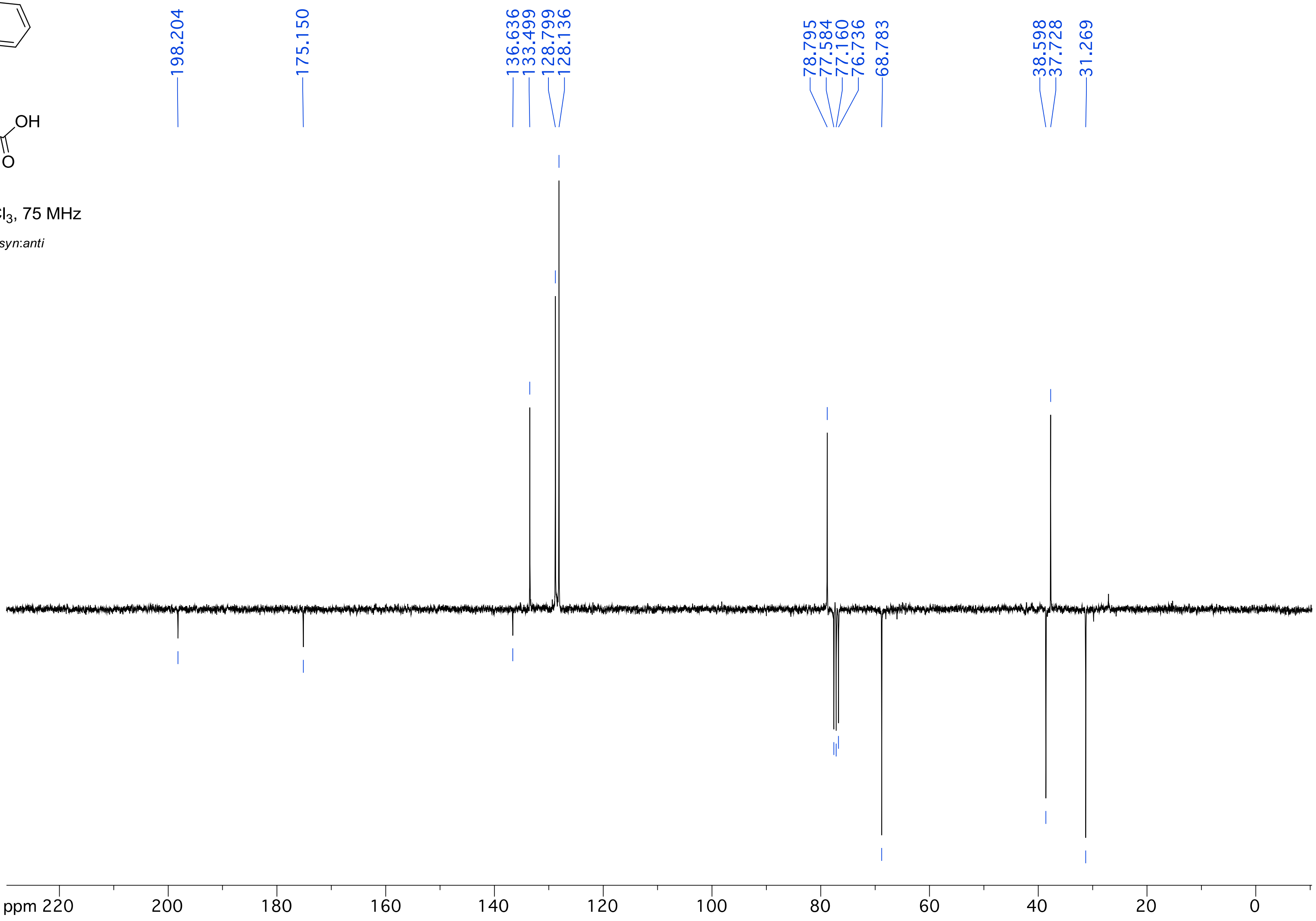

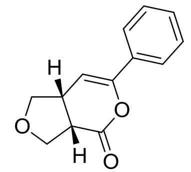

43

^{1}H, $CDCl_3$, 300 MHz

>99:1 $dr_{syn:anti}$

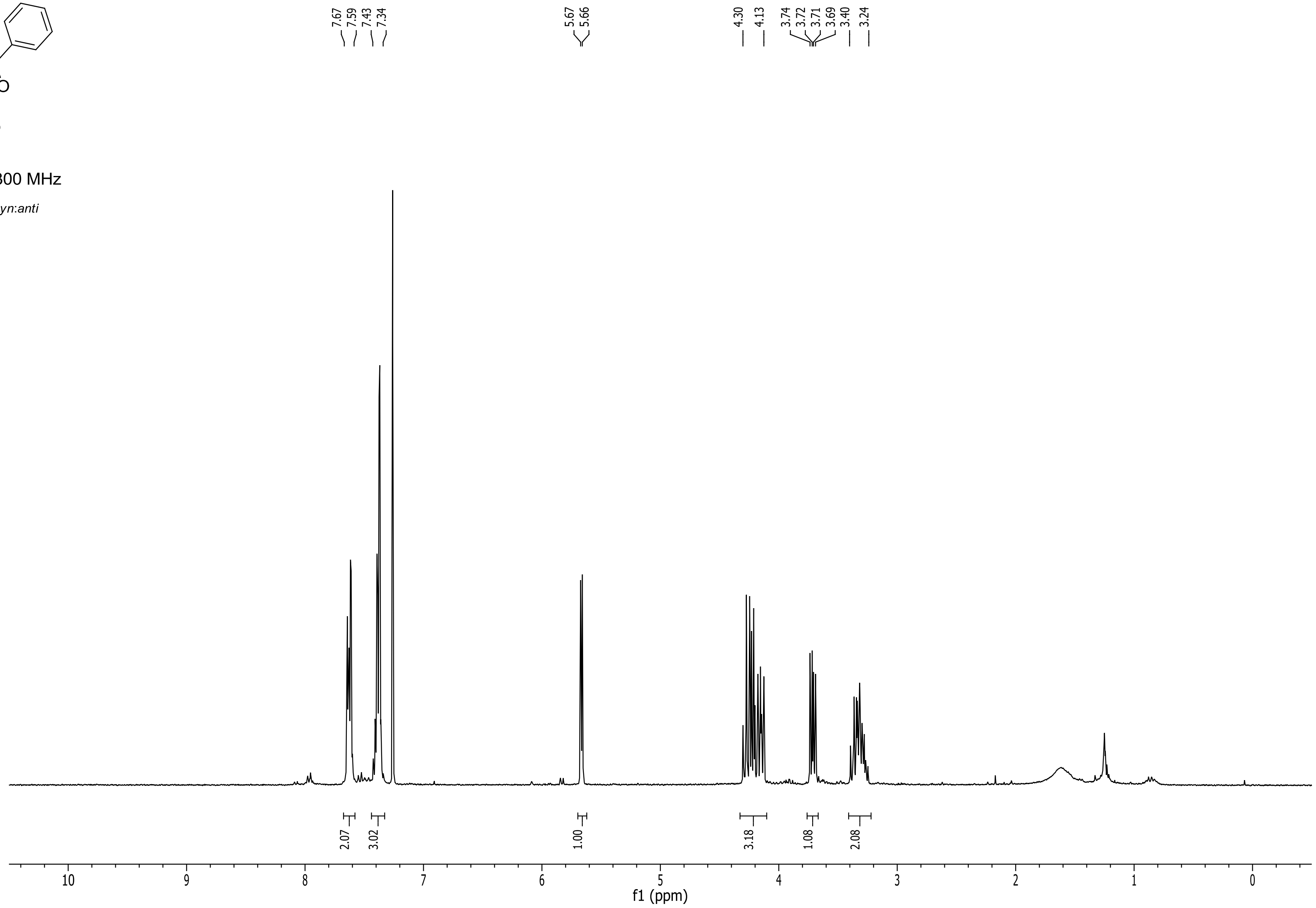

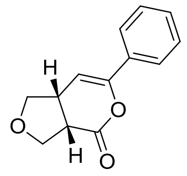

43

$^{13}C\{^{1}H\}$, $CDCl_3$, 75 MHz

>99:1 dr$_{syn:anti}$

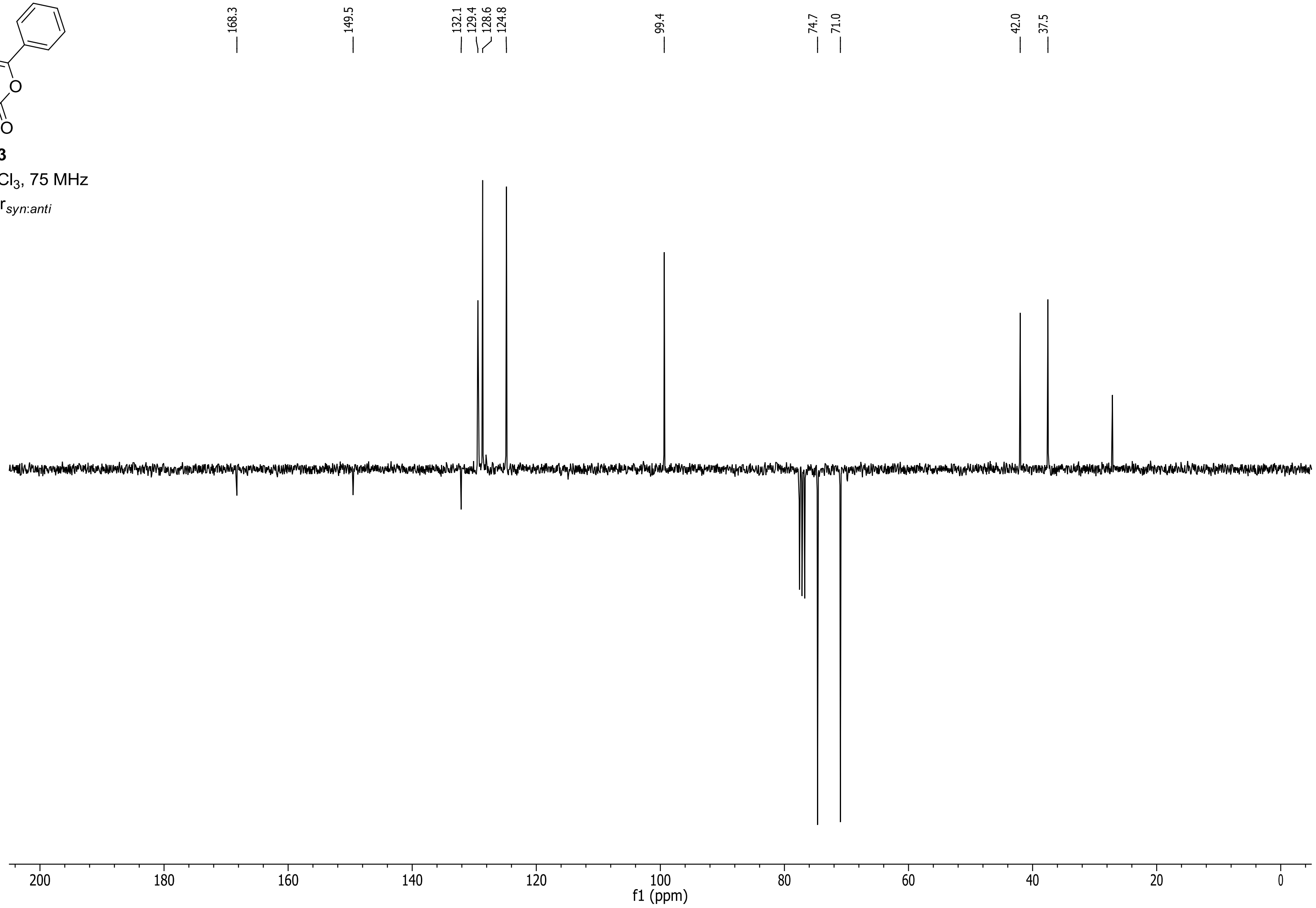

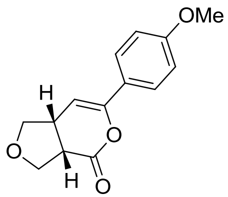

44

^{1}H, $CDCl_3$, 300 MHz

>99:1 dr$_{syn:anti}$

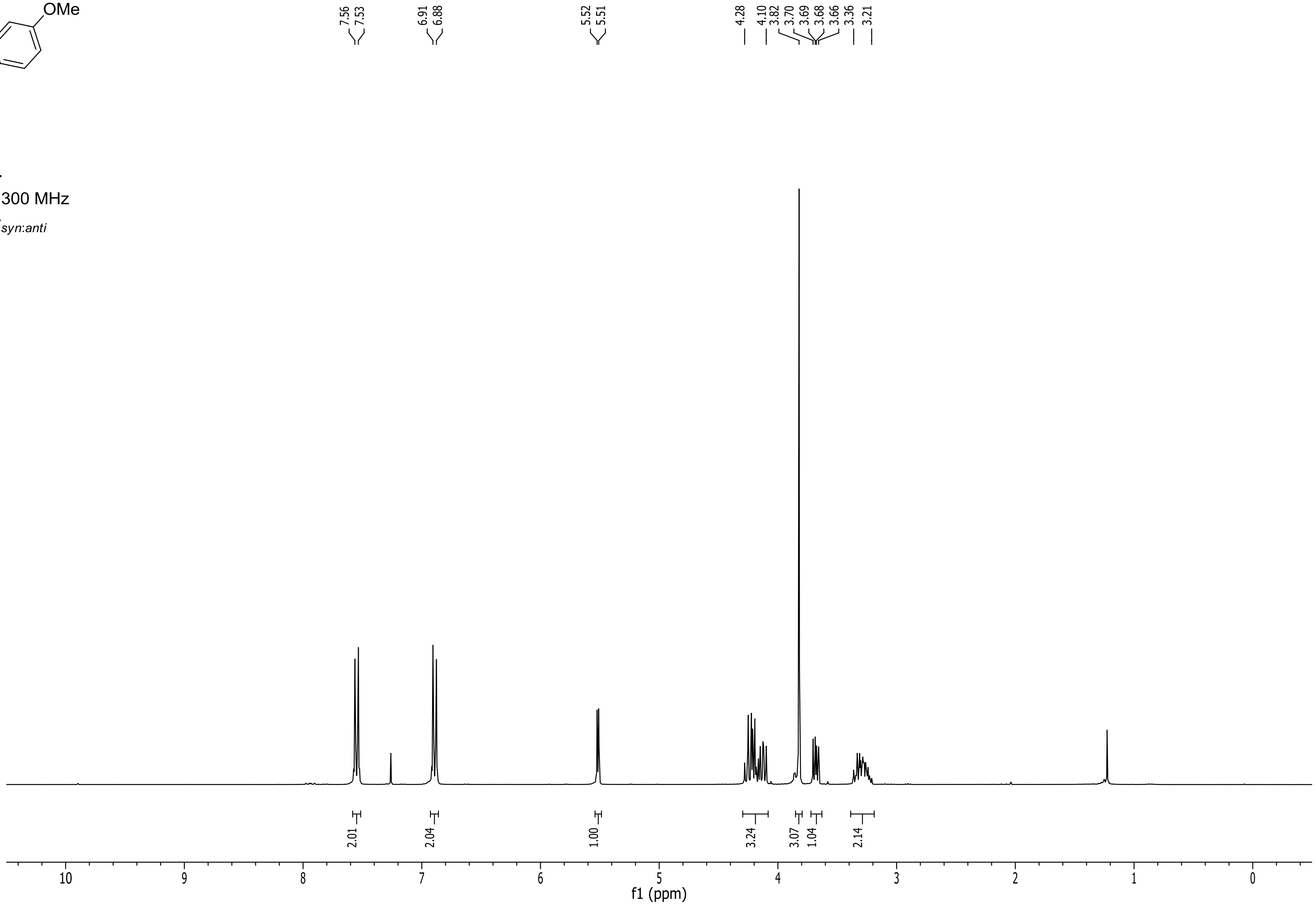

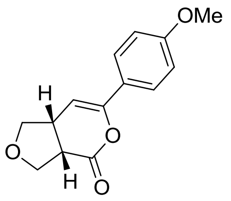

44

$^{13}C\{^{1}H\}$, $CDCl_3$, 75 MHz

>99:1 dr$_{syn:anti}$

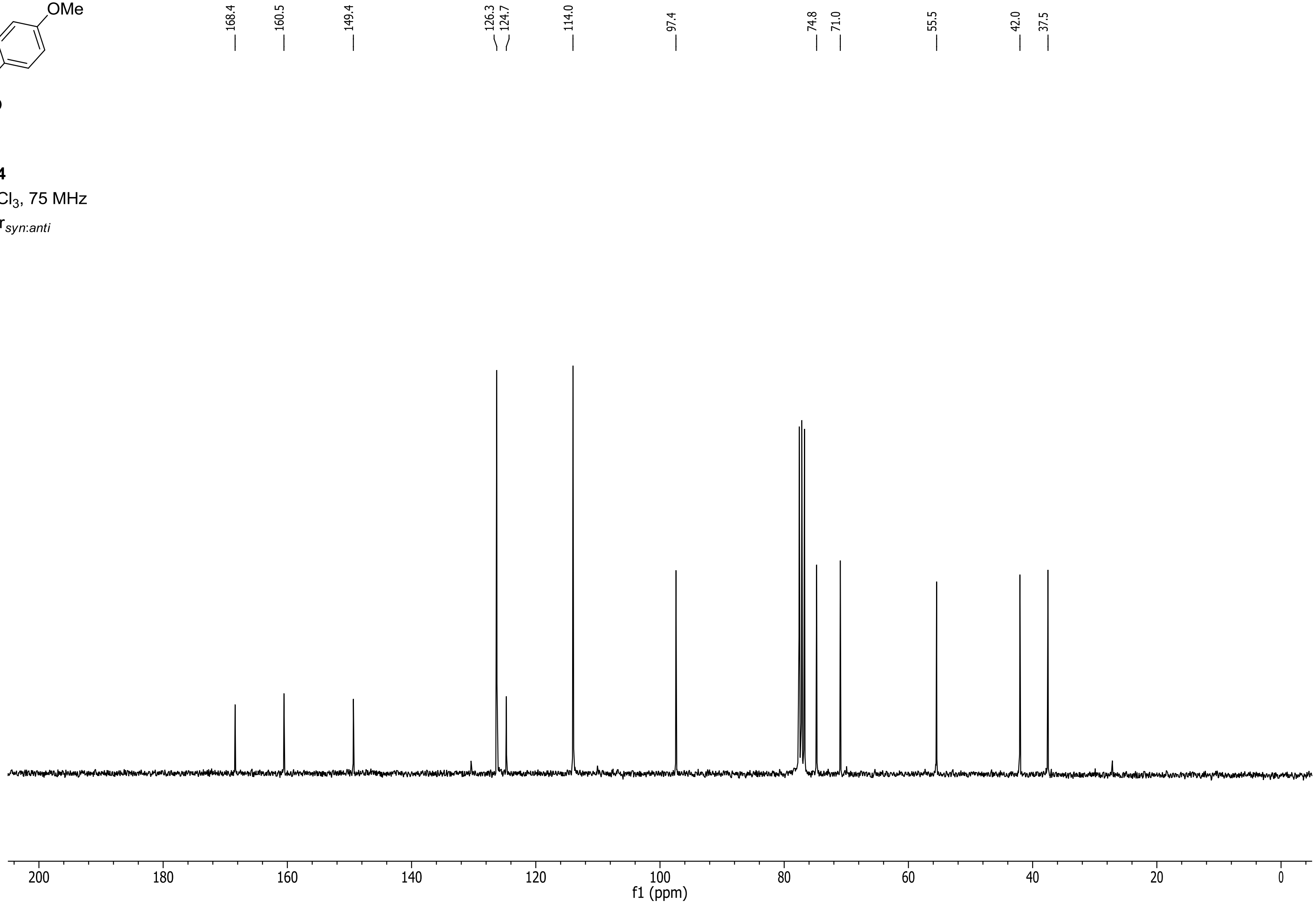

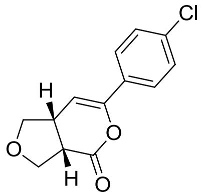

45

^{1}H, $CDCl_3$, 400 MHz

>99:1 $dr_{syn:anti}$

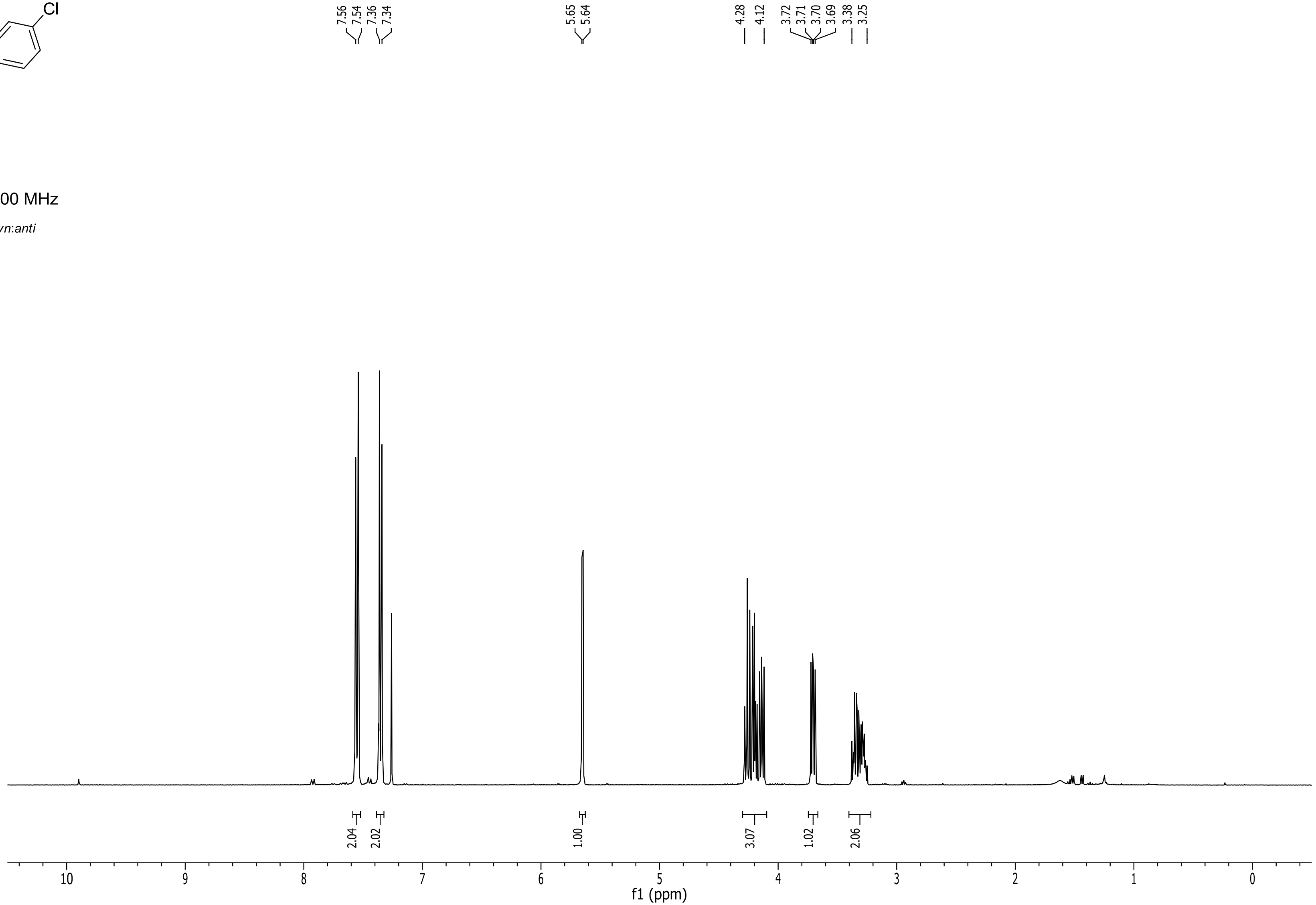

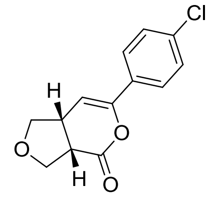

45

$^{13}C\{^{1}H\}$, $CDCl_3$, 100 MHz

>99:1 $dr_{syn:anti}$

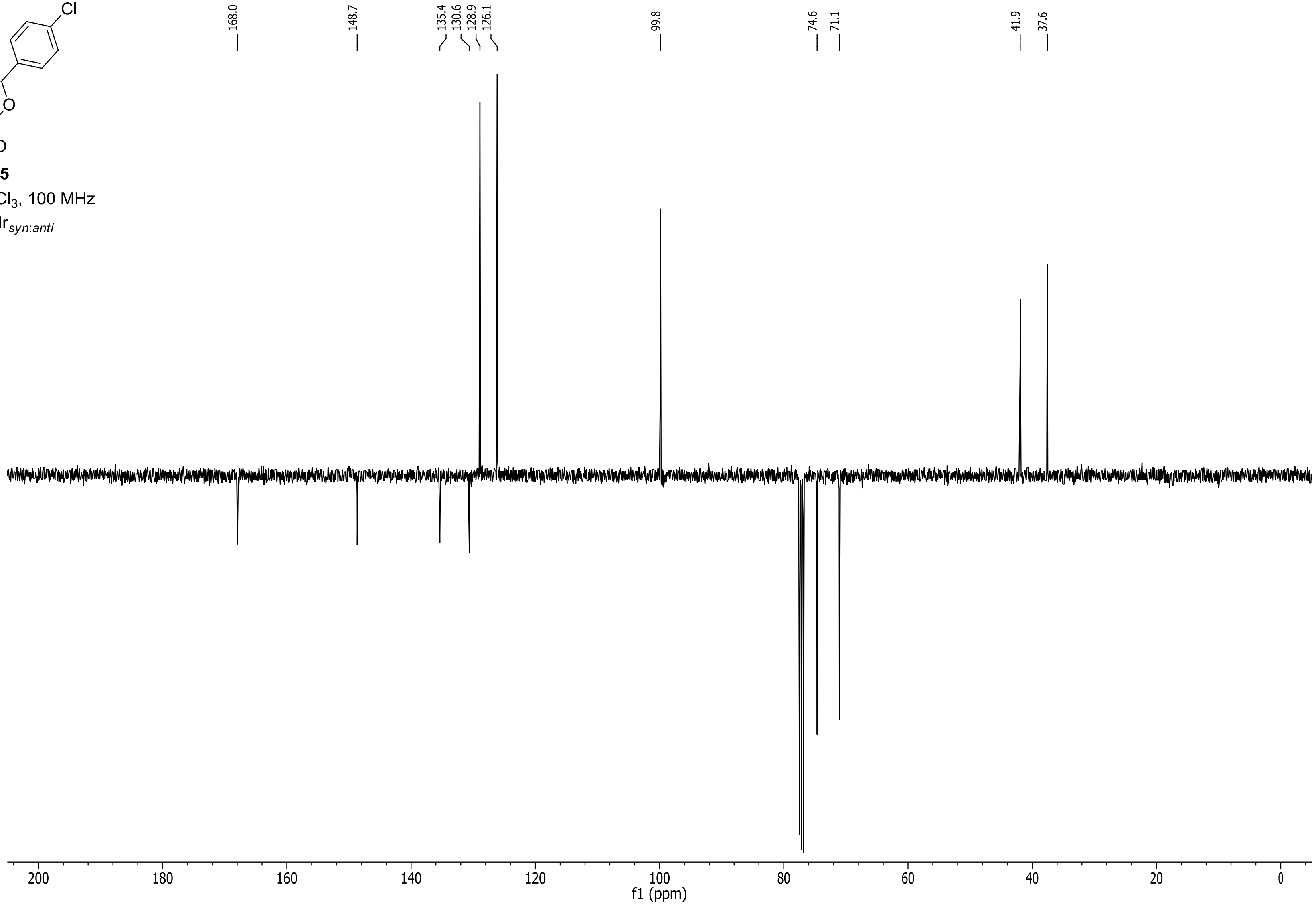

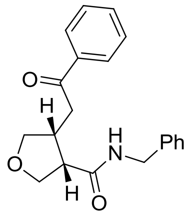

46

^{1}H, $CDCl_3$, 400 MHz

>99:1 $dr_{syn:anti}$

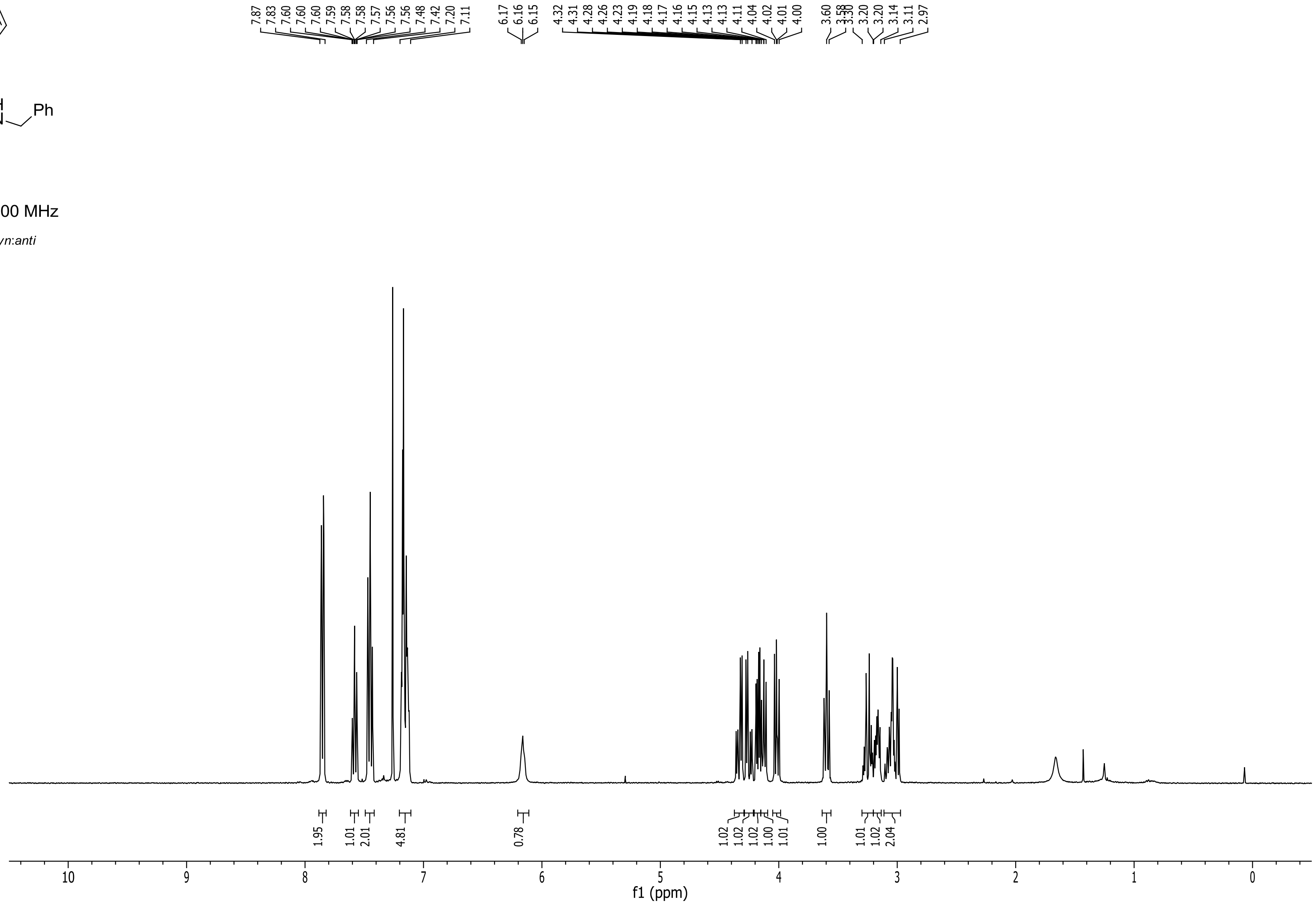

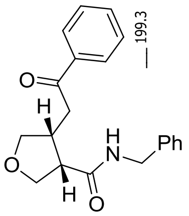

46

$^{13}C\{^{1}H\}$, $CDCl_3$, 100 MHz

>99:1 $dr_{syn:anti}$

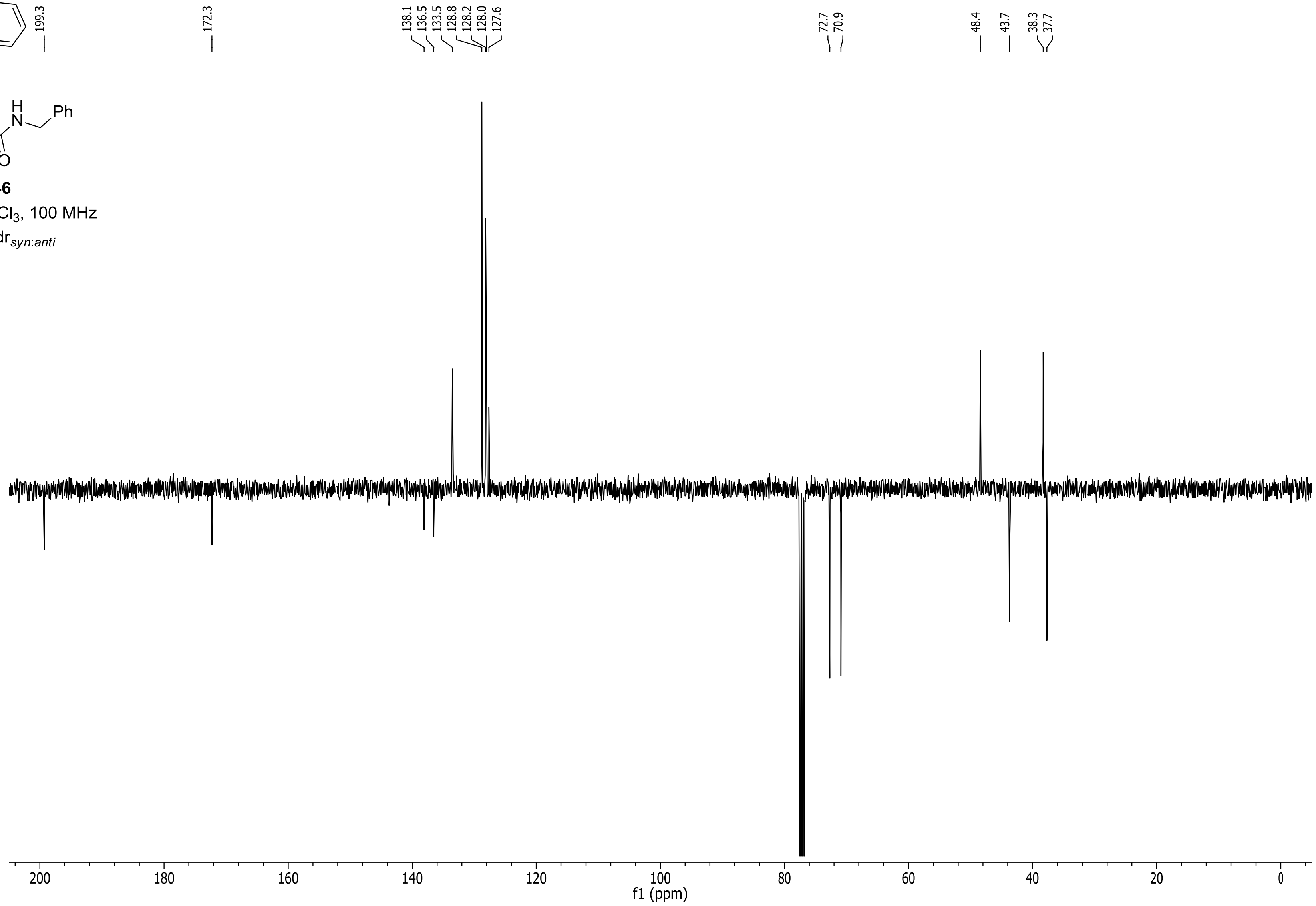

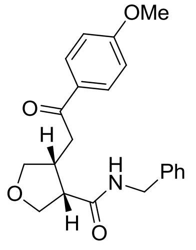

47

^1H, $CDCl_3$, 300 MHz

>99:1 $dr_{syn:anti}$

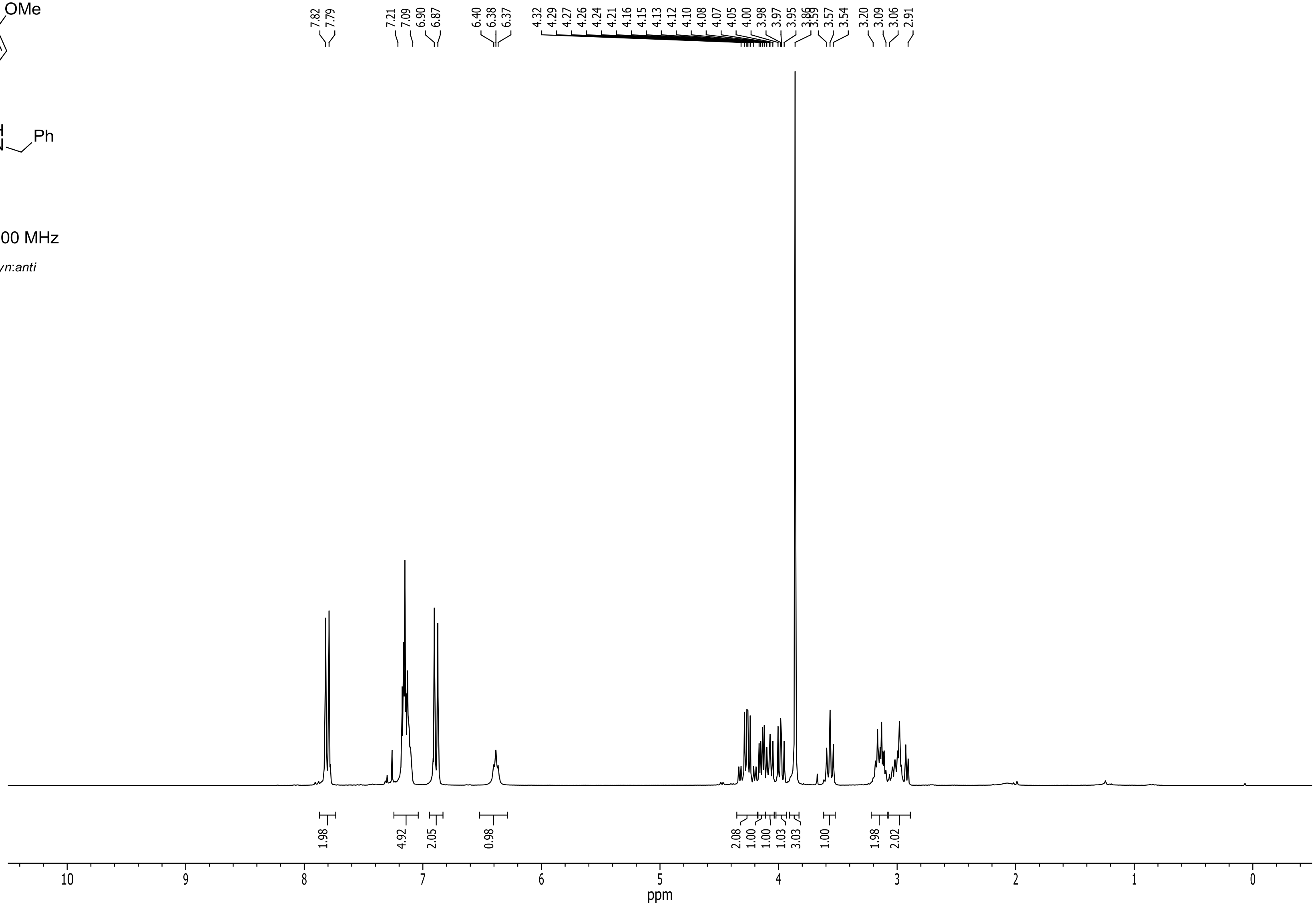

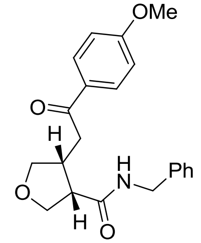

47

$^{13}C\{^{1}H\}$, $CDCl_3$, 75 MHz

>99:1 $dr_{syn:anti}$

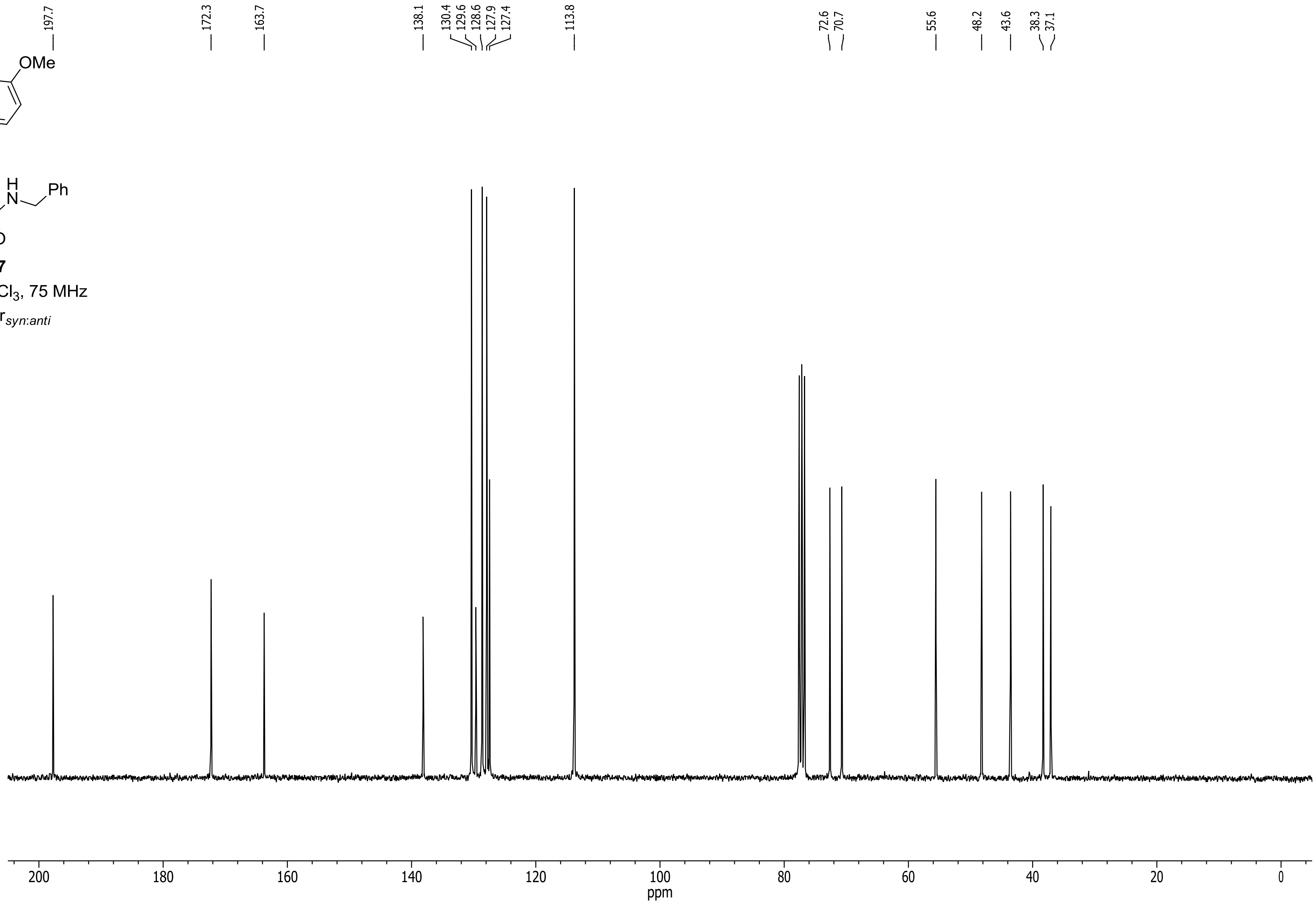

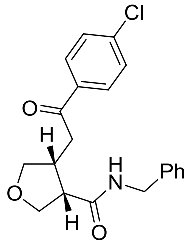

48

^{1}H, $CDCl_3$, 500 MHz

>99:1 $dr_{syn:anti}$

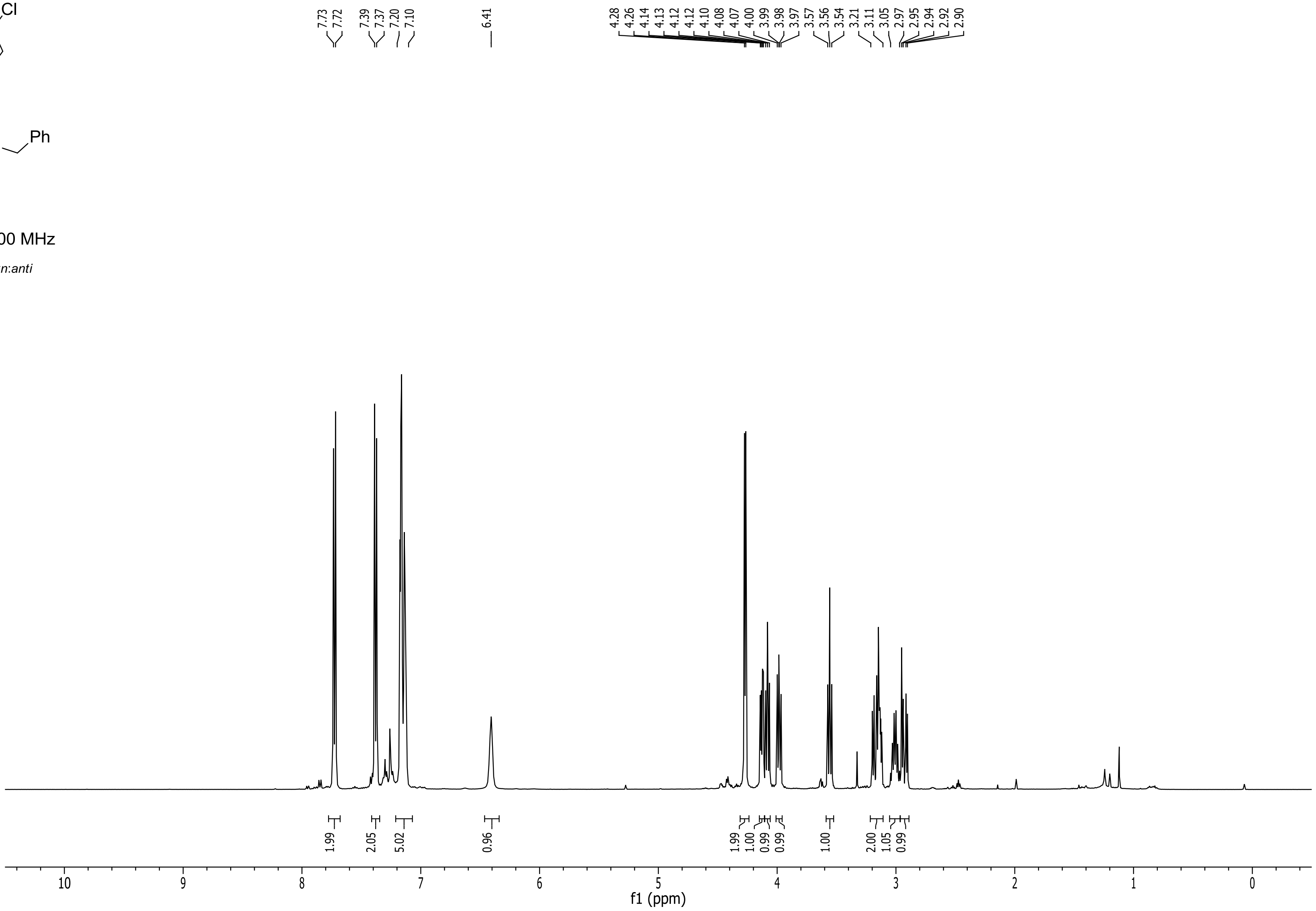

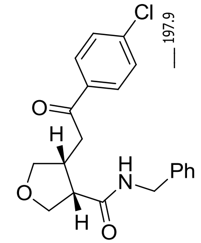

48

$^{13}C\{^{1}H\}$, $CDCl_3$, 125 MHz

>99:1 $dr_{syn:anti}$

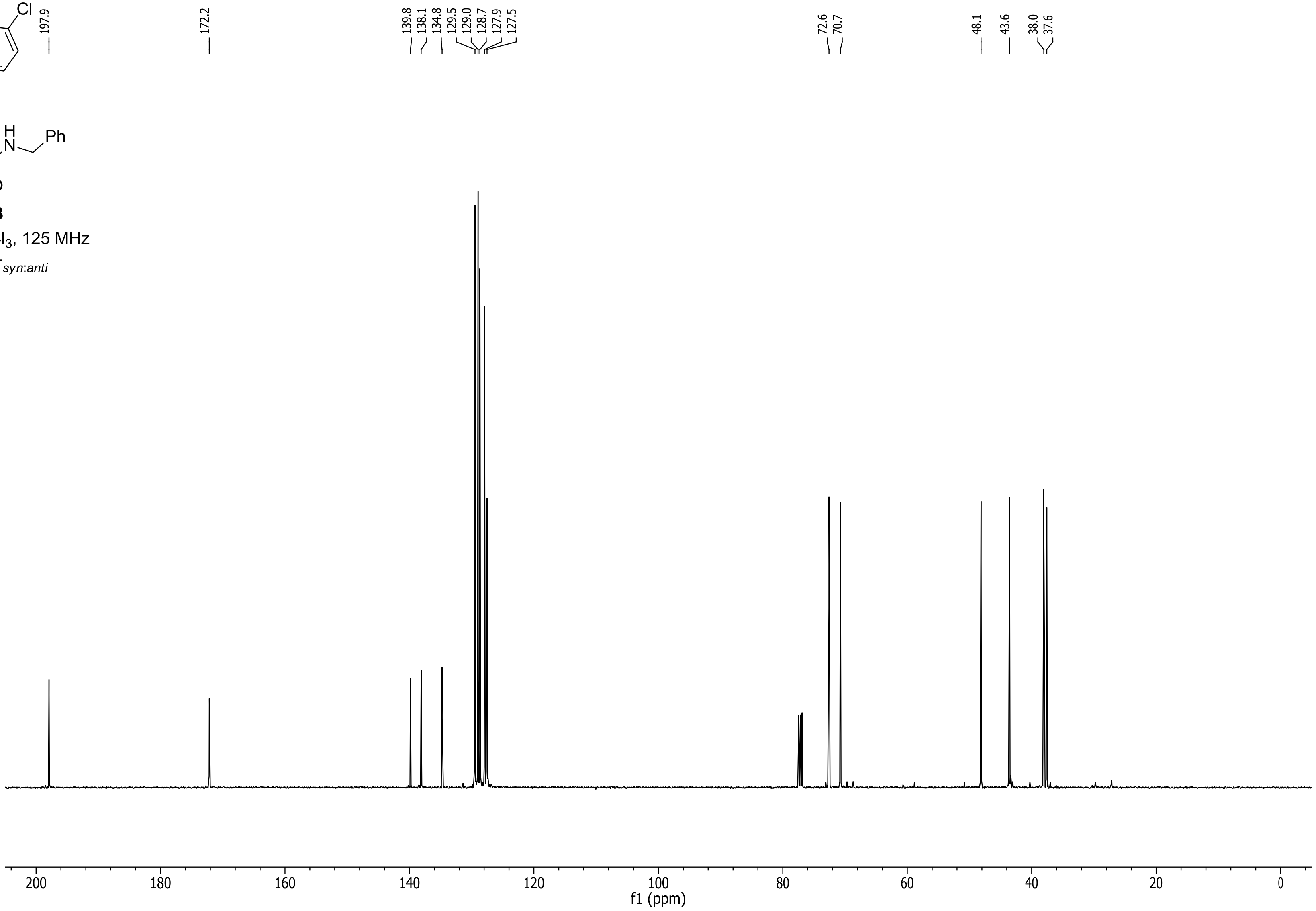

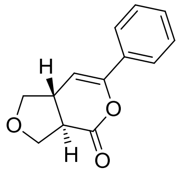

49

^{1}H, $CDCl_3$, 500 MHz

>1:99 $dr_{syn:anti}$

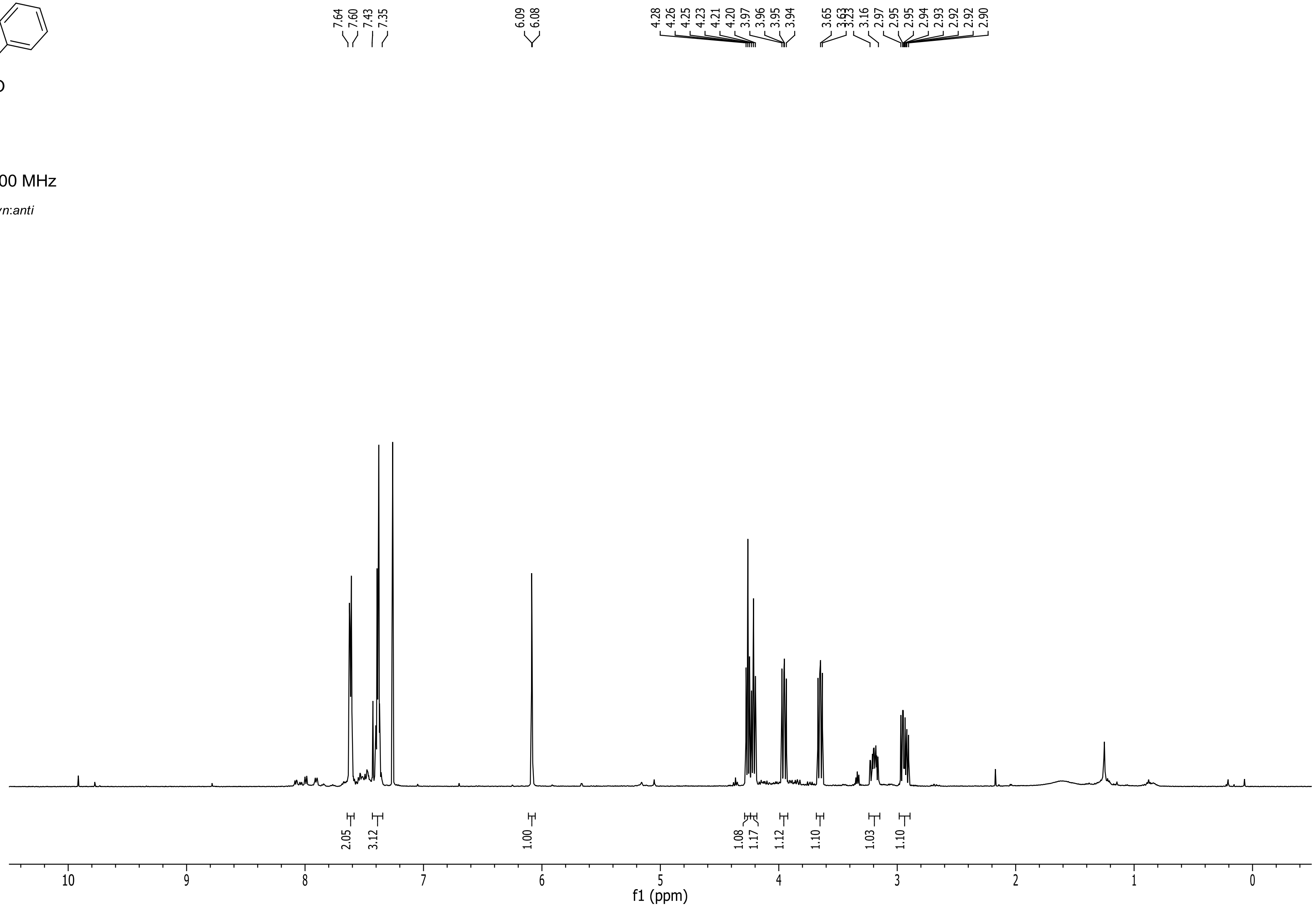

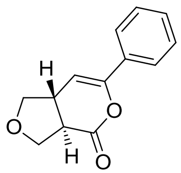

49

$^{13}C\{^{1}H\}$, $CDCl_3$, 125 MHz

>1:99 $dr_{syn:anti}$

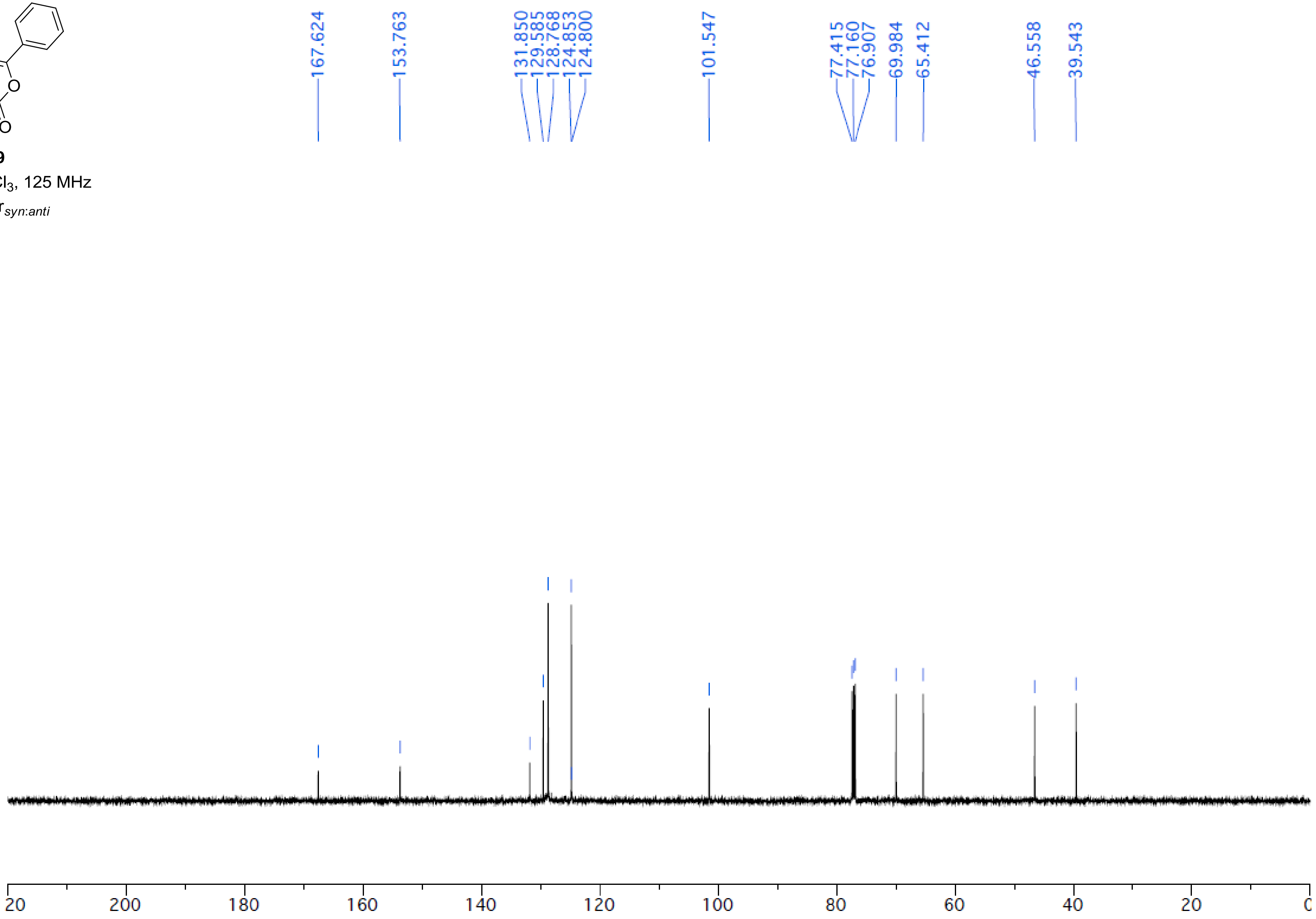

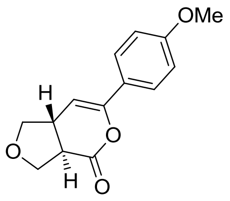

50

^{1}H, $CDCl_3$, 300 MHz

15:85 $dr_{syn:anti}$

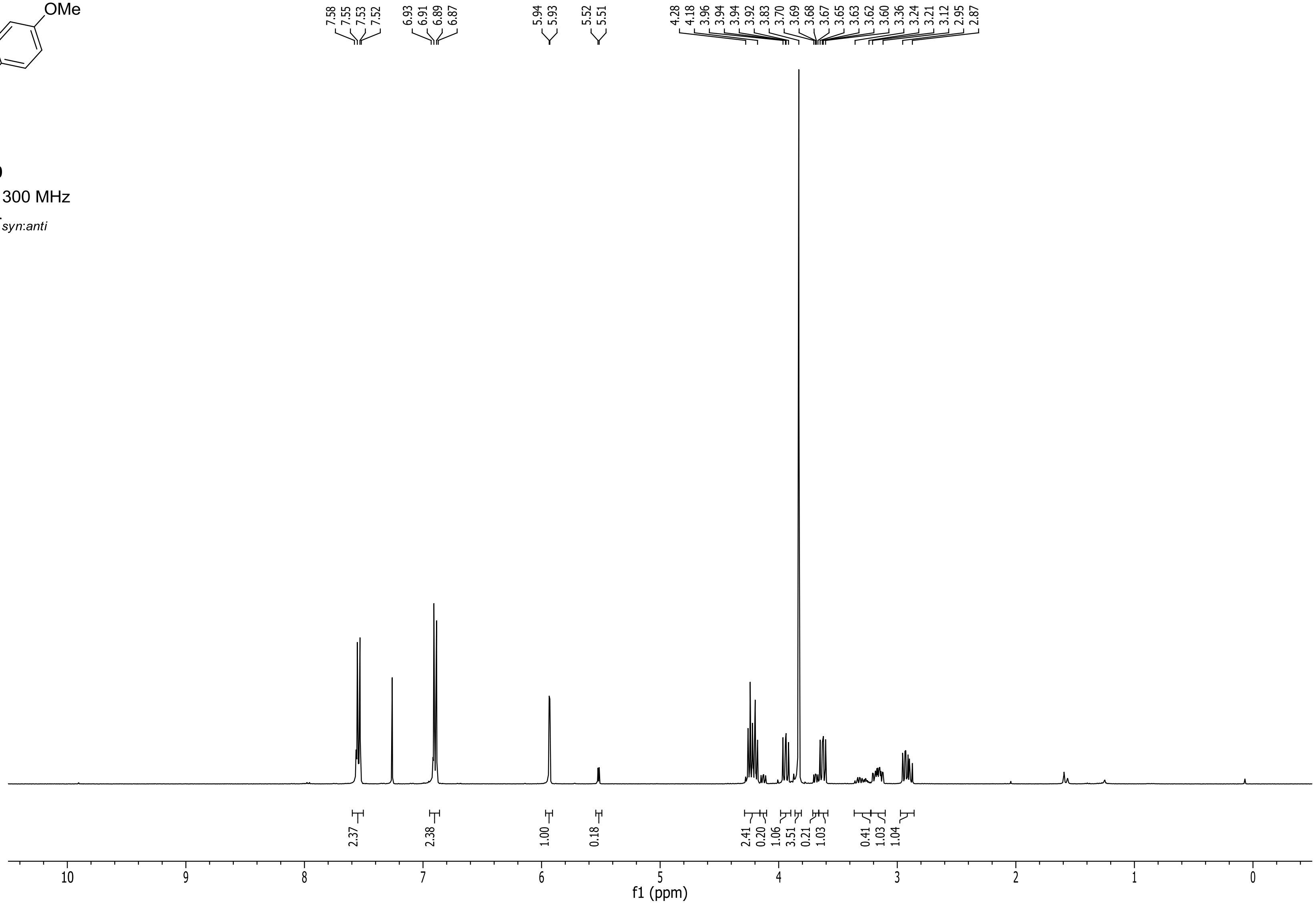

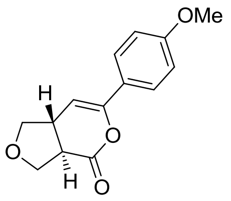

50

$^{13}C\{^{1}H\}$, $CDCl_3$, 75 MHz

15:85 $dr_{syn:anti}$

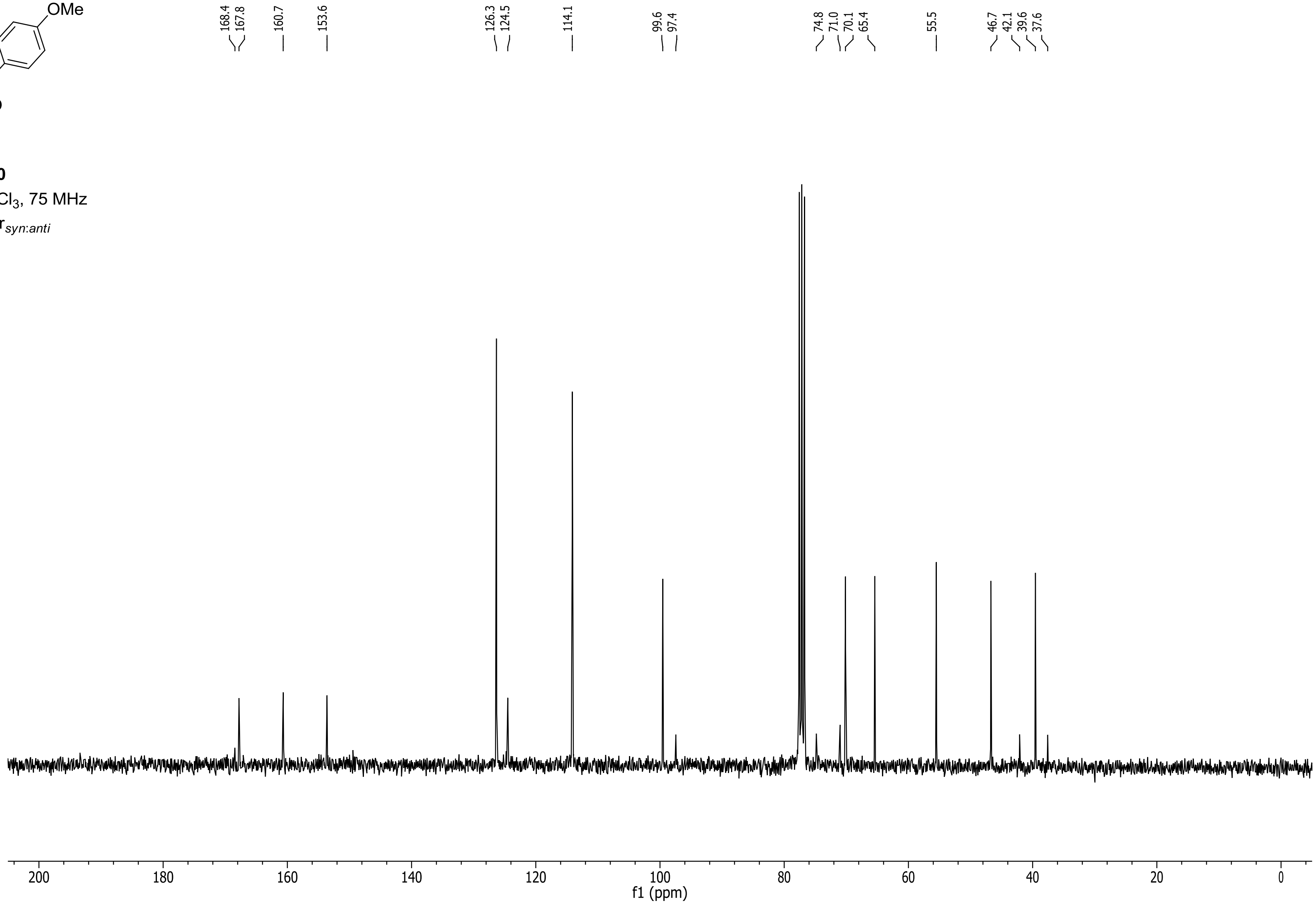

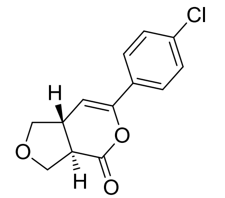

51

^{1}H, $CDCl_3$, 300 MHz

18:82 $dr_{syn:anti}$

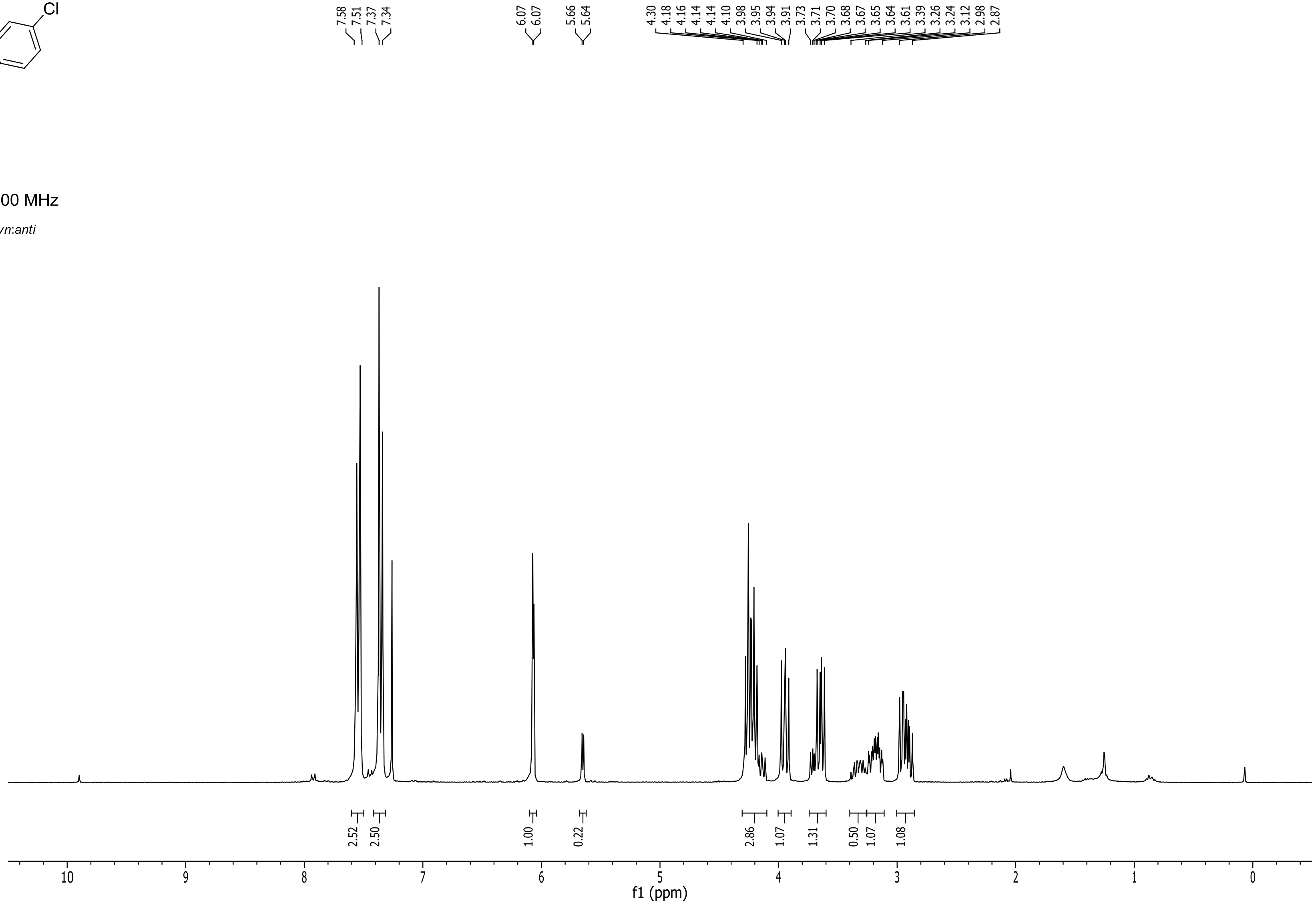

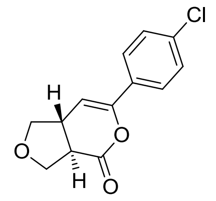

51

$^{13}C\{^{1}H\}$, $CDCl_3$, 125 MHz

18:82 $dr_{syn:anti}$

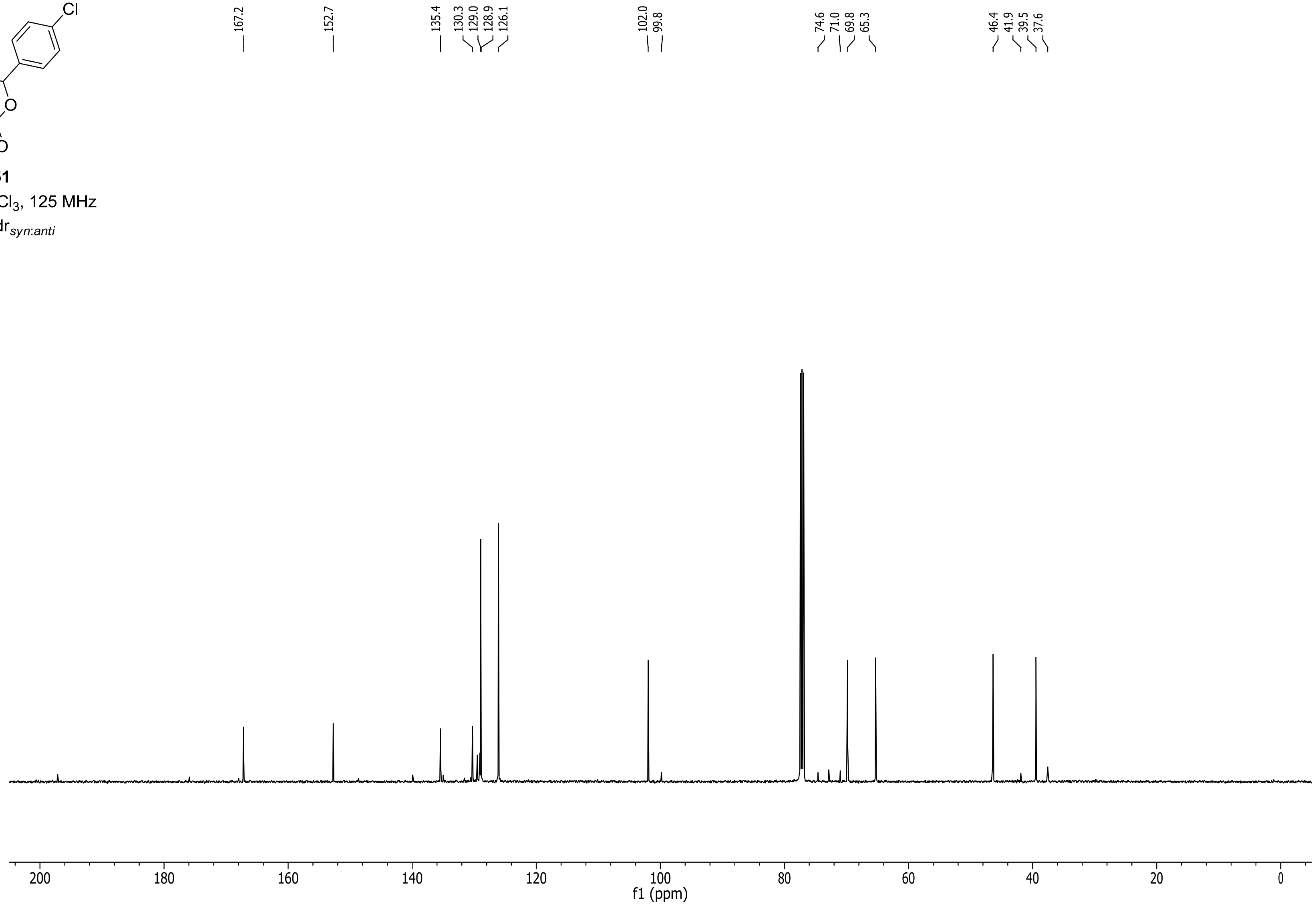

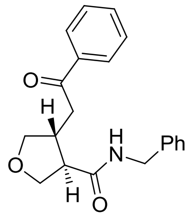

52

^{1}H, $CDCl_3$, 300 MHz

20:80 dr$_{syn:anti}$

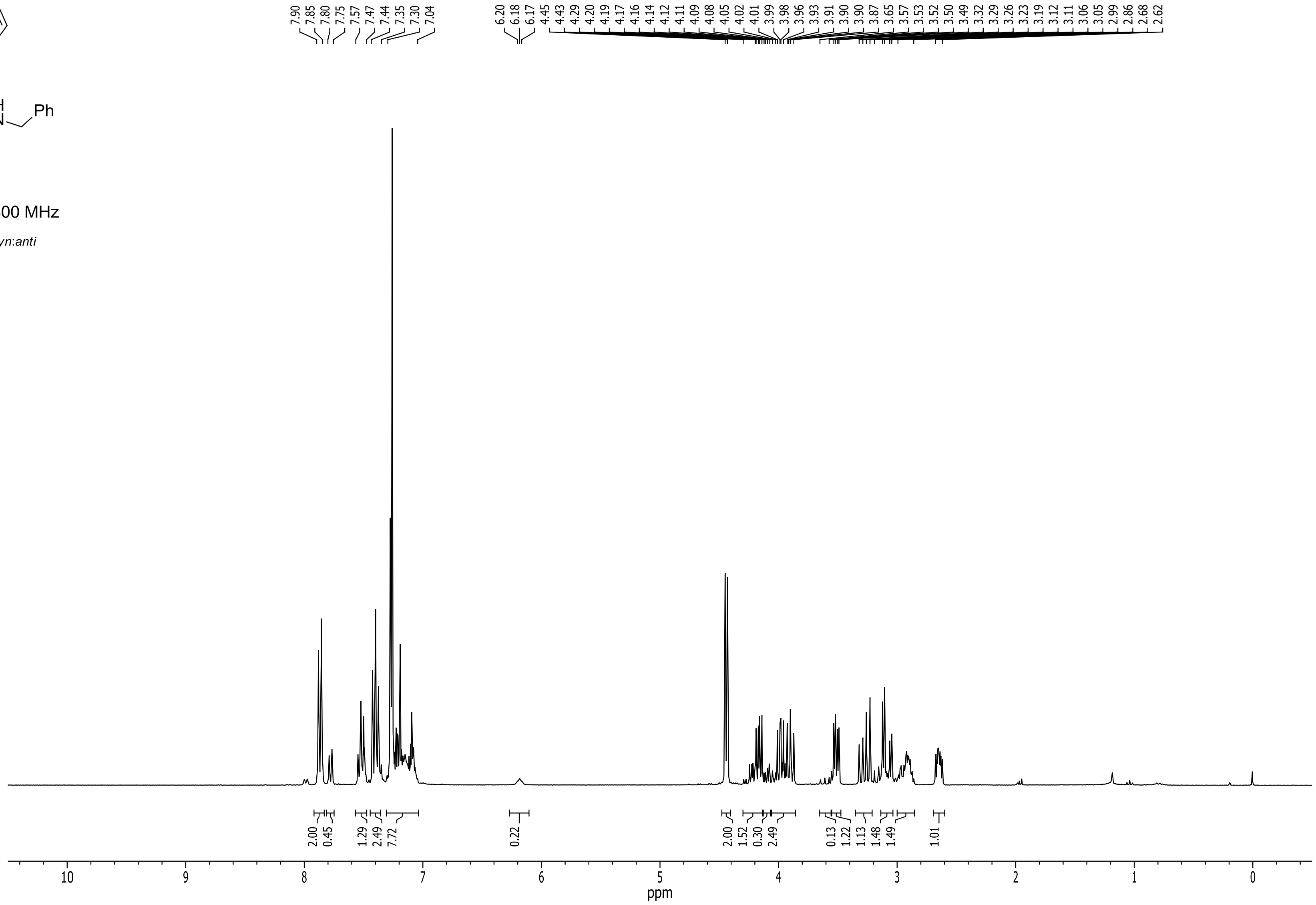

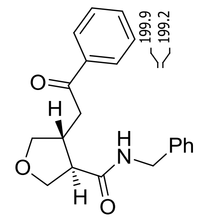

52

$^{13}C\{^1H\}$, $CDCl_3$, 75 MHz

20:80 $dr_{syn:anti}$

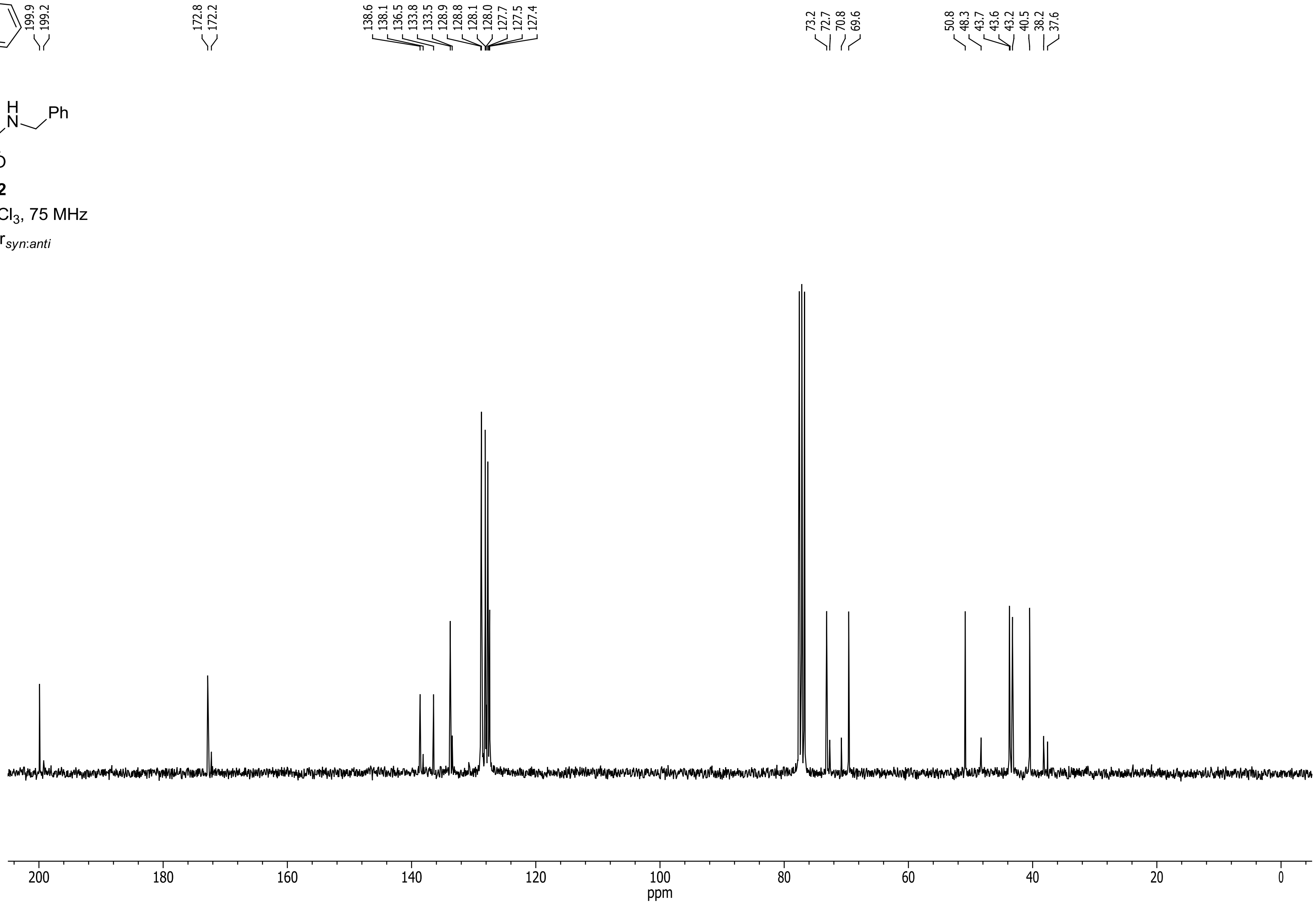

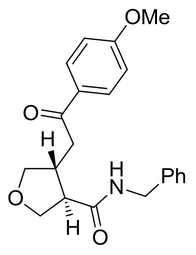

53

^{1}H, $CDCl_3$, 300 MHz

10:90 $dr_{syn:anti}$

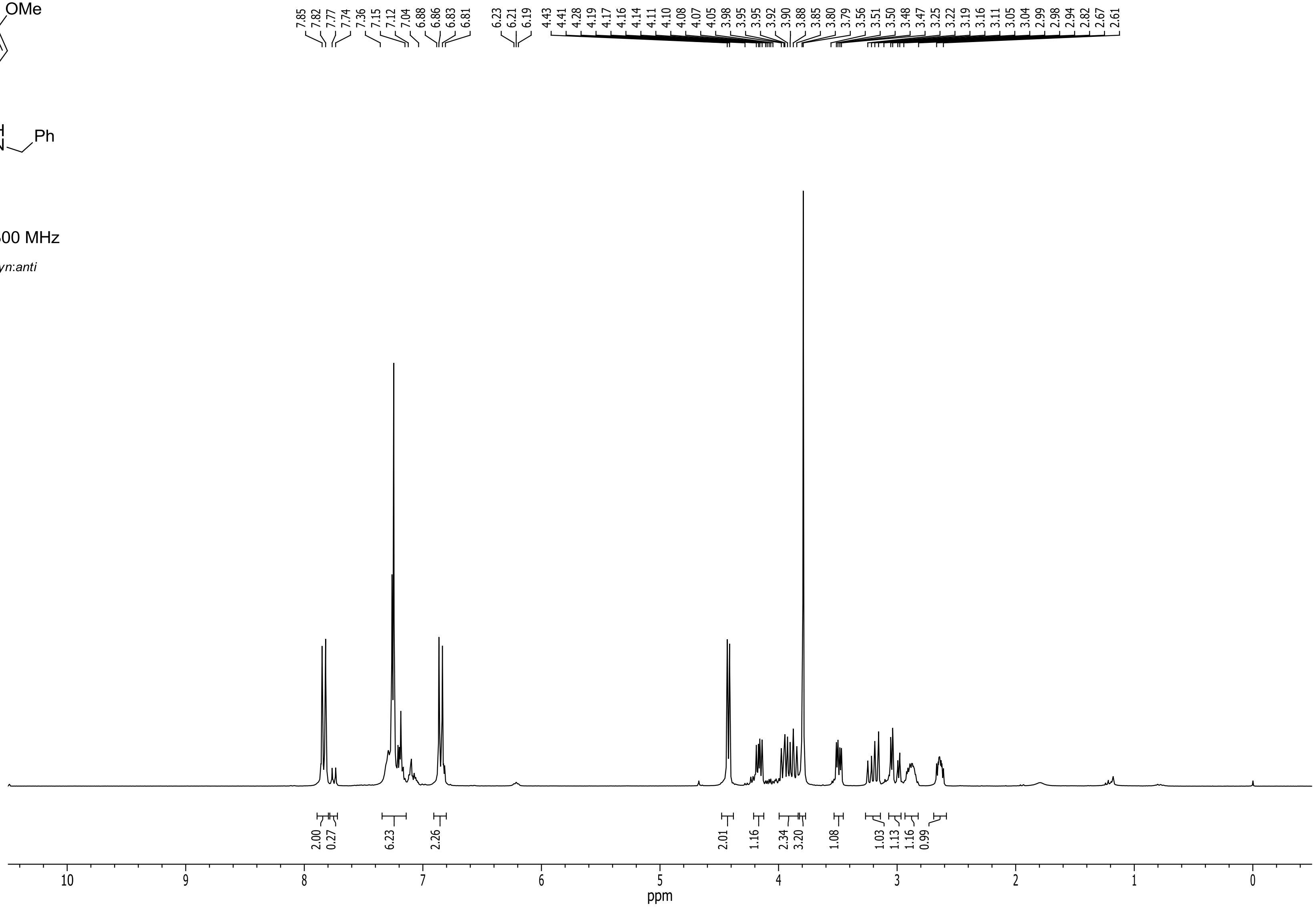

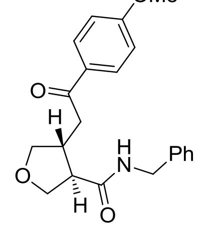

53

$^{13}C\{^1H\}$, $CDCl_3$, 75 MHz

10:90 $dr_{syn:anti}$

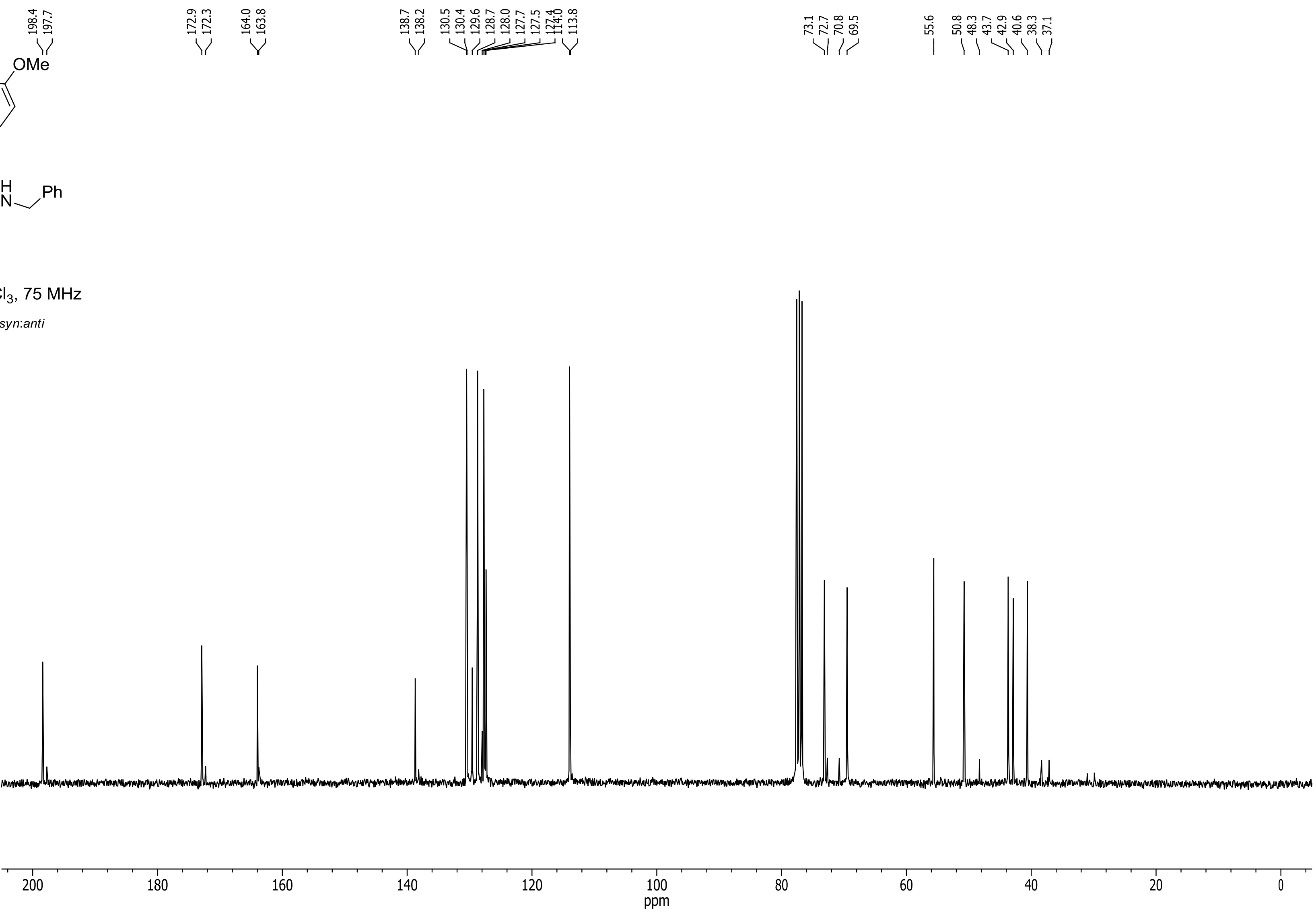

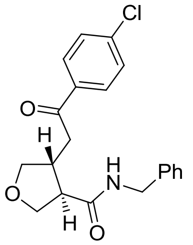

54

^{1}H, $CDCl_3$, 300 MHz

>1:99 $dr_{syn:anti}$

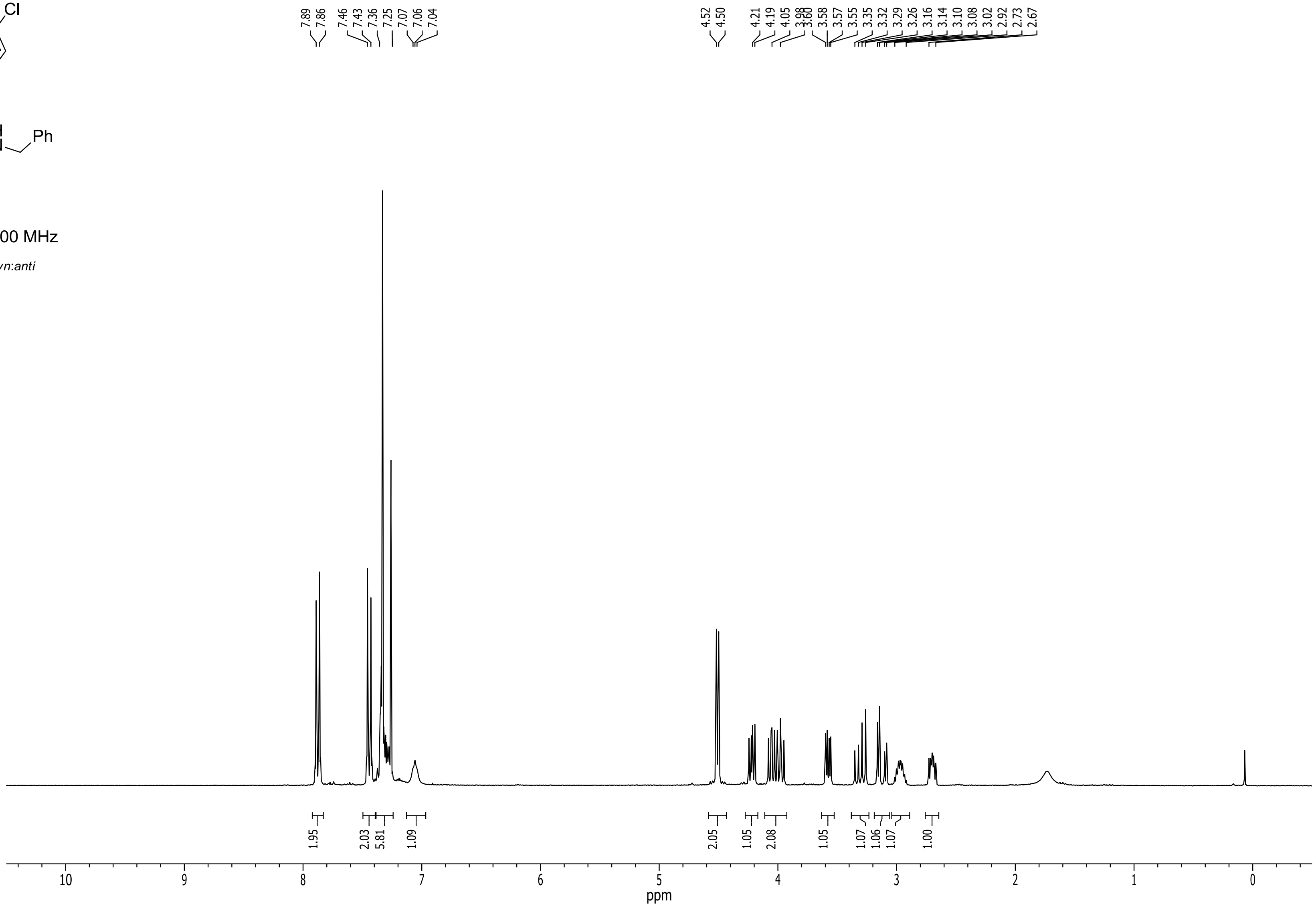

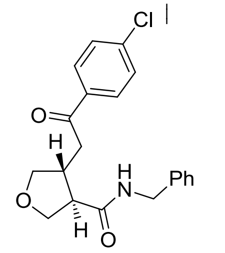

54

$^{13}C\{^{1}H\}$, $CDCl_3$, 75 MHz

>1:99 $dr_{syn:anti}$

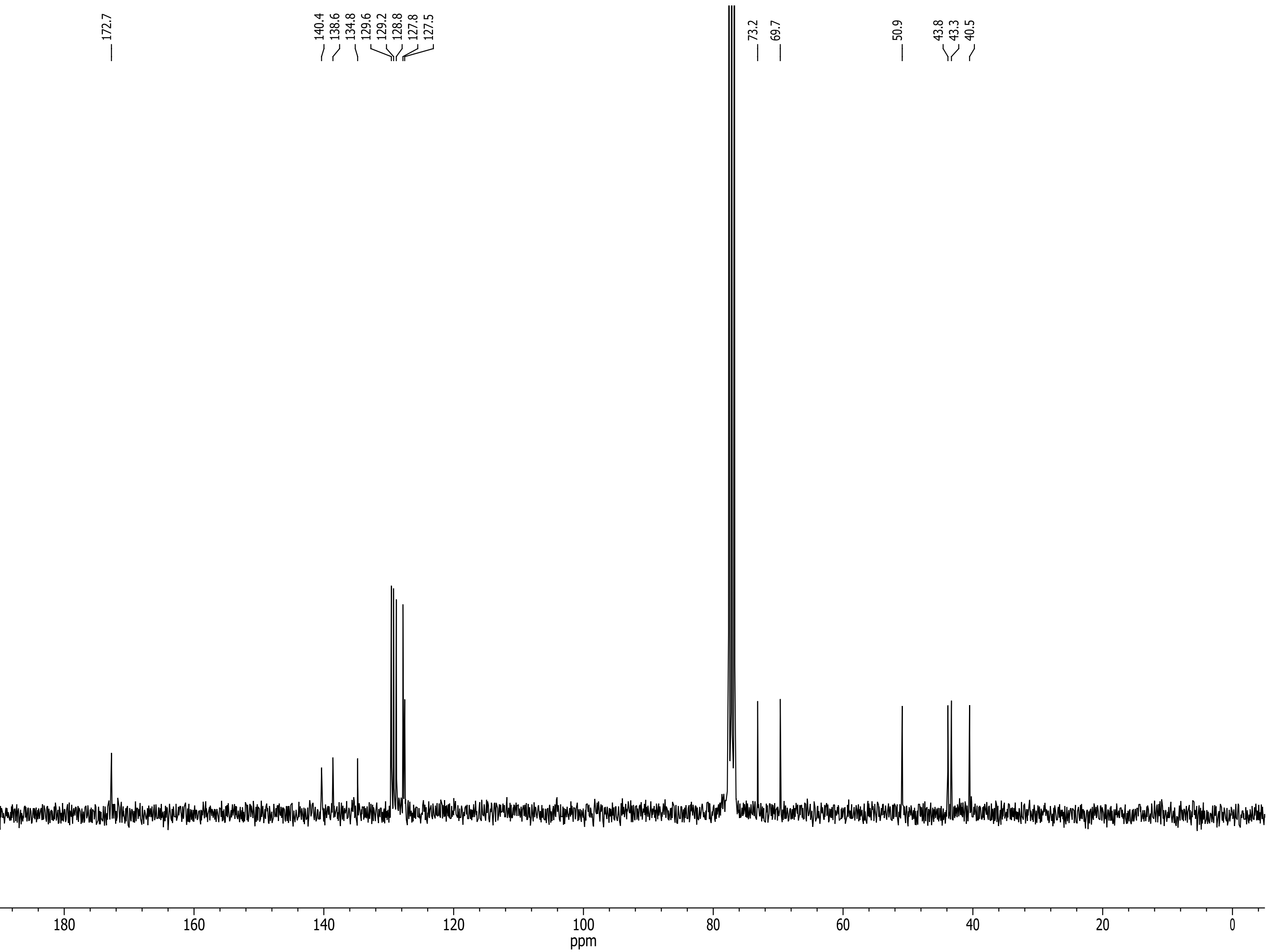

HPLC Data for *ent*-**10**: Chiralpak AD-H (85:15 hexane : IPA, flow rate 1.0 mLmin^{-1}, 254 nm) t_R (2*R*,3*S*): 14.8 min, t_R (2*S*,3*R*): 15.9 min, 94% ee.

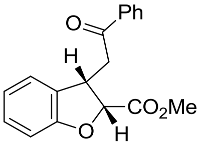

	Inj. Number	Peak Name	R. Time	Area	Area %
1	1.00	*1	14.62	2897787.75	49.62
	1.00	2	15.75	2942107.00	50.38

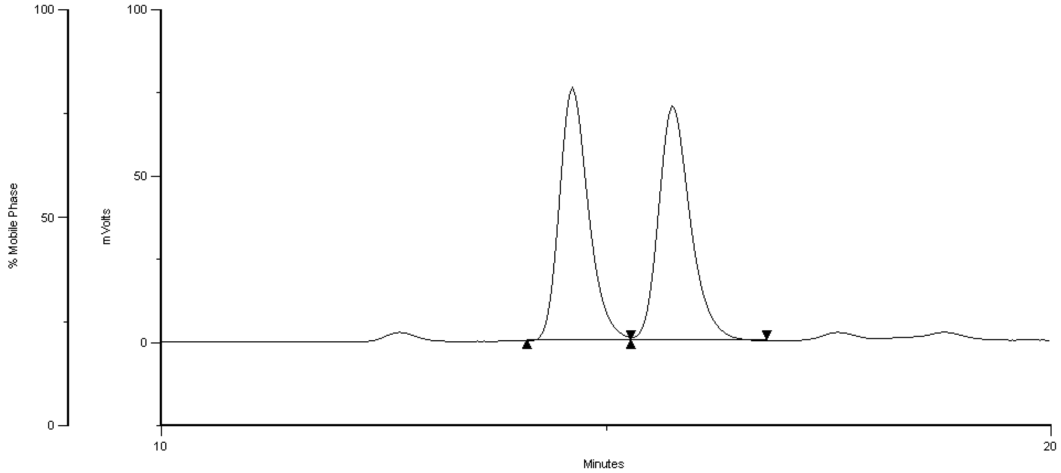

	Inj. Number	Peak Name	R. Time	Area	Area %
1	1.00	*1	14.78	3080689.25	96.89
	1.00	2	15.98	98759.45	3.11

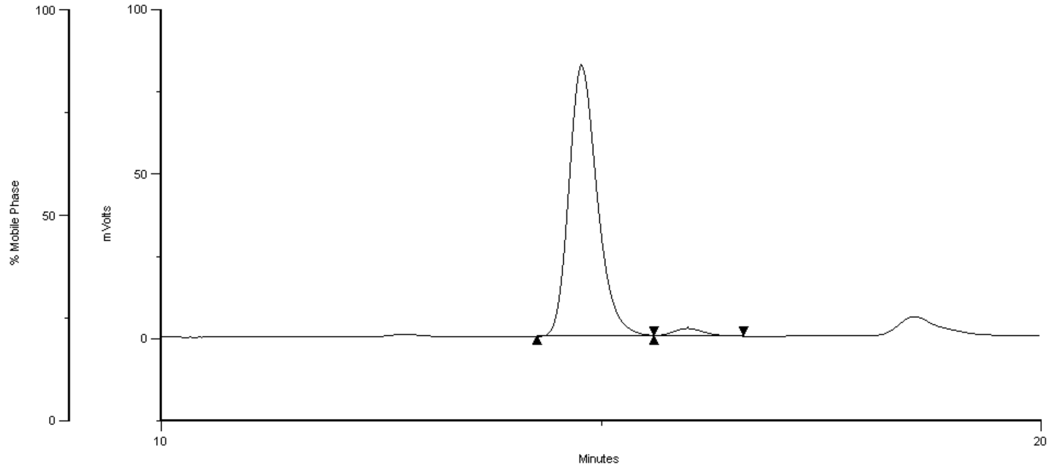

HPLC Data for **20**: Chiralpak AD-H (85:15 hexane : IPA, flow rate 0.5 mLmin^{-1}, 254 nm) t_R (2*R*,3*S*): 36.9 min, t_R (2*S*,3*R*): 42.9 min, 99% ee.

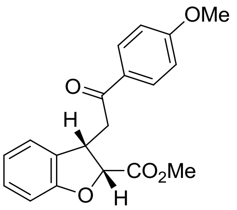

Peak Name	R. Time	Area	Area %
*1	37.67	73547736.00	51.82
2	43.27	68394632.00	48.18

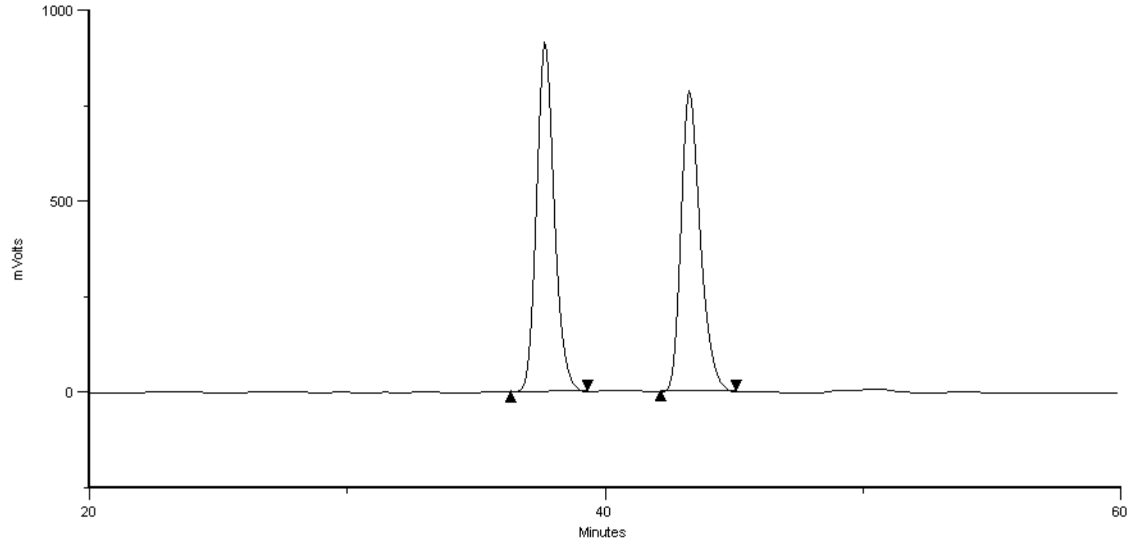

Peak Name	R. Time	Area	Area %
*1	36.88	36513188.00	99.73
*2	42.87	97704.79	0.27

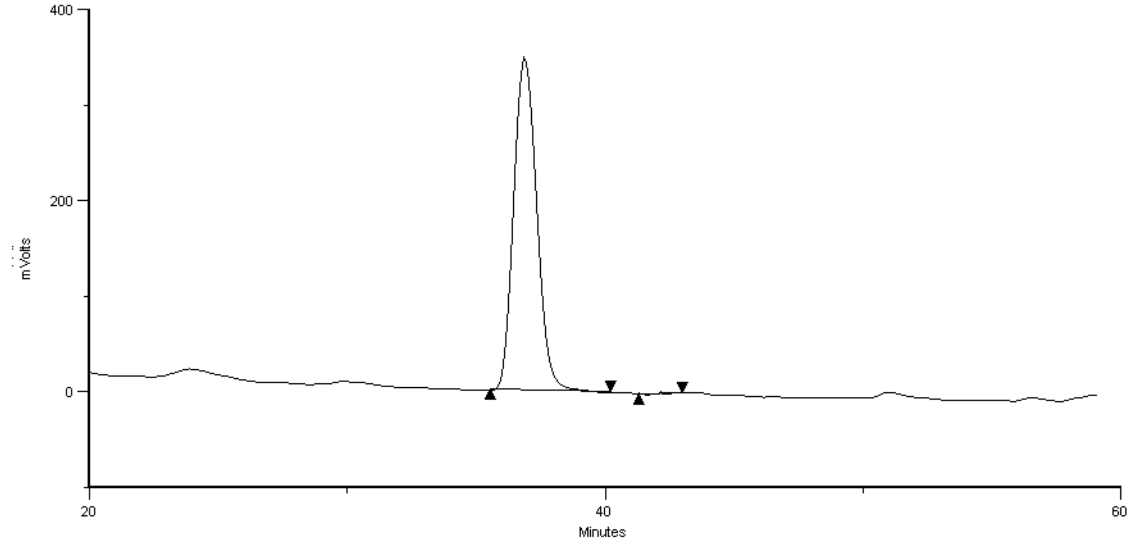

HPLC Data for **21**: Chiralpak AD-H (85:15 hexane : IPA, flow rate 1.0 mLmin^{-1}, 254 nm) t_R (2*S*,3*R*): 12.9 min, t_R (2*R*,3*S*): 14.9 min, 94% ee.

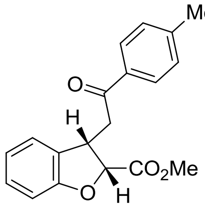

	Inj. Number	Peak Name	R. Time	Area	Area %
1	1.00	*1	12.92	20830320.00	49.40
2	1.00	2	15.02	21332738.00	50.60

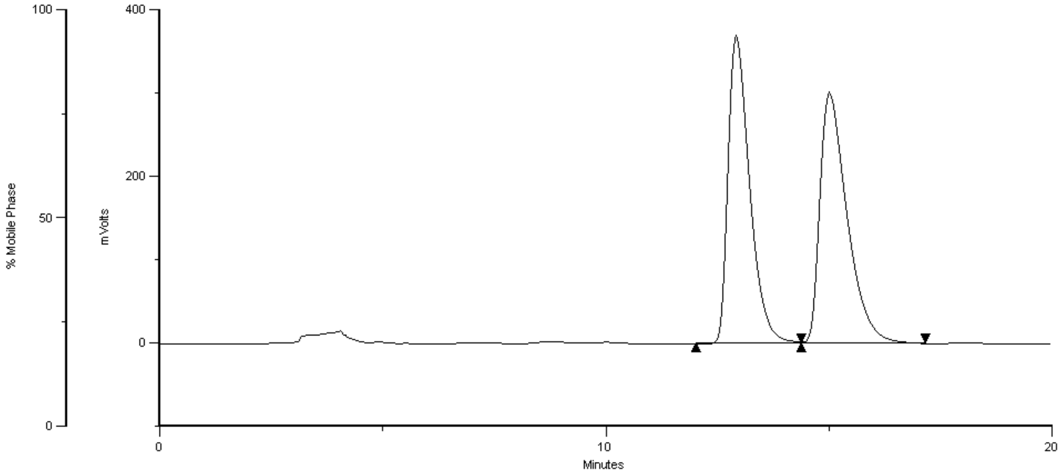

	Inj. Number	Peak Name	R. Time	Area	Area %
1	1.00	*1	12.92	632576.19	3.21
2	1.00	2	14.94	19045170.00	96.79

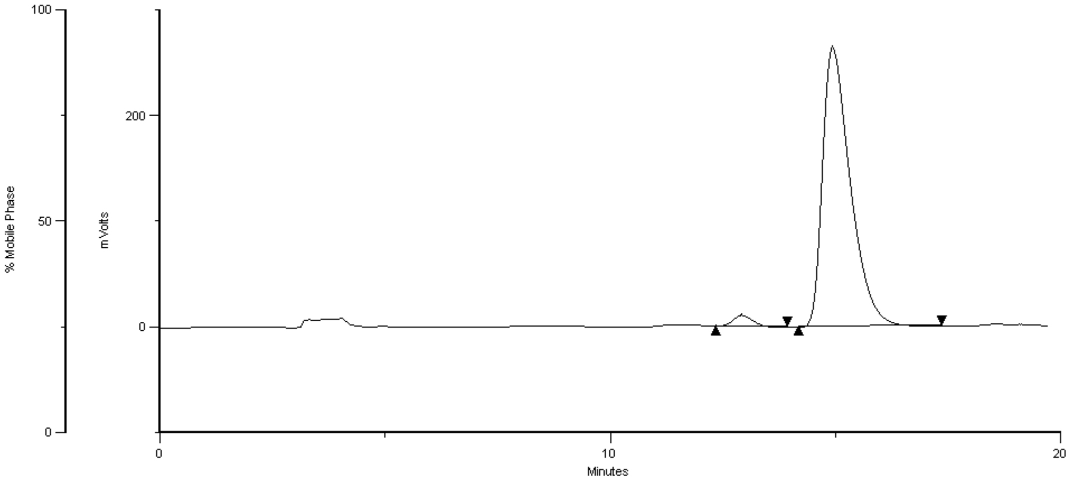

HPLC Data for **22**: Chiralpak AD-H (97:3 hexane : IPA, flow rate 1.0 mLmin^{-1}, 211 nm, 30 °C) t_R (2*R*,3*S*): 27.2 min, t_R (2*S*,3*R*): 30.5 min, 94% ee.

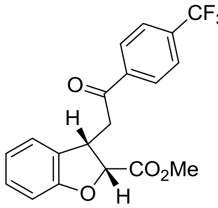

Peak Name	R. Time	Area	Area %
*1	27.63	41324440.00	50.82
2	31.13	39986568.00	49.18

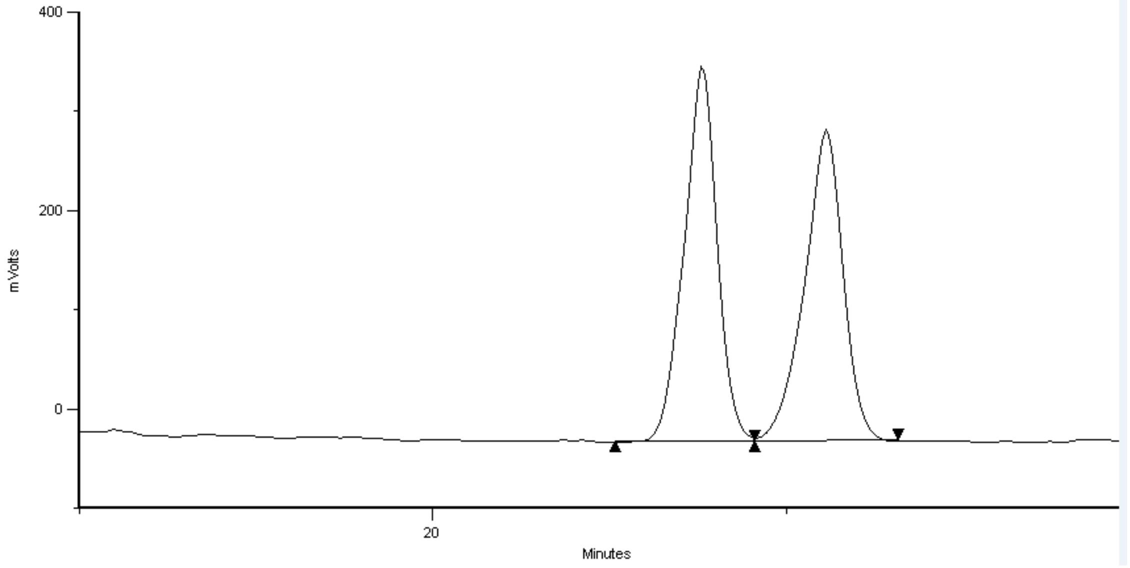

Peak Name	R. Time	Area	Area %
*1	27.15	1716296.75	2.81
2	30.49	59434236.00	97.19

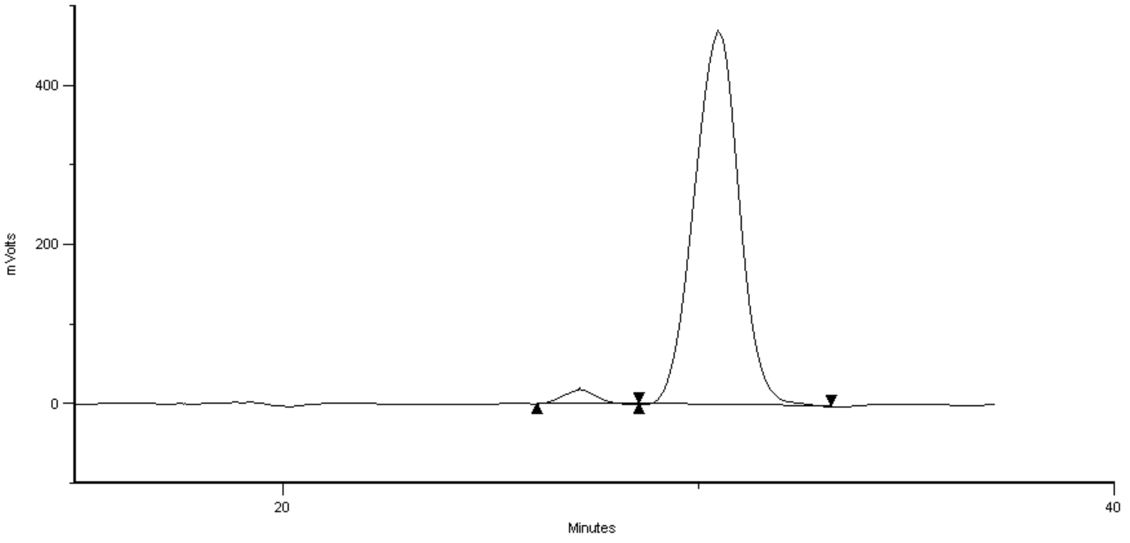

HPLC Data for **23**: Chiralpak AD-H (85:15 hexane : IPA, flow rate 1.0 mLmin^{-1}, 254 nm) t_R (2*R*,3*S*): 15.9 min, t_R (2*S*,3*R*): 19.2 min, 85% ee.

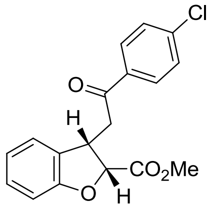

	Inj. Number	Peak Name	R. Time	Area	Area %
1	1.00	*1	15.97	11173199.00	49.51
2	1.00	*2	19.41	11395758.00	50.49

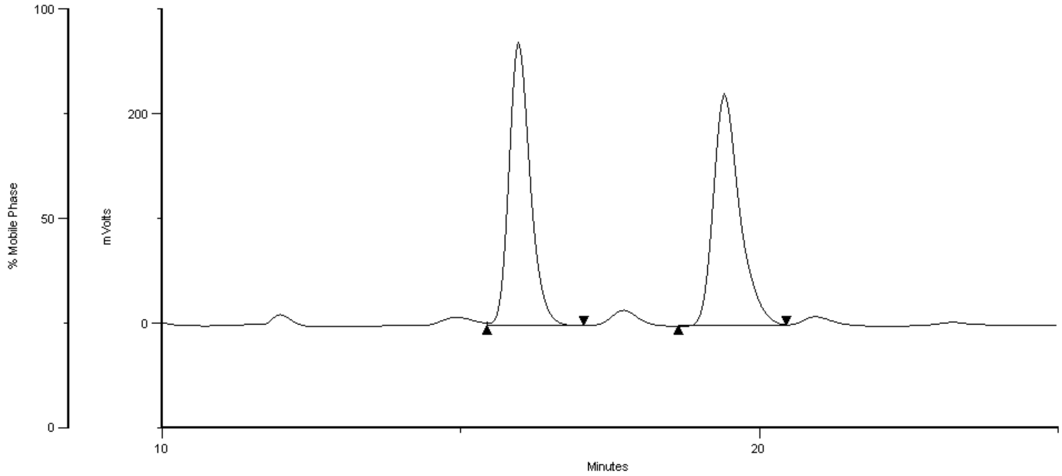

	Inj. Number	Peak Name	R. Time	Area	Area %
1	1.00	*1	15.89	7674463.50	92.33
	1.00	*2	19.24	637094.56	7.67

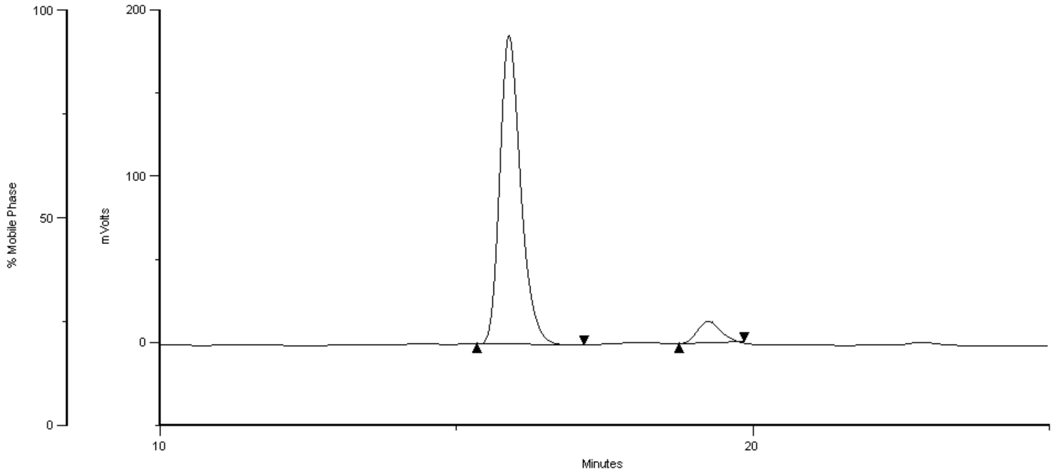

HPLC Data for **24**: Chiralpak AD-H (85:15 hexane : IPA, flow rate 1.0 mLmin^{-1}, 254 nm) t_R (2*S*,3*R*): 16.4 min, t_R (2*R*,3*S*): 18.2 min, 95% ee.

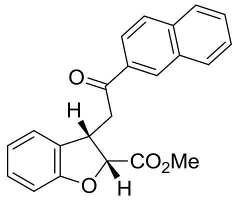

Pic	Rt (min)	Area (%)
1	16.88	41.83
2	18.94	42.43
3	23.33	7.40
4	27.55	8.33

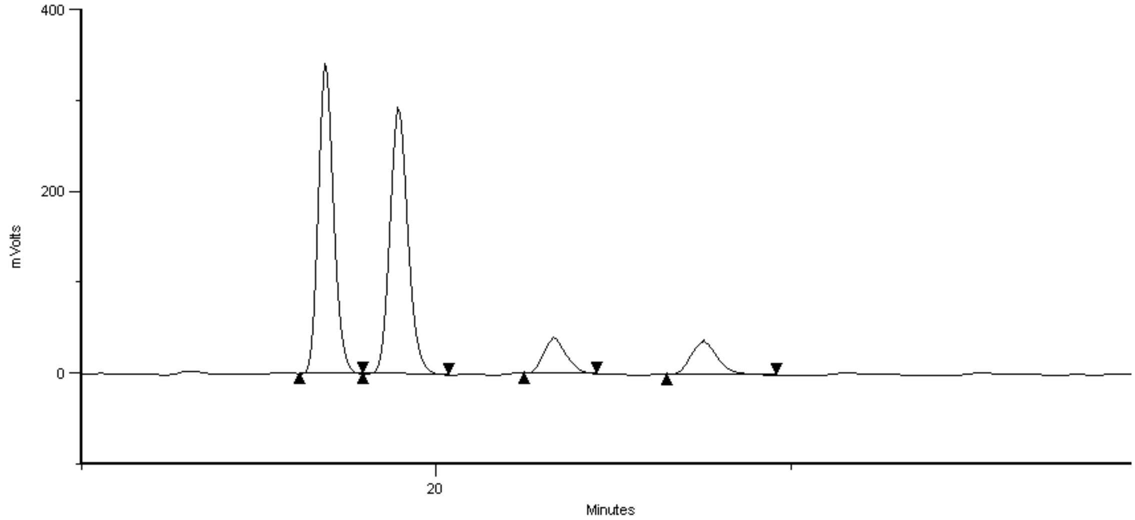

Pic	Rt (min)	Area (%)
1	16.36	2.42
2	18.24	97.58

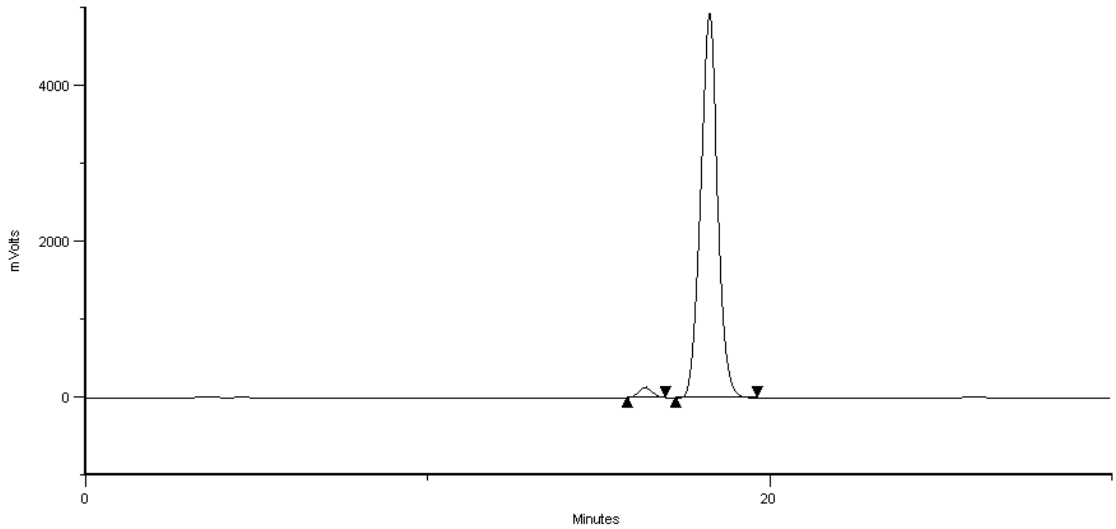

HPLC Data for **25**: Chiralpak AD-H (85:15 hexane : IPA, flow rate 1.0 mLmin^{-1}, 254 nm) t_R (2*S*,3*R*): 7.8 min, t_R (2*R*,3*S*): 8.8 min, 95% ee.

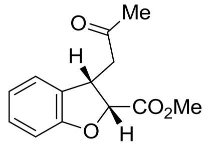

	Inj. Number	Peak Name	R. Time	Area	Area %
1	1.00	1	7.28	708000.88	52.09
	1.00	2	8.33	651271.69	47.91

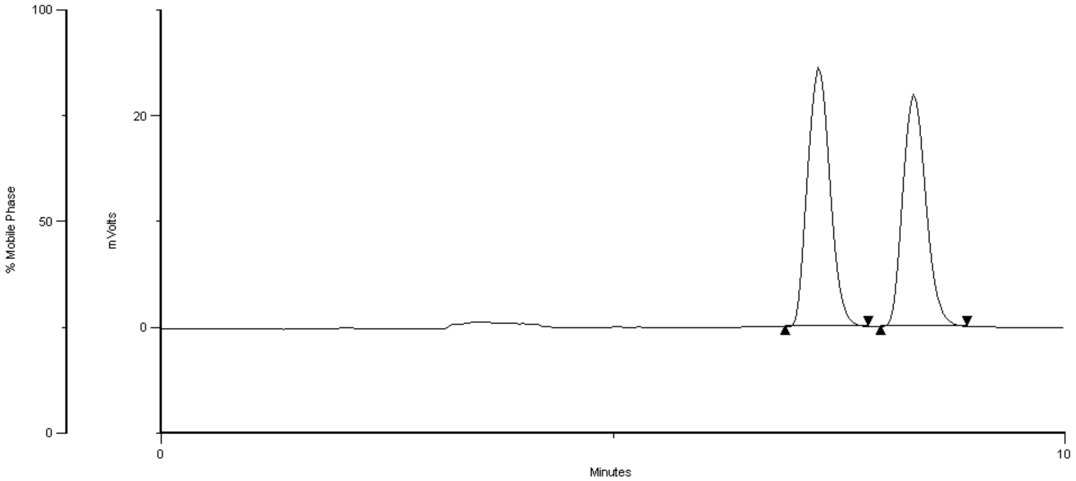

	Inj. Number	Peak Name	R. Time	Area	Area %
1	1.00	1	7.79	34705.00	2.46
2	1.00	2	8.83	1375978.25	97.54

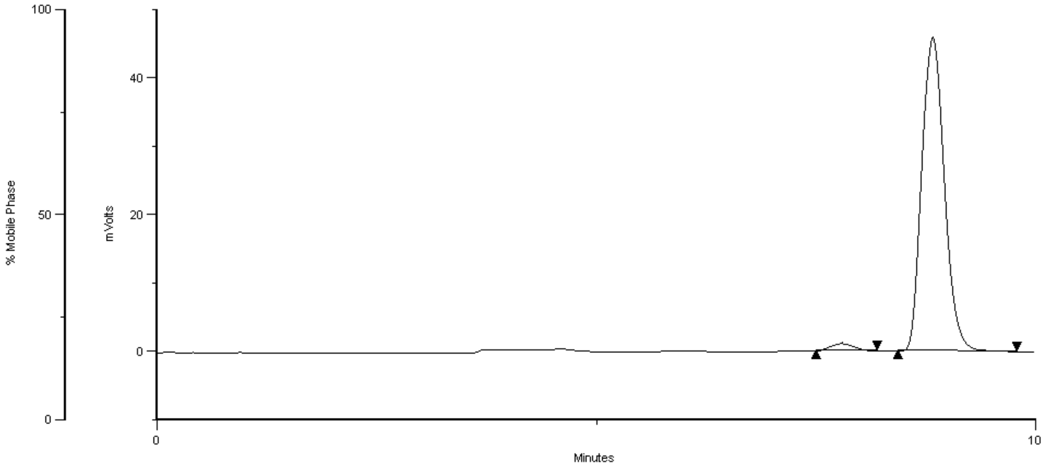

HPLC Data for **11**: Chiralpak AD-H (85:15 hexane : IPA, flow rate 1.0 mLmin^{-1}, 254 nm, 30 °C) t_R (2*R*,3*R*): 15.1 min, t_R (2*S*, 3*S*): 20.4 min, 98% ee

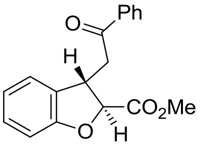

Peak	t_R(min)	Area (%)
1	12.63	31.40
2	14.64	19.25
3	16.93	31.82
4	21.46	17.53

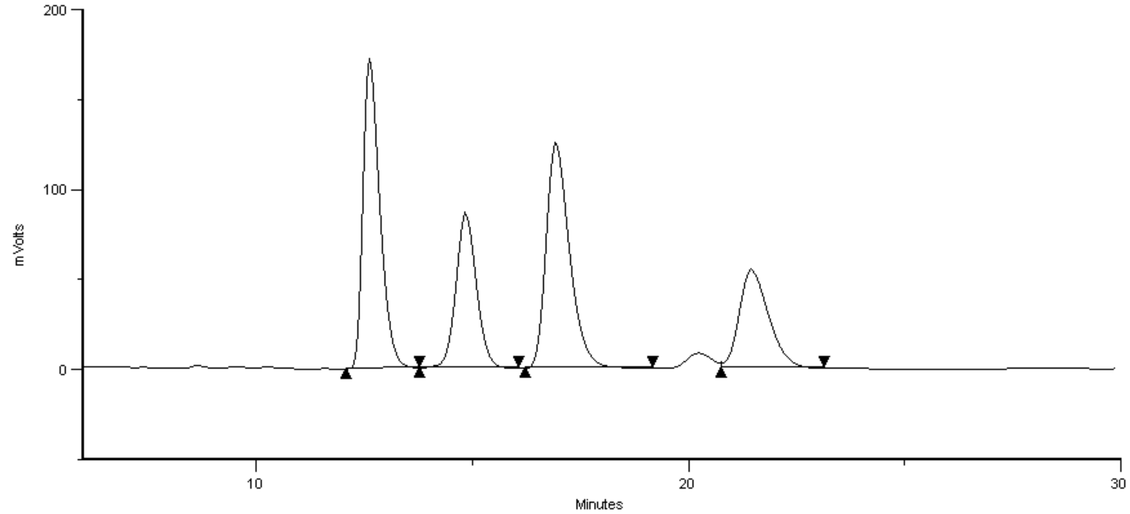

Peak	t_R (min)	Area (%)
1	15.15	1.05
2	20.36	98.95

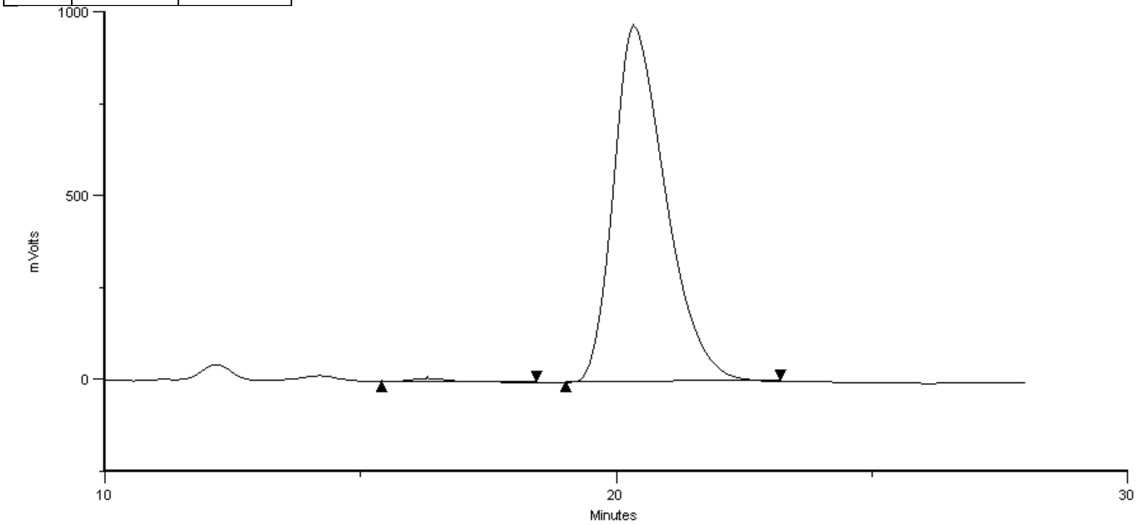

HPLC Data for **10**: Chiralpak AD-H (85:15 hexane : IPA, flow rate 1.0 mLmin^{-1}, 254 nm, 30 °C) t_R (2*S*, 3*R*): 12.4 min, t_R (2*R*, 3*S*) 16.7 min, 63% ee.

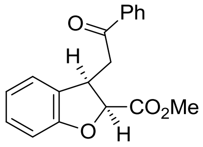

Peak	t_R (min)	Area (%)
1	13.12	50.28
2	16.70	49.72

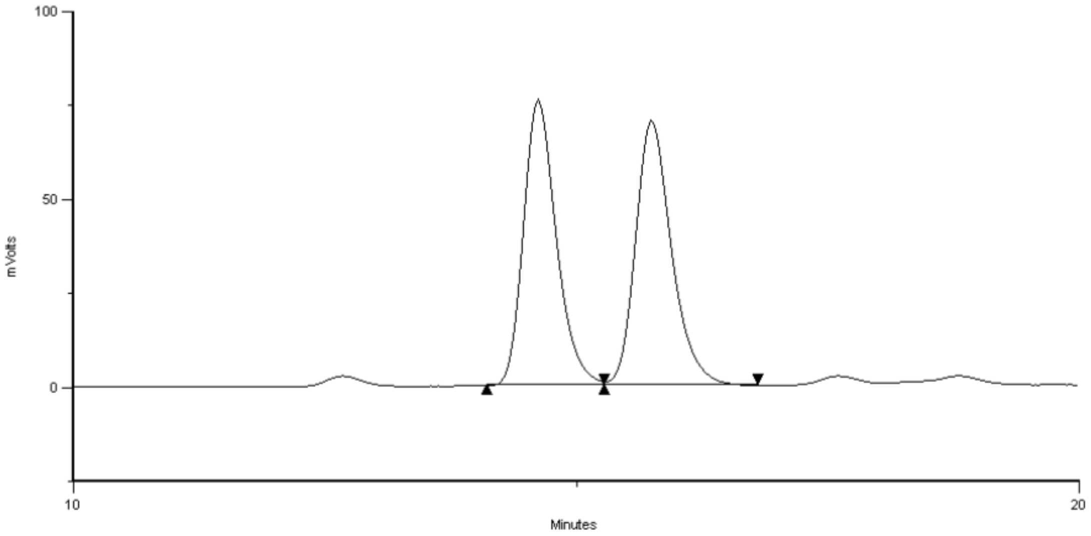

Peak	t_R(min)	Area (%)
1	12.42	81.40
2	16.66	18.60

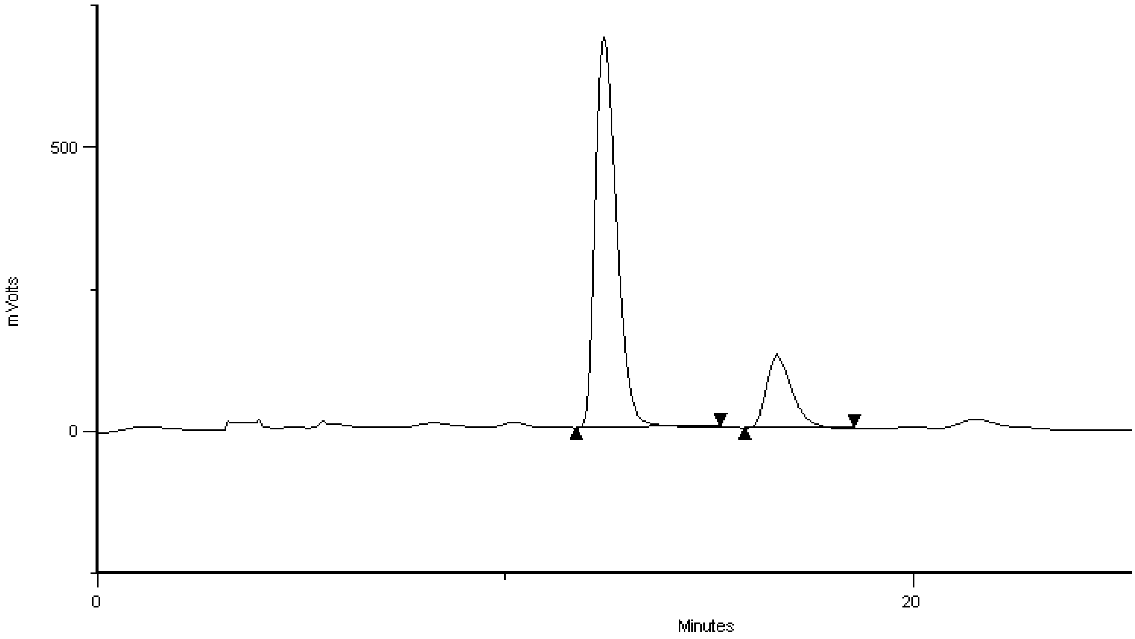

HPLC Data for **26**: Chiralpak AD-H (85:15 hexane : IPA, flow rate 0.5 mLmin^{-1}, 254 nm, 30 °C) t_R (2*R*,3*R*): t_R (2*S*,3*S*): 48.5 min, t_R (2*R*,3*R*): 53.3 min, 99% ee.

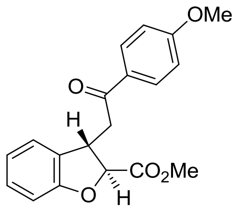

Peak	t_R(min)	Area (%)
1	47.93	50.04
2	52.92	49.96

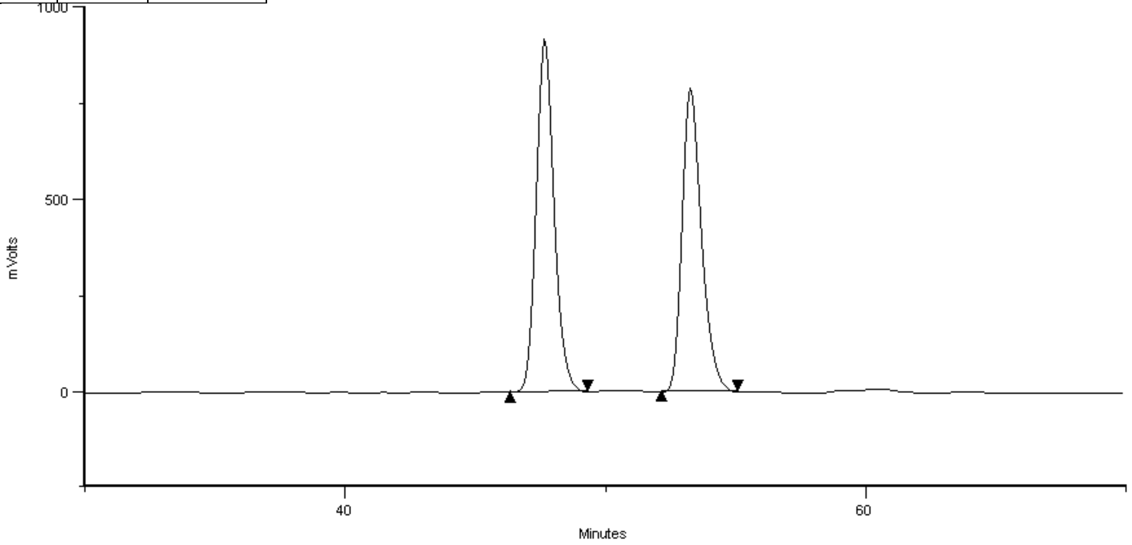

Peak	t_R (min)	Area (%)
1	48.55	99.3
2	53.33	0.70

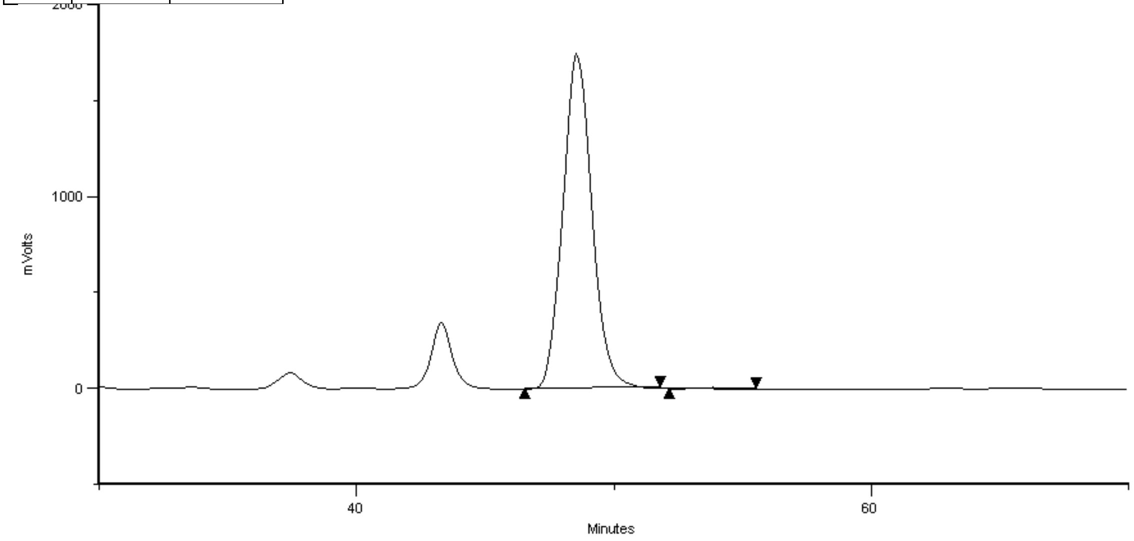

HPLC Data for *ent*-**20**: Chiralpak AD-H (85:15 hexane : IPA, flow rate 0.5 mLmin^{-1}, 254 nm, 30 °C) t_R (2*R*,3*S*): 37.1 min, t_R (2*S*,3*R*): 42.8 min, 51% ee.

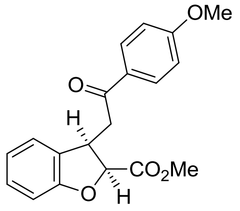

Peak	t_R (min)	Area (%)
1	37.67	51.82
2	43.27	48.18

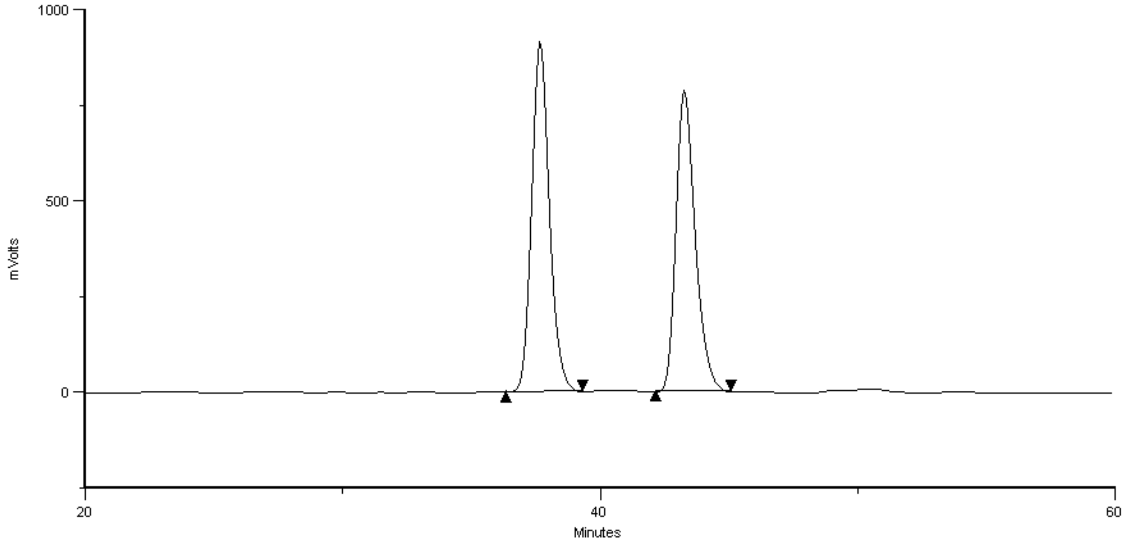

Peak	t_R (min)	Area (%)
1	37.07	24.65
2	42.78	75.35

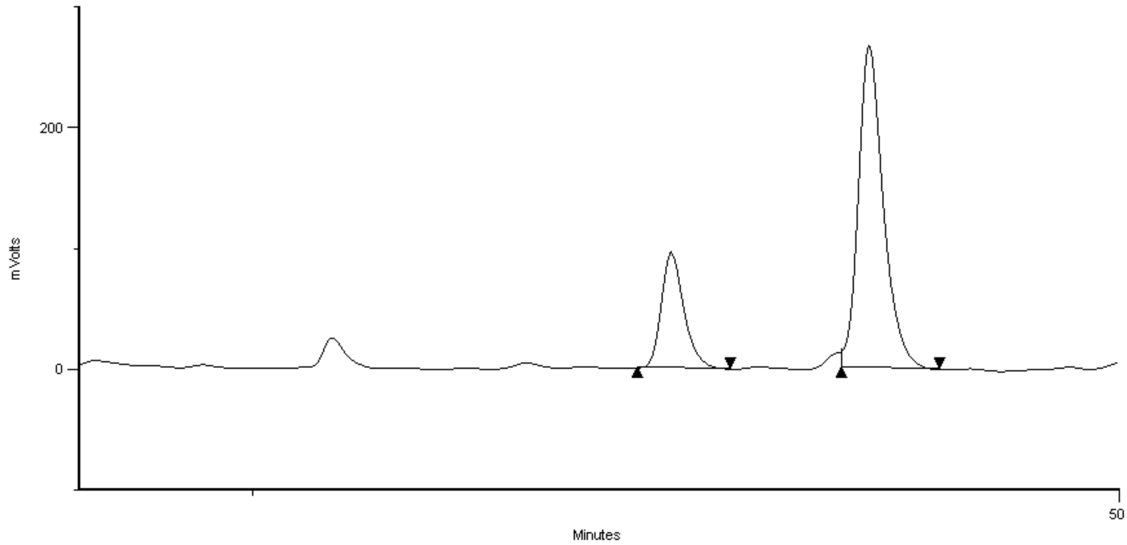

HPLC Data for **27**: Chiralpak AD-H (85:15 hexane : IPA, flow rate 0.5 mLmin^{-1}, 254 nm, 30 °C) t_R (2*R*,3*R*): 29.4 min, t_R (2*S*,3*S*): 32.0 min, 98% ee.

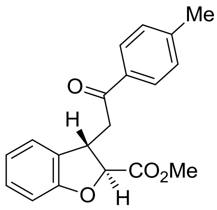

Peak	t_R(min)	Area (%)
1	26.14	19.27
2	29.07	18.58
3	31.47	32.32
4	33.72	29.83

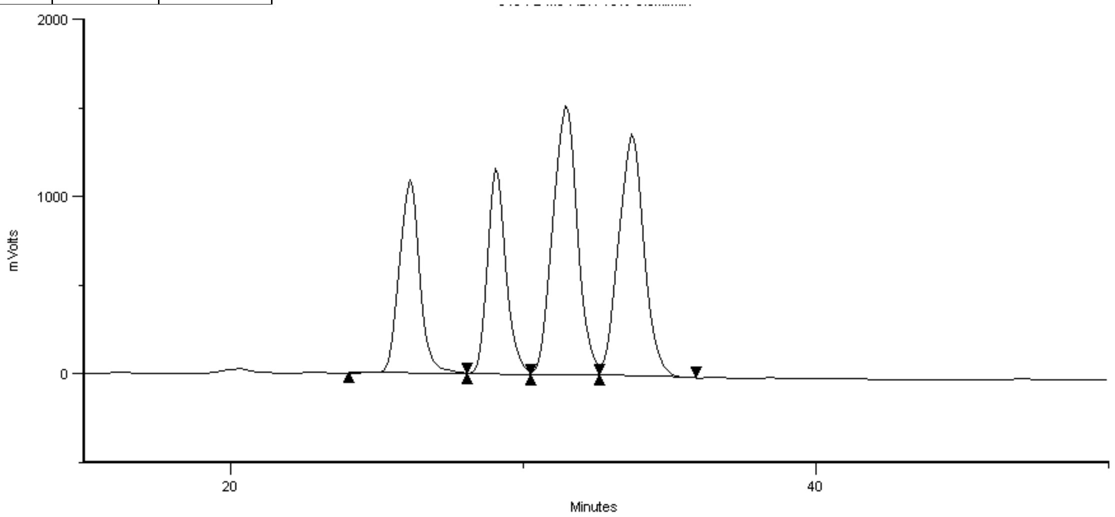

Peak	t_R (min)	Area (%)
1	29.38	0.98
2	32.02	99.11

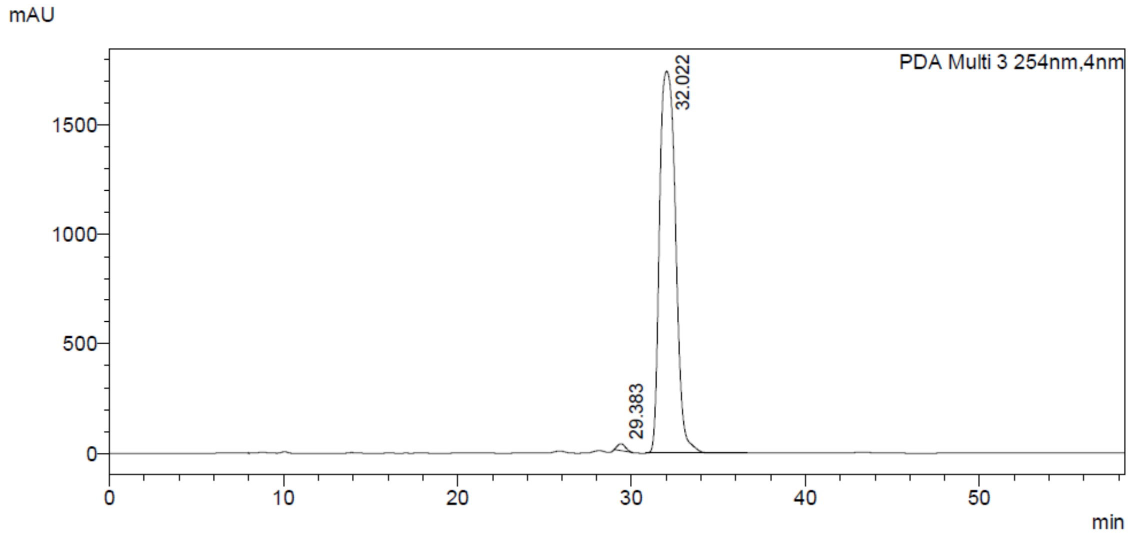

HPLC Data for *ent*-**21**: Chiralpak AD-H (85:15 hexane : IPA, flow rate 0.5 mLmin^{-1}, 240 nm, 30 °C) t_R (2*R*,3*S*): 25.4 min, t_R (2*S*,3*R*): 28.1 min, 63% ee.

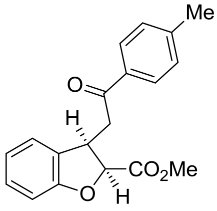

Peak	t_R(min)	Area (%)
1	26.53	50.79
2	29.14	49.21

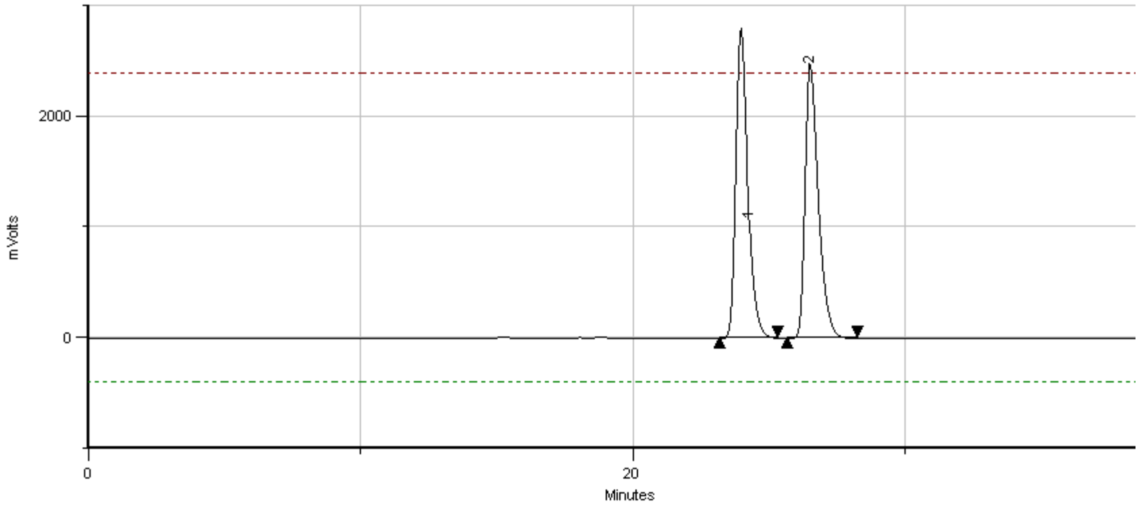

Peak	t_R (min)	Area (%)
1	25.42	18.36
2	28.14	81.64

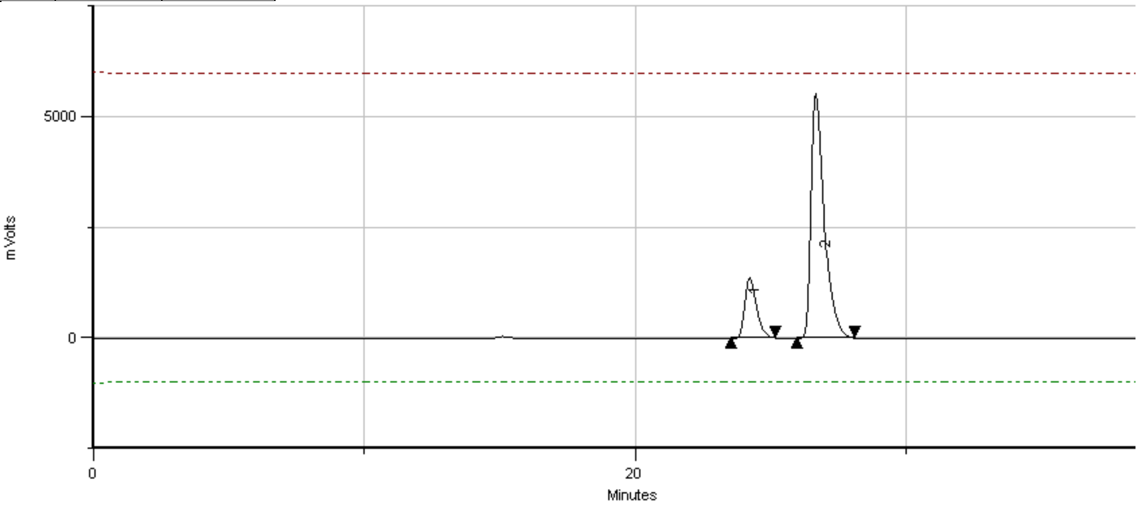

HPLC Data for **28**: Chiralpak AD-H (90:10 hexane : IPA, flow rate 1.0 mLmin^{-1}, 254 nm, 30 °C) t_R (2*S*,3*S*): 19.1 min, t_R (2*R*,3*R*): 21.7 min, 94% ee.

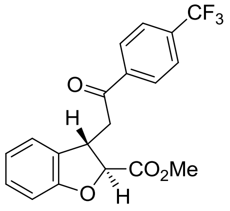

Peak	t_R(min)	Area (%)
1	19.11	40.92
2	21.34	50.08

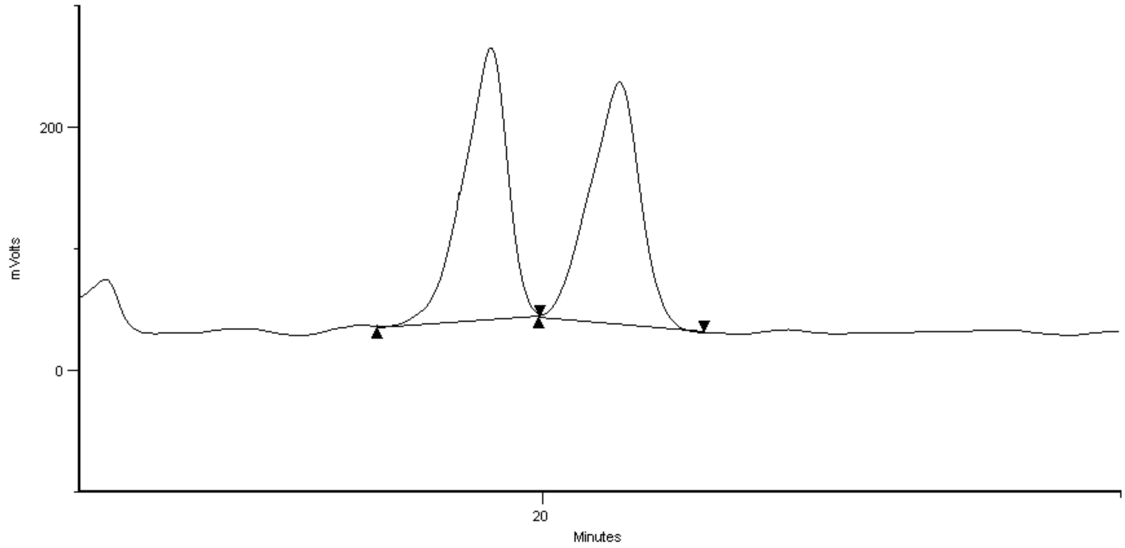

Peak	t_R (min)	Area (%)
1	19.08	96.94
2	21.71	3.06

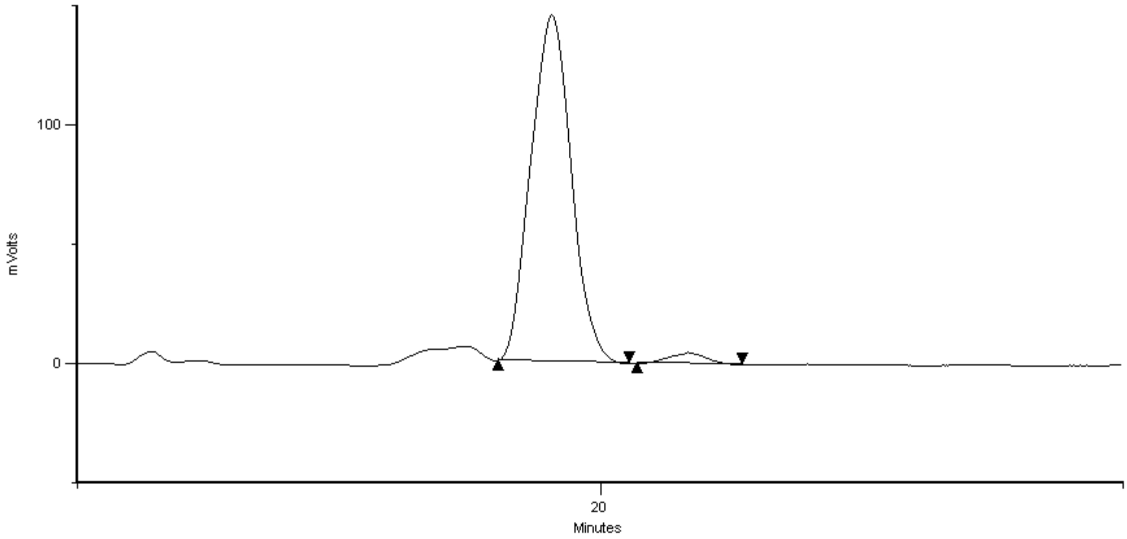

HPLC Data for *ent*-**22**: Chiralpak AD-H (97:3 hexane : IPA, flow rate 1.0 mLmin^{-1}, 254 nm, 30 °C) t_R (2*S*,3*R*): 27.4 min, t_R (2*R*, 3*S*): 31.1 min, 48% ee.

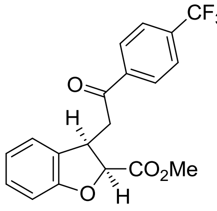

Peak	t_R(min)	Area (%)
1	27.63	50.82
2	31.13	49.18

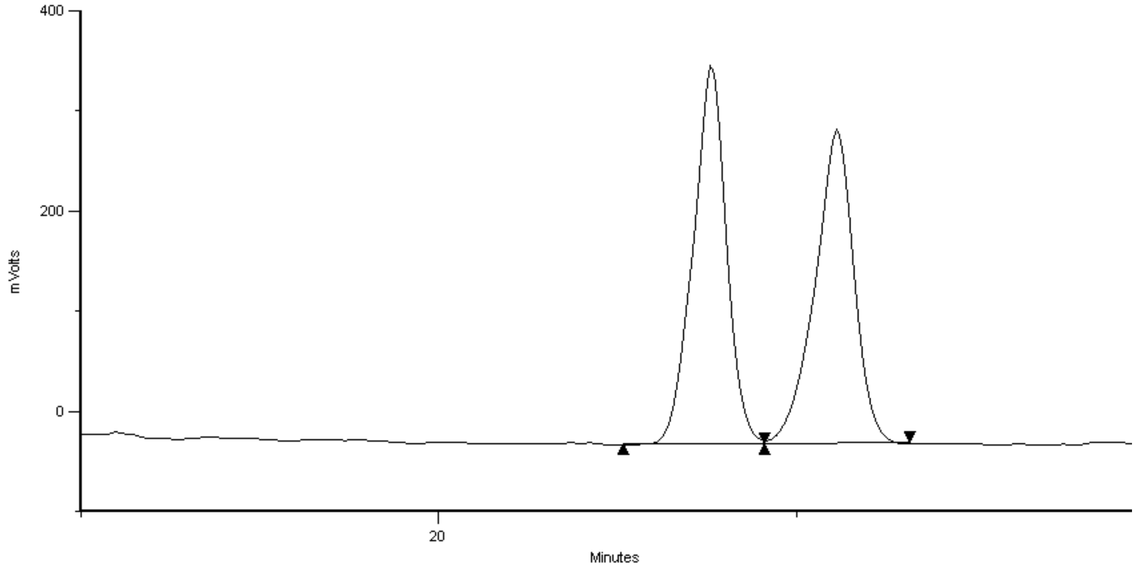

Peak	t_R(min)	Area (%)
1	27.49	74.02
2	31.09	25.98

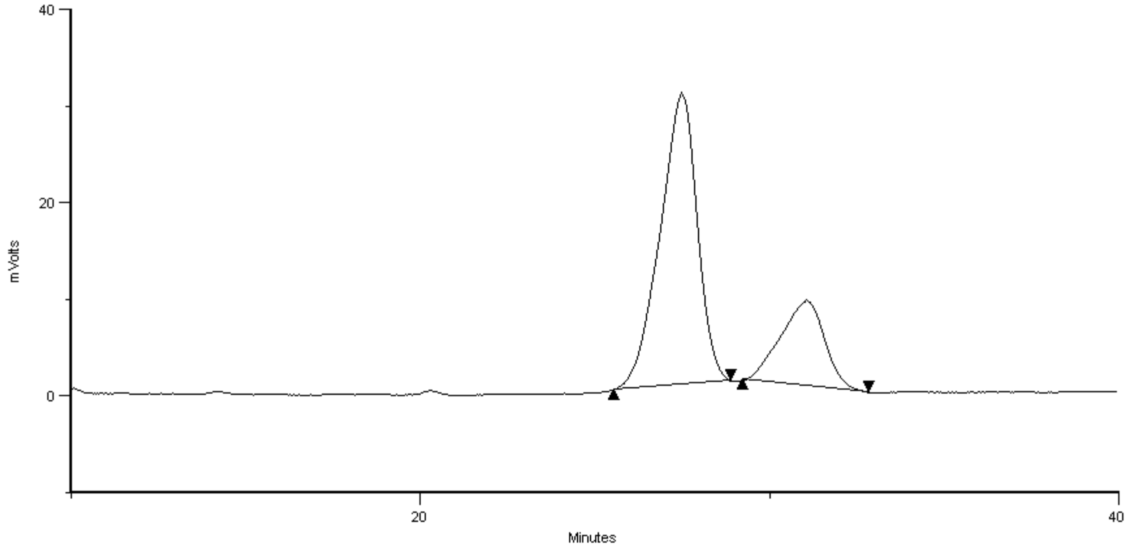

HPLC Data for **29**: Chiralpak AD-H (85:15 hexane : IPA, flow rate 0.5 mLmin^{-1}, 254 nm, 30 °C) t_R (2*S*,3*S*): 34.3 min, t_R (2*R*,3*R*): 37.7min, 96% ee.

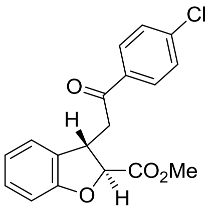

Peak	t_R(min)	Area (%)
1	27.47	15.74
2	32.39	14.62
3	34.28	35.55
4	37.55	34.09

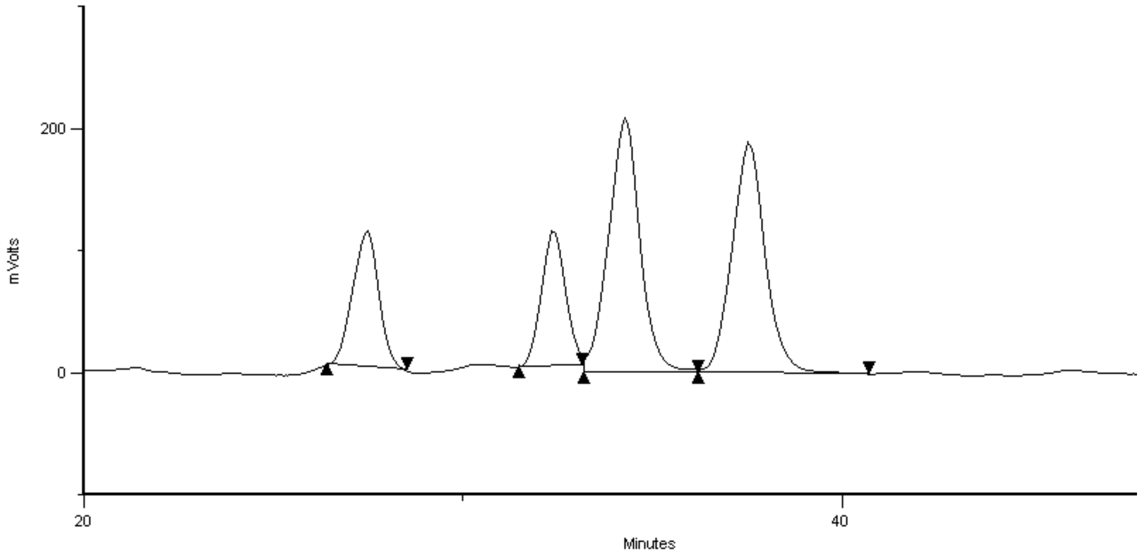

Peak	t_R (min)	Area (%)
1	34.31	98.18
2	37.75	1.82

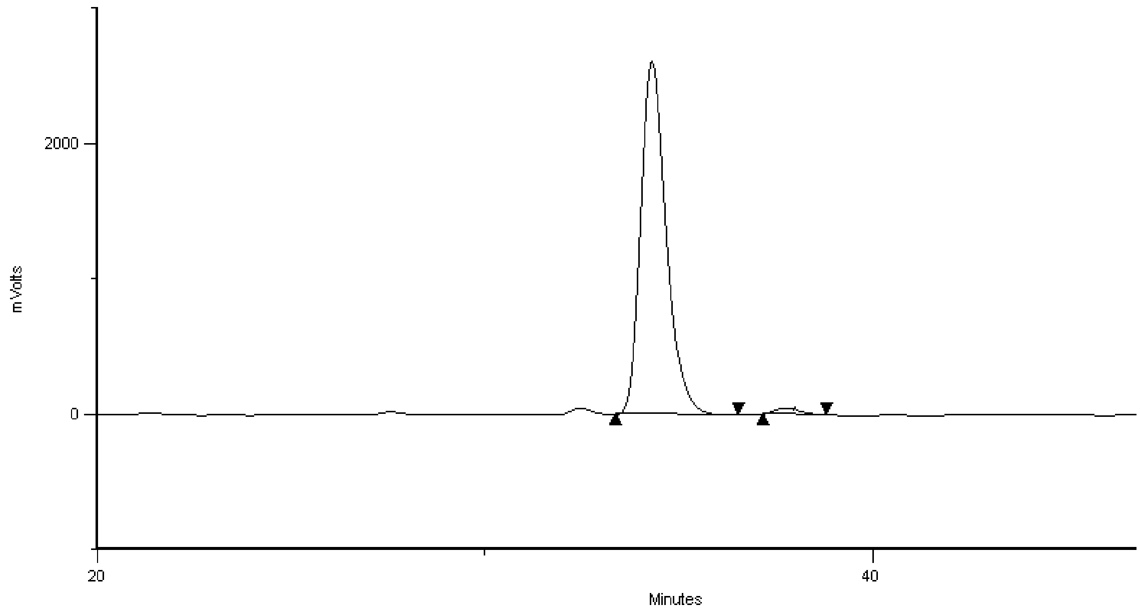

HPLC Data for *ent*-**23**: Chiral HPLC analysis, Chiralpak AD-H (85:15 hexane : IPA, flow rate 0.5 mLmin^{-1}, 254 nm, 30 °C) t_R (2*R*,3*S*): 27.6 min, t_R (2*S*,3*R*): 32.6 min, 42% ee.

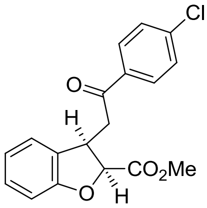

Peak	t_R(min)	Area (%)
1	27.47	15.74
2	32.39	14.62
3	34.28	35.55
4	37.55	34.09

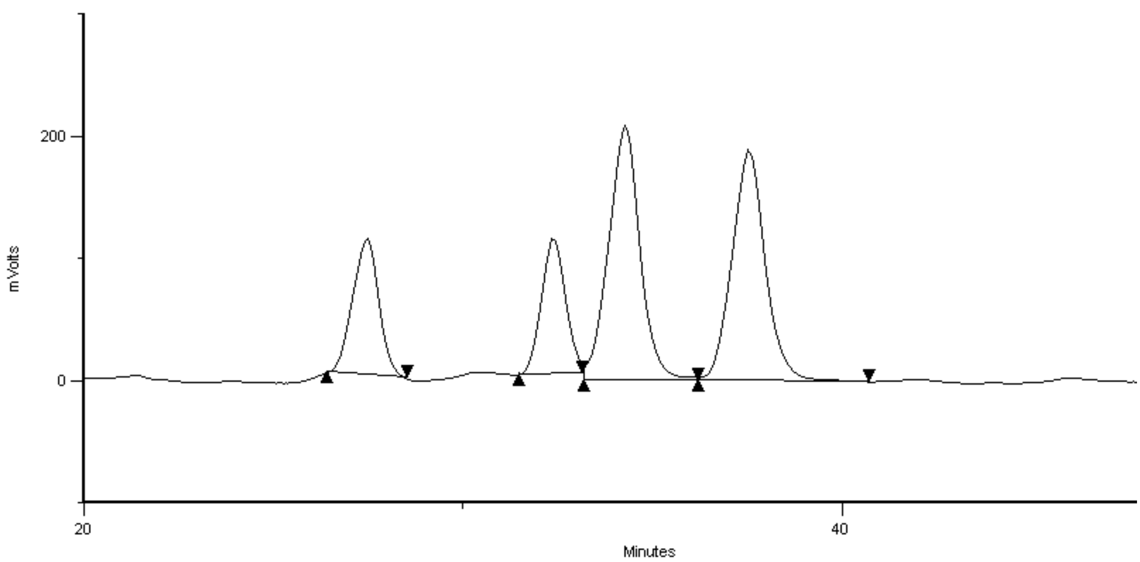

Peak	t_R(min)	Area (%)
1	27.66	28.76
2	32.63	71.24

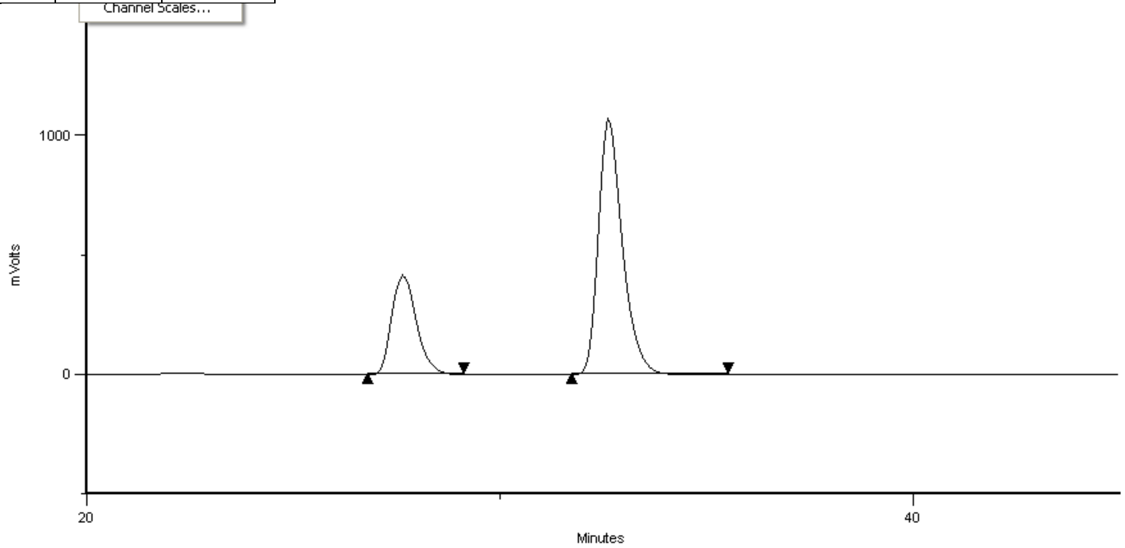

HPLC Data for **30**: Chiralpak AD-H (85:15 hexane : IPA, flow rate 1.0 mLmin^{-1}, 254 nm, 30 °C) t_R (2*S*,3*S*): 23.5 min, t_R (2*R*,3*R*): 27.6 min), 97% ee.

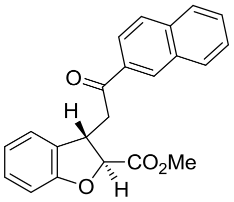

Peak	t_R (min)	Area (%)
1	16.81	5.27
2	18.94	4.59
3	23.33	44.40
4	27.33	45.74

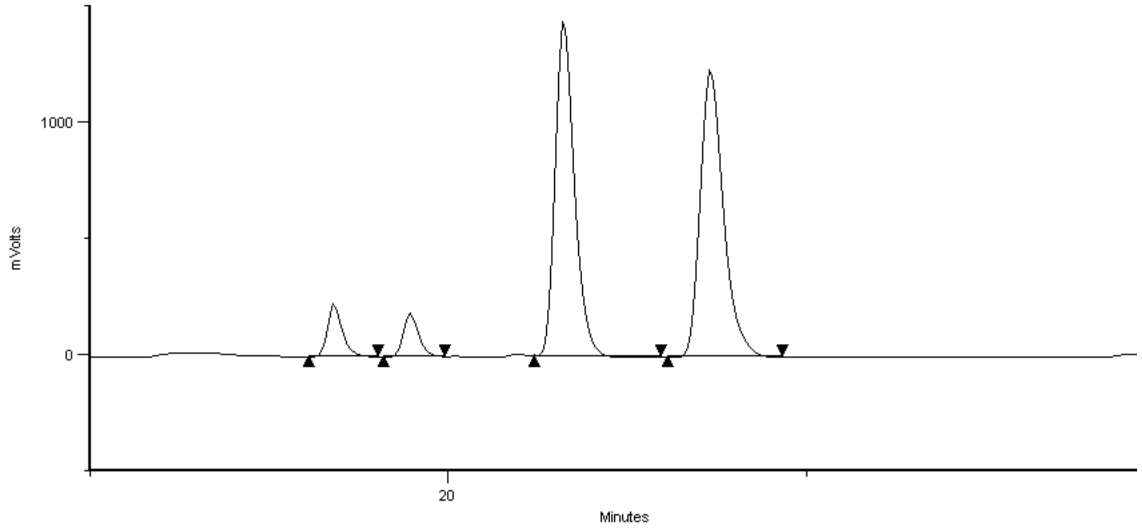

Peak	t_R (min)	Area (%)
1	23.55	98.46
2	27.67	1.54

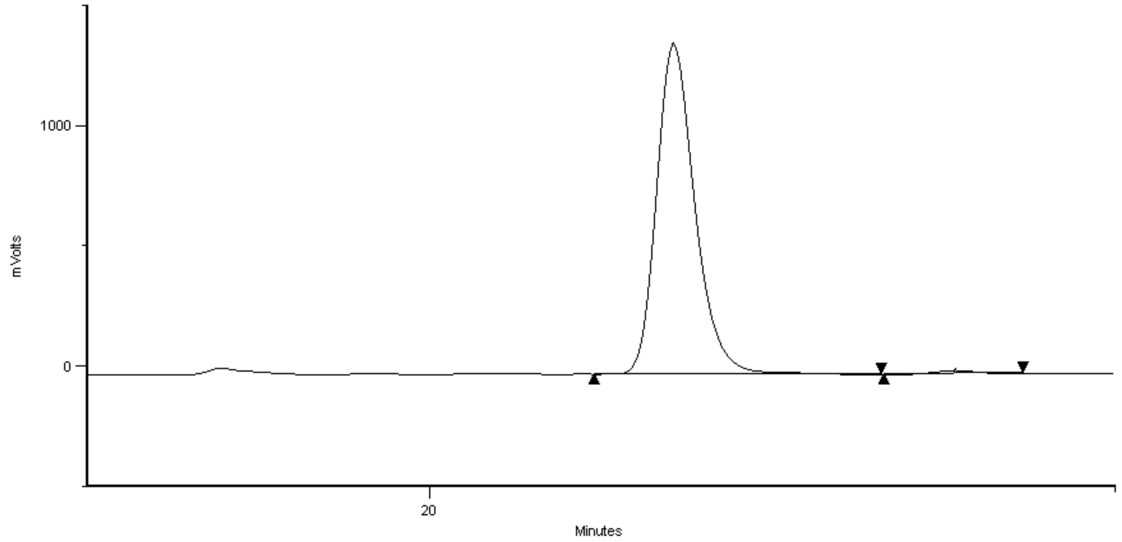

HPLC Data for *ent*-**24**: Chiralpak AD-H (85:15 hexane : IPA, flow rate 1.0 mLmin^{-1}, 254 nm, 30 °C) t_R (2*S*,3*R*): 16.8min, t_R (2*R*,3*S*): 18.9 min, 57% ee.

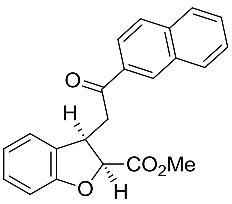

Peak	t_R (min)	Area (%)
1	16.88	41.83
2	18.94	42.43
3	23.33	7.40
4	27.55	8.33

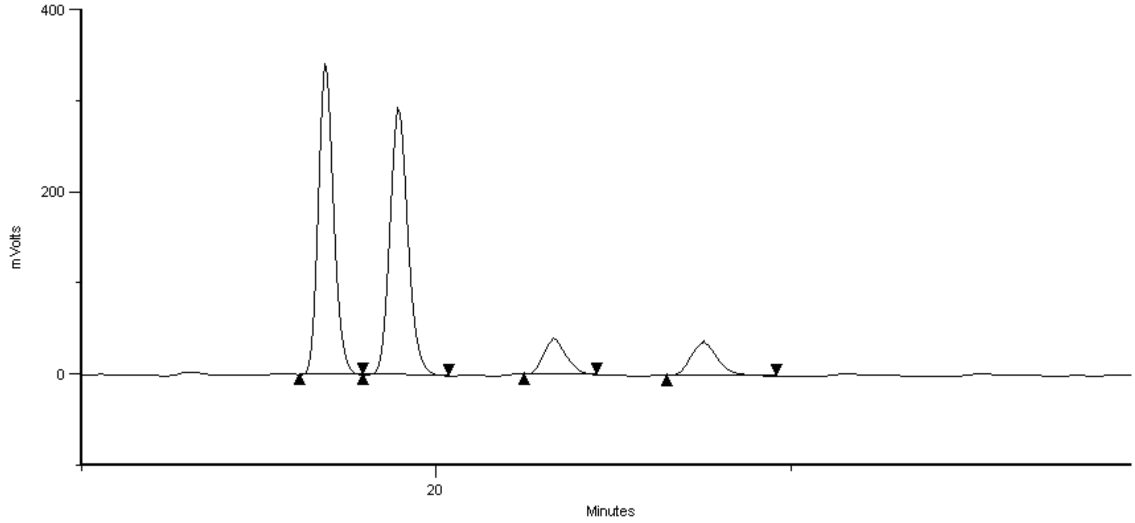

Peak	t_R (min)	Area (%)
1	16.84	78.29
2	18.95	21.71

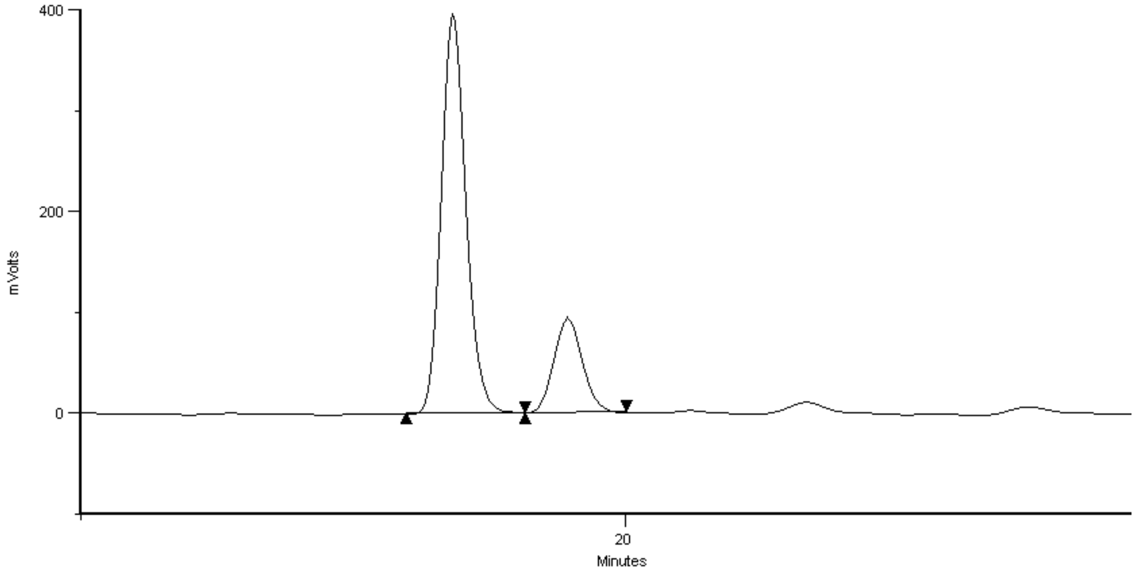

HPLC Data for **31** and *ent*-**25** Chiralpak AD-H (85:15 hexane : IPA, flow rate 1.0 mLmin^{-1}, 220 nm, 30 °C) **31**: t_R (2*S*,3*S*): 11.3 min, t_R (2*R*,3*R*): 12.0 min, 99% ee; *ent*-**25**: t_R (2*S*,3*R*): 7.35 min, t_R (2*R*,3*S*): 8.44 min, 43% ee.

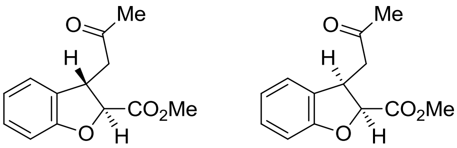

Peak	t_R (min)	Area (%)
1	7.35	31.56
2	8.42	32.94
3	11.21	17.60
4	12.08	17.90

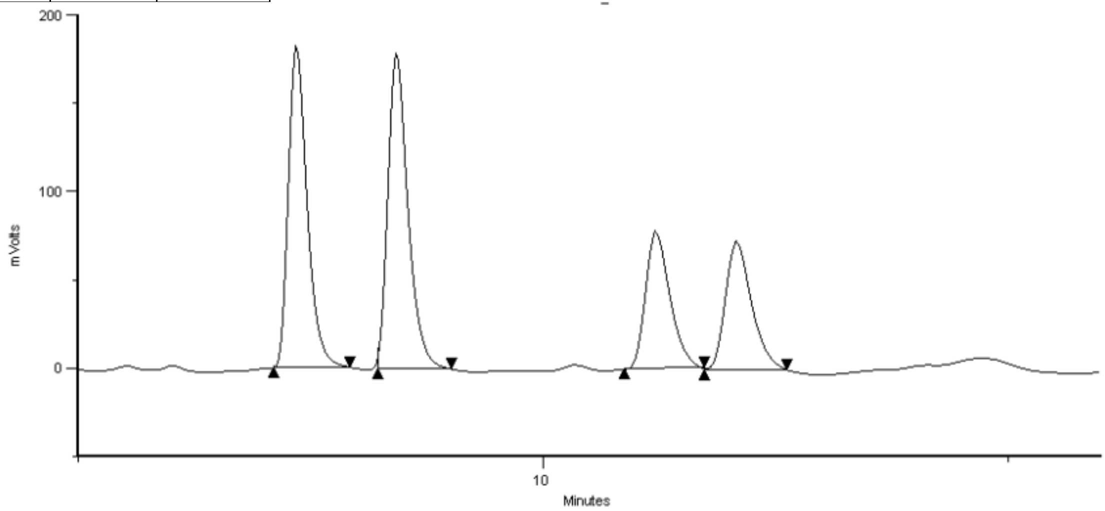

Peak	t_R (min)	Area (%)
1	7.35	21.14
2	8.44	8.48
3	11.29	70.04
4	12.03	0.35

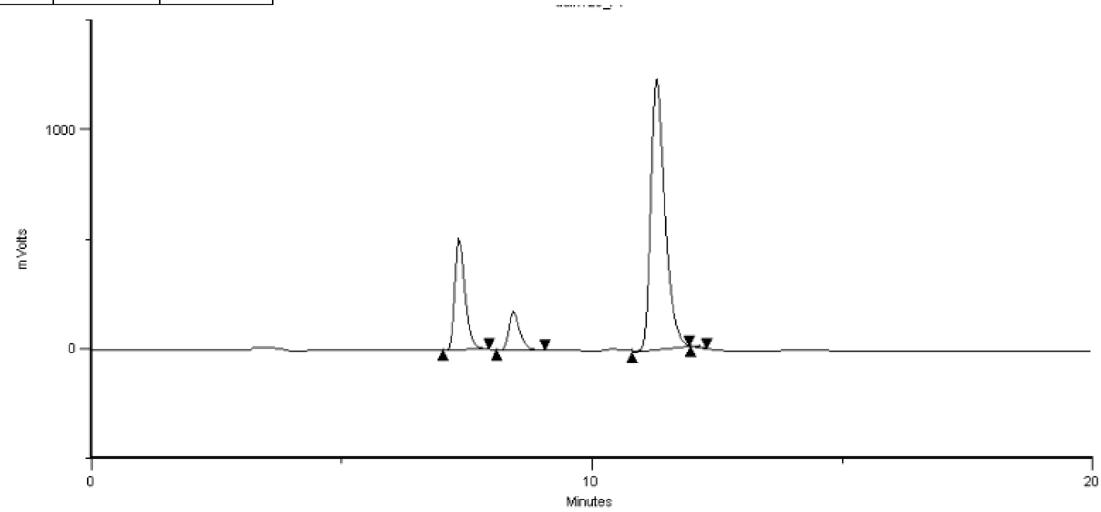

HPLC Data for **32**: Chiralpak IA (90:10 hexane : IPA, flow rate 1.0 mLmin^{-1}, 211 nm, 40 °C) t_R (2*R*,3*R*): 17.0 min, t_R (2*S*,3*S*): 21.1 min, >99% ee.

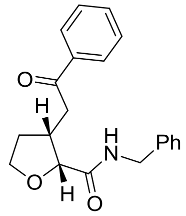

PDA Ch1 211nm

Peak#	Ret. Time	Area	Area%
1	17.299	6804841	49.497
2	20.879	6943062	50.503
Total		13747903	100.000

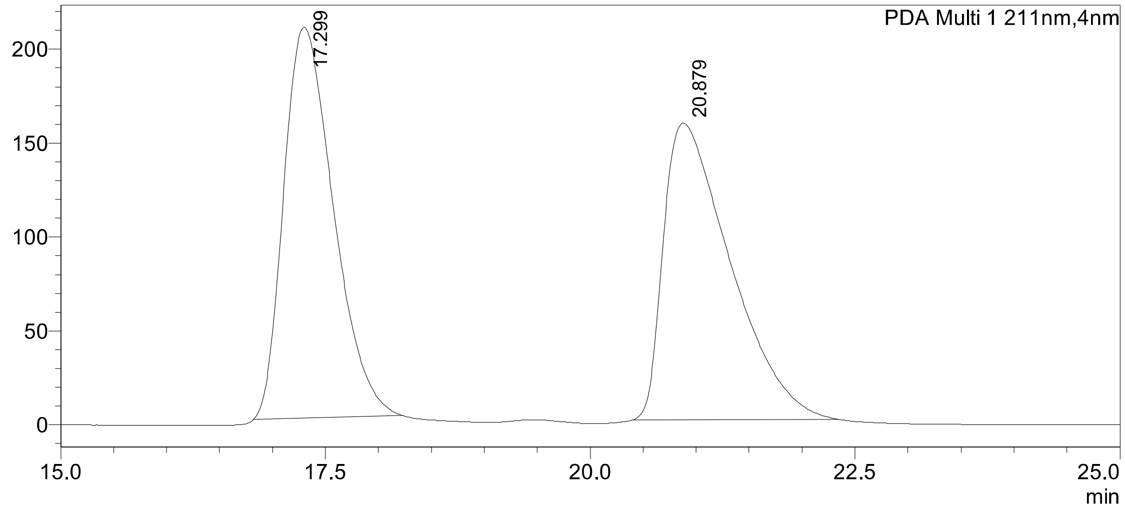

PDA Ch1 211nm

Peak#	Ret. Time	Area	Area%
1	17.067	24056116	100.255
2	20.763	-61241	-0.255
Total		23994875	100.000

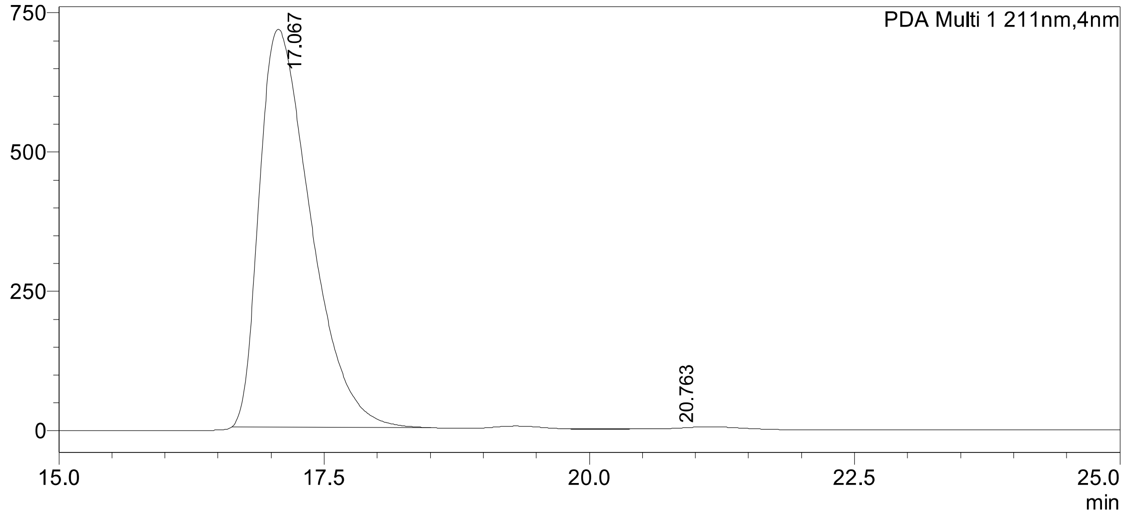

HPLC Data for **33**: Chiralpak IA (90:10 hexane : IPA, flow rate 1.0 mLmin^{-1}, 211 nm, 40 °C) t_R (2*R*,3*R*): 26.0 min, t_R (2*S*,3*S*): 31.3 min, 99% ee.

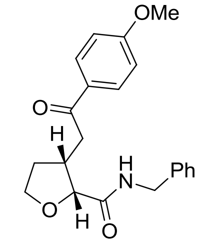

PDA Ch1 211nm

Peak#	Ret. Time	Area	Area%
1	26.193	12722006	50.308
2	31.111	12566333	49.692
Total		25288339	100.000

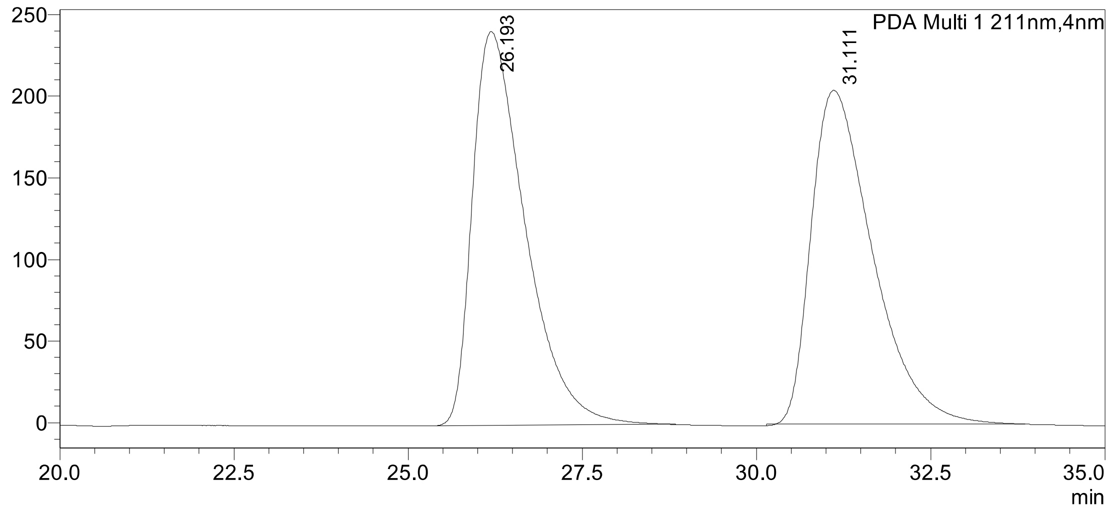

PDA Ch1 211nm

Peak#	Ret. Time	Area	Area%
1	25.957	23764586	99.450
2	31.340	131497	0.550
Total		23896084	100.000

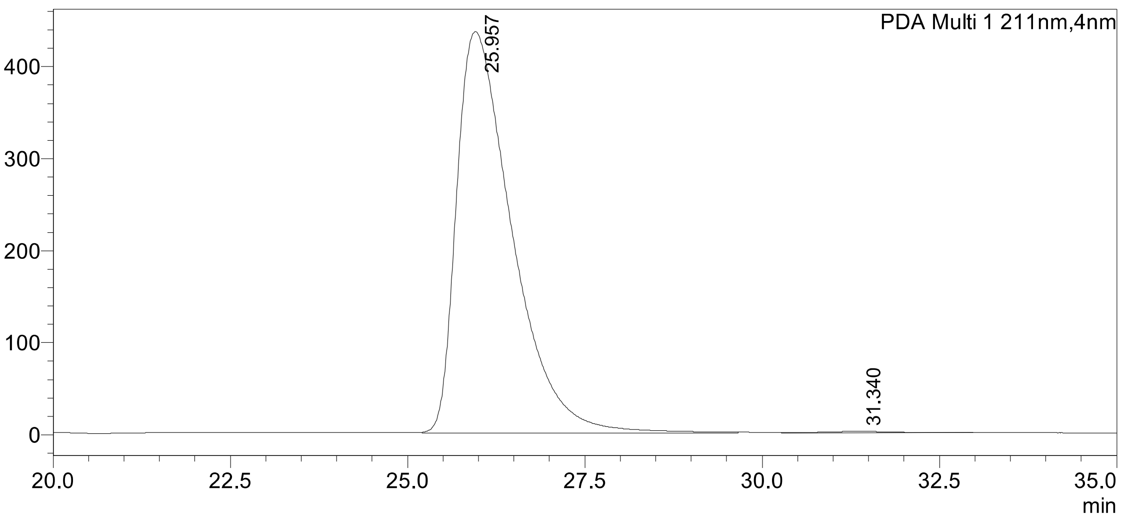

HPLC Data for **34**: Chiralpak IA (95:5 hexane : IPA, flow rate 1.0 mLmin^{-1}, 254 nm, 40 °C) t_R (2*R*,3*R*): 36.4 min, t_R (2*S*,3*S*): 41.3 min, 99% ee.

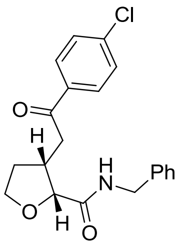

PDA Ch1 254nm

Peak#	Ret. Time	Area	Area%
1	36.008	2084418	50.438
2	40.556	2048195	49.562
Total		4132613	100.000

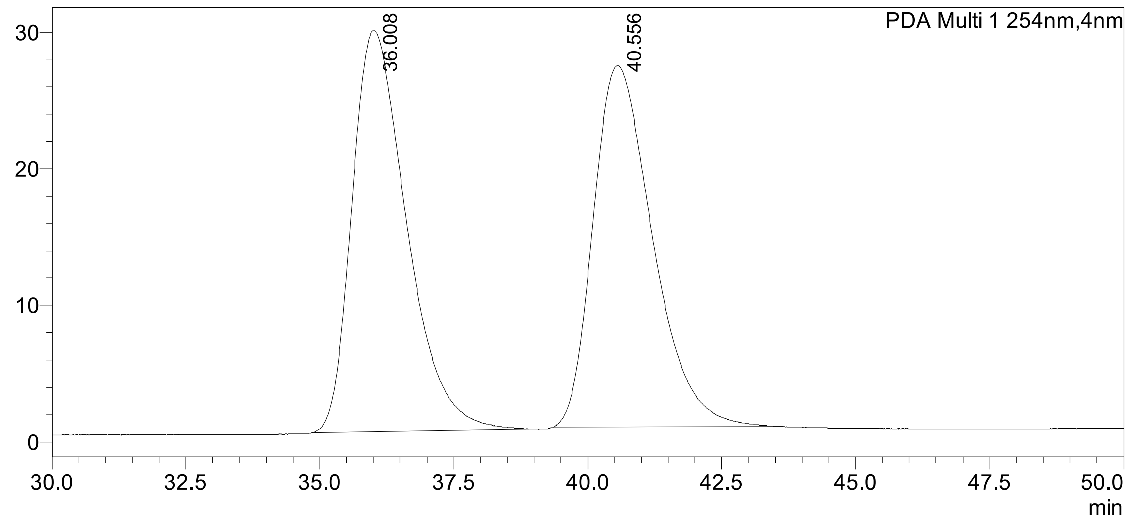

PDA Ch1 254nm

Peak#	Ret. Time	Area	Area%
1	36.449	8594122	99.255
2	41.282	64474	0.745
Total		8658595	100.000

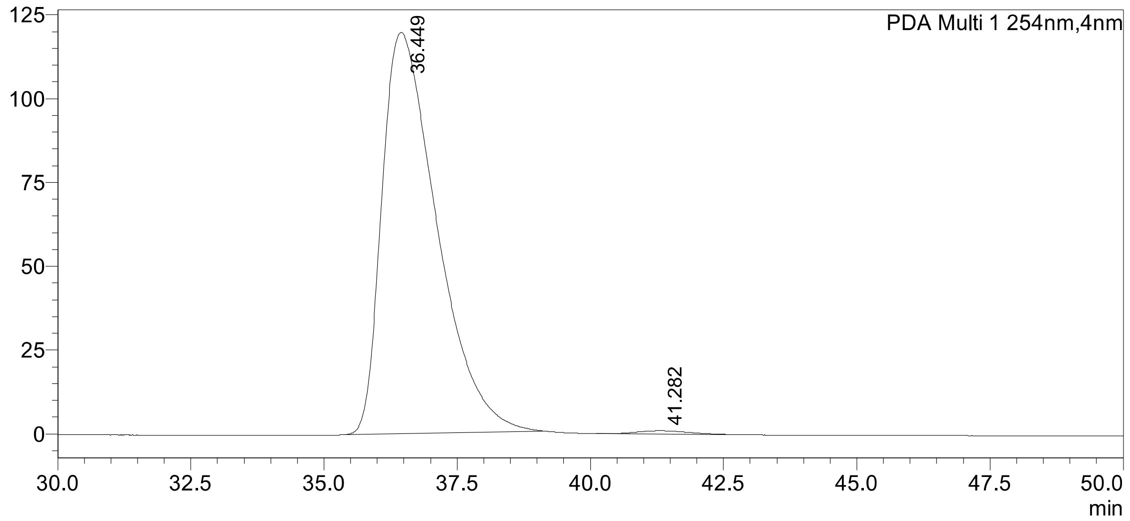

HPLC Data for **35**: Chiralpak IA (90:10 hexane : IPA, flow rate 1.0 mLmin^{-1}, 220 nm, 40 °C) t_R (2*R*,3*R*): 10.5 min, t_R (2*S*,3*S*): 12.4 min, 98% ee.

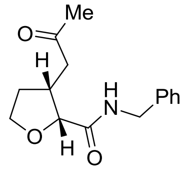

PDA Ch1 220nm

Peak#	Ret. Time	Area	Area%
1	10.710	1750133	50.372
2	12.328	1724271	49.628
Total		3474404	100.000

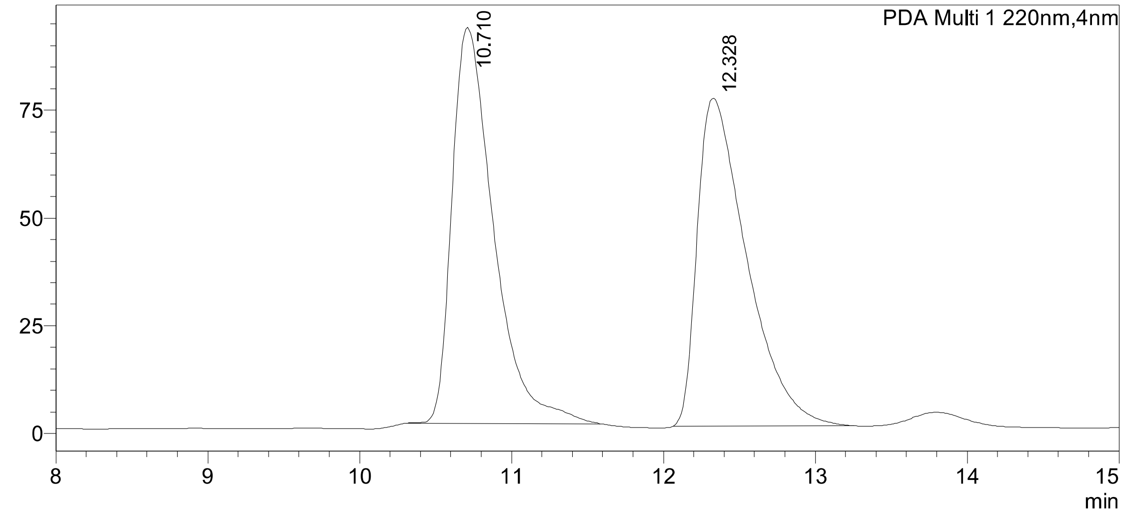

PDA Ch1 220nm

Peak#	Ret. Time	Area	Area%
1	10.475	13982492	98.755
2	12.444	176263	1.245
Total		14158755	100.000

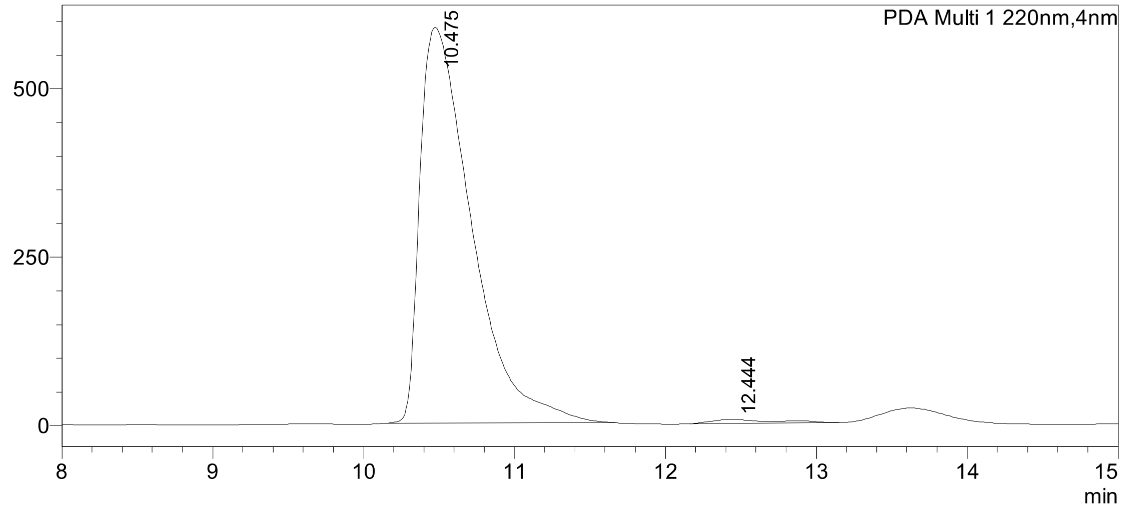

HPLC Data for **36**: Chiralpak AS-H (90:10 hexane : IPA, flow rate 1.0 mLmin^{-1}, 211 nm, 40 °C) t_R (2*S*,3*S*): 9.0 min, t_R (2*R*,3*R*): 13.2 min, 98% ee.

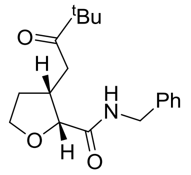

PDA Ch1 211nm

Peak#	Ret. Time	Area	Area%
1	8.935	8159815	49.492
2	13.366	8327413	50.508
Total		16487228	100.000

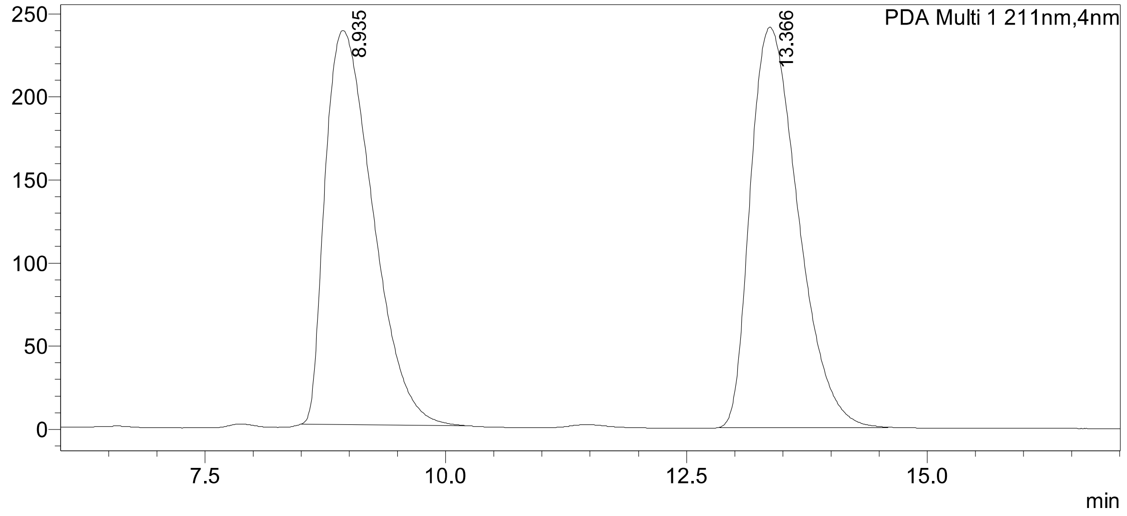

PDA Ch1 211nm

Peak#	Ret. Time	Area	Area%
1	9.000	91549	0.735
2	13.216	12355799	99.265
Total		12447348	100.000

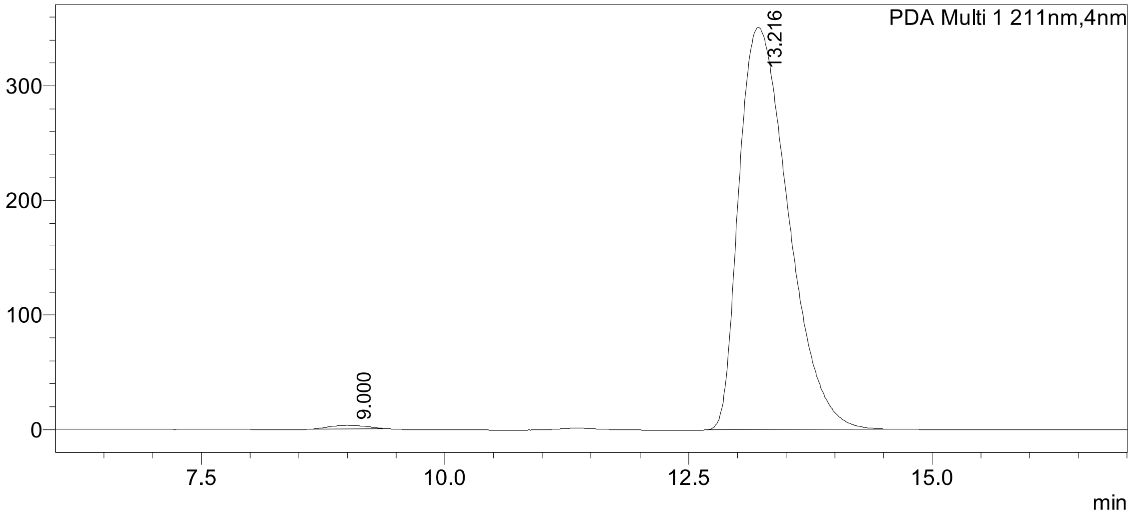

HPLC Data for **37**: Chiralpak IB (95:5 hexane : IPA, flow rate 1.0 mLmin^{-1}, 211 nm, 40 °C) t_R (2*S*,3*S*): 13.8 min, t_R (2*R*,3*R*): 15.9 min, >99% ee.

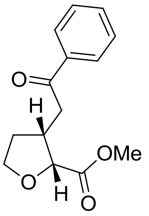

PDA Ch2 211nm

Peak#	Ret. Time	Area	Area%
1	13.796	28536221	50.894
2	15.898	27533742	49.106
Total		56069963	100.000

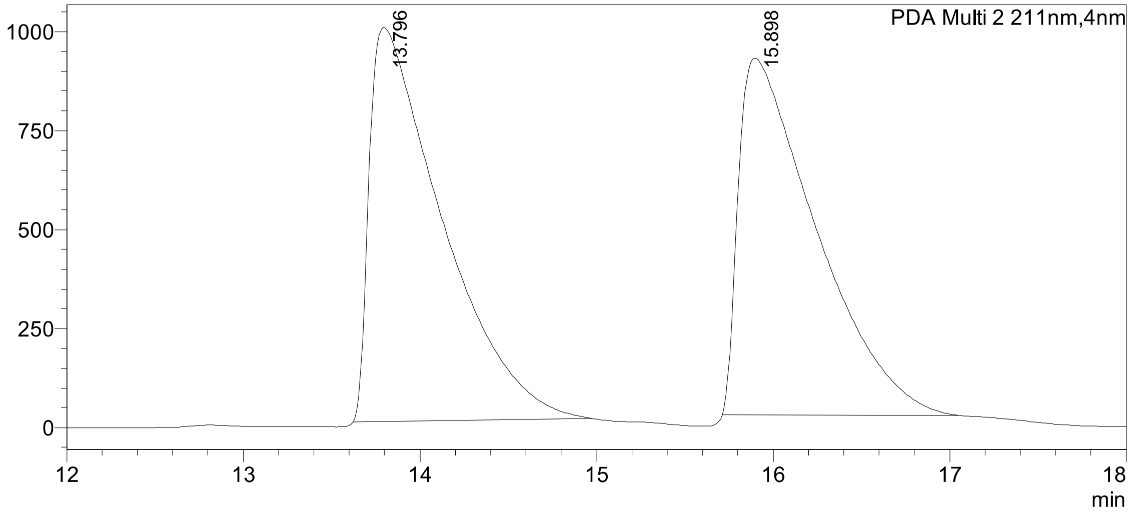

PDA Ch1 211nm

Peak#	Ret. Time	Area	Area%
1	13.777	-17	-0.000
2	15.853	26512758	100.000
Total		26512741	100.000

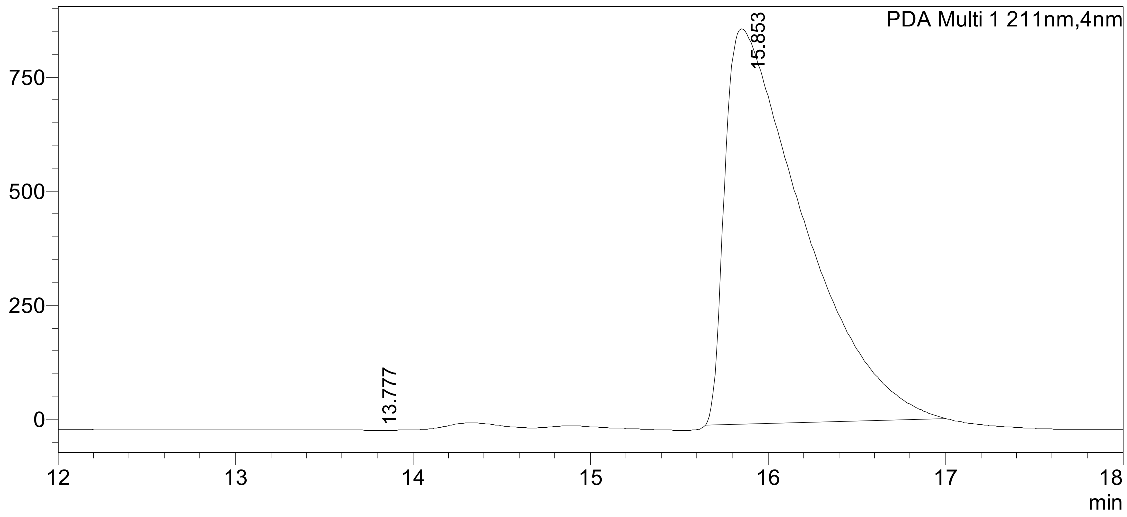

HPLC Data for **38**: Chiralpak IA (90:10 hexane : IPA, flow rate 1.0 mLmin^{-1}, 211 nm, 40 °C) t_R (2*R*,3*R*): 17.3 min, t_R (2*S*,3*S*): 24.3 min, 99% ee.

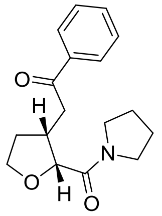

PDA Ch1 211nm

Peak#	Ret. Time	Area	Area%
1	17.398	18636985	50.047
2	23.813	18602175	49.953
Total		37239160	100.000

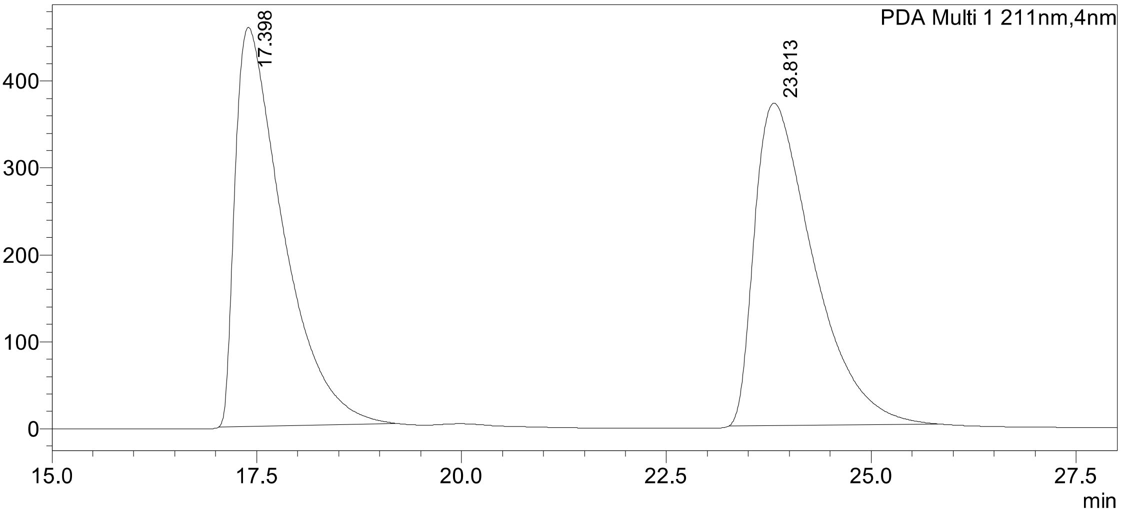

PDA Ch1 211nm

Peak#	Ret. Time	Area	Area%	Height%
1	17.344	26786253	98.996	99.321
2	24.340	271655	1.004	0.679
Total		27057908	100.000	100.000

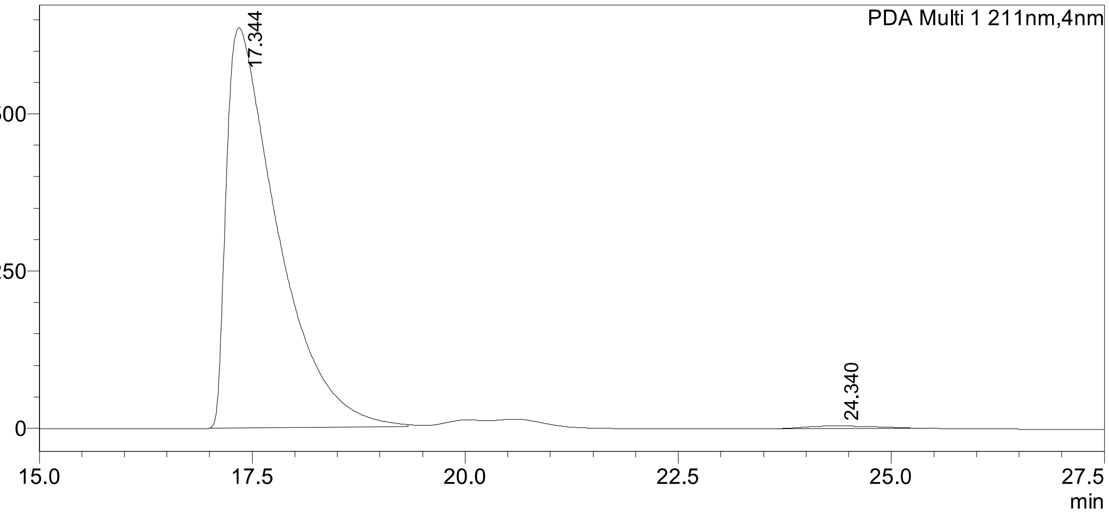

HPLC Data for **39**: Chiralpak AS-H (90:10 hexane : IPA, flow rate 1.0 mLmin^{-1}, 211 nm, 40 °C) t_R (2*R*,3*R*): 6.3 min, t_R (2*S*,3*S*): 9.9 min, >99% ee.

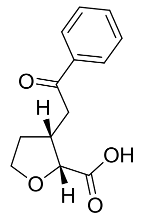

PDA Ch1 211nm

Peak#	Ret. Time	Area	Area%
1	6.246	11451029	49.592
2	9.799	11639328	50.408
Total		23090357	100.000

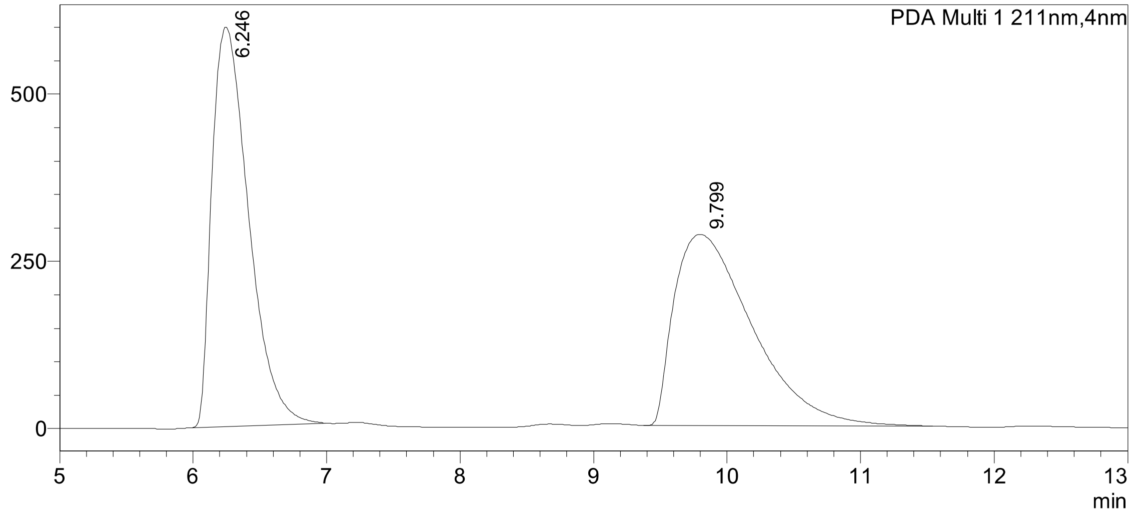

PDA Ch1 211nm

Peak#	Ret. Time	Area	Area%
1	6.268	5759282	100.006
2	9.872	-343	-0.006
Total		5758938	100.000

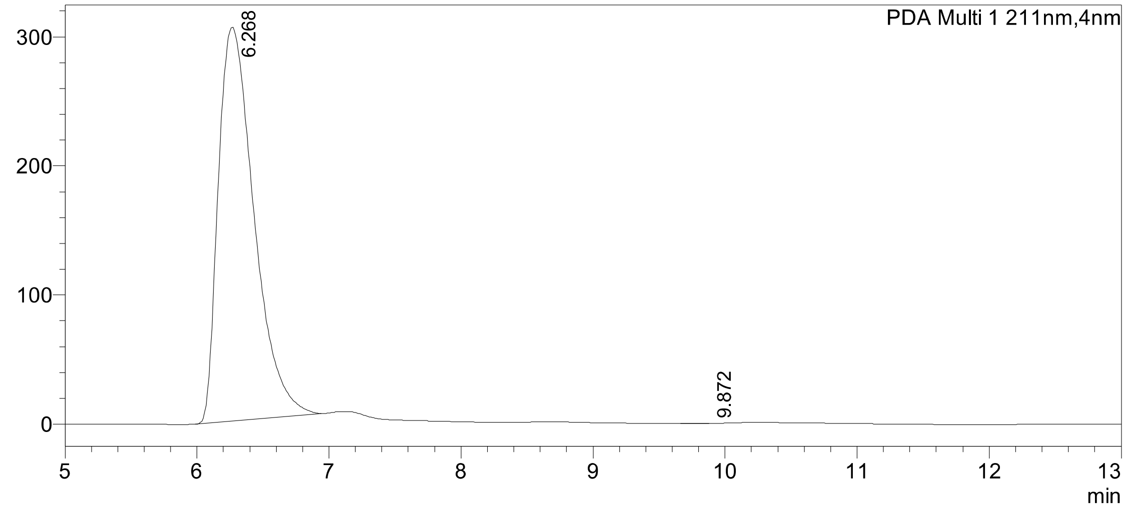

HPLC Data for **43**: Chiralpak IA (95:5 hexane : IPA, flow rate 1.00 mLmin^{-1}, 211 nm, 40 °C) t_R (3a*R*,7a*S*): 18.9 min, t_R (3a*S*,7a*R*): 25.3 min, 98% ee.

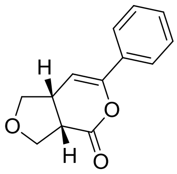

PDA Ch1 211nm

Peak#	Ret. Time	Area	Area%
1	18.860	4357250	50.299
2	25.261	4305420	49.701
Total		8662670	100.000

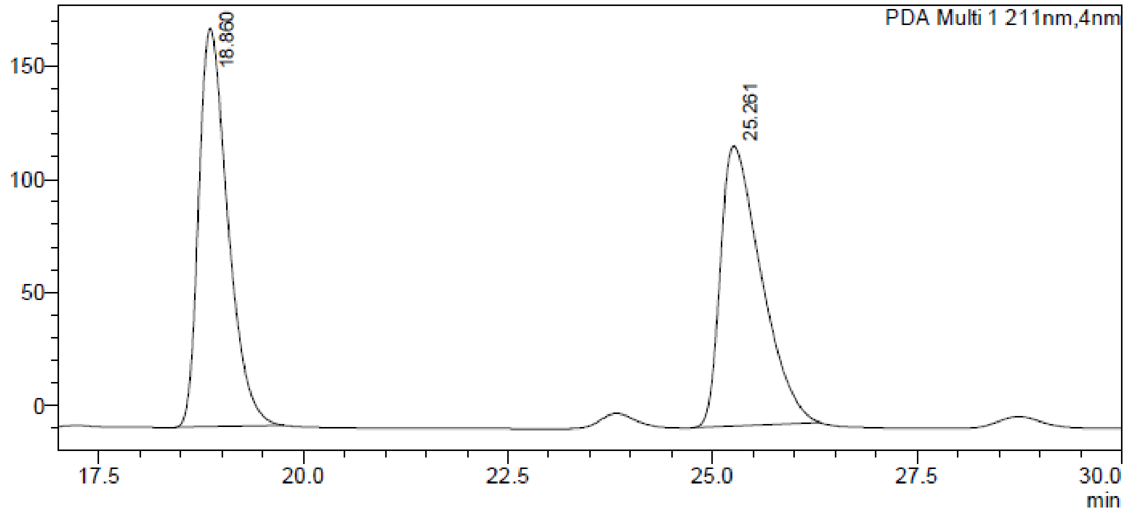

PDA Ch1 211nm

Peak#	Ret. Time	Area	Area%
1	18.919	149379	0.882
2	24.909	16791957	99.118
Total		16941335	100.000

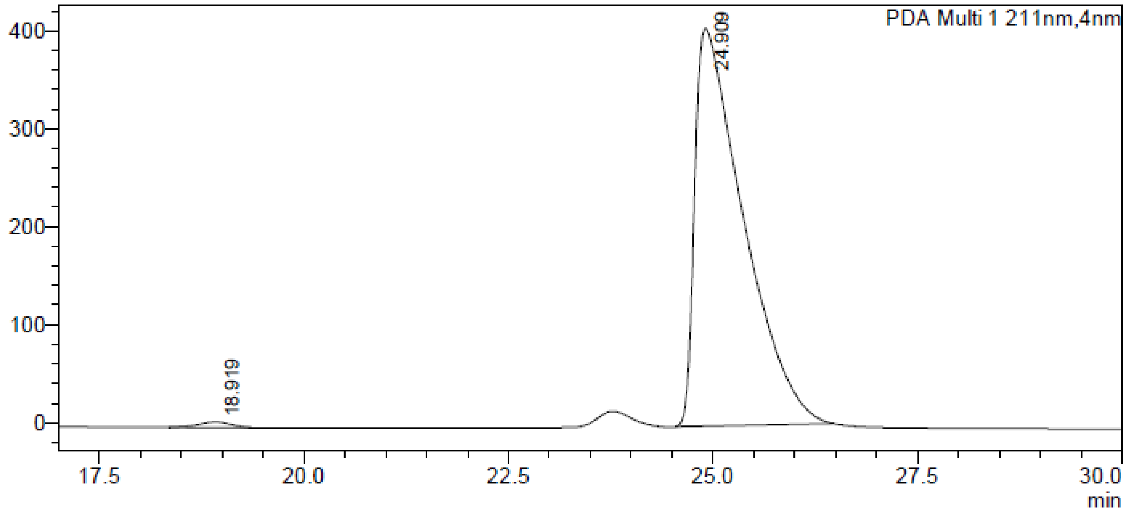

HPLC Data for **44**: Chiralcel OD-H (90:10 hexane : IPA, flow rate 1.50 mLmin^{-1}, 211 nm, 40 °C). t_R (3a*S*,7a*R*): 11.3 min, t_R (3a*R*,7a*S*): 14.0 min, 99% ee.

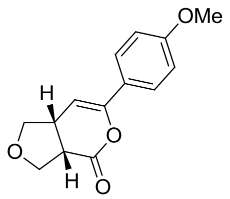

PDA Ch1 211nm

Peak#	Ret. Time	Area%
1	11.29	40.477
2	13.18	9.292
3	13.96	40.549
4	30.68	9.683
Total		100.000

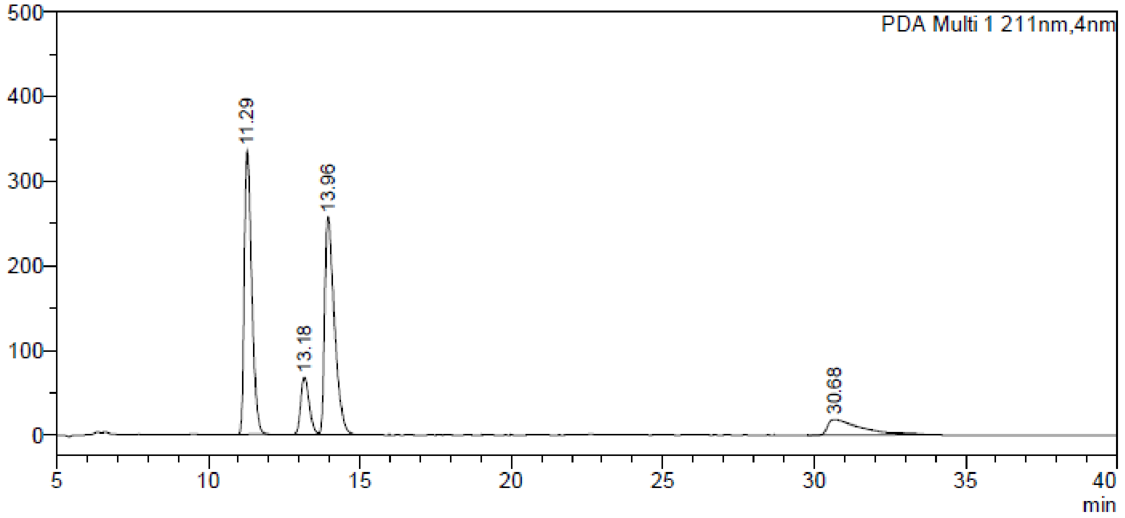

PDA Ch1 211nm

Peak#	Ret. Time	Area%
1	11.36	99.257
2	14.30	0.743
Total		100.000

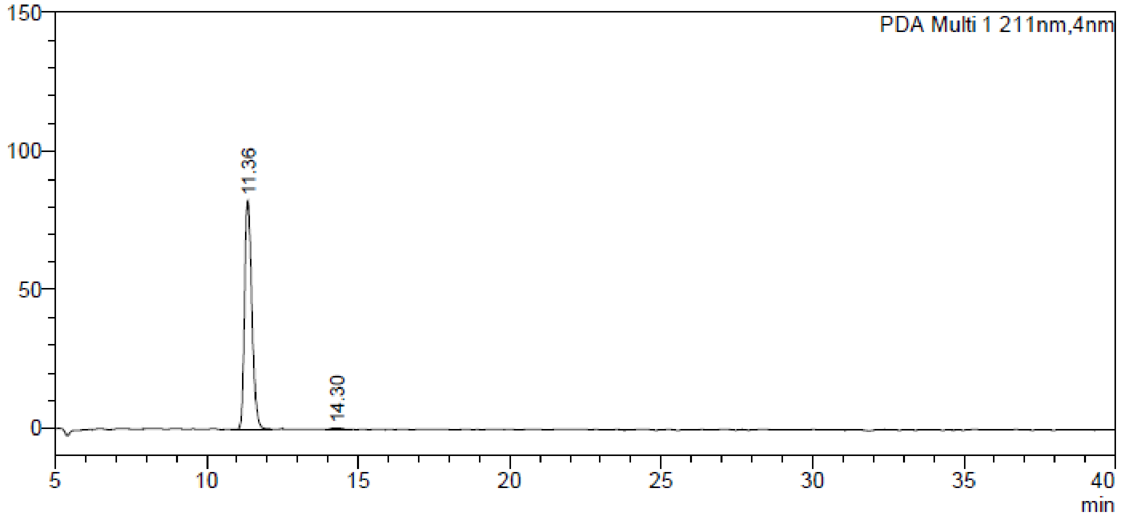

HPLC Data for **45**: Chiralpak IA (95:5 hexane : IPA, flow rate 1.50 mLmin^{-1}, 211 nm, 40 °C) t_R (3a*R*,7a*S*): 13.7min, t_R (3a*S*,7a*R*): 16.5min, 94% ee.

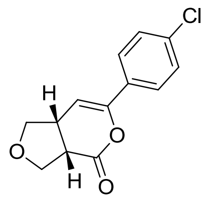

PDA Ch1 211nm

Peak#	Ret. Time	Area	Area%
1	13.417	8709400	49.921
2	16.361	8737015	50.079
Total		17446415	100.000

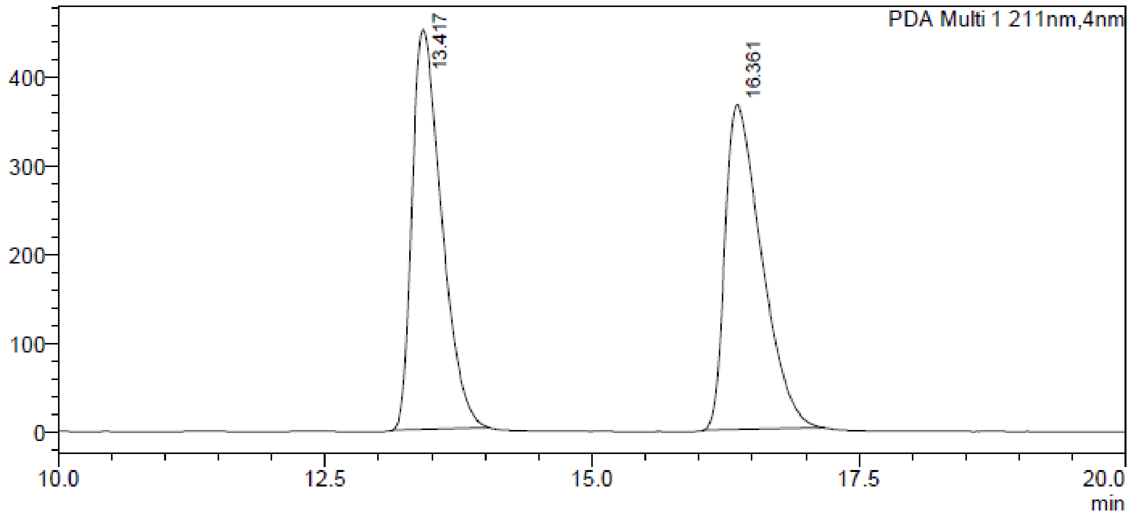

PDA Ch1 211nm

Peak#	Ret. Time	Area	Area%
1	13.736	448310	2.906
2	16.546	14980523	97.094
Total		15428833	100.000

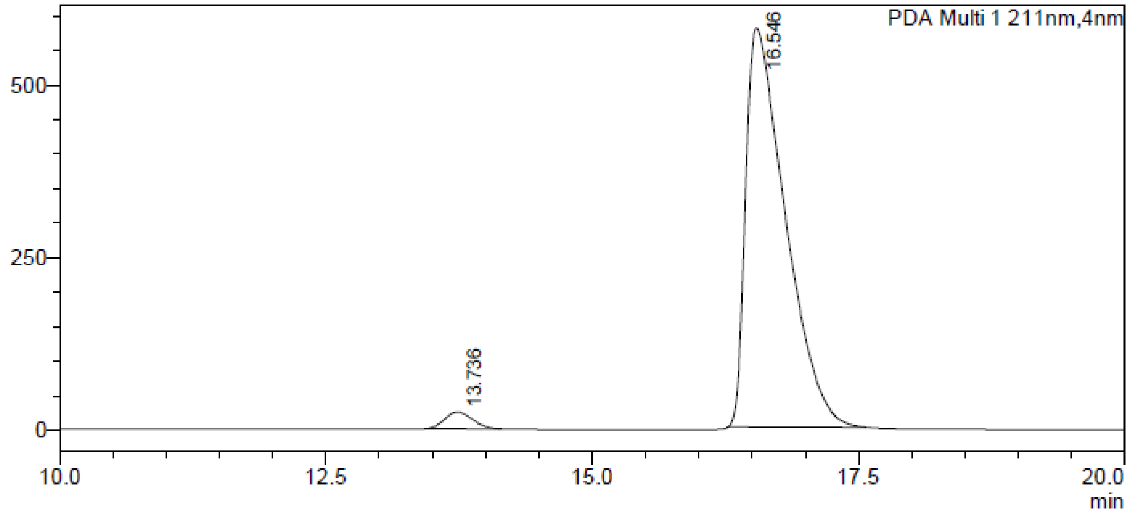

HPLC Data for **46**: Chiralpak IA (90:10 hexane : IPA, flow rate 1.5mLmin^{-1}, 254 nm, 40 °C) t_R (3*S*,4*R*): 14.0min, t_R (3*R*,4*S*): 20.6min, 96% ee.

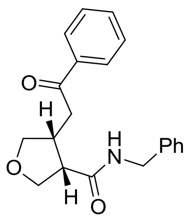

PDA Ch1 254nm

Peak#	Ret. Time	Area	Area%
1	14.079	9454487	50.162
2	20.152	9393423	49.838
Total		18847910	100.000

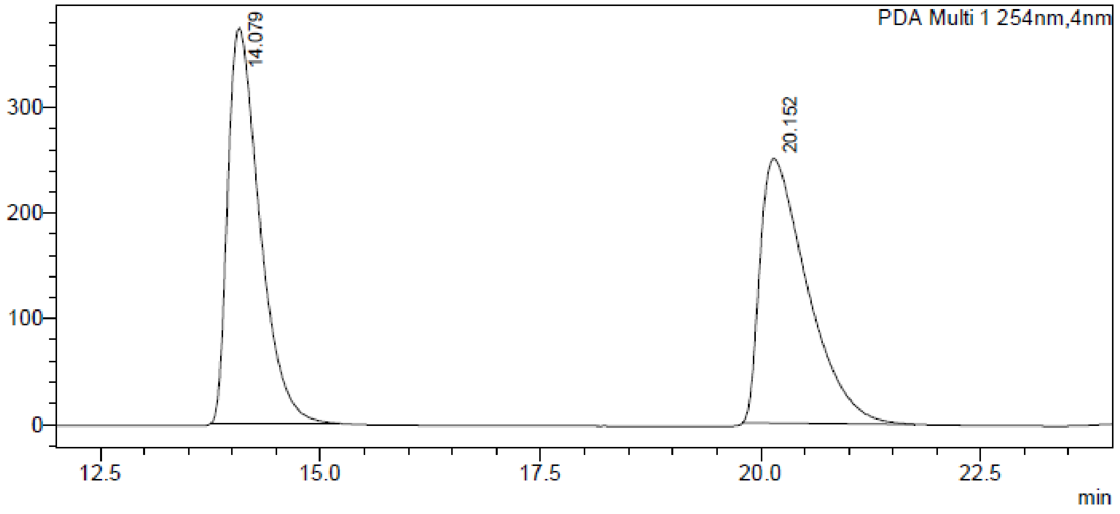

PDA Ch1 254nm

Peak#	Ret. Time	Area	Area%
1	14.041	14954289	98.110
2	20.591	288123	1.890
Total		15242412	100.000

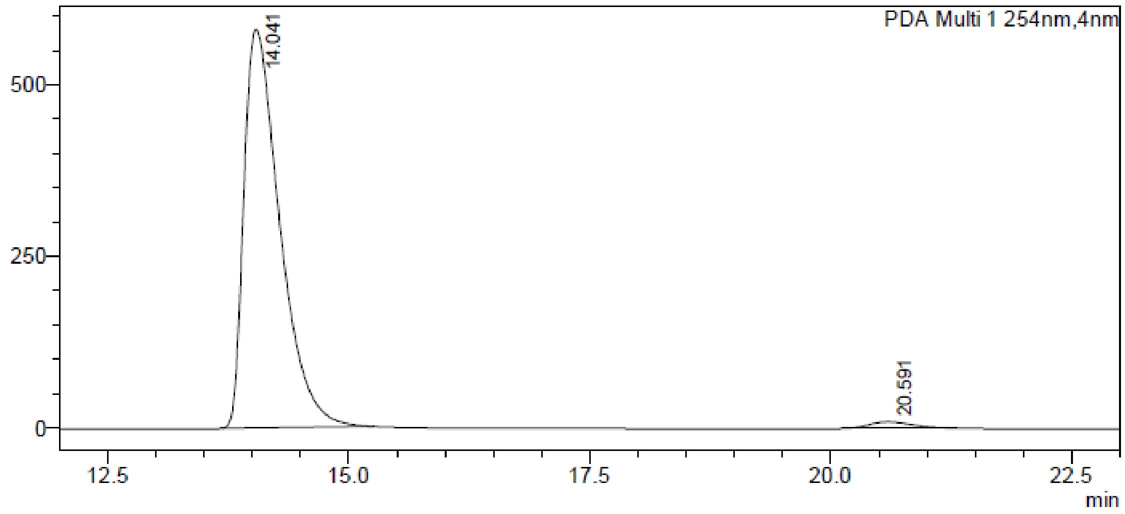

HPLC Data for **47**: Chiralcel OJ-H (90:10 hexane : IPA, flow rate 1.50 mLmin^{-1}, 211 nm, 40 °C). t_R (3*S*,4*R*): 22.5 min, t_R (3*R*,4*S*): 28.2 min, 97% ee.

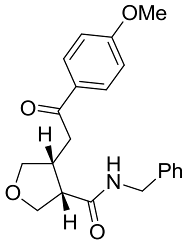

PDA Ch1 211nm

Peak#	Ret. Time	Area%
1	22.643	33.196
2	27.999	32.650
3	53.634	16.893
4	57.108	17.260
Total		100.000

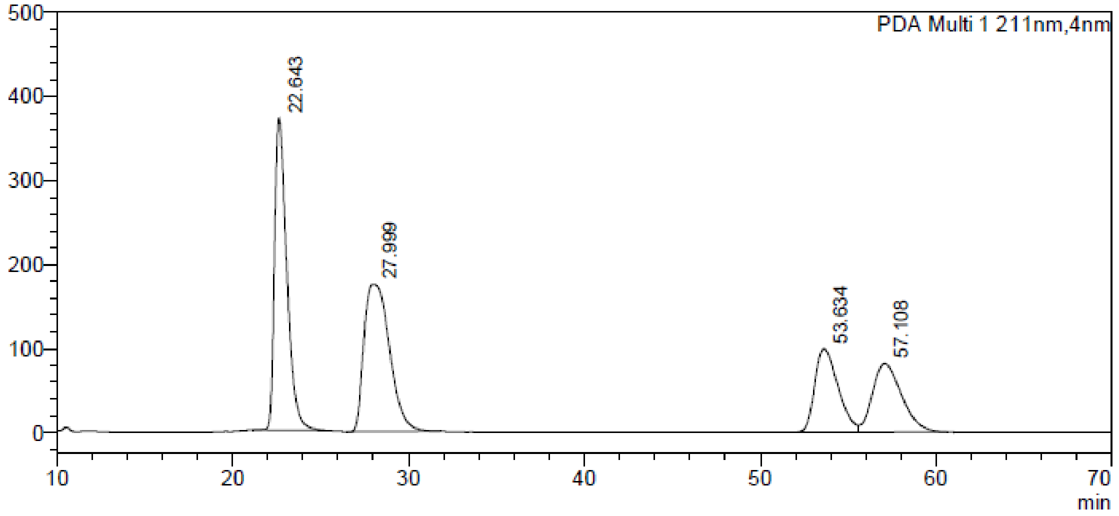

PDA Ch1 211nm

Peak#	Ret. Time	Area%
1	22.474	97.441
2	28.242	1.249
3	54.163	1.311
Total		100.000

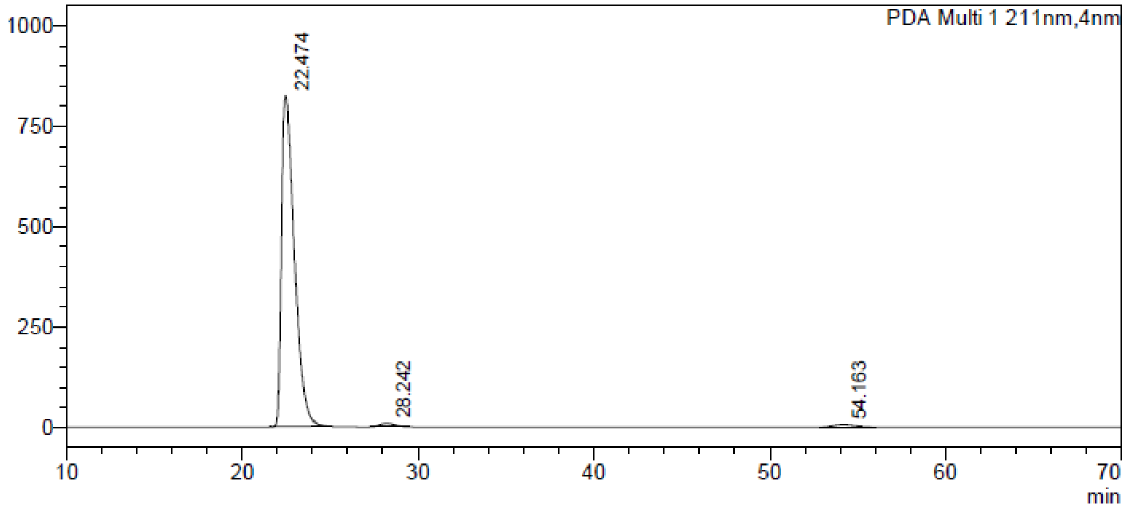

HPLC Data for **48**: Chiralpak IA (90:10 hexane:IPA, flow rate 1 mLmin^{-1}, 254 nm, 40 °C) t_R (3*S*,4*R*): 18.1min, t_R (3*R*,4*S*): 25.0min, 98% ee.

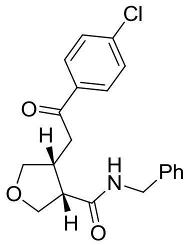

PDA Ch2 254nm

Peak#	Ret. Time	Area	Area%
1	18.343	9644327	49.533
2	24.608	9826073	50.467
Total		19470399	100.000

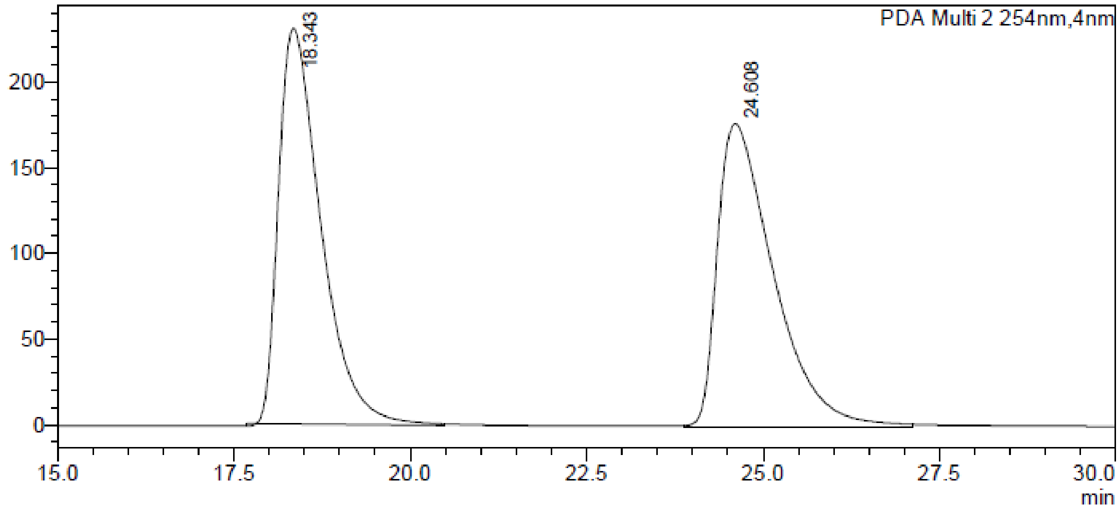

PDA Ch1 254nm

Peak#	Ret. Time	Area	Area%
1	18.142	35820848	99.000
2	25.013	361859	1.000
Total		36182707	100.000

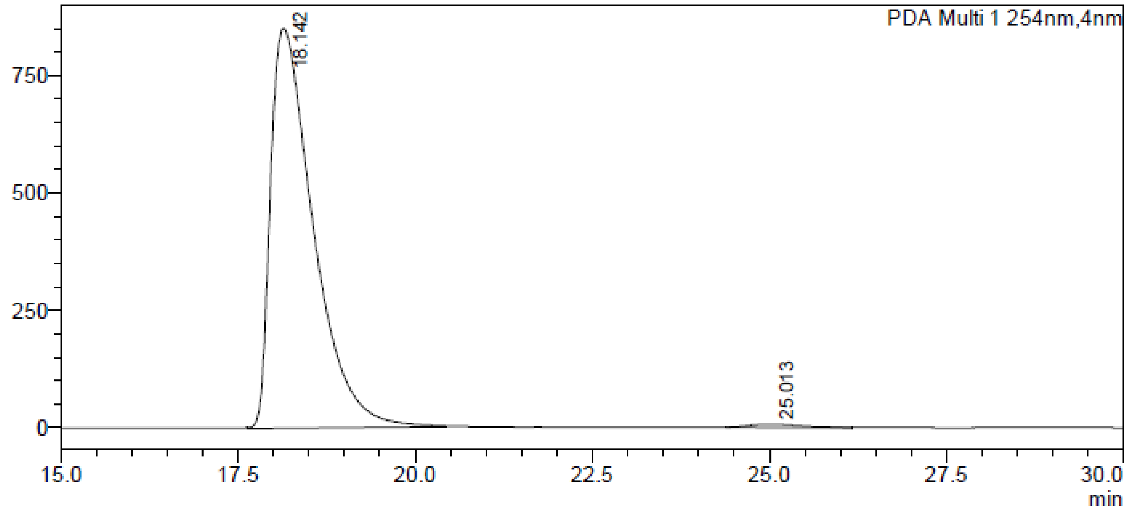

HPLC Data for **49** and *ent*-**43**: Chiralpak IA (95:5 hexane : IPA, flow rate 1.70 mLmin^{-1}, 211 nm, 40 °C) **49**: t_R (3a*S*,7a*S*): 23.4 min, t_R (3a*R*,7a*R*): 27.0 min, >99% ee; *ent*-**43**: t_R (3a*R*,7a*S*): 18.5 min, t_R (3a*S*,7a*R*): 25.0 min, 78% ee.[7]

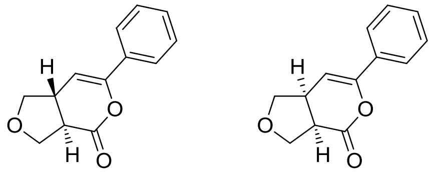

PDA Ch1 211nm

Peak#	Ret. Time	Area	Area%
1	18.513	14571514	30.420
2	23.152	8610203	17.975
3	24.799	8122662	16.957
4	27.678	16596395	34.647
Total		47900774	100.000

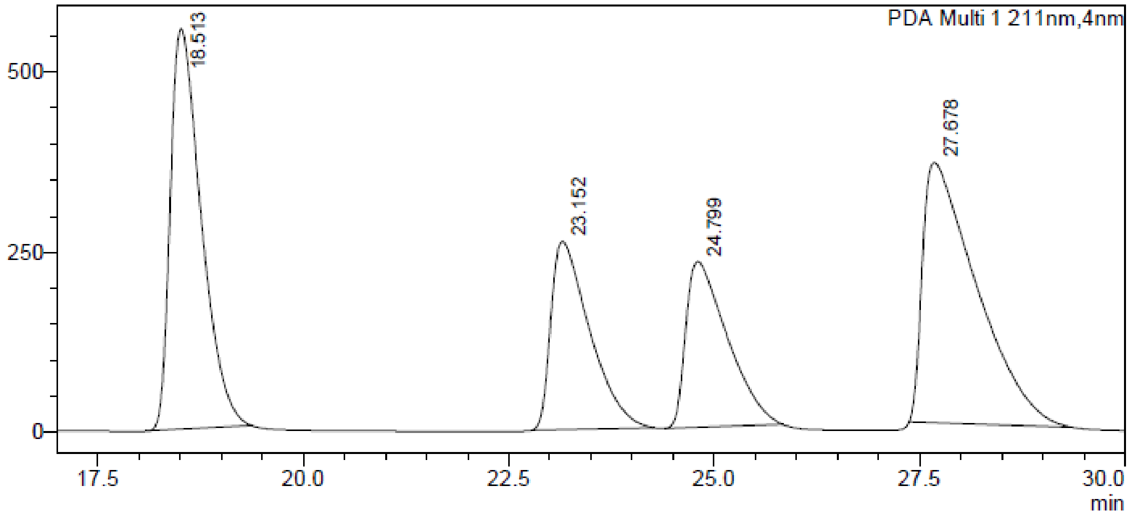

PDA Ch1 211nm

Peak#	Ret. Time	Area	Area%
1	18.500	4674401	8.537
2	23.387	89704	0.164
3	24.982	565439	1.033
4	26.958	49424373	90.266
Total		54753918	100.000

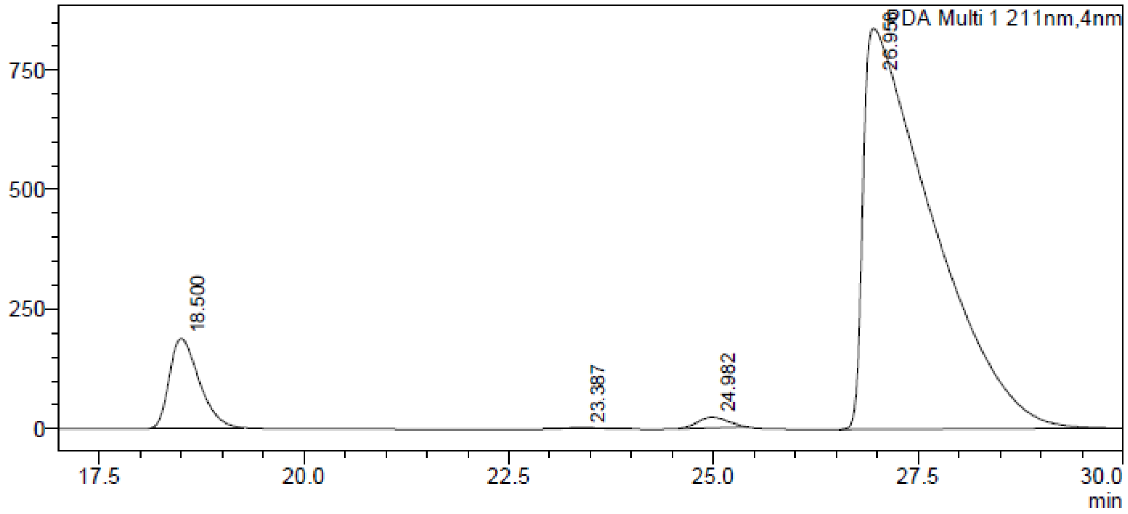

HPLC Data for **50** and *ent*-**44**: Chiralcel OD-H (90:10 hexane : IPA, flow rate 1.50 mLmin^{-1}, 211 nm, 40 °C) **50**: t_R (3a*R*,7a*R*): 13.1 min, t_R (3a*S*,7a*S*): 31.5 min, >99% ee; *ent*-**44**: t_R (3a*S*,7a*R*): 11.4 min, t_R (3a*R*,7a*S*): 14.2 min, 77% ee.

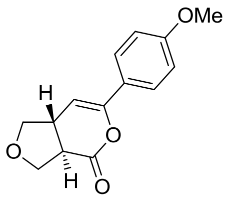

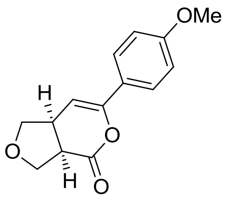

PDA Ch1 211nm

Peak#	Ret. Time	Area%
1	11.29	40.477
2	13.18	9.292
3	13.96	40.549
4	30.68	9.683
Total		100.000

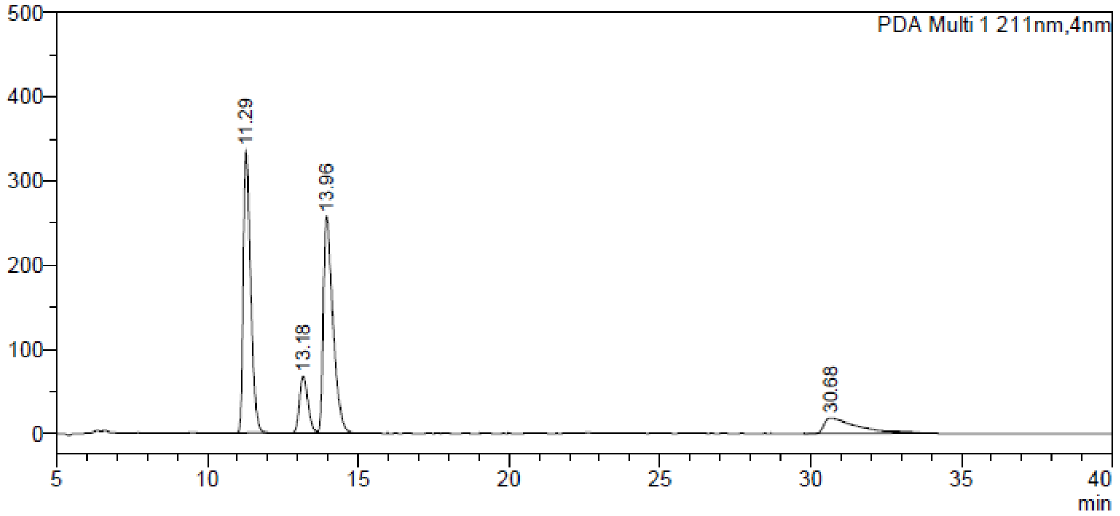

PDA Ch1 211nm

Peak#	Ret. Time	Area%
1	11.42	1.838
2	13.12	83.838
3	14.15	14.282
4	31.54	0.043
Total		100.000

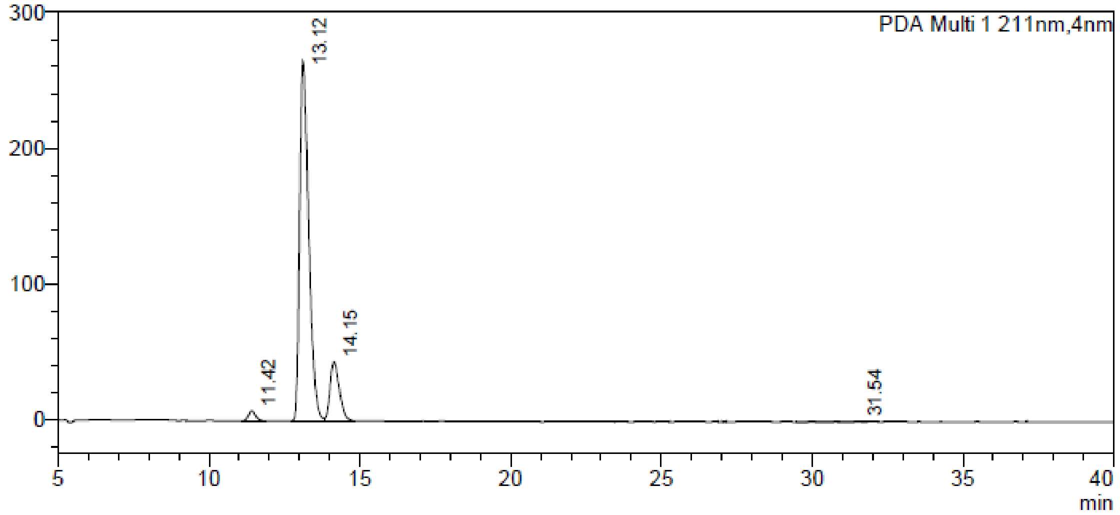

HPLC Data for **51** and *ent*-**48**: Chiralcel OD-H (90:10 hexane : IPA, flow rate 1.50 mLmin^{-1}, 211 nm, 40 °C) **51**: t_R (3a*R*,7a*R*): 11.1 min, t_R (3a*S*,7a*S*): 26.0 min, >99% ee; *ent*-**48**: t_R (3a*S*,7a*R*): 8.9 min, t_R (3a*R*,7a*S*): 9.6 min, 75% ee.

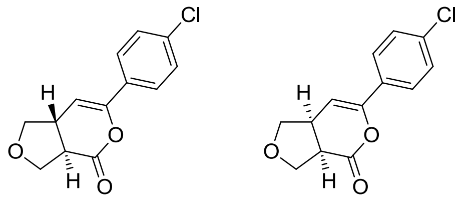

PDA Ch1 211nm

Peak#	Ret. Time	Area%
1	8.919	45.515
2	9.511	45.723
3	11.333	4.374
4	25.038	4.389
Total		100.000

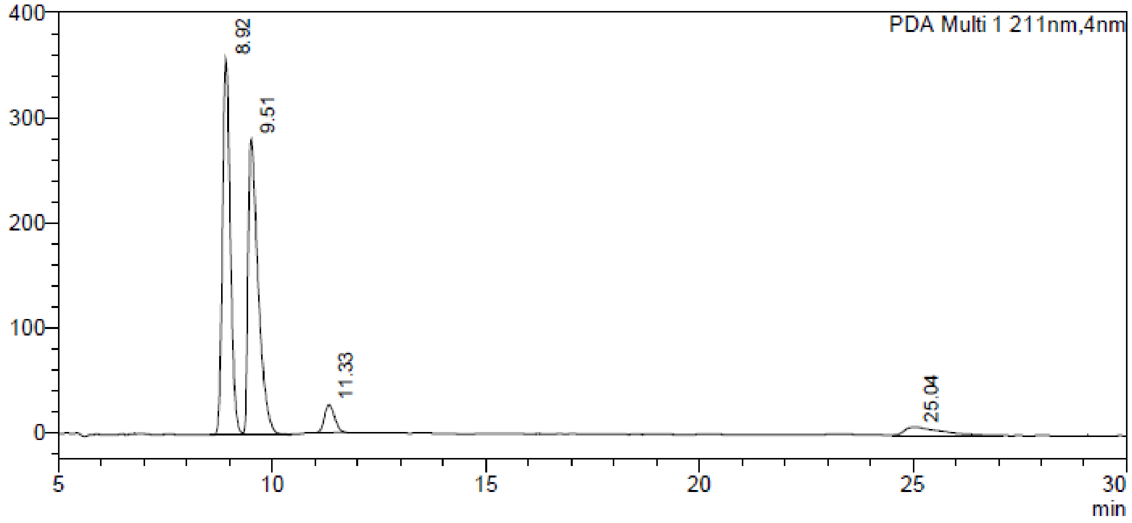

PDA Ch1 211nm

Peak#	Ret. Time	Area%
1	8.937	0.923
2	9.590	6.421
3	11.100	92.562
4	25.950	0.094
Total		100.000

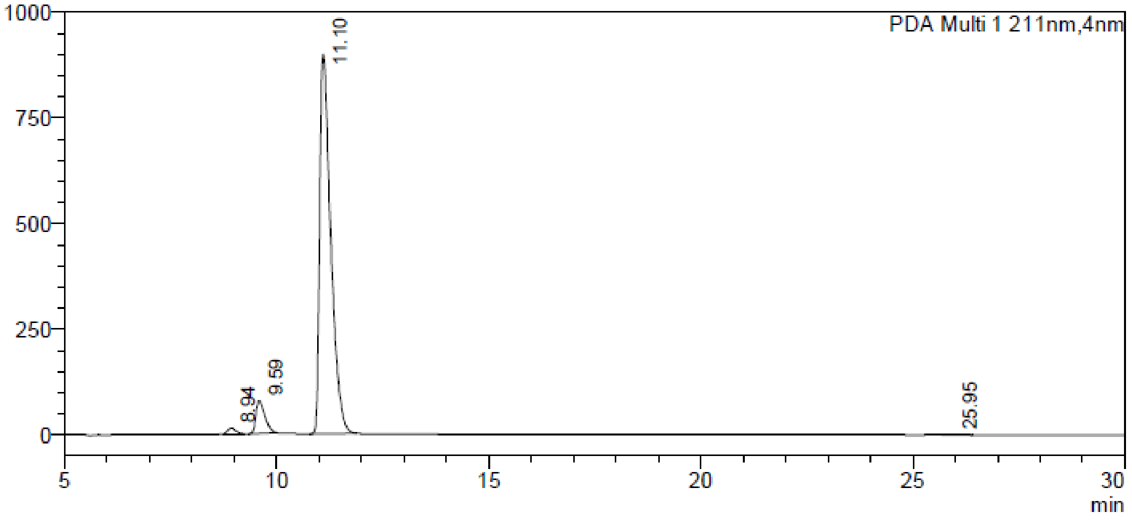

HPLC Data for **52** and *ent*-**46**: Chiralpak IB (92.5:7.5 hexane : IPA, flow rate 1.50 mLmin^{-1}, 211 nm, 40 °C) **52**: t_R (3*R*,4*R*): 29.7 min, t_R (3*S*,4*S*): 32.5 min, >99% ee; *ent*-**46**: t_R (3*R*,4*S*): 17.3 min, t_R (3*S*,4*R*): 19.5 min, 81% ee.

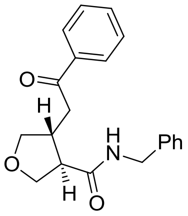

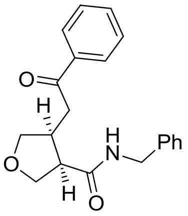

PDA Ch1 211nm

Peak#	Ret. Time	Area%
1	16.971	36.799
2	18.810	37.156
3	30.267	12.899
4	32.540	13.147
Total		100.000

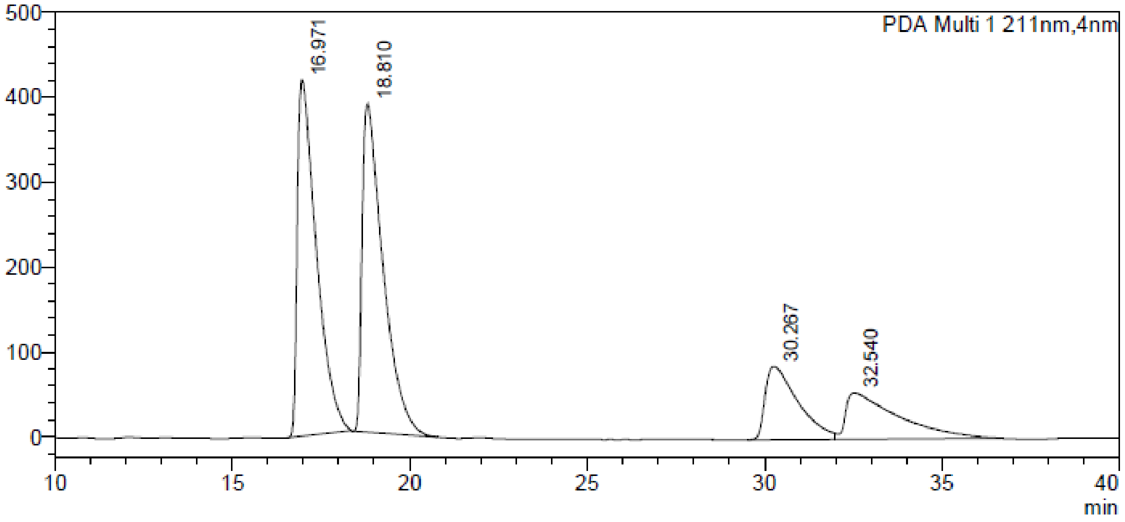

PDA Ch1 211nm

Peak#	Ret. Time	Area%
1	17.331	15.791
2	19.452	1.698
3	29.711	82.512
Total		100.000

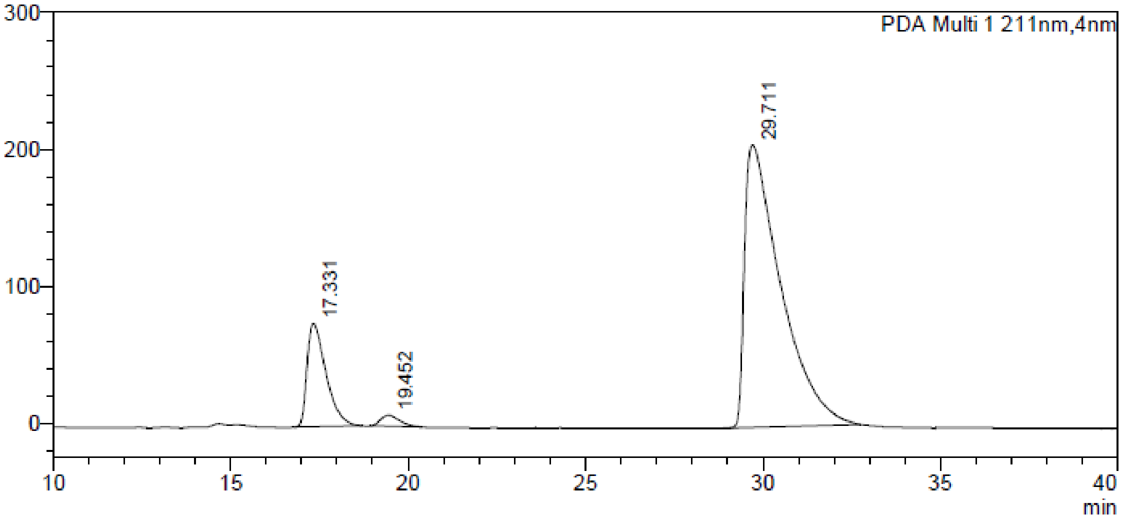

HPLC Data for **53** and *ent*-**47**: Chiralcel OJ-H (90:10 hexane : IPA, flow rate 1.50 mLmin^{-1}, 211 nm, 40 °C) **53**: t_R (3*S*,4*S*): 53.6 min, t_R (3*R*,4*R*): 57.1 min, >99% ee; *ent*-**47**: t_R (3*S*,4*R*): 23.0 min, t_R (3*R*,4*S*): 28.0 min, 80% ee.

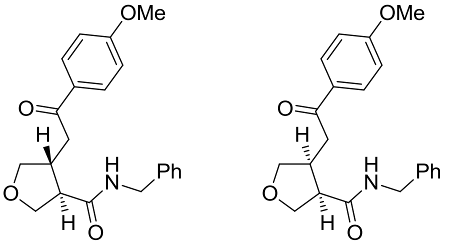

PDA Ch1 211nm

Peak#	Ret. Time	Area%
1	22.643	33.196
2	27.999	32.650
3	53.634	16.893
4	57.108	17.260
Total		100.000

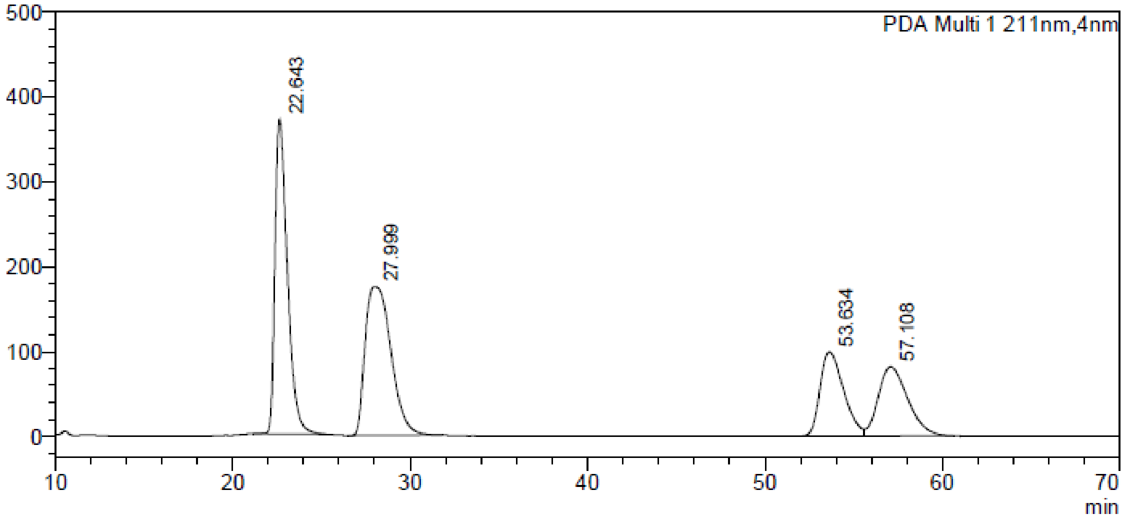

PDA Ch1 211nm

Peak#	Ret. Time	Area%
1	23.023	1.198
2	28.018	10.696
3	57.008	88.106
Total		100.000

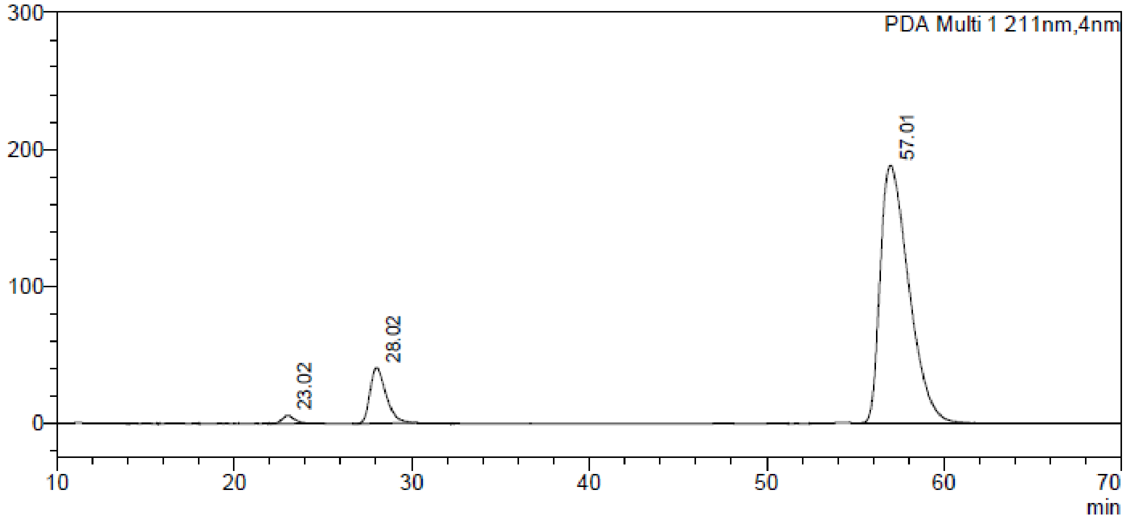

HPLC Data for **54** and *ent*-**48**: Chiralpak AD-H (95:5 hexane : IPA, flow rate 1.25 mLmin^{-1}, 211 nm, 40 °C) **54**: t_R (3*R*,4*R*): 55.3 min, t_R (3*S*,4*S*): 59.7 min, >99% ee; *ent*-**48**: t_R (3*S*,4*R*): 28.1 min, t_R (3*R*,4*S*): 38.4 min, 76% ee.

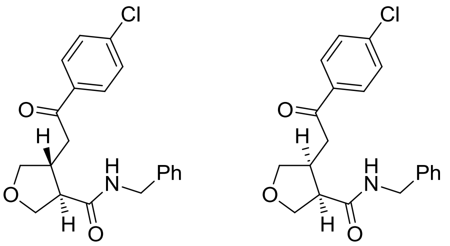

PDA Ch1 211nm

Peak#	Ret. Time	Area%
1	27.823	39.087
2	37.886	38.703
3	55.592	11.222
4	59.746	10.987
Total		100.000

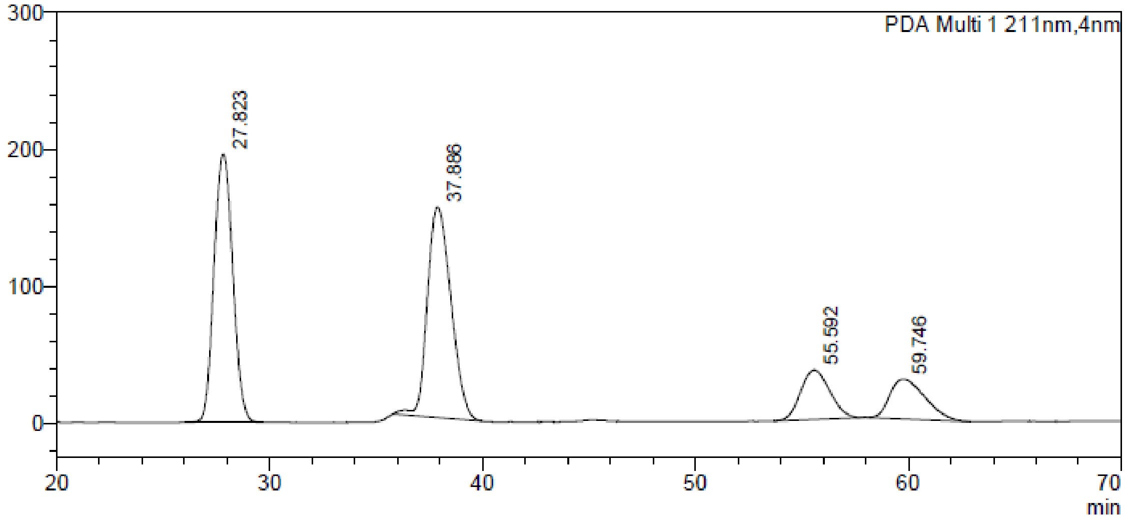

PDA Ch1 211nm

Peak#	Ret. Time	Area%
1	28.141	0.206
2	38.359	1.441
3	55.285	98.353
Total		100.000

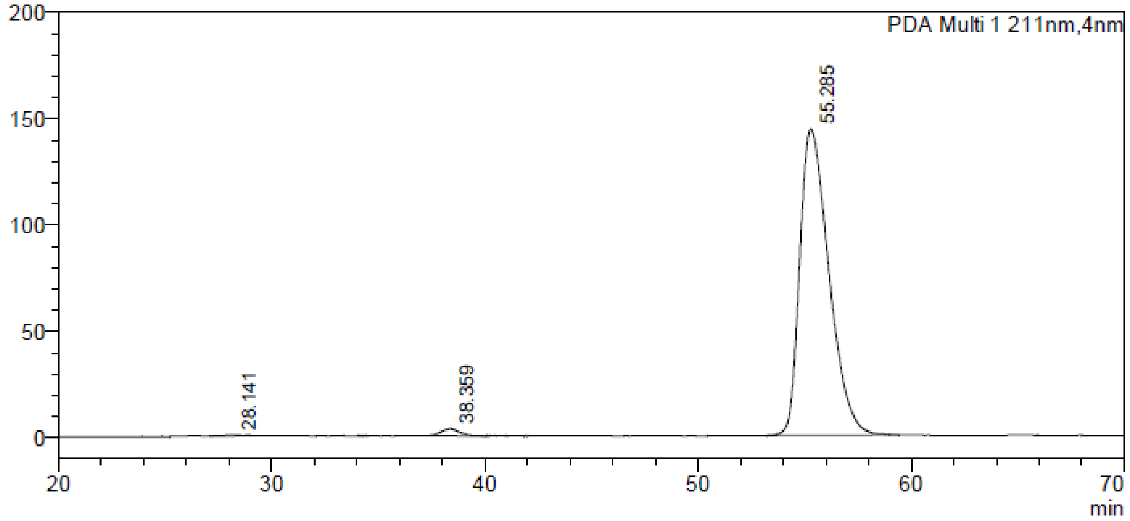

Supplement: Supplementary file 1 — miscellaneous_information [file chem0020-9762-sd1.pdf]
